# Chromosome-scale assemblies reveal the structural evolution of African cichlid genomes

**DOI:** 10.1093/gigascience/giz030

**Published:** 2019-04-03

**Authors:** Matthew A Conte, Rajesh Joshi, Emily C Moore, Sri Pratima Nandamuri, William J Gammerdinger, Reade B Roberts, Karen L Carleton, Sigbjørn Lien, Thomas D Kocher

**Affiliations:** 1 Department of Biology, University of Maryland, College Park, MD 20742, USA; 2 Centre for Integrative Genetics (CIGENE), Department of Animal and Aquacultural Sciences, Faculty of Biosciences, Norwegian University of Life Sciences, PO Box 5003, Ås, Norway; 3 Department of Biological Sciences and W. M. Keck Center for Behavioral Biology, North Carolina State University, Raleigh, NC 27695, USA

**Keywords:** genome assembly, African cichlids, comparative genomics, genome rearrangements, chromosome evolution, karyotype, inversion, recombination, transposable elements, genetic maps

## Abstract

**Background:**

African cichlid fishes are well known for their rapid radiations and are a model system for studying evolutionary processes. Here we compare multiple, high-quality, chromosome-scale genome assemblies to elucidate the genetic mechanisms underlying cichlid diversification and study how genome structure evolves in rapidly radiating lineages.

**Results:**

We re-anchored our recent assembly of the Nile tilapia (*Oreochromis niloticus*) genome using a new high-density genetic map. We also developed a new *de novo* genome assembly of the Lake Malawi cichlid, *Metriaclima zebra*, using high-coverage Pacific Biosciences sequencing, and anchored contigs to linkage groups (LGs) using 4 different genetic maps. These new anchored assemblies allow the first chromosome-scale comparisons of African cichlid genomes. Large intra-chromosomal structural differences (∼2–28 megabase pairs) among species are common, while inter-chromosomal differences are rare (<10 megabase pairs total). Placement of the centromeres within the chromosome-scale assemblies identifies large structural differences that explain many of the karyotype differences among species. Structural differences are also associated with unique patterns of recombination on sex chromosomes. Structural differences on LG9, LG11, and LG20 are associated with reduced recombination, indicative of inversions between the rock- and sand-dwelling clades of Lake Malawi cichlids. *M. zebra* has a larger number of recent transposable element insertions compared with *O. niloticus*, suggesting that several transposable element families have a higher rate of insertion in the haplochromine cichlid lineage.

**Conclusion:**

This study identifies novel structural variation among East African cichlid genomes and provides a new set of genomic resources to support research on the mechanisms driving cichlid adaptation and speciation.

## Background

African cichlid fishes, owing to their phenotypic diversity and rapid speciation over the past several million years, are a model system for studying the mechanisms of evolution [1]. Many recent studies of cichlid speciation have used short read data to perform genome scans of single-nucleotide polymorphisms (SNPs) and small insertion or deletions (indels) in order to identify genomic regions under selection [2–4]. However, there are numerous other ways that genomes can evolve, including the accumulation of larger indels, as well as intra- and inter-chromosomal rearrangements. Identification of these types of mutation requires high-quality, nearly complete genome sequences.

Draft genomes of five African cichlid species were previously generated using Illumina short-read sequencing and used in an initial analysis exploring some of the forces at play in African cichlid speciation [5]. The draft genome assembly of the Lake Malawi cichlid, *Metriaclima zebra*, was at the time one of the most continuous and accurate genomes assembled from short reads, as revealed in the Assemblathon 2 competition [6]. However, these five draft genome assemblies still contained many gaps, and only the assembly of the Nile tilapia, *Oreochromis niloticus*, was anchored to linkage groups (LGs), making it difficult to compare the structure of cichlid genomes at chromosomal scales.

To improve these cichlid genome resources, we have used long-read Pacific Biosciences (PacBio) single-molecule, real-time (SMRT) sequencing [7]. Long-read DNA sequencing technology has made it much easier to create accurate and contiguous genome assemblies [8–12]. In particular, long-read technologies have allowed the assembly of repetitive sequences and the identification of structural variants. We previously improved the genome assembly for the Lake Malawi cichlid, *M. zebra*, using 16.5× coverage of PacBio reads to fill in gaps and characterize repetitive sequences [13]. We also produced a new high-quality genome assembly of *O. niloticus*, using 44× coverage PacBio sequencing. We were able to anchor 86.9% of the assembly to LGs, which allowed us to characterize the structure of two sex determination regions in tilapias [14].

Cichlid karyotypes are highly similar among species. The diploid chromosome number (2n) varies from 32 to 60, but >60% of species have a diploid number of 48 [15]. Most of the chromosomes are acrocentric, but between 0 and 9 metacentric pairs are present in each species [16, 17]. Karyotypic changes may have played an important role in the evolution and speciation of African cichlids. Classic cytogenetic techniques are able to characterize differences in chromosome number and large fusion or translocation events, which are easily seen under the microscope. However, they are less suited to studying smaller genome rearrangements, including inversions smaller than several megabases. Comparisons of chromosome-scale assemblies in other vertebrate groups have begun to identify extensive structural differences at both the cytogenetic and the sequence assembly level [18, 19], but the role of chromosome rearrangements in recent adaptive radiations has not been well studied.

Chromosome-scale assemblies can be achieved either by physical mapping techniques [20] or by anchoring the contigs of the sequence assembly with genetic linkage maps. Genetic maps have the advantage of reflecting another important feature of genomes, namely, variation in recombination rate, which has manifold impacts on the levels of genetic polymorphism [21] and on the efficiency of genome scans [22].

Herein we describe chromosome-scale assemblies of two cichlid genomes. First, we re-anchor our previously published PacBio assembly of the *O. niloticus* genome [14] using a new high-density genetic map [23]. Second, we present a new assembly of *M. zebra* based on 65× coverage of long PacBio sequence reads. Finally, we anchor the *M. zebra* assembly with several recombination maps produced from hybrid crosses among closely related species from Lake Malawi. The anchored genome assemblies of these two species allow for this first chromosome-scale comparison of African cichlid genomes. We focus our analyses on three aspects of genome evolution that are revealed by these new chromosome-scale assemblies: variation in recombination rate across the genome, structural variation among cichlid lineages, and the landscape of transposable elements (TEs).

First, we describe the pattern of recombination along each chromosome. Spatial variation in recombination rate has implications for patterns of genetic variation [24, 25], the evolution of sex chromosomes [26], and the analysis of genome-wide associations between phenotypes and genotypes [22]. Despite the importance of recombination in shaping genome architecture [27], patterns of recombination are only beginning to be studied in cichlids [28]. A great diversity of sex chromosomes have evolved in East African cichlids, likely the result of sexual genetic conflict [29]. Rapid changes in sex determination mechanism, which are frequently variable even within species, may play an important role in cichlid speciation [1]. The evolution of new sex chromosomes often involves chromosomal inversions, which also change the pattern of recombination [30–34]. Studies of these changing patterns of recombination, and their effects on genetic variation, have been hampered by the incomplete nature of the previous draft genome assemblies.

Second, we characterize the patterns of chromosome rearrangement among species. It has been suggested that teleost karyotypes have remained largely stable since the fish-specific whole-genome duplication >300 million years ago [35]. This is in contrast to recent reports of chromosomal fusions among closely related cichlid species [36–38], and a large number of putative inversions associated with the evolution of sex chromosomes in various species [14, 32, 33]. Chromosome-scale assemblies of cichlids allow us to quantify the levels of synteny among teleost lineages, and the rate of intra-chromosomal rearrangement among cichlid lineages in East Africa. To further explore these distinct patterns of recombination and structural changes in cichlids, we also compare the cichlid genomes to the detailed genomic history of the medaka (*Oryzias latipes*). Previous studies of medaka have shown that, subsequent to the teleost-specific whole-genome duplication 320–350 million years ago, one subset of medaka chromosomes remained stable while another subset underwent more extensive fusion and translocation events [35, 39]. Related comparisons using additional teleost species have shown that the number of chromosomes is relatively stable (24–25 chromosome pairs in 58% of teleosts) except for instances in which chromosome fusion events in particular species have decreased the chromosome number [40].

Finally, we quantify the abundance and distribution of various TE families in each genome. Several studies have documented the expansion of particular transposon families in East African cichlids (AFC TEs) [41, 42]. TEs may play an important role in shaping genome architecture, particularly the divergence of sex chromosomes. TEs may also be an important source of regulatory mutations [43]. Because TEs may have been involved in the evolution of many other phenotypes, it is important that these sequences be well characterized in genome assemblies. Unfortunately, TEs are not well represented in genome assemblies that are based on short Illumina sequence reads. Our previous work has shown that long-read sequencing greatly improves both the length and quantity of TE repeats in cichlid genome assemblies [13, 14]. A comparative analysis of TEs will improve our understanding of the patterns of transposon insertion and deletion during the radiation of East African cichlids.

## Data Description

To begin this study of chromosome-scale comparisons of African cichlid genomes, we used a new high-density map of *O. niloticus* [23] to improve the anchoring of our recent genome assembly [14]. We also generated a high-quality *M. zebra* genome assembly from a single male specimen caught on Mazinzi Reef in Lake Malawi. Single-molecule PacBio sequencing was performed to 65× coverage, and a *de novo* assembly of the reads was constructed. Additional File A provides the distribution of read lengths for this new 65× coverage PacBio dataset of *M. zebra*. The mean subread length is 7,885 base pairs (bp) and the subread length N50 is 11,031 bp. Two new genetic maps are presented here based on interspecific crosses of several Lake Malawi species. These maps, along with two previously published genetic maps, were used to quality check the assembly, break misassembled contigs, and anchor the sequence contigs to chromosomes. These new anchored genome assemblies of *O. niloticus* and *M. zebra* were then aligned to one another to compare their structure. The *O. niloticus* anchored assembly and sequencing reads are available under NCBI BioProject PRJNA344471. The *M. zebra* anchored assembly and sequencing reads are available under NCBI BioProject PRJNA60369.

## Analyses

### Anchoring the *O. niloticus* assembly to a high-density linkage map

The recently assembled *O. niloticus* genome [14] was re-anchored using a new high-density map that includes 40,190 SNP markers (see Methods and [23]). This new map identified 22 additional misassemblies not identified by previous maps. Table 1 provides a comparison of the previous O_niloticus_UMD1 assembly with this newly anchored O_niloticus_UMD_NMBU assembly.

**Table 1:** Anchoring comparison of O_niloticus_UMD1 and O_niloticus_UMD_NMBU

LG	O_niloticus_UMD1 LG (bp)	O_niloticus_UMD_NMBU LG (bp)	Change (bp)
LG1	38,372,991	40,673,430	2,300,439
LG2	35,256,741	36,523,203	1,266,462
LG3	68,550,753	87,567,345	19,016,592
LG4	38,038,224	35,549,522	−2,488,702
LG5	34,628,617	39,714,817	5,086,200
LG6	44,571,662	42,433,576	−2,138,086
LG7	62,059,223	64,772,279	2,713,056
LG8	30,802,437	30,527,416	−275,021
LG9	27,519,051	35,850,837	8,331,786
LG10	32,426,571	34,704,454	2,277,883
LG11	36,466,354	39,275,952	2,809,598
LG12	41,232,431	38,600,464	−2,631,967
LG13	32,337,344	34,734,273	2,396,929
LG14	39,264,731	40,509,636	1,244,905
LG15	36,154,882	39,688,505	3,533,623
LG16	43,860,769	36,041,493	−7,819,276
LG17	40,919,683	38,839,487	−2,080,196
LG18	37,007,722	38,636,442	1,628,720
LG19	31,245,232	30,963,196	−282,036
LG20	36,767,035	37,140,374	373,339
LG22	37,011,614	39,199,643	2,188,029
LG23	44,097,196	45,655,644	1,558,448
Total anchored (%)	868,591,263 (86.0%)	907,601,988 (90.2%)	39,010,725 (4.2%)

The previous O_niloticus_UMD1 assembly anchored a total of 868.6 megabase pairs (Mbp) while the new O_niloticus_UMD_NMBU assembly anchored a total of 907.6 Mbp (90.2%). Much of the newly anchored sequence is on LG3, which increased by 19.0 Mbp, from 68.6 to 87.6 Mbp. In the O_niloticus_UMD_1 assembly, LG3 was broken into LG3a and LG3b. The new assembly merged these into a single LG3. LG3 is the largest and most repetitive chromosome in *O. niloticus* [16] and is a sex chromosome in the closely related species *Oreochromis aureus* [44]. A total of 54.7% of LG3 was annotated as repetitive, compared with 37% across the whole genome (see Methods). The repetitive nature of *O. niloticus* LG3 is also highlighted by the fact that it required this new dense map to anchor many small contigs to this LG. Several chromosomes (e.g., LG16) have fewer total bp anchored in the new assembly. This is due to the fact that misassembled contigs that have been broken according to the new map are now assigned to a different LG.

### Diploid sequence assembly of *Metriaclima zebra*

We assembled 65× coverage PacBio reads using FALCON/FALCON-unzip [8] to generate the new diploid *M. zebra* assembly, “M_zebra_UMD2.” FALCON first assembles the PacBio reads into primary contigs (p-contigs) and associate contigs (a-contigs) that correspond to alternate alleles. During the FALCON-unzip step, reads are assigned to haplotypes by phasing of heterozygous SNPs and then a final set of phased p-contigs and haplotigs are produced. Table   2 provides the assembly summary statistics for each of these assembly parts. The length of the p-contigs (total size 957 Mbp), compared with the estimated cichlid genome size of ∼1 gigabase (Gbp) pair based on Feulgen densitometry [ 45], suggests that the assembly is relatively complete. The haplotigs of this diploid assembly represent the regions of the genome that are heterozygous. Thus, for portions of the genome that are heterozygous, the diploid assembly should be represented by both a p-contig and a corresponding haplotig. If one were to align the smaller haplotigs to the larger p-contigs, one could determine which regions of this genome were heterozygous (where haplotigs align) or homozygous (where haplotigs do not align).

**Table 2:**
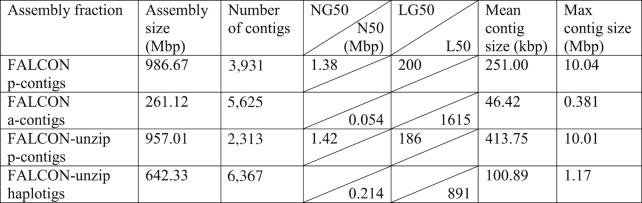
FALCON assembly results for *M. zebra*. NG50 and LG50 are based on an estimated genome size of 1 Gbp pair [45]. N50 and L50 sizes are provided for a-contigs and haplotigs because the size for the alternate haplotype is not known

### Anchoring the *M. zebra* genome assembly

Four genetic recombination maps were used to detect misassemblies, anchor contigs to chromosomes, and compare species-level structural differences. The four maps were all produced from interspecific F_2_ crosses genotyped with restriction site–associated DNA (RAD) sequencing strategies and involve six Lake Malawi cichlid species in total. The two previously generated maps were estimated using 160 F_2_ from a cross of *M. zebra* and *Metriaclima mbenjii* [46] and 262 F_2_ from a cross of *Labeotropheus fuelleborni* and *Tropheops “red cheek”* [47]. The 2 new maps consisted of crosses of *M. mbenjii* × *Aulonocara koningsi* (331 F_2_) (Emily Moore and Reade Roberts, in preparation) and *M. mbenjii* × *Aulonocara baenschi* (161 F_2_) [48]. Table 3 provides the total bp anchored to each LG for each of the four maps. The final M_zebra_UMD2 assembly anchors 760.7 Mbp.

**Table 3:** Anchoring of the *M. zebra* assembly with four different genetic linkage maps. The FALCON assembly was anchored to each map separately, and the total bases anchored are shown for each LG and map. The anchored map LGs that were used for the M_zebra_UMD2 anchoring are indicated in boldface. The *L. fuelleborni*×*T. “red cheek”* map had four LGs that were combined into two (LG10a/LG10b and LG13a/LG13b). Selection of particular LGs for the final anchoring is based on accuracy and not necessarily overall length. The total lengths including unanchored contigs differ slightly because the number of gaps (100 bp) inserted were different for each anchoring

LG	*M. zebra*×*M. mbenjii* (160 F_2_)	*L. fuelleborni*×*T. “red cheek”* (262 F_2_)	*M. mbenjii*×*A. koningsi* (331 F_2_)	*M. mbenjii*×*A. baenschi* (161 F_2_)	M_zebra_UMD2
LG1	31,191,433	32,150,205	**38,662,702**	36,192,366	38,662,702
LG2	25,783,542	28,952,651	**32,647,892**	33,362,328	32,647,892
LG3	18,498,838	14,707,016	**37,717,145**	24,847,713	37,309,556
LG4	28,418,370	24,424,243	**29,889,472**	23,743,562	30,507,480
LG5	29,725,229	34,008,850	**36,154,892**	30,984,548	36,154,892
LG6	15,868,181	32,717,361	**39,879,506**	32,438,073	39,760,669
LG7	29,333,014	57,016,972	**64,381,187**	50,973,986	64,889,811
LG8	19,307,854	16,999,744	**24,280,574**	18,082,738	23,959,896
LG9	**21**,**018**,**370**	22,620,859	18,771,712	24,011,483	21,018,370
LG10	25,942,318	26,176,893	**32,583,833**	25,149,136	32,346,187
LG11	**32**,**253**,**887**	30,903,800	34,404,464	31,577,152	32,434,411
LG12	23,231,402	31,401,442	**34,043,602**	31,595,605	34,077,077
LG13	25,893,161	24,034,634	**31,886,878**	28,831,406	32,061,881
LG14	32,750,971	32,025,991	**37,909,455**	30,978,148	37,855,742
LG15	28,015,059	28,462,857	**34,537,245**	28,405,563	34,537,245
LG16	24,665,172	26,935,058	**34,727,877**	29,158,962	34,727,877
LG17	28,473,329	31,631,813	**35,766,785**	31,607,415	35,766,785
LG18	19,927,984	23,757,304	**29,457,134**	30,047,761	29,494,144
LG19	24,076,222	19,992,035	**25,739,093**	22,726,673	25,955,740
LG20	28,281,247	30,800,769	24,975,175	**29,774,176**	29,774,176
LG22	27,460,019	31,372,369	**34,717,234**	30,512,954	34,717,234
LG23	27,069,552	27,967,022	**42,736,004**	37,848,175	42,076,657
Total anchored (%)	567,185,154 (59.3%)	629,059,888 (65.7%)	755,869,861 (79.0%)	662,849,923 (69.3%)	760,736,424 (79.5%)
Total including unanchored	957,158,042	957,163,242	957,185,442	957,167,042	957,200,631

Prior to the final anchoring, these four maps were also used to detect and confirm potential misassemblies in the FALCON contigs. Additional File B lists the FALCON p-contigs for which markers from two or more different LGs aligned, an indicator of potential inter-LG misassembly. Each of these potential misassemblies was further evaluated using alignments of a 40 kilobase pair (kbp) Illumina mate-pair library [5], RefSeq gene annotations [49], and repeat annotations (see Methods). In some cases, it was determined that the map marker sequences were repetitive, giving a false signal of misassembly. A total of 33 potential misassemblies were inspected and 16 likely misassemblies were identified and broken. An example of one of these misassemblies is provided in Additional File C. Whole-genome alignment comparisons (see section below) detected one additional intra-chromosomal misassembly at 6,922,000 on contig 000000F on LG12. This brought the final total to 17 misassemblies.

The *M. mbenjii* × *A. koningsi* map typically anchored more of the *M. zebra* assembly contigs, and in a more accurate order (i.e., greater collinearity with *O. niloticus*), than did the other three maps. This is likely due to the fact that the *M. mbenjii* × *A. koningsi* map had both more F_2_ individuals and more map markers than the other three Lake Malawi cichlid maps, giving it the highest resolution. Anchoring with the other three maps resulted in anchoring of more contigs on LG2, LG9, LG18, and LG20 (see Table   3). However, the map that produced the longest anchored LG did not always seem to be the most accurate. To determine this accuracy, each *M. zebra* LG (anchored with each of the four maps) was aligned to the anchored *O. niloticus* assembly and compared (Additional File D). The *M. zebra* × *M. mbenjii* map was chosen to anchor LG9 because it showed the most similar ordering relative to the *O. niloticus* assembly (Additional File D). The *M. zebra* × *M. mbenjii* map was also chosen to anchor LG11 because the other three maps showed large putative structural differences (Additional File D and also seen in the recombination maps, presented below). LG20 was best represented by the *M. mbenjii* × *A. baenschi* map based on alignment to *O. niloticus*, overall size, and by ordering of markers in the recombination maps. Thus, the final M_zebra_UMD2 anchoring used three of the four maps to assign, order, and orient contigs. The *L. fuelleborni*×*T*.*“red cheek”* map was not used in the final anchoring but did help confirm many misassemblies and provided information on structural differences. Several LGs have slightly different overall sizes than when the assembly was anchored with just a single map (e.g., LG3 changed from 37,717,154 to 37,309,556 bp; Table 3). This is due to the fact that several small contigs are assigned to different LGs by the four different maps. Although the final *M. zebra* anchoring is based on a combination of the four different maps, no contigs were represented multiple times in the final anchoring.

An anchoring analysis that sequentially chained together the anchored assemblies from all 4 Lake Malawi cichlid maps resulted in a slightly longer anchored assembly (833 Mbp total compared with 760 Mbp for M_zebra_UMD2). However, the ordering of contigs in this combined anchored assembly was far less accurate (when aligned to *O. niloticus*) and so it was not used. There was only a single contig longer than 1 Mbp (“000254F”) that was not anchored by at least one map.

### Minimal inter-chromosomal differences among Lake Malawi cichlid genomes

The process of anchoring the M_zebra_UMD2 assembly using the four genetic maps also allowed us to look for large structural differences among the six species used to generate the maps. Specifically, we looked for p-contigs that were assigned to different LGs in any of the four maps. Table   4 provides the list of the 9 p-contigs that were assigned to a different LG by at least one map and which represent putative inter-chromosomal rearrangements.

**Table 4:** Putative inter-chromosomal differences as identified by map anchoring comparison. The number of markers aligned to each contig for each LG is indicated in parentheses. “NA” indicates that a particular map had no markers aligned to that contig

Contig name	Contig size	*M. zebra*×*M. mbenjii* map LG (160 F_2_)	*L. fuelleborni*×*T. “red cheek”* map LG (262 F_2_)	*M. mbenjii*×*A. koningsi* map LG (331 F_2_)	*M. mbenjii*×*A. baenschi* map LG (161 F_2_)	Notes
000084F_pilon|quiver	2,383,905	LG1 (1)	LG3 (3)	LG3 (6)	LG3 (3)	
000105F_pilon|quiver_1_1312536	1,312,536	NA	LG10a (1)	LG2 (1)	LG2 (3)	
000201F_pilon|quiver	1,489,552	LG3 (1)	LG1 (3)	LG3 (3)	LG3 (1)	
000223F_pilon|quiver	1,452,516	LG8 (4)	LG8 (8)	LG3 (2)	LG8 (4)	Repetitive markers on LG3
000256F_pilon|quiver	1,241,607	LG20 (1)	LG20 (1)	NA	LG9 (1)	
000414F_pilon|quiver	805,874	LG5 (1)	LG5 (1)	NA	LG3 (1)	
000521F_pilon|quiver	566,343	LG15 (2)	NA	LG17 (1)	NA	Repetitive marker on LG17
000541F_pilon|quiver	515,490	NA	LG2 (1)	LG3 (1)	NA	
000671F_pilon|quiver	374,096	LG23 (1)	NA	LG23 (1)	LG22 (1)	

Seven of these nine contigs are anchored to a different LG in one of the maps by only a single marker. It is difficult to determine whether these represent true inter-chromosomal differences with such little evidence. Even when all nine contig anchoring differences are considered, it amounts to only 10.1 Mbp of total inter-chromosomal differences between the species used to generate the maps. It is possible that there are some other significant inter-chromosomal differences that we did not detect in the unanchored portion of the genome. If they do exist, they are likely to be highly repetitive portions of these genomes that could not be assembled into the long contigs that can be accurately anchored.

### Localization of centromeric repeats and karyotype differences

**Figure 1:**
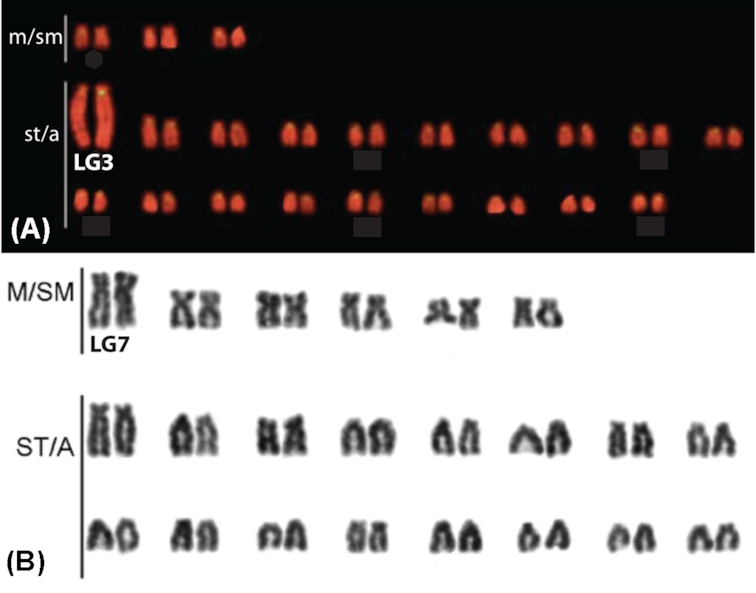
A) Chromosome mapping of ONSATA DNA reproduced and modified with permission from Ferreira et al. [16]. The SATA sequences are labelled in yellow against the background staining with propidium iodide. B) Giemsa-stained karyograms of the Lake Malawi *M. lombardoi* reproduced and modified with permission from Clark et al. [34]. LG3 in *O. niloticus* (A) and LG7 in *Metriaclima* (B) are labeled based on Mazzuchelli et al. [37].

The location of centromeres is key to understanding structural rearrangements in the karyotype. Figure 1 shows the karyotype of *O. niloticus* and *Metriaclima lombardoi* (a species closely related to *M. zebra)*. The *O. niloticus* satellite A consensus repeat (ONSATA) [50] is common to the centromeres of many East African cichlids [16] and closely matches the satellite repeats identified in a recent analysis of centromeres across many taxa [51].

*Oreochromis* and *Metriaclima* diverged 17–28 million years ago [52]. Their karyotypes each have 22 chromosome pairs, as do the majority of African cichlids, but *O. niloticus* has 1–3 meta-submetacentric and 19–21 subtelo-acrocentric chromosomes according to 2 previous karyotypes [16, 53], whereas *M. zebra* has 6 meta-submetacentric and 16 subtelo-acrocentric chromosomes. The chromosomes in Fig. 1 have been ordered by type and then by size, but only LG3 and LG7 have been assigned to the karyotypes. BAC and additional marker sequences have been used for specific labeling of chromosomes in each species [37, 54], but correspondence of chromosomes between species has not been established.

**Figure 2:**
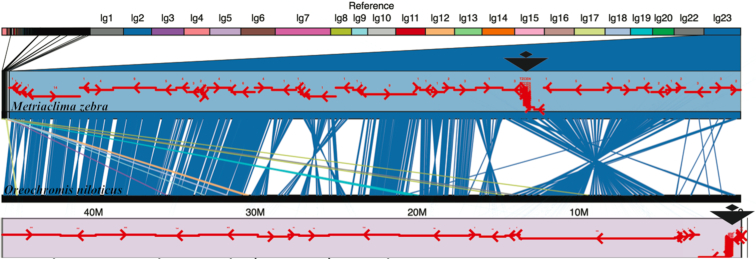
Comparative alignment of LG23 in *M. zebra* and *O. niloticus*. Centromere repeats in each assembly are indicated by large black triangles. Anchored contigs in each assembly are shown as red arrows indicating the orientation of each contig.

To understand the structural basis for these differences in karyotype, we constructed and visualized whole-genome alignments of M_zebra_UMD2 and O_niloticus_UMD_NMBU (Additional File D). Figure 2 shows the LG23 alignment of *M. zebra* and *O. niloticus*. The placement of centromere repeats identifies a large structural rearrangement on LG23 that shows that this chromosome is subtelo-acrocentric in *O. niloticus* but meta-submetacentric in *M. zebra*.

Centromere repeats were not assembled on every chromosome for either *M. zebra* and *O. niloticus*. However, on chromosomes where centromere repeats were placed in both assemblies and a large structural difference was observed, we were able to identify centromere repositioning events, including acrocentric/metacentric changes on LG3, LG16, LG17, and LG23. Although we were not able to identify the centromeres in both genome assemblies, similar rearrangement events suggest possible acrocentric/metacentric changes on LG2, LG6, LG20, and/or LG22 as well (Additional File D).

The whole-genome alignment comparisons of *M. zebra* and *O. niloticus* also identified a number of large intra-chromosomal structural rearrangements that do not directly involve the centromere. On LG2 there are two large rearrangements of ∼15 and ∼20 Mbp (Additional File D). The largest single structural change appears on LG19, where there is a ∼23-Mbp rearrangement between *M. zebra* and *O. niloticus*. A similar ∼20-Mbp rearrangement is present on LG20. There is an ∼11-Mbp rearrangement at one end of LG22 that may be associated with another change in centromere location, although the centromere was not localized on LG22 in either assembly.

Perhaps the most diverged chromosome in terms of size, structure, and repeat content is LG3. The karyotype of *O. niloticus* LG3 is much larger and more repetitive than the corresponding LG3 in Lake Malawi cichlids (Fig. 1 and [16, 53]). Additional File E shows an F_ST_ comparison of the *O. aureus* male versus female pools described in [14]. There is a very wide region of sex-patterned differentiation in *O. aureus* on LG3 from ∼40 to 85 Mbp. The large karyotype of LG3 in *O. niloticus* reflects both this large region of differentiation associated with the sex determination locus (>40 Mbp), as well as the vast amounts of repetitive sequence that have accumulated in this region.

### Variation in recombination rate among species

To compare the rates and patterns of recombination across the chromosomes, each set of map markers was aligned to the corresponding assembly and their recombination map positions plotted against physical distance. Male and female recombination in *O. niloticus* is plotted against the O_niloticus_UMD_NMBU assembly in Additional File F. Typically, the *O. niloticus* chromosomes are characterized by low recombination on the ends of chromosomes and higher recombination in the middle of chromosomes. Each of the *O. niloticus* chromosomes shows a difference in recombination between males and females. The typical pattern is higher recombination in the females than the males. However, LG6 and large parts of LG4, LG9, LG20, and LG22 show higher recombination in males than females. LG3 and LG23 are both known sex determination chromosomes in tilapias [44, 55], and each deviates from the normal recombination patterns. On LG3, there is very low recombination for ∼70 Mbp. On LG23 there is a ∼28-Mbp region of greatly reduced recombination.

**Figure 3:**
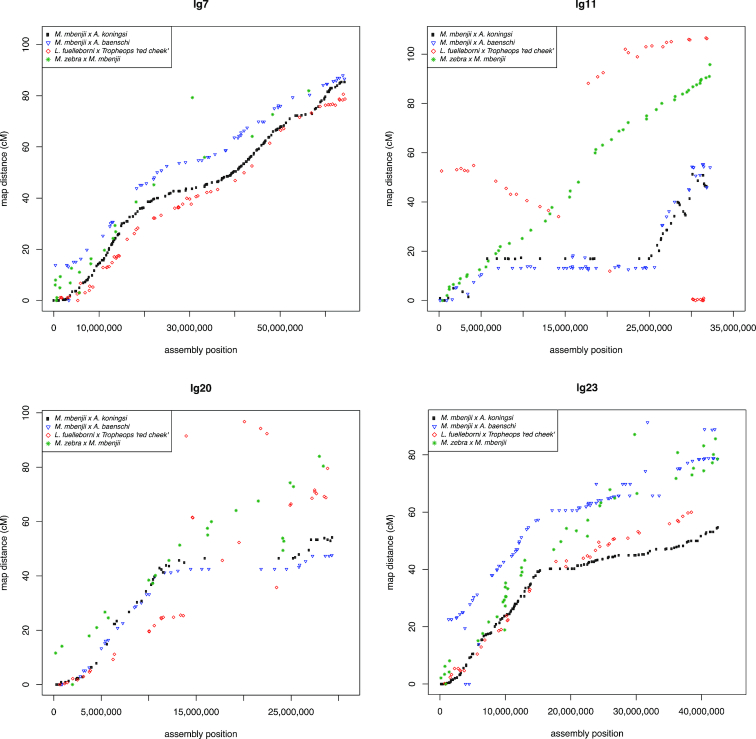
Comparison of the four genetic maps relative to M_zebra_UMD2 for LG7, LG11, LG20, and LG23. Maps for all LGs are provided in Additional File G.

Likewise, the markers in the four Lake Malawi genetic recombination maps were aligned to the final M_zebra_UMD2 assembly and their recombination map positions were plotted against physical distance. Figure   3 highlights the comparison of the four Lake Malawi genetic recombination maps relative to the M_zebra_UMD2 anchored assembly for four chromosomes. Additional File G contains plots for the other chromosomes. Similar to the *O. niloticus* chromosomes, many Lake Malawi chromosomes show low recombination on the ends of chromosomes and higher recombination in the middle of chromosomes, with several notable exceptions that are indicative of structural changes. In the Lake Malawi maps (Additional File G) there is a region of low recombination for the first ∼15 Mbp of LG2 that corresponds with a large structural rearrangement relative to *O. niloticus* (Additional File D). On LG7 (Fig. 3) the usual pattern of low recombination at the ends of the chromosomes is observed in all four maps, but there is also a region of low recombination in the middle of the chromosome (at ∼30 Mbp in M_zebra_UMD2), near several smaller scale rearrangements relative to *O. niloticus* (Additional File D). XY sex determination loci has been identified in this region of LG7 in many Lake Malawi species [ 30, 56]. There is also evidence of large structural rearrangements on LG9 in all four Lake Malawi crosses, as evidenced by both the whole-genome alignment and recombination map comparisons (Additional Files D and G). There appears to be a ∼2-Mbp inversion on LG10 (relative to *O. niloticus*) that is associated with lowered recombination around 20 Mbp in M_zebra_UMD2 (Additional Files D and G). LG11 (Fig.   3) follows the typical recombination pattern for the *M. zebra* × *M. mbenjii* map, but there appears to be a large 15-Mbp inversion in the genus *Aulonocara*, inferred from a large region of complete recombination suppression found in both the *M. mbenjii* × *A. koningsi* and *M. mbenjii* × *A. baenschi* maps. This likely corresponds to another sex locus, as has been suggested in a recent analysis of many sand-dwelling Lake Malawi cichlids [57]. Previous studies would also suggest that the *Metriaclima* species of these crosses likely contributed an XX allele [30] and the *Aulonocara* species likely contributed a heterozygous XY sex-determining allele, but this has yet to be determined. The *L. fuelleborni* × *T*.*“red cheek”* map also shows a large, but different, rearrangement on LG11 when compared with *O. niloticus*. LG15 has a region of lower recombination in the middle that is also associated with structural rearrangements relative to *O. niloticus* (Additional Files D and G). There is a large structural rearrangement on LG20 present in each of the four anchored assemblies that is also associated with a large (∼15 Mbp) region of low recombination (Fig.   3 and Additional Files D and G). Each of the four maps shows high recombination from 0 to 15 Mbp and then much lower recombination to the end of LG23, although the *M. zebra* × *M. mbenjii* map does not show as much reduction in recombination as the other 3 maps (Fig. 3). The centromere of LG23 is placed at 30.1 Mbp and is in the middle of the region of low recombination.

### Major structural rearrangements of ancient cichlid chromosomes

We also aligned the O_niloticus_UMD_NMBU assembly to the recently published “HSOK” *O. latipes* medaka assembly [39]. *O. niloticus* has 22 chromosome pairs, while the medaka HSOK genome has 24 chromosome pairs. Table 5 is a comparison of cichlid chromosomes and medaka HSOK chromosomes.

**Table 5:** Correspondence between *O. niloticus* and *O. latipes* chromosomes. Alignment lengths are provided for chromosomes with large fusion/translocation events

O_niloticus_UMD_NMBU chromosome	Primary medaka HSOK chromosome (alignment length)	Secondary medaka HSOK chromosome (alignment length)
LG1	3	
LG2	10	
LG3	18	
LG4	8	
LG5	5	
LG6	1	
LG7	6 (32 Mbp)	12 (31 Mbp)
LG8	19	
LG9	20	
LG10	14	
LG11	16	
LG12	9	
LG13	15	
LG14	13	
LG15	24 (31 Mbp)	4 (5 Mbp)
LG16	21	
LG17	23 (23 Mbp)	4 (12 Mbp)
LG18	17	
LG19	22	
LG20	7	
LG22	11	
LG23	2 (23 Mbp)	4 (17 Mbp)

**Figure 4:**
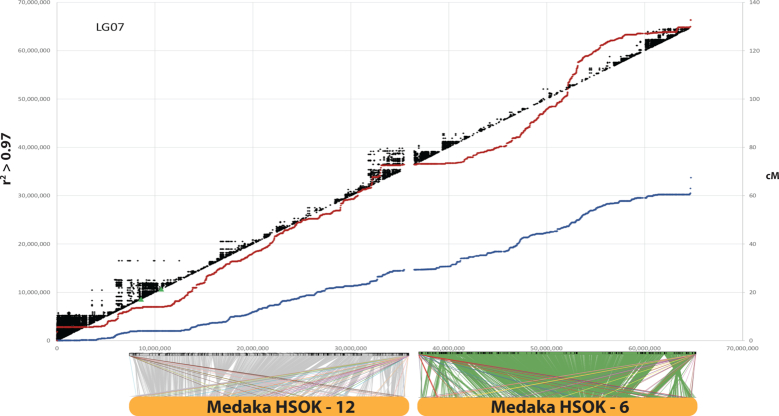
O_niloticus_UMD_NMBU LG7 is an ancient cichlid-specific fusion corresponding to medaka HSOK 12 and 6. Female (red) and male *O. niloticus* recombination curves are shown along with LD (*r*^2^ > 0.97) in black. Alignment of LG7 to medaka HSOK 12 and 6 is shown on the bottom.

**Figure 5:**
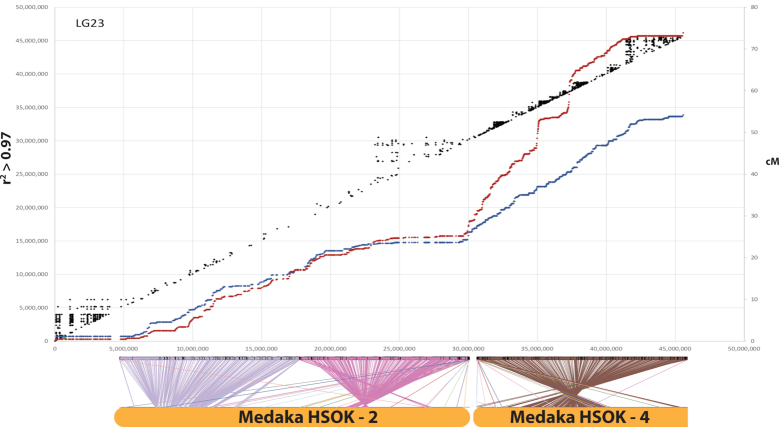
O_niloticus_UMD_NMBU LG23 is an ancient cichlid-specific fusion corresponding to medaka HSOK 2 and part of medaka HSOK 4. Female (red) and male *O. niloticus* recombination curves are shown along with LD (*r*^2^ > 0.97) in black. Alignment of LG23 to medaka HSOK 2 and 4 is shown on the bottom.

We identified several large chromosome rearrangements that occurred in a cichlid ancestor. Tilapia LG7, the second largest chromosome (Table 1), is composed of medaka chromosomes 6 and 12 in their entirety (Fig. 4). This indicates a fusion of these ancestral chromosomes in cichlids relative to medaka, as had been previously suggested [38]. Tilapia LG23, the third largest chromosome (Table 1), is composed of medaka chromosome 2 in its entirety and 17 Mbp, or roughly half, of medaka chromosome 4 (Fig.   5). The other half of medaka chromosome 4 was likely translocated onto LG15 and LG17. While the remaining 18 chromosomes have undergone extensive intra-chromosomal rearrangements, they have largely maintained a correspondence to individual medaka chromosomes over the course of the 120 million years of evolution since the last common ancestor of these species.

While LG3 is the largest tilapia chromosome (Table 1), it surprisingly does not show any evidence of a chromosomal fusion or translocation event. Tilapia LG3 aligns well to medaka chromosome 18 along the first ∼30 Mbp of LG3, and the remainder of LG3 aligns to medaka chromosome 18 with much less contiguity.

**Figure 6:**
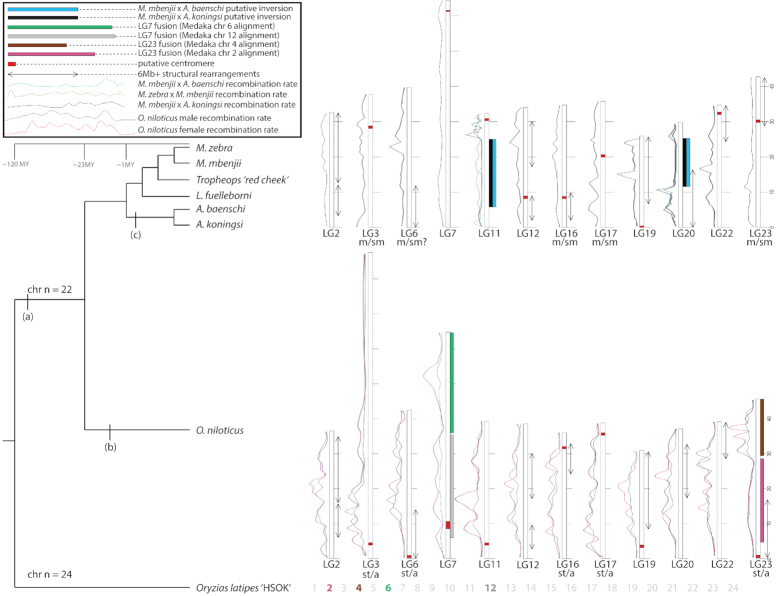
Summary of large structural changes in African cichlid genomes. (a) Chromosome fusion events on LG7 and LG23. (b) Expansion of repetitive LG3 in the *Oreochromis* lineage likely in conjunction with its role as ZW sex chromosome. (c) Putative inversions in *Aulonocara* on LG11 and LG20. Chromosomes that have undergone a large (>6 Mbp) structural change are displayed. Other chromosomes that have not undergone a large change in the seven cichlid species studied are not shown. Likely changes in meta-/sub-metacentric (“m/sm”) and subtelomeric/acrocentric (“st/a”) chromosomes from Malawi and *O. niloticus* are labeled. Recombination rates are shown as LOESS smoothed curves. Male and female recombination rate curves are shown for *O. niloticus*. Typical recombination rate curves for Lake Malawi cichlids are usually represented by the *M. mbenjii* × *A. koningsi* map. Recombination curves in crosses involving *Aulonocara* are shown for LG11 and LG20 to highlight large differences in recombination on those particular chromosomes. Several rearrangements, such as LG2, are more complex than depicted in this figure. Refer to Additional File D for detailed whole-genome alignments and Additional Files F and G for detailed recombination plots. Divergence times were obtained from Kumar et al. [52].

Figure 6 provides a summary of the major structural features in the evolution of cichlid chromosomes including recombination rates, putative centromeres, karyotype differences, fusions, and large inversions >6 Mbp. The details of each of these chromosomal features can be found in Additional Files D, F, and G.

### Linkage disequilibrium

There is substantial linkage disequilibrium (LD) over extended physical distances in the tilapia GST® population (see Methods), as shown in Figs   4 and 5. As expected, the regions of low recombination near the ends of the chromosome show the highest levels of LD. Large blocks of LD are also evident around the centromere on LG15 (Additional File F) and in the low-recombination regions associated with the ancestral chromosome fusions on LG7 (Fig. 4) and LG23 (Fig. 5).

### Repeat landscape of the *Metriaclima zebra* assembly

**Figure 7:**
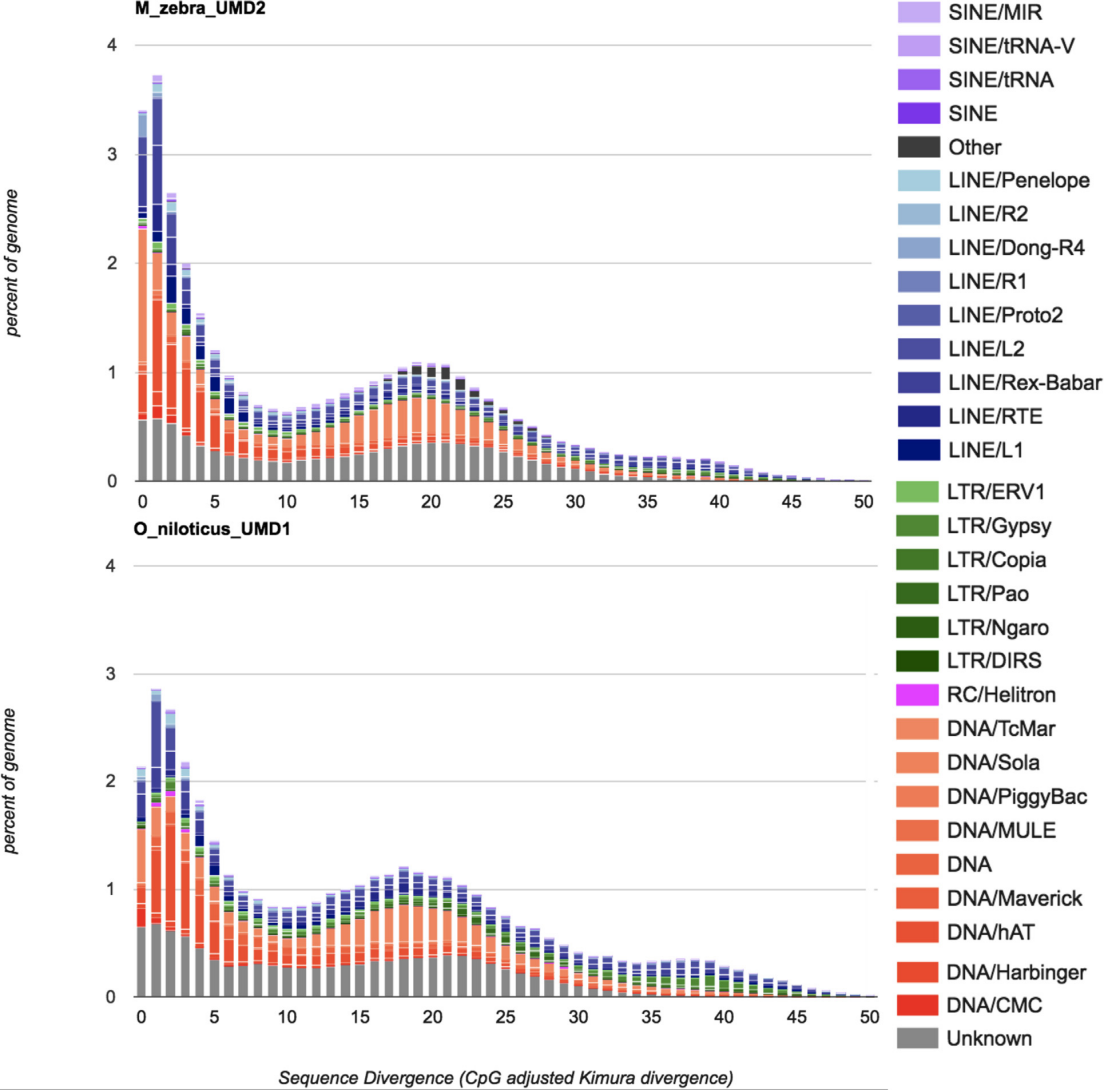
Comparison of the repeat landscape in the *M. zebra* and *O. niloticus* genome assemblies.

The M_zebra_UMD2 assembly is 35% repetitive, similar to the O_niloticus_UMD1 assembly, which is 37% repetitive [14]. Figure 7 shows the repeat landscape for the *M. zebra* and *O. niloticus* assemblies. While the *O. niloticus* genome assembly does have a slightly larger total quantity of annotated repeats, the *M. zebra* genome assembly has a noticeably larger amount of recent TE insertions (sequence divergence <2%). To further test that this difference was not an artifact of the two different assembly processes, we assembled the *M. zebra* PacBio reads at the same 44× coverage as the *O. niloticus* assembly. A comparison of the read length distribution of the 44× subsampled *M. zebra* read dataset and the original 44× *O. niloticus* read dataset is provided in Additional File H. This subsampled 44× *M. zebra* assembly was performed with the same parameters, using the same version of Canu as was performed for the O_niloticus_UMD1 assembly. RepeatMasker was subsequently run on this assembly, and the pattern of more recent insertion in *M. zebra* relative to *O. niloticus* was even more pronounced (Additional File I). The reason it is more pronounced is likely due to differences in the output of repetitive regions between the FALCON and Canu assemblers.

Three TE families account for most of the difference in the recent TE activity between the two species. Recent insertions (defined as 0–1% sequence divergence) of the class II DNA transposon superfamily Tc1-Mariner make up 0.5% of the total O_niloticus_UMD1 assembly but make up 1.3% of the M_zebra_UMD2 assembly. Recent insertions of another class II DNA transposon superfamily, hAT, make up 0.15% of the O_niloticus_UMD1 but make up 0.45% of the M_zebra_UMD2 assembly. Recent insertions of the class I retrotransposon superfamily, long interspersed nuclear element (LINE)-Rex-Babar, make up 0.2% of the O_niloticus_UMD1 assembly but make up 0.6% of the M_zebra_UMD2 assembly. Other TE superfamilies show smaller increases in *M. zebra* as well. This indicates that *M. zebra*, and perhaps Lake Malawi cichlids in general, have experienced more recent TE expansion than the *O. niloticus* lineage.

The insertion locations (with respect to gene structure) of these three most abundant TE superfamilies were categorized by defining promoters as either 1 or 15 kbp upstream of transcriptional start sites and summarized (Additional File J). The LINE-Rex and DNA-TcMar superfamilies both have an increased amount of TE insertion in the 15-kbp promoter regions of *M. zebra* compared with *O. niloticus* (1,422 and 338, respectively), although there are fewer DNA-hAT elements present in the *M. zebra* promoters compared with *O. niloticus*. There is an increase of these recent TE superfamilies in intronic and intergenic regions, with the LINE-Rex elements having the largest increase in intronic regions (1,376 additional intronic insertions) and DNA-hAT having the largest increase in intergenic regions of *M. zebra* compared with *O. niloticus*. Similar overall patterns of insertion exist when considering a 1-kbp promoter, except for DNA-TcMar, where slightly fewer 1-kbp promoter insertions were found in *M. zebra* than in *O. niloticu*s.

Overall, the amount of TEs assembled has increased from the original Illumina-only–based *M. zebra* assembly [5], to the moderate PacBio coverage gap–filled M_zebra_UMD1 assembly [13], to the high PacBio coverage M_zebra_UMD2 assembly. Additional File K provides a comparison of repeat landscapes for each of these three *M. zebra* assemblies. The overall number of TEs, and particularly the most recently inserted TEs, are better represented as the assemblies improve. The African cichlid–specific AFC-SINEs (short interspersed nuclear elements) and AFC-LINEs [58] have been assembled in greater length as well. For example, the ∼7.1-kbp “L1-1_AFC” LINE was assembled into 2,874 copies (across 1.29 Mbp) in the original M_zebra_v0 assembly, 1,350 copies (across 1.66 Mbp) in the M_zebra_UMD1 assembly, and 2,295 copies (across 4.77 Mbp) in the new M_zebra_UMD2 assembly.

### Genome completeness and annotation

Benchmarking Universal Single-Copy Orthologs (BUSCO) [59, 60] was used to assess the completeness of the new *M. zebra* genome assembly. A total of 2,586 complete vertebrate BUSCOs were searched and 2,465 (95.3%) complete BUSCOs were found, of which 71 (2.7%) were duplicated and 2,394 were single-copy. Only 82 (3.2%) were reported as fragmented, and just 39 (1.5%) BUSCOs were reported as missing.

The M_zebra_UMD2 assembly was annotated using the NCBI RefSeq annotation pipeline for eukaryotic genomes [49]. Table 6 shows the improvement in gene annotation for the new M_zebra_UMD2 assembly relative to the previous version of the *M. zebra* assembly [5, 13].

**Table 6:** Annotation improvement of the M_zebra_UMD2 assembly gathered from RefSeq annotation reports [61, 62]

Feature	M_zebra_UMD1	M_zebra_UMD2	Difference (%)
**Genes and pseudogenes**	27,328	32,471	5,143 (18.8)
Protein coding	24,290	25,898	1,608 (6.6)
Non-coding	2,468	5,149	2,681 (108.6)
Pseudogenes	443	1,238	795 (179.5)
**mRNAs**	44,123	46,160	2,037 (4.6)
Fully supported	41,957	43,159	1,202 (2.9)
Partial	1,184	655	−529 (−44.7)
With filled gaps	796	246	−550 (−69.1)
Known RefSeq (NM_)	9	12	3 (33.3)
Model RefSeq (XM_)	44,114	46,148	2,034 (4.6)
**Non-coding RNAs**	3,192	6,209	3,017 (94.5)
Fully supported	2,228	4,047	1,819 (81.6)
Model RefSeq (XR_)	2,518	4,851	2,333 (92.7)
**CDSs**	44,263	46,358	2,095 (4.7)
Fully supported	41,957	43,159	1,202 (2.9)
Partial	1,055	654	−401 (−38.0)
With major corrections	358	478	120 (33.5)
Known RefSeq (NP_)	9	12	3 (33.3)
Model RefSeq (XP_)	44,127	46,161	2,034 (4.6)

## Discussion

### Anchoring to produce chromosome-scale assemblies

The genetic recombination maps and whole-genome alignment comparisons to the *O. niloticus* assembly were very useful for identifying large and mostly inter-chromosomal misassemblies in the new *M. zebra* assembly. A 40-kbp Illumina jumping library was also used in this process to determine whether disagreements between the maps and the assembly were true misassemblies, errors in the maps, or structural differences between samples. It is likely that several misassemblies still remain in the final M_zebra_UMD2 anchoring. However, these potential misassemblies are probably only present on smaller contigs where there were not enough markers to detect misassembly events. Only one contig longer than 1 Mbp was not anchored by two or more markers from one of the four Lake Malawi maps. Therefore, any possible remaining misassemblies are likely to involve smaller contigs. A high-density map of *M. zebra* would be a useful resource for future studies.

### Patterns of continuity in genome assemblies

The longest contigs tend to be anchored in the middle of chromosomes and in regions where there is greater recombination. The ends of chromosomes, typically in regions of lower recombination, tend to have smaller contigs. Perhaps the clearest example of this is on LG13 (Additional Files D and G). On LG7, smaller contigs appear in the middle of the chromosome where there is also a reduction in recombination uncharacteristic of most other chromosomes. Smaller contigs likely correspond to regions with a large fraction of repetitive sequence that lead to a more fragmented assembly. These regions have likely accumulated large TE arrays that are not spanned by even the longest of the reads in our datasets. It is known that TEs accumulate in regions of suppressed recombination [63]. These chromosomal regions with smaller contigs also tend to have more structural rearrangements relative to *O. niloticus*, which suggests an important role for TEs in formation of the rearrangements. The role of various TE families in the formation of genomic rearrangements has been previously demonstrated in a variety of organisms [64–68]. This pattern could also be caused by ambiguities in the maps due to there being fewer recombination events and therefore less map resolution in these regions. There are also fewer markers used to anchor smaller contigs, which may also contribute to this pattern. Longer read lengths and alternative mapping technologies, such as optical mapping and Hi-C, may complement the genetic recombination maps and be useful for defining the structure of these regions in finer detail.

### Patterns of recombination in *O. niloticus*

Several patterns are evident in the recombination maps for *O. niloticus*. First, although the pattern of recombination is generally similar in males and females, the level of recombination in females is generally higher than in males. The total female map length is 1,641 cM, while the male map is only 1,321 cM. The sex differences in recombination rate of *O. niloticus* are smaller than observed in salmonids [69–72], stickleback [73], Japanese flounder [74], and zebrafish [75]. Second, the pattern of recombination on each chromosome is usually sigmoidal, with relatively little recombination over ∼5 Mbp at the ends of each chromosome. The highest levels of recombination are found in the middle of each chromosome. This pattern is exactly opposite the pattern observed in stickleback and catfish, where recombination is highest at the ends of the chromosomes [76, 77].

These patterns of recombination have implications for the pattern of LD along each chromosome, which varies substantially across the genome. Blocks of LD are much longer in the regions of low recombination (Figs 4 and 5 and Additional File F), such as near the ends of each chromosome. Regions of low recombination tend to accumulate repetitive TEs [63]. These regions are also likely to experience episodes of genetic hitchhiking, which will alter the pattern of genetic differentiation among populations across the genome, as shown in stickleback [73, 76]. The extent of LD affects the probability of fixation of adaptive variants and may affect the probability that a given chromosomal segment can evolve into a new sex chromosome [73]. Interestingly, extensive LD is present on LG3 in *O. niloticus*. One evolutionary interpretation of this finding is that high LD on LG3 predated, and facilitated evolution of, the LG3 sex chromosome present in *O. aureus* [44]. Alternatively, recombination suppression may have evolved as a result of sex-chromosome–associated evolution at LG3; in this scenario, the lineage leading to *O. niloticus* may have had, and subsequently lost, the dominant LG3 sex determination allele, but the traces of sex chromosome evolution remain in the genome.

### Patterns of recombination in Lake Malawi cichlids

The four genetic maps of Lake Malawi cichlids show the same general pattern of recombination as *O. niloticus*. Again, the pattern of recombination on most Lake Malawi chromosomes is characterized by low recombination at the ends of the chromosomes and high recombination in the middle of the chromosomes. The several exceptions all indicate lineage-specific, intra-chromosomal rearrangements among the Lake Malawi species.

Perhaps the most striking difference between these four maps is a large (∼19 Mbp) putative inversion on LG11 in *Aulonocara*, as evidenced by the lack of recombination in the *M. mbenjii* × *A. koningsi* and *M. mbenjii* × *A. baenschi* maps (Fig. 3). This putative inversion on LG11 likely corresponds to the same LG11 region recently reported to be associated with bower-building behavior in sand-dwelling cichlids [57]. Large putative inversions and regions of low or no recombination are also evident on LG2, LG9, and LG20 (Fig. 3 and Additional File G). As additional genetic maps of other African cichlids are developed, this framework can be used to see what additional variation in recombination and structure exists and what can be learned from it.

### Patterns of recombination on sex chromosomes

Sex chromosomes typically accumulate inversions that reduce recombination between the sex-determining gene and linked sexually antagonistic alleles [78]. The strain of *O. niloticus* used to generate the genome assembly contigs [14] has an XY sex determination locus on LG1 [32, 79]. The strain of *O. niloticus* used to generate the map [23] and anchor those contigs to chromosomes has an XY sex determination locus on LG23 [80]. We observed reduced recombination in males relative to females adjacent to the sex locus at 34.5 Mbp on *O. niloticus* LG23 (Additional File F). As previously mentioned, LG3 carries a ZW sex locus in several species of *Oreochromis* [14, 44] but not in the *O. niloticus* line assembled here. The ∼70-Mbp sex interval (Additional File E) is associated with the large reduction in recombination of both males and females (Additional File F). We also observed substantial differences in recombination between the sexes on LG7, LG11, LG14, and LG15. An XY sex locus has been identified on LG14 in *Oreochromis mossambicus* [81], and XY sex loci have been identified on LG7 [30, 56] and LG11 (Thomas Kocher, unpublished data) in Lake Malawi cichlids. Notably, alleles of the LG7 XY sex determination system segregate in three of the four Lake Malawi crosses (the *M. mbenjii* × *A. baenschi* cross is unknown) (47, **82**, and Tom Kocher - unpublished results ). However, LG7 shows relatively low recombination suppression compared with some other chromosomes. Recombination is reduced in the middle of LG7, centered at ∼32 Mbp, but this is not associated with the centromere (located at 61 Mbp). While this region is near the LG7 XY sex determination interval, the overall shape of recombination on LG7 is likely the result of the chromosome fusion event that occurred in the cichlid ancestor (Fig.   4 and discussed below). As discussed for *Oreochromis* above, it is unclear whether recombination suppression or sex determination evolved first at this locus. It should also be noted that there is a single marker in this region that appears out of order in the *M. zebra* × *M. mbenjii* map, perhaps indicating a structural difference (Additional File G and Fig. 3). Further investigation will be needed to determine whether other regions of the genome that display large differences in sex-specific recombination are associated with previously identified and/or novel sex determination loci.

### Conservation of ancient synteny

Synteny is remarkably conserved among even distantly related teleosts [40, 83]. Medaka show few inter-chromosomal rearrangements since shortly after the fish-specific whole-genome duplication >300 million years ago [35]. Our whole-genome alignment of tilapia to medaka supports the previously reported findings that the syntenic organization of teleost genomes is largely stable. The ancestral teleost chromosome number was 24 pairs [40]. In cichlids, where 22 chromosome pairs is most common [17], we find evidence for two large fusion events on LG7 and LG23 (Figs   4 and 5). Clearly, the variation in diploid number observed in other cichlid species implies that there have been additional inter-chromosomal rearrangements, but we predict that these will be simple fission/fusion events and not the result of scrambling of these ancient syntenic relationships.

The patterns of recombination across these particular chromosomes provide additional evidence of fusion and translocation events (Figs 4 and 5). There are large deviations from the slope of the recombination curves located precisely where these fusion and translocation events have occurred. This also suggests that the pattern of recombination evolves slowly because these oddly shaped recombination patterns have persisted for ≥15 million years since the divergence of the common ancestor of *O. niloticus* and the Lake Malawi species. Interestingly, although LG3 is the longest *O. niloticus* chromosome and has an odd pattern of recombination, LG3 does not seem to be the result of a chromosome fusion event. This lends support to the hypothesis that the size of LG3 is due to accumulated repetitive sequences after LG3 became a sex chromosome, and that this sex chromosome signature and associated recombination suppression persists in *O. niloticus* even following loss of the LG3 sex determination system.

There are many examples of large-scale (>2 Mbp) intra-chromosomal rearrangements between *O. niloticus* and Lake Malawi cichlids, as well as rearrangements evident among the Lake Malawi species. In some cases, the anchoring of the *M. zebra* assembly using each map showed the same large structural rearrangement relative to *O. niloticus* for each map (see LG2, LG19, LG20 in Additional File D). This suggests that these rearrangements happened prior to the Lake Malawi radiation or are specific to *O. niloticus*. In other cases, there are large structural differences relative to *O. niloticus* that are different among the four maps (LG12, Additional File D), which suggests that these rearrangements occurred during the radiation in Lake Malawi. For example, on LG11, the *M. zebra* × *M. mbenjii* map is mostly colinear with *O. niloticus*, but the other three maps show a large rearrangement and some differences in the order of this rearrangement. LG9 of *M. zebra* was particularly difficult to anchor with the *M. mbenjii* × *A. koningsi* map (Table 3). Additional work is needed to better define the structure of these chromosomes in each lineage.

### Evolution of centromere position and sequence

Long-read sequencing has made it possible to assemble centromere repeats [84–86]. A recent study of centromere evolution in medaka provides an example of the role of centromere evolution in speciation [39]. The study showed that the centromere position of many medaka chromosomes has remained unchanged among *Oryzias* species in both acrocentric and non-acrocentric chromosomes. In other chromosomes, the position of centromeres did change and sometimes these chromosomes underwent major structural rearrangements involving other chromosomes. Alignment of the O_niloticus_UMD_NMBU assembly to these new medaka assemblies showed that cichlids have a different set of conserved and variable chromosomes compared with medaka. Additionally, the medaka study showed that centromere sequence repeats were more conserved in the chromosomes that remained acrocentric than in chromosomes that switched between acro- and non-acrocentric or that were non-acrocentric. Assembly and placement of cichlid centromere repeats in multiple species will provide insight into centromere evolution at the sequence level. Are there differences in centromere sequence/rate of evolution between acrocentric and non-acrocentric chromosomes? Are these differences great enough to create meiotic incompatibilities in hybrids? Are the positions of centromeres conserved across many species? This study provides a starting point to answer these questions.

### Evolutionary patterns of African cichlid karyotypes

The karyotypes of *O. niloticus* and *M. zebra* in Fig. 1 show that there have been at least five changes from subtelo-acrocentric to meta-submetacentric chromosomes. The clearest example of this is the 15-Mbp rearrangement on LG23 (Fig.   2). The ONSATA and the TZSAT (*Tilapia zillii* satellite repeat) satellite sequences [87] have not been explicitly shown to be centromeric binding sequences but rather are highly associated with the centromeres via *in situ* hybridization [16]. We were able to identify these ONSATA and TZSAT centromere-containing repeats on both the *M. zebra* and *O. niloticus* assemblies in just over half of the chromosomes (LGs 3, 4, 5, 7, 8, 9, 11, 13, 14, 16, 17, 19, 23). It is possible that these ONSATA and TZSAT repeat sequences may be present in other portions of the chromosome or that some of them have been assembled incorrectly. Indeed, there are several chromosomes where the ONSATA and TZSAT repeats were identified in multiple distant locations along the chromosome in one or both assemblies (LG6, LG16, LG17, LG19).

Two of the chromosomes with identifiable karyotype changes have also been shown to harbor sex-determining loci in African cichlids. One is the previously mentioned XY sex determination region in *O. niloticus* on LG23 [80] and the ZW sex determination region on LG3 in *O. aureus* (Additional File E) [14], which corresponds to a low-recombination region in male and female *O. niloticus*. The assembled and anchored chromosomes support the karyotypes (Fig. 1) because the largest *O. niloticus* assembled chromosome is LG3 and the largest *M. zebra* chromosome is LG7 (Tables 1 and 3). We suggest that LG3 expanded in the *O. niloticus* lineage by means of the accumulation of a large amount of TEs and segmental duplications, likely while linked to sex determination in a basal *Oreochromis* [14]. It is not clear whether this apparent runaway elongation of LG3 in *Oreochromis* is due to suppressed recombination of a sex-determination locus or some other mechanism. Additional genome assemblies of similar quality in related *Oreochromis* species should allow for further refinement of the evolutionary history of this large sex chromosome in the Oreochromini.

There is also a large (∼28 Mbp) region of greatly reduced recombination on LG23 in the *O. niloticus* map, as well as in each of the four Lake Malawi maps. LG23 is also the second largest anchored chromosome in the *M. zebra* assembly and third largest chromosome in the *O. niloticus* assembly. It is possible that this arm of LG23 is accumulating TEs similar to LG3, but at an earlier stage. There is an XY sex determination locus on LG23 in *O. niloticus* [55, 80], and in at least one species of Lake Victoria cichlid [88], which may be contributing to changes in the size and rate of recombination on this chromosome. Three scenarios may explain these observations: (i) LG23 is an ancient sex chromosome, and though lost in the Malawi lineage, associated recombination suppression remains in Lake Malawi cichlids; (ii) the LG23 sex determination locus is indeed segregating in Lake Malawi cichlids but has yet to be identified and described; or (iii) the recombination pattern on LG23 is not due to sex chromosome–associated evolution but has been maintained by unknown factors in both lineages.

While many chromosomes have shown extensive rearrangement, it should also be noted that several chromosomes have undergone very little change since the divergence of *M. zebra* and *O. niloticus*. Other than relatively small structural changes at the ends of chromosomes, conserved synteny seems to have been maintained across the entire length of LGs 13, 14, 17, and 18 (Additional File D). It is possible that selective pressures have acted to maintain the synteny of these chromosomes. Because 20% of the *M. zebra* and 10% of the *O. niloticus* genome assemblies remain unanchored, future studies may provide additional structural insights. For example, LG9 in *M. zebra* remains under-anchored. Future *in situ* and physical mapping studies should confirm these results in *O. niloticus* and *M. zebra*. Our work will greatly inform fine-scale cytogenetic studies aimed at characterizing intra-chromosomal differences among cichlid species.

### Recent TE expansion in *M. zebra*

TEs have been shown to modulate gene regulatory networks, especially when they insert in regulatory promoters and introns [64, 65]. In cichlids, recent evidence has shown that AFC-SINE indels in *cis*-regulatory regions of genes are associated with innovative cichlid phenotypes such as egg-spots [89]. A deletion that may be TE mediated is responsible for controlling the expression of the *SWS2A* opsin [90]. It is likely that other AFC-specific and TE-mediated mutations have contributed to the diverse phenotypes of African cichlids. Therefore, it is important that these TE insertion events be well represented in genome assemblies.

The present study has found that *M. zebra* has a higher number of recent TE insertions (sequence divergence <2%) than *O. niloticus* (Fig. 7 and Additional File I) and that many recent TE insertions occur in both promoter and intron regions (Additional File J). It remains to be seen whether these recent TEs have been co-opted to alter gene regulatory networks and have played a large role in generating phenotypic diversity of African cichlids.

Because the *O. niloticus* assembly is 43.4 Mbp longer than the *M. zebra* assembly, it is possible that the rate of recent TE insertions is even greater than we have quantified here. We present this finding with several caveats. It is possible that the two species have divergent patterns of insertion across the genome. We previously suggested that *O. niloticus* contains larger clusters of repeat arrays that are experiencing recent insertions [14]. These very long arrays do not seem to be present at the same frequency in the *M. zebra* genome. It is possible that many recent TE insertions in *O. niloticus* were not assembled completely and remain hidden in these large arrays. Differences in effective population size (*N_e_*) between the two species may also account for differences in the rate of TE accumulation because larger populations will be able to purge deleterious insertions more efficiently. Other unknown technical factors may also have contributed to the difference that we have described. Future comparisons of additional samples and species assembled using the same sequencing coverage and assembly software/parameters will help to more accurately quantify the recent TE expansion in African Great Lake cichlids.

### Diploid assembly

We present the new *M. zebra* assembly in both haploid and diploid representations. The majority of current genomics tools assume a haploid reference assembly and all subsequent analyses are based on this haploid representation. The use of multiple diploid assemblies will be required to capture population-level patterns of heterozygosity and complex structural variation. The genome assemblies reported here should therefore be considered the beginning of a larger effort to properly represent cichlid genomes. A study of *Arabidopsis thaliana* and *Vitis vinifera* (Cabernet Sauvignon) showed that the phased diploid assemblies produced by FALCON-unzip improved identification of haplotype structure and heterozygous structural variation [8]. Sequencing and assembly of F_1_ in cattle has also been shown to recover these complex regions better and may be the way forward for assembly of diploid genomes [91]. Graph genome representations [92, 93] have been shown to improve variant calling in complex regions such as the human leukocyte antigen [94], major histocompatibility complex [95], and centromeres [96]. Additional long-read diploid assemblies will be able to better represent genetic variation, particularly in regions of complex variation that current long-read assemblies are beginning to span [85].

## Potential Implications

This study highlights the evolutionary insights that can be gained using a comparison of high-quality chromosome-scale genome assemblies, genetic recombination maps, and cytogenetics across multiple related and, in this case, rapidly evolving species. It further illustrates the need for high-quality, chromosome-scale genome assemblies for answering many basic biological questions. This study illustrates the structural changes that can occur in the genomes of a rapidly evolving clade. It will be interesting to make comparisons to other radiations in the tree of life, both large and small. The present study provides a wide-angle view of African cichlid genome history (summarized in Fig. 6) and demonstrates how these high-quality resources can be used for many different types of evolutionary genomic analyses. As additional high-quality cichlid genomes are generated, this study will provide the foundation for comparisons of structural variation, recombination, cytogenetics, and repetitive sequences across the cichlid phylogeny. Many new questions have been generated here. How do the structural changes of African cichlid genomes compare to other groups? Is the pattern of few inter-chromosomal, but many intra-chromosomal differences seen here found in additional Lake Malawi genera as well as other radiations in Lake Tanganyika and Lake Victoria? Are these patterns of recombination observed across the majority of cichlids? Are any deviations from these typical recombination patterns related to specific phenotypic traits or sex chromosome history? How have these chromosomes evolved structurally? We look forward to the new dawn in cichlid genomics.

## Methods

### *O. niloticus* SNP array map, misassembly detection, and new anchoring

Offspring (n = 689) and parents from 41 full-sibling families belonging to the 20th, 24th, and 25th generations of the GST® strain were analyzed using a custom 57K SNP Axiom Nile Tilapia Genotyping Array (Affymetrix, Santa Clara, CA, USA) [23]. SNPs classified as “PolyHighRes” or “No-MinorHom” by Axiom Analysis Suite (Affymetrix, Santa Clara, CA, USA), and having a minor-allele frequency ≥0.05 and call rate ≥0.85 were used in genetic map construction (*n* = 40,548). Lep-MAP2 [97] was used to order these SNPs into LGs in a stepwise process beginning with SNPs being assigned to LGs using the “SeparateChromosomes” command. Logarithm of the odds (LOD) thresholds were adjusted until 22 LGs were generated, which correspond with the *O. niloticus* karyotype. Unassigned SNPs were subsequently added to LGs using the “JoinSingles” command and a more relaxed LOD threshold, and ordered within each LG using the “OrderMarkers” command.

Sequence flanking each SNP (2 × 35 nucleotides) was used to precisely position 40,190 SNPs to the O_niloticus_UMD1 assembly (NCBI accession MKQE00000000) and thereby integrate the linkage and physical maps. This revealed 22 additional contig misassemblies (i.e., contigs containing SNPs from different LGs) that were not detected in the original anchoring for O_niloticus_UMD1. These contigs were subsequently broken. Linkage information was subsequently used to order and orient contigs and build sequences for 22 Nile tilapia LGs in the new O_niloticus_UMD_NMBU assembly following the previous cichlid nomenclature [5, 14, 54, 98].

LD results (*r*^2^ > 0.97) presented in Figs 4 and 5 and Additional file F were produced in PLINK2 version 1.90b3w [99] using the pedigree described above and SNP positions given in [23].

### PacBio Sequencing of *M. zebra*

The previous version of the *M. zebra* assembly, M_zebra_UMD1 [13], included 16.5× PacBio sequencing (25 SMRT cells using the P5-C3 chemistry) on a PacBio RS II machine [13]. An additional library was prepared using the same Qiagen MagAttract HMW DNA extraction and Blue Pippin pulsed-field gel electrophoresis size selection. An additional 60 SMRT cells (using the P6-C4 chemistry) were sequenced on the same PacBio RS II at the University of Maryland Genomics Resource Center as the previous 16.5× P5-C3 data. These P6-C4 SMRT cells comprised ∼48.5× coverage to bring the combined total to ∼65× coverage.

### *M. zebra* diploid genome assembly

The 65× PacBio reads were assembled using FALCON-integrate/FALCON_unzip (version 0.4.0) [8]. The following parameters were used for the “fc_run.py” assembly step:

length_cutoff = 9000
length_cutoff_pr = 9000
pa_HPCdaligner_option = –v –dal128 –H10000 –M60 –t16 –e.70 –l2000 –s100 –k14 –h480 –w8
ovlp_HPCdaligner_option = –v –dal128 –H10000 –M60 –t32 –h1024 –e.96 –l1000 –s100 –k24
falcon_sense_option = –output_multi –min_idt 0.70 –min_cov 4 –max_n_read 350 –n_core 5
overlap_filtering_setting = –max_diff 100 –max_cov 150 –min_cov 0 –bestn 10 –n_core 18

This was followed by the unzip step (“fc_unzip.py”) and quiver polishing of the diploid assembly with the “fc_quiver.py” assembly step.

### Polishing of the *M. zebra* diploid genome assembly

The diploid assembly described above includes a PacBio polishing (quiver) step. However, there were also Illumina reads available for *M. zebra* from the first version of the assembly [5]. Trimming and filtering of the raw *M. zebra* Illumina reads have been described for the previous version of the assembly [13]. The trimmed and filtered fragment library corresponded to 30.1× coverage and the trimmed and filtered 2-3 kb plibrary corresponded to 32.6× coverage, for a total of 62.7× Illumina coverage. These Illumina reads were aligned to the diploid assembly with BWA mem [100] (version 0.7.12-r1044). Pilon [101] (version 1.22) was run, supplying the fragment library with the "–frags" option, and the 2-3 kbp library with the "–jumps" option and the following options: "–diploid –fix bases –mindepth 10 –minmq 1 –minqual 1 –nostrays".

This intermediate, Illumina-polished assembly was then polished again with the PacBio reads using SMRT-Analysis [102] (version 2.3.0.140936) using the 65× raw PacBio reads. First, each SMRT cell was separately aligned to the intermediate polished assembly using pbalign (version 0.2.0.138342) with the “–forQuiver" flag. Next, cmph5tools.py (version 0.8.0) was used to merge and sort (with the "–deep” flag) the pbalign .h5 output files for each SMRT cell. Finally, Quiver (GenomicConsensus version 0.9.2 and ConsensusCore version 0.8.8) was run on the merged and sorted pbalign output to produce an initial polished assembly.

### Detecting misassemblies in *M. zebra*

To detect misassemblies present in the intermediate polished assembly, several datasets were analyzed and compared. This included four genetic maps: a genetic map with 834 markers generated from RAD genotyping of 160 F_2_ individuals from a cross of *M. zebra* and *M. mbenjii* [46], a genetic map with 946 markers generated from RAD genotyping of 262 F_2_ individuals from a cross of *L. fuelleborni* and *T. “red cheek”* [47], a genetic map of 2,553 markers generated from RAD genotyping of 331 F_2_ individuals from a cross of *M. mbenjii* and *A. koningsi*, and a genetic map of 1,217 markers generated from RAD genotyping of 161 F_2_ individuals from a cross of *M. mbenjii* and *A. baenschi*.

The markers for each of the four maps were aligned to the intermediate polished assembly using BWA mem [100] (version 0.7.12-r1044) and a separate SAM file was generated. Chromonomer [103] (version 1.05) was run for each map using these respective SAM files and map information as input. Chromonomer detected contigs in the intermediate assembly that were mapped to multiple LGs.

To narrow the location of these identified misassemblies, the Illumina 40-kbp mate-pair library from the first *M. zebra* assembly [5] was aligned to the intermediate assembly. The raw PacBio reads were aligned using BLASR [104] (version 1.3.1.127046) with the following parameters: *“*–minMatch 8 -minPctIdentity–70 –bestn 1 –nCandidates 10 –maxScore –500 –nproc 40 –noSplitSubreads –sam*.”* Regions of abnormal coverage in the PacBio read alignments, as well as abnormal clone coverage in the 40-kbp mate-pair, were identified for most potential misassemblies identified by the genetic maps. These misassembly regions were manually inspected using these alignments in IGV [105]. Additionally, RefSeq [49] (release 76) *M. zebra* transcripts were aligned to the intermediate assembly using GMAP [106] (version 2015-07-23) and RepeatMasker [107] repeat annotations were considered when defining the exact location of a misassembly break.

One additional misassembly was identified during the comparison of linkage maps (next section) and was subsequently broken using the same process as above.

### *M. zebra* assembly anchoring

The same four genetics maps used above for misassembly detection were also used for anchoring the assembly contigs (after breaking) into the final set of LGs. Chromonomer [103] (version 1.05) was run on each of these four genetic maps to anchor the polished and misassembly-corrected contigs. BWA mem (version 0.7.12-r1044*)* was used to create the input SAM file by aligning each respective map marker sequences to these contigs. Gaps of 100 bp were placed between anchored contigs. To accomplish the anchoring with multiple maps, the markers for each of those respective maps and LGs were used with Chromonomer as described above.

### *M. zebra* repeat annotation

RepeatModeler [108] (version open-1.0.8) was first used to identify and classify *de novo* repeat families present in the final anchored assembly. These *de novo* repeats were combined with the RepBase-derived RepeatMasker libraries [109]. RepeatMasker [107] (version open-4.0.5) was run on the final anchored assembly using NCBI BLAST+ (version 2.3.0+) as the engine (“–e ncbi”) and specifying the combined repeat library (“–lib”). The more sensitive slow search mode (“–s”) was used. The repeat landscape was generated with the RepeatMasker “calcDivergenceFromAlign.pl” and “createRepeatLandscape.pl” utility scripts.

The “genomation” package [110] within R (version 3.4.1) was used to determine the overlap of the RepeatMasker annotated elements DNA/TcMar, DNA/hAT, and LINE/Rex with the NCBI RefSeq gene models for both *M. zebra* and *O. niloticus*.

### *M. zebra* BUSCO genome completeness analysis

BUSCO (version 3.0.2) was run on the M_zebra_UMD2 anchored assembly in the genome mode (–m geno) and compared against the vertebrate BUSCO set (“vertebrata_odb9”).

### Whole-genome alignment of *M. zebra* to *O. niloticus*

The final anchored M_zebra_UMD2 assembly was aligned to the O_niloticus_UMD_NMBU assembly using the “nucmer” program of the MUMmer package [111] (version 3.1). The default nucmer parameters were used and the raw nucmer alignments were filtered using the “delta-filter” program with the following options: “–o 50 –l 50 –1 –i 10 –u 10.” These filtered alignments were converted to a tab-delimited set of coordinates using the “show-coords” program with the following options: “–I 10 –L 5000 –l –T –H.” This set of coordinates was then visualized using Ribbon [112] and used to generate the images in Additional File D.

### Whole-genome alignment of *M. zebra* to medaka

The HSOK medaka genome assembly version 2.2.4 was downloaded from [113] and corresponds to NCBI accession GCA_002234695.1. Similar to the M_zebra_UMD2 comparison, O_niloticus_UMD_NMBU was aligned to the medaka HSOK genome with nucmer. The “delta-filter” settings were adjusted to “-o 50 -l 50 -1 -i 10 -u 10” to account for the increased divergence between the 2 more distantly related species. The “show-coords” settings were also adjusted to “-I 10 -L 5000 -l -T -H.” Alignments were again viewed with Ribbon to identify putative chromosome fusion and translocation events and used to generate the part of the images in Figs   4 and 5.

### Summary figure

KaryoplotR [114] was used to generate the chromosome images, recombination curves, and large rearrangements in Fig. 6. The kpPlotLoess function was used to generate the recombination curves as locally estimated scatterplot smoothing (LOESS) smoothed curves using the markers for each respective map. A span of 0.17 and an interval of 0.1 was used for each curve.

## Availability of supporting data and materials

The *O. niloticus* Whole Genome Shotgun project has been deposited at DDBJ/ENA/GenBank under the accession MKQE00000000 (O_niloticus_UMD1). The version described in this article is version MKQE02000000 (O_niloticus_UMD_NMBU). The *M. zebra* Whole Genome Shotgun project has been deposited at DDBJ/ENA/GenBank under the accession AGTA00000000. The version described in this article is version AGTA05000000. All data are also available from the *GigaScience* GigaDB repository [115].

## Additional files

**Additional File A**—Read length distribution of the 65X coverage *M. zebra* PacBio reads.

**Additional File B**—M_zebra_UMD2 FALCON p-contigs where markers from two or more different LGs maps aligned, indicating a potential inter-LG misassembly.

**Additional File C**—Screenshot of IGV view to inspect potential misassemblies. In this example, a misassembly on this contig was confirmed at position 420,665 (indicated by the white arrows). The top red box shows the portion of the contig that is being visualized. LG17 markers aligned at 186 and 308 kbp, while LG10a markers aligned at 760 and 1.6 Mbp as indicated by the red arrows. The top two tracks below that are the read coverage plots for the PacBio read alignments against the diploid and haploid sets of contigs. There is a sharp decrease in PacBio read coverage at the misassembly location. The track below shows 40 kbp mate-pair alignments and also shows no clone coverage at the location of the misassembly.

**Additional File D**—*M. zebra* assembly contigs anchored with each of the 4 maps and aligned to O_niloticus_UMD_NMBU (indicated as black on bottom with contigs in red for each panel). Centromeres indicated with black triangles. Contigs are represented as red lines above each respective assembly.

**Additional File E**—(a) F_ST_ comparison of male and female *O. aureus* LG3 ZW. (b) *O. niloticus* recombination curve of LG3 from Additional File F.

**Additional File F**—*O. niloticus* recombination curves for females (red) and males (blue). Centromere repeats are displayed as green triangles where applicable. X-axis represents the location along the anchored LG. Left Y-axis represents linkage disequilibrium (black points, *r*^2^ > 0.97) and right Y-axis shows the map location for each marker.

**Additional File G**—Comparison of recombination in the four Lake Malawi genetic maps. LGs from maps that needed to be reversed from their original published order are indicated in Additional File M. The detected misassembly on LG12 is included on page 13 of this file.

**Additional File H**—Histogram of read length distributions for the 44× coverage PacBio read sets from *M. zebra* and *O. niloticus*. These read sets were used for the closer comparison of recent repeats between the two species.

**Additional File I**—Comparison of the repeat landscape in the *M. zebra* and *O. niloticus* genome assemblies using same assembly parameters and 44× coverage PacBio data. Note that the Y-axis is different for the two repeat landscapes.

**Additional File J**—Spreadsheet of TE insertion locations by defining promoter regions as either 1 or 15 kbp.

**Additional File K**—Comparison of the repeat landscape in the three *M. zebra* assembly versions.

**Additional File L**—Table of the orientation of Lake Malawi recombination maps for each LG. The forward and reverse orientation information of each map was used to generate recombination plots in the same orientation for Additional File G.

## Abbreviations

a-contig: associate contig; AFC: African cichlid–specific repetitive element; bp: base pairs; BUSCO: Benchmarking Universal Single-Copy Orthologs; cM: centimorgan; indel: insertion or deletion; kbp: kilobase pairs; LD: linkage disequilibrium; LG: linkage group; LINE: long interspersed nuclear element; LOD: logarithm of the odds; LOESS: locally estimated scatterplot smoothing; Mbp: megabase pairs; N50: shortest contig/scaffold/read/sequence length at 50% of the genome/read set; NCBI: National Center for Biotechnology Information; NG50: shortest contig/scaffold/read/sequence length at 50% of the estimated genome/read set size; ONSATA: *Oreochromis niloticus* satellite A repeat; PacBio: Pacific Biosciences; p-contig: primary contig; RAD: restriction site–associated DNA; RefSeq: NCBI Reference Sequence Database; SMRT: single molecule, real-time; SNP: single-nucleotide polymorphism; TE: transposable element; TZSAT: *Tilapia zillii* satellite repeat.

## Animal care

Animal procedures were conducted in accordance with University of Maryland Institutional Animal Care and Use Committee Protocol No. R-10-74.

## Competing interests

The authors declare that they have no competing interests.

## Funding

This work was supported by the US Department of Agriculture under project No. MD.W-2014-05906 to T.D.K., the National Science Foundation under grant No. DEB-1143920 to T.D.K., the National Institutes of Health project R01-EY024639 to K.L.C., and the Beckman Young Investigator Award from the Arnold and Mabel Beckman Foundation to R.B.R.

## Authors' contributions

M.A.C., T.D.K., and K.L.C. conceived the study. T.D.K. carried out HMW DNA extraction. M.A.C. carried out computational analyses. R.J., E.C.M., S.P.N., R.B.R., and S.L. performed genetic map construction. M.A.C. and S.L. integrated the tilapia linkage map with the assembly. W.J.G. organized map data for anchoring. M.A.C. and T.D.K. wrote the manuscript. All authors read and approved the manuscript.

## Supplementary Material

GIGA-D-18-00286_Original-Submission.pdf

GIGA-D-18-00286_Revision 2.pdf

GIGA-D-18-00286_Revision-1.pdf

Response-to-Reviewer-Comments-Revision-1.pdf

Response-to-Reviewer-Comments_Original-Submission.pdf

Reviewer-1-Report-Original-Submission -- Alexander Nater 9/7/2018 Reviewed

Reviewer-1-Report-Revision-1 -- Alexander Nater 2/1/2019 Reviewed

Reviewer-2-Report-Original-Submission -- Milan Malinsky 9/10/2018 Reviewed

Supplement_Files.zip
