# Supplementary material for: Chromosome-scale assemblies reveal the structural evolution of African cichlid genomes
Source: Gigascience. 2019 Apr 3;8(4):giz030. doi: 10.1093/gigascience/giz030 (PMC6447674; doi:10.1093/gigascience/giz030)
Supplement: GIGA-D-18-00286_Revision-1.pdf [file giz030_giga-d-18-00286_revision-1.pdf]

GigaScience

Chromosome-scale assemblies reveal the structural evolution of African cichlid genomes

--Manuscript Draft--

**Manuscript Number:**	GIGA-D-18-00286R1
**Full Title:**	Chromosome-scale assemblies reveal the structural evolution of African cichlid genomes
**Article Type:**	Research
**Funding Information:**	National Institute of Food and Agriculture (MD.W-2014-05906) — Dr. Thomas D Kocher<br>National Science Foundation (DEB-1143920) — Dr. Thomas D Kocher<br>National Institutes of Health (R01-EY024639) — Dr. Karen L Carleton<br>Arnold and Mabel Beckman Foundation (US) (Beckman Young Investigator Award) — Dr. Reade B Roberts

Abstract:

Background

African cichlid fishes are well known for their rapid radiations and are a model system for studying evolutionary processes. Here we compare multiple, high-quality, chromosome-scale genome assemblies to understand the genetic mechanisms underlying cichlid diversification and study how genome structure evolves in rapidly radiating lineages.

Results

We re-anchored our recent assembly of the Nile tilapia (Oreochromis niloticus) genome using a new high-density genetic map. We developed a new de novo genome assembly of the Lake Malawi cichlid, Metriaclima zebra, using high-coverage PacBio sequencing, and anchored contigs to linkage groups (LGs) using four different genetic maps. These new anchored assemblies allow the first chromosome-scale comparisons of African cichlid genomes.
Large intra-chromosomal structural differences (~2-28Mbp) among species are common, while inter-chromosomal differences are rare (< 10Mbp total). Placement of the centromeres within chromosome-scale assemblies identifies large structural differences that explain many of the karyotype differences among species. Structural differences are also associated with unique patterns of recombination on sex chromosomes. Structural differences on LG9, LG11 and LG20 are associated with reductions in recombination, indicative of inversions between the rock- and sand-dwelling clades of Lake Malawi cichlids. M. zebra has a larger number of recent transposable element (TE) insertions compared to O. niloticus, suggesting that several TE families have a higher rate of insertion in the haplochromine cichlid lineage.

Conclusion

This study identifies novel structural variation among East African cichlid genomes and provides a new set of genomic resources to support research on the mechanisms driving cichlid adaptation and speciation.

**Corresponding Author:**	Matthew A Conte<br>University of Maryland<br>College Park, Maryland UNITED STATES
**Corresponding Author Secondary Information:**	
**Corresponding Author's Institution:**	University of Maryland
**Corresponding Author's Secondary Institution:**	

Powered by Editorial Manager® and ProduXion Manager® from Aries Systems Corporation

**First Author:**	Matthew A Conte, PhD
**First Author Secondary Information:**	
**Order of Authors:**	Matthew A Conte, PhD
	Rajesh Joshi
	Emily C Moore, PhD
	Sri Pratima Nandamuri, PhD
	William J Gammerdinger, PhD
	Reade B Roberts, PhD
	Karen L Carleton, PhD
	Sigbjørn Lien, PhD
	Thomas D Kocher, PhD
**Order of Authors Secondary Information:**	
**Response to Reviewers:**	We thank both reviewers for their careful reading of our manuscript. We have worked to significantly shorten and tighten the text. We detail our response to each of their specific comments in the paragraphs below.<br><br>Reviewer #1:<br>This paper by Conte and colleagues describes two new chromosome-scale genomic assemblies of African cichlids. The authors used multiple genetic maps to anchor contigs from high-coverage PacBio sequencing and correct misassemblies. Based on these two high-quality genomes and the genetic maps, the authors performed comprehensive comparative analyses of recombination landscapes, large-scale chromosomal rearrangements, and transposable element insertions. The paper presents extensive genomic resources, which will be valuable for future studies in the field.<br>However, the manuscript in its current form is highly descriptive and many parts of the paper are repetitive and very tedious to read. I'm convinced that the appeal of the paper for a broader readership could be improved considerably by shortening the main text and putting a focus on the biologically interesting aspects. The purely descriptive details could be presented more effectively in tables and figures or additional supporting materials. For instance, instead of the lengthy description of rearrangements, regions of unusual recombination, and putative sex determination loci, I would like to see a carefully designed summary figure, which provides the reader with a good overview of these events in the two genomes.<br>In its current form, many interesting aspects are buried in large amounts of text that provide information of little biological relevance. Most importantly, the discussion part should be written much more concisely, as it still largely descriptive and repeats most of the information that was already provided in the results section. Here, the authors should refrain from discussing every single aspect of their results and rather focus on the biological interpretation of the most interesting findings of the study.<br><br>Minor comments:<br>Page 4, line 15: Define "indel" here.<br>We have added this definition.<br><br>Page 4, line 52: Provide reference for PacBio sequencing.<br>We have added this reference.<br><br>Page 10, Table 1: Improve the labelling of the table. It is not immediately clear that the numbers represent base pairs.<br>We have improved the labeling of this table.<br><br>Page 11, line 42: "relatively complete" compared to what?<br>We have edited this sentence to clarify.<br><br><br>Page 11, line 35: The description here seems to imply that the final p-contigs are not

phased. The p-contigs are phased within the borders of their associated haplotigs. Please provide a more detailed explanation of p-contigs and haplotigs here.
We have modified this sentence to remove the wrong implication and have added a more detailed explanation of p-contigs and haplotigs.

Page 11, lines 42-55: This part is unclear and should be rewritten. What are "theoretical sizes of heterozygous regions"? Do you mean the theoretical expectation of the distribution of distances between heterozygous sites? The associated Additional File A is completely unclear and needs a much more detailed explanation and legend, e.g. what is the relationship between the two x-axes (length in base pairs and recombination rate) or the two y-axes (frequency and E(r2))? How do you derive information about the completeness of haplotigs from this graph?
The axes of File A were poorly labelled, and our conclusions a little overextended. We have revised the text, figure and legend to improve the presentation of these results. The main point is that the size distribution of the homozygous regions in the genome assembly closely matches the expected size distribution of homozygous regions in an individual drawn from a population with an Ne of 1,000 – 2,000 individuals. We no longer make any claims about the completeness of the genome assembly from this graph.

Page 12, line 55: Provide full genus name for A. koningsi.
We have added this.

Page 13, Table 3: Why does the total length differ for the four different maps, given that it includes both anchored and unanchored contigs?
We had added a sentence that clarifies this.

Page 15, lines 34-42: Given that the anchoring is based on a combination of four different maps, is it possible that certain contigs are represented multiple times in the final assembly?
We have added a sentence that addresses this question.

Page 16, lines 4-47: Given that all genetic maps are from inter-species crosses, what are the expectations for inter-chromosomal rearrangements that are only present in one of the two species? It seems unlikely, that the given approach would have power to detect rearrangements in such cases.
The reviewer is correct that we would be unlikely to detect inter-chromosomal rearrangements in interspecific crosses among Lake Malawi species. Recombination in such regions in hybrids would likely produce inviable gametes or offspring, effectively suppressing recombination. So, such rearrangements would produce only the weak signal of reduced recombination through these regions, which we would not likely detect.

Page 16 line 34: "… at most 1% of these Lake Malawi genomes is affected by inter-chromosomal rearrangements …"
We have deleted this sentence. While we believe it is likely true, based on the limited amount of inter-chromosomal rearrangement between tilapia and Metriaclima, we currently do not have the data to support this statement.

Page 23, lines 46-48: This sentence doesn't make sense without a distance qualifier, i.e. significant linkage disequilibrium over extended physical distances.
We have added this qualifier.

Page 26, line 59: "Only one contig longer than 1Mbp was not anchored …"
We have modified this sentence.

Page 27, line 7: "Contigs in the M_zebra_UMD2 assembly were primarily anchored with" or "The M_zebra_UMD2 anchoring was primarily performed with"
We have changed this sentence.

Page 27, lines 46-48: The suggested link between TEs and chromosomal rearrangements seems a bit far-fetched. It appears more likely to me that low recombination is facilitating the enrichment of both TEs and rearrangements due to reduced Ne and therefore reduced efficacy of selection against slightly deleterious

events in these regions.
We agree that there is an enrichment of TEs and rearrangements in regions of low recombination due to reduced efficacy of selection against slightly deleterious events in these regions and had already included a citation supporting that idea (Dolgin et al 2008). However, the link between TEs and chromosomal rearrangements is not far-fetched, and many studies have demonstrated this. We have added a sentence with several citations to support our claim.

Page 27, line 56: Not clear what is meant by "orthogonal mapping technologies" here. Alternative mapping technologies?
We have clarified this sentence.

Page 28, line 51: Genetic differentiation between what?
We have added to this sentence to be more explicit.

Page 30, line 33: Linkage group information is missing for the sex determination locus.
We have added this.

Page 30, lines 45-49: Rather the alleles of the sex determination system segregate in three crosses.
We have modified this sentence.

Page 32, lines 36-44: The connection between lack of evidence for a chromosome fusion event on LG3 and the accumulation of repetitive elements is not clear.
We have added some text to clarify this connection.

Page 34, line 29: What are "centromere-containing repeats" and does this refer to the ONSATA and TZSAT satellite sequences in the next sentence? Please rephrase this part.
We have rearranged and modified a sentence to make this clear.

Page 37, line 38: "will be able to purge"
This has been incorporated.

Page 40, line 9: Incomplete sentence
This sentence has been modified.

Page 40, line 29: Omit "that"
This sentence has been modified.

Page 40, line 44: Check reference. Reference to PLINK software doesn't make sense in this context.
We are unsure what the reviewer means, as one of the many things that PLINK can do is report LD statistics as described.

Reviewer #2:

General comments:
With their high speciation rates and remarkable phenotypic diversity, African cichlid fishes serve as a model for studying a broad range of evolutionary processes. In this manuscript, the authors use two high-quality genome assemblies and five genetic maps to investigate large scale structural variation, changes in broad-scale patterns of recombination, and to compare transposable element contents of the two assemblies. I think that the work is generally well executed and provides valuable new insights. The manuscript is also clearly written and the arguments are easy to follow. My only critical comment related to the current text is its repetitiveness, with the same statements often occurring in several places (see examples below). Especially the discussion is very long and, even though it offers some additional interpretation and explanations, it reads largely as a restatement of the analyses and the results.

Overall, I congratulate the authors on their work. I have some (relatively minor) comments and suggestions that the authors should consider before publication.

Specific comments/suggestions:
Data Description - this could be expanded, especially considering the GigaScience journal's focus. For example, for the new PacBio reads, I would be interested to see the read length distribution which is a crucial factor in genome assembly, especially influencing its contiguity and the types of repetitive elements that can be assembled. Perhaps the two "new genetic maps" should also have a brief description here? I don't know what the GigaScience journal policy is, but this work is not fully reproducible until the "new genetic maps" are published. I highlighted this in the comments to the Editor.

We have added a figure of the read length distribution as well as several statistics that provides more information when evaluating the genome assembly as the reviewer suggests. We have also added a brief description of the two new genetic maps. The new genetic maps themselves have already been uploaded to the GigaScience FTP to make the work reproducible.

Analyses
Page 10 - "The anchored assembly of LG3 is 54.7% repetitive, compared to repeat rate of 37% genome-wide" Perhaps a clarification of what is meant "repetitive" would be useful. Do the numbers quoted by the authors refer to the portions of the chromosomes masked by the RepeatMasker software?
We have modified this sentence to clarify this point.

Page 11 -"To measure the completeness of the haplotigs, the theoretical sizes of heterozygous regions under null expectations of recombination rates and effective population sizes were compared to the size distribution of the haplotigs. Additional File A shows the…" I don't understand this analysis and the associated "Additional File A". What exactly are the "haplotigs"? What determines their boundaries? Perhaps the authors should elaborate, and/or at the very least provide a reference to the theory that predicts how large the haplotigs should be.
See response to Reviewer #1 above. The theory on this point is explained on page 540 (Figure 9.8) of Hedrick's "Genetics of Populations" textbook (4th edition). The theory is attributed to Hill and Robertson 1968 (TAG 38:226-231) and Ohta and Kimura 1969 (Genet. Res. 13:47).

Page 24 - "…on the same compute cluster…"
I am not sure how the identity of the compute cluster could affect genome assembly. Read length and quality are two more likely factors that are not mentioned.
We have added an additional file that provides a comparison of the read length distributions of both datasets used in this particular analysis as well as some additional sentences to explain our inferences based on this comment.

Page 24 - "M. zebra genome assembly has a noticeably larger amount of recent TE insertions." Would be interesting to know where these insertions localised. For example, how many of these localised in gene promoters.
We have performed this requested analysis and provided the results as an additional file as well as providing additional text in the results and discussion.

Discussion
Page 27 - "optical mapping, may be needed to resolve the structure of these regions in finer detail." Again, perhaps longer reads that can span the repeats would help?
We have modified this sentence to include longer reads as a possibility.

Page 29 - "…suggesting the inversion may represent an evolved difference between the Metriaclima and Aulonocara lineages." I am not sure what exactly is meant by "an evolved difference". Isn't any difference between the lineages evolved? I suggest rephrasing this…
We have reworded this.

Page 30 - "…suggesting that the rearrangement occurred in the Lake Malawi ancestor and has maintained reduced recombination in this region across all lineages."
The Malawi species used for the maps represent only one genus of mbuna and Aulonocara - a small subset of Lake Malawi lineages. So I suggest rephrasing this, instead of "Lake Malawi ancestor" something like "ancestor of the Lake Malawi species used in our crosses".

Powered by Editorial Manager® and ProduXion Manager® from Aries Systems Corporation

	This sentence has been removed in our efforts to remove some of the repetitive regions from our manuscript as the reviewer suggests below.<br><br>Page 30 - "…has an XY sex determination locus on [76]"<br>I think there is a word missing at the end of the sentence.<br>We have added this.<br><br>Some examples of repetitiveness in the current text:<br>* on page 10 we learn that LG3 is "the largest and most repetitive chromosome in O. niloticus [15], and is a sex chromosome in the closely related species, O. aureus", then on page 19 we read that "In the related species O. aureus, sex determination is controlled by a locus on LG3 [13,46]. ", then on page 20 we read "LG3, the largest chromosome in O. niloticus", then on page 23 that "LG3 is the largest tilapia chromosome", then on page 29 that LG3 is "where a sex determination locus is located in a sister species, O. aureus.", then on page 31 that this is the most complete assembly of "LG3, the largest chromosome in the O. niloticus karyotype (Figure 1). This chromosome carries a ZW sex locus in several species of Oreochromis [13,46]" on page 35 we read that "On LG3, a WZ sex determination region was previously identified [46] and characterized [13] in the congener species O. aureus" , then "the largest assembled chromosome in O. niloticus is LG3" on the same page. This is all rather tiresome.<br>* A different example: on page 44 is a description of which genetic map was used for anchoring particular LGs in the M. zebra assembly. But this is also shown in Table 3, and also described in even more detail in pages 14 and 15… and then reiterated again (without details) on page 27.<br>We have removed this repetition except in one case where it is needed to make a separate point.
**Additional Information:**	
**Question**	**Response**
Are you submitting this manuscript to a special series or article collection?	No
**Experimental design and statistics**<br><br>Full details of the experimental design and statistical methods used should be given in the Methods section, as detailed in our Minimum Standards Reporting Checklist. Information essential to interpreting the data presented should be made available in the figure legends.<br><br>Have you included all the information requested in your manuscript?	Yes
**Resources**<br><br>A description of all resources used, including antibodies, cell lines, animals and software tools, with enough information to allow them to be uniquely identified, should be included in the Methods section. Authors are strongly	Yes

encouraged to cite Research Resource Identifiers (RRIDs) for antibodies, model organisms and tools, where possible.<br><br>Have you included the information requested as detailed in our Minimum Standards Reporting Checklist?	
**Availability of data and materials**<br><br>All datasets and code on which the conclusions of the paper rely must be either included in your submission or deposited in publicly available repositories (where available and ethically appropriate), referencing such data using a unique identifier in the references and in the "Availability of Data and Materials" section of your manuscript.<br><br>Have you have met the above requirement as detailed in our Minimum Standards Reporting Checklist?	Yes

Manuscript
Click here to access/download;Manuscript;cichlid_chromosome_assemblies_r
Click here to view linked References

Title page

Chromosome-scale assemblies reveal the structural evolution of African cichlid genomes

Authors

Matthew A. Conte[1], Rajesh Joshi[2], Emily C. Moore[3], Sri Pratima Nandamuri[1], William J. Gammerdinger[1], Reade B. Roberts[3], Karen L. Carleton[1], Sigbjørn Lien[2], Thomas D. Kocher[1*].

Affiliations

1 Department of Biology, University of Maryland, College Park, MD 20742, USA
2 Centre for Integrative Genetics (CIGENE), Department of Animal and Aquacultural Sciences, Faculty of Biosciences, Norwegian University of Life Sciences, Ås, Norway
3 Department of Biological Sciences and W. M. Keck Center for Behavioral Biology, North Carolina State University, Raleigh, USA

*** -** Corresponding author orcid.org/0000-0002-7547-0133

Abstract

Background

African cichlid fishes are well known for their rapid radiations and are a model system for studying evolutionary processes. Here we compare multiple, high-quality, chromosome-scale genome assemblies to understand the genetic mechanisms underlying cichlid diversification and study how genome structure evolves in rapidly radiating lineages.

Results

We re-anchored our recent assembly of the Nile tilapia (*Oreochromis niloticus*) genome using a new high-density genetic map. We also developed a new *de novo* genome assembly of the Lake Malawi cichlid, *Metriaclima zebra*, using high-coverage PacBio sequencing, and anchored contigs to linkage groups (LGs) using four different genetic maps. These new anchored assemblies allow the first chromosome-scale comparisons of African cichlid genomes.

Large intra-chromosomal structural differences (~2-28Mbp) among species are common, while inter-chromosomal differences are rare (< 10Mbp total). Placement of the centromeres within the chromosome-scale assemblies identifies large structural differences that explain many of the karyotype differences among species. Structural differences are also associated with unique patterns of recombination on sex chromosomes. Structural differences on LG9, LG11 and LG20 are associated with reduced recombination, indicative of inversions between the rock- and sand-dwelling clades of Lake Malawi cichlids. *M. zebra* has a larger number of

recent transposable element (TE) insertions compared to *O. niloticus*, suggesting that several TE families have a higher rate of insertion in the haplochromine cichlid lineage.

Conclusion

This study identifies novel structural variation among East African cichlid genomes and provides a new set of genomic resources to support research on the mechanisms driving cichlid adaptation and speciation.

Keywords

Genome assembly; African cichlids; comparative genomics; genome rearrangements; chromosome evolution; karyotype; inversion; recombination; transposable elements; genetic maps

Background

African cichlid fishes, due to their phenotypic diversity and rapid speciation over the last several million years, are a model system for studying the mechanisms of evolution [1]. Many recent studies of cichlid speciation have used short read data to perform genome scans of single nucleotide polymorphisms (SNPs) and small insertion or deletions (indels) in order to identify genomic regions under selection [2–4]. However, there are numerous other ways that genomes can evolve, including the accumulation of larger indels, as well as intra- and inter-chromosomal rearrangements. Identification of these types of mutation requires high quality, nearly complete genome sequences.

Draft genomes of five African cichlid species were previously generated using Illumina short-read sequencing and used in an initial analysis exploring some of the forces at play in African cichlid speciation [5]. The draft genome assembly of the Lake Malawi cichlid, *Metriaclima zebra*, was at the time one of the most continuous and accurate genomes assembled from short reads, as revealed in the Assemblathon 2 competition [6]. However, these five draft genome assemblies still contained many gaps, and only the assembly of the Nile tilapia, *Oreochromis niloticus*, was anchored to linkage groups (LGs), making it difficult to compare the structure of cichlid genomes at chromosomal scales.

To improve these cichlid genome resources, we have employed long-read Pacific Bioscience SMRT sequencing [7]. Long-read DNA sequencing technology has made it much easier to create accurate and contiguous genome assemblies [8–12]. In particular, long-read technologies have allowed the assembly of repetitive

sequences, and the identification of structural variants. We previously improved the genome assembly for the Lake Malawi cichlid, *M. zebra*, using 16.5X coverage of PacBio reads to fill in gaps and characterize repetitive sequences [13]. We also produced a new high-quality genome assembly of *O. niloticus*, using 44X coverage PacBio sequencing. We were able to anchor 86.9% of the assembly to linkage groups, which allowed us to characterize the structure of two sex determination regions in tilapias [14].

Cichlid karyotypes are highly similar among species. The diploid chromosome number (2n) varies from 32-60, but more than 60% of species have a diploid number of 48 [15]. Most of the chromosomes are acrocentric, but between 0 and 9 metacentric pairs are present in each species [16,17]. Karyotypic changes may have played an important role in the evolution and speciation of African cichlids. Classical cytogenetic techniques are able to characterize differences in chromosome number and large fusion or translocation events, which are easily seen under the microscope. However, they are less suited to studying smaller genome rearrangements, including inversions smaller than several megabases. Comparisons of chromosome scale assemblies in other vertebrate groups have begun to identify extensive structural differences at both the cytogenetic and the sequence assembly level [18,19], but the role of chromosome rearrangements in recent adaptive radiations has not been well studied.

Chromosome-scale assemblies can be achieved either by physical mapping techniques [20], or by anchoring the contigs of the sequence assembly with genetic linkage maps. Genetic maps have the advantage of reflecting another important

feature of genomes, namely variation in recombination rate, which has manifold impacts on the levels of genetic polymorphism [21] and on the efficiency of genome scans [22].

Here we describe chromosome-scale assemblies of two cichlid genomes. First, we re-anchor our previously published PacBio assembly of the *O. niloticus* genome [14] using a new high-density genetic map [23]. Second, we present a new assembly of *M. zebra* based on 65X coverage of long PacBio sequence reads. Finally, we anchor the *M. zebra* assembly with several recombination maps produced from hybrid crosses among closely related species from Lake Malawi. The anchored genome assemblies of these two species allow for this first chromosome-scale comparison of African cichlid genomes. We focus our analyses on three aspects of genome evolution that are revealed by these new chromosome-scale assemblies: variation in recombination rate across the genome, structural variation among cichlid lineages, and the landscape of transposable elements.

First, we describe the pattern of recombination along each chromosome. Spatial variation in recombination rate has implications for patterns of genetic variation [24,25], the evolution of sex chromosomes [26], and the analysis of genome-wide associations between phenotypes and genotypes [22]. Despite the importance of recombination in shaping genome architecture [27], patterns of recombination are only beginning to be studied in cichlids [28]. A great diversity of sex chromosomes have evolved in East African cichlids, likely the result of sexual genetic conflict [29]. Rapid changes in sex determination mechanism, which are frequently variable even within species, may play an important role in cichlid

speciation [1]. The evolution of new sex chromosomes often involves chromosomal inversions, which also change the pattern of recombination [30–34]. Studies of these changing patterns of recombination, and their effects on genetic variation, have been hampered by the incomplete nature of the previous draft genome assemblies.

Second, we characterize the patterns of chromosome rearrangement among species. It has been suggested that teleost karyotypes have remained largely stable since the fish-specific whole genome duplication more than 300 million years ago [35]. This is in contrast to recent reports of chromosomal fusions among closely related cichlid species [36–38], and a large number of putative inversions associated with the evolution of sex chromosomes in various species [14,32,33]. Chromosome-scale assemblies of cichlids allow us to quantify the levels of synteny among teleost lineages, and the rate of intra-chromosomal rearrangement among cichlid lineages in East Africa. To further explore these distinct patterns of recombination and structural changes in cichlids, we also compare the cichlid genomes to the detailed genomic history of the medaka (*Oryzias latipes*). Previous studies of medaka have shown that, subsequent to the teleost-specific whole-genome duplication 320-350 million years ago, one subset of medaka chromosomes remained stable while another subset underwent more extensive fusion and translocation events [35,39]. Related comparisons using additional teleost species have shown that the number of chromosomes is relatively stable (24-25 chromosome pairs in 58% of teleosts) except for instances where chromosome fusion events in particular species have lowered the chromosome number [40].

Finally, we quantify the abundance and distribution of various transposable element (TEs) families in each genome. Several studies have documented the expansion of particular transposon families in East African cichlids (AFC TEs) [41,42]. Transposable elements may play an important role in shaping genome architecture, particularly the divergence of sex chromosomes. Transposable elements may also be an important source of regulatory mutations [43]. Since transposable elements may have been involved in the evolution of many other phenotypes, it is important that these sequences be well characterized in genome assemblies. Unfortunately, transposable elements are not well represented in genome assemblies that are based on short Illumina sequence reads. Our previous work has shown that long-read sequencing greatly improves both the length and quantity of TE repeats in cichlid genome assemblies [13,14]. A comparative analysis of transposable elements will improve our understanding of the patterns of transposon insertion and deletion during the radiation of East African cichlids.

Data Description

To begin this study of chromosome-scale comparisons of African cichlid genomes, we used a new high-density map of *O. niloticus* [23] to improve the anchoring of our recent genome assembly [14]. We also generated a high-quality *M. zebra* genome assembly from a single male caught on Mazinzi Reef in Lake Malawi. Single-molecule PacBio sequencing was performed to 65X coverage and a *de novo* assembly of the reads was constructed. Additional File A provides the distribution of

read lengths for this new 65X coverage PacBio dataset of *M. zebra*. The mean subread length is 7,885bp and the subread length N50 is 11,031bp. Two new genetic maps are presented here based on interspecific crosses of several Lake Malawi species. These maps, along with two previously published genetic maps, were used to quality check the assembly, break misassembled contigs, and anchor the sequence contigs to chromosomes. These new anchored genome assemblies of *O. niloticus* and *M. zebra* were then aligned to one another to compare their structure. The *O. niloticus* anchored assembly and sequencing reads are available under NCBI BioProject PRJNA344471. The *M. zebra* anchored assembly and sequencing reads are available under NCBI BioProject PRJNA60369.

Analyses

Anchoring the *O. niloticus* assembly to a high-density linkage map

The recently assembled *O. niloticus* genome [14] was re-anchored using a new high-density map that includes 40,190 SNP markers, see Methods and [23]. This new map identified 22 additional misassemblies not identified by previous maps. Table 1 provides a comparison of the previous O_niloticus_UMD1 assembly with this newly anchored O_niloticus_UMD_NMBU assembly.

Table 1 – Anchoring comparison of O_niloticus_UMD1 and O_niloticus_UMD_NMBU.

**Linkage group**	**O_niloticus_UMD1 LG (bp)**	**O_niloticus_UMD_NMBU LG (bp)**	**Change (bp)**
LG1	38,372,991	40,673,430	2,300,439
LG2	35,256,741	36,523,203	1,266,462
LG3	68,550,753	87,567,345	19,016,592
LG4	38,038,224	35,549,522	-2,488,702
LG5	34,628,617	39,714,817	5,086,200
LG6	44,571,662	42,433,576	-2,138,086
LG7	62,059,223	64,772,279	2,713,056
LG8	30,802,437	30,527,416	-275,021
LG9	27,519,051	35,850,837	8,331,786
LG10	32,426,571	34,704,454	2,277,883
LG11	36,466,354	39,275,952	2,809,598
LG12	41,232,431	38,600,464	-2,631,967
LG13	32,337,344	34,734,273	2,396,929
LG14	39,264,731	40,509,636	1,244,905
LG15	36,154,882	39,688,505	3,533,623
LG16	43,860,769	36,041,493	-7,819,276
LG17	40,919,683	38,839,487	-2,080,196
LG18	37,007,722	38,636,442	1,628,720
LG19	31,245,232	30,963,196	-282,036
LG20	36,767,035	37,140,374	373,339
LG22	37,011,614	39,199,643	2,188,029
LG23	44,097,196	45,655,644	1,558,448
Total anchored (%)	868,591,263 (86.0%)	907,601,988 (90.2%)	39,010,725 (4.2%)

The previous O_niloticus_UMD1 assembly anchored a total of 868.6Mbp while the new O_niloticus_UMD_NMBU assembly anchored a total of 907.6Mbp (90.2%). Much of the newly anchored sequence is on LG3, which increased by 19Mbp, from 68.6Mbp to 87.6Mbp. In the O_niloticus_UMD_1 assembly, LG3 was broken into LG3a and LG3b. The new assembly merged these into a single LG3. LG3 is the largest and most repetitive chromosome in *O. niloticus* [16], and is a sex chromosome in the closely related species, *O. aureus* [44]. 54.7% of LG3 was annotated as repetitive, compared to 37% across the whole genome (see Methods).

The repetitive nature of *O. niloticus* LG3 is also highlighted by the fact that it required this new dense map to anchor many small contigs to this linkage group. Several chromosomes (e.g. LG16) have fewer total bp anchored in the new assembly. This is due to the fact that misassembled contigs that have been broken according to the new map are now assigned to a different LG.

Diploid sequence assembly of *Metriaclima zebra*

We assembled 65X coverage PacBio reads using FALCON/FALCON-unzip [8] to generate the new diploid *M. zebra* assembly, "M_zebra_UMD2". FALCON first assembles the PacBio reads into primary contigs (p-contigs) and associate contigs (a-contigs) that correspond to alternate alleles. During the FALCON-unzip step, reads are assigned to haplotypes by phasing of heterozygous SNPs and then a final set of phased p-contigs and haplotigs are produced. Table 2 provides the assembly summary statistics for each of these assembly parts. The length of the p-contigs (total size 957Mb), compared to the estimated cichlid genome size of ~1Gbp based on Feulgen densitometry [45], suggests the assembly is relatively complete. The haplotigs of this diploid assembly represent the regions of the genome that are heterozygous. So, for portions of the genome that are heterozygous, the diploid assembly should be represented by both a p-contig and a corresponding haplotig. If one were to align the smaller haplotigs to the larger p-contigs, one could determine which regions of this genome were heterozygous (where haplotigs align) or homozygous (where haplotigs do not align). To measure the completeness of the haplotigs, we compare the haplotig size distribution with theoretical sizes of heterozygous regions under null expectations of recombination rates and effective

population sizes [46–48]. Additional File B shows the size distribution of the assembled haplotigs and how it relates to the theoretical recombination rate for several different effective population sizes (N_e). The shape of this haplotig size distribution is closest to the curves representing effective population sizes of 1,000-2,500, which closely matches a recent estimate of the effective population size in *M. zebra* [49]. Variance in recombination rate across the genome may bias this estimate.

Table 2. FALCON assembly results for *M. zebra*. NG50 and LG50 are based on an estimated genome size of 1Gbp [45]. N50 and L50 sizes are provided for a-contigs and haplotigs since the size for the alternate haplotype is not known.

Assembly fraction	Assembly size (Mbp)	Number of contigs	NG50 / N50 (Mbp)	LG50 / L50	Mean contig size (kbp)	Max contig size (Mbp)
FALCON p-contigs	986.67	3931	1.38	200	251.00	10.04
FALCON a-contigs	261.12	5625	0.054	1615	46.42	0.381
FALCON-unzip p-contigs	957.01	2313	1.42	186	413.75	10.01
FALCON-unzip haplotigs	642.33	6367	0.214	891	100.89	1.17

Anchoring the *M. zebra* genome assembly

Four genetic recombination maps were used to detect misassemblies, anchor contigs to chromosomes, and compare species level structural differences. The four maps were all produced from interspecific F_2 crosses genotyped with RADseq strategies and involve six Lake Malawi cichlid species in total. The two previously generated maps were estimated using 160 F_2 from a cross of *Metriaclima zebra* and *Metriaclima mbenjii* [50] and 262 F_2 from a cross of *Labeotropheus fuelleborni* and

Tropheops 'red cheek' [51]. The two new maps consisted of crosses of *M. mbenjii* x *Aulonocara koningsi* (331 F_2) (*in preparation*) and *M. mbenjii* x *A. baenschi* (161 F_2) [52]. Table 3 provides the total bp anchored to each LG for each of the four maps. The final M_zebra_UMD2 assembly anchors 760.7Mbp.

Table 3. Anchoring of the *M. zebra* assembly with four different genetic linkage maps. The FALCON assembly was anchored to each map separately, and the total bases anchored are shown for each LG and map. The anchored map LGs that were used for the M_zebra_UMD2 anchoring are indicated in **bold**. The *L. fuelleborni x Tropheops 'red cheek'* map had four LGs that were combined into two (LG10a/LG10b and LG13a/LG13b). Selection of particular linkage groups for the final anchoring is based on accuracy and not necessarily overall length. The total lengths including unanchored contigs differ slightly in length since the number of gaps (100bp) inserted were different for each anchoring.

**Linkage group**	*M. zebra x M. mbenjii* (160 F2)	*L. fuelleborni x Tropheops 'red cheek'* (262 F2)	*M. mbenjii x A. koningsi* (331 F2)	*M. mbenjii x A. baenschi* (161 F2)	M_zebra_UMD2
LG1	31,191,433	32,150,205	**38,662,702**	36,192,366	38,662,702
LG2	25,783,542	28,952,651	**32,647,892**	33,362,328	32,647,892
LG3	18,498,838	14,707,016	**37,717,145**	24,847,713	37,309,556
LG4	28,418,370	24,424,243	**29,889,472**	23,743,562	30,507,480
LG5	29,725,229	34,008,850	**36,154,892**	30,984,548	36,154,892
LG6	15,868,181	32,717,361	**39,879,506**	32,438,073	39,760,669
LG7	29,333,014	57,016,972	**64,381,187**	50,973,986	64,889,811
LG8	19,307,854	16,999,744	**24,280,574**	18,082,738	23,959,896
LG9	**21,018,370**	22,620,859	18,771,712	24,011,483	21,018,370
LG10	25,942,318	26,176,893	**32,583,833**	25,149,136	32,346,187
LG11	**32,253,887**	30,903,800	34,404,464	31,577,152	32,434,411
LG12	23,231,402	31,401,442	**34,043,602**	31,595,605	34,077,077
LG13	25,893,161	24,034,634	**31,886,878**	28,831,406	32,061,881
LG14	32,750,971	32,025,991	**37,909,455**	30,978,148	37,855,742
LG15	28,015,059	28,462,857	**34,537,245**	28,405,563	34,537,245
LG16	24,665,172	26,935,058	**34,727,877**	29,158,962	34,727,877
LG17	28,473,329	31,631,813	**35,766,785**	31,607,415	35,766,785
LG18	19,927,984	23,757,304	**29,457,134**	30,047,761	29,494,144

LG19	24,076,222	19,992,035	**25,739,093**	22,726,673	25,955,740
LG20	28,281,247	30,800,769	24,975,175	**29,774,176**	29,774,176
LG22	27,460,019	31,372,369	**34,717,234**	30,512,954	34,717,234
LG23	27,069,552	27,967,022	**42,736,004**	37,848,175	42,076,657
Total anchored (%)	567,185,154 (59.3%)	629,059,888 (65.7%)	755,869,861 (79.0%)	662,849,923 (69.3%)	760,736,424 (79.5%)
Total including unanchored	957,158,042	957,163,242	957,185,442	957,167,042	957,200,631

Prior to the final anchoring, these four maps were also used to detect and confirm potential misassemblies in the FALCON contigs. Additional File C lists the FALCON p-contigs for which markers from two or more different LGs aligned, an indicator of potential inter-LG misassembly. Each of these potential misassemblies was further evaluated using alignments of a 40kb Illumina mate-pair library [5], RefSeq gene annotations [53], and repeat annotations (see Methods). In some cases, it was determined that the map marker sequences were repetitive, giving a false signal of misassembly. A total of 33 potential misassemblies were inspected and 16 likely misassemblies were identified and broken. An example of one of these misassemblies is provided in Additional File D. Whole genome alignment comparisons (see section below) detected one additional intra-chromosomal misassembly at 6,922,000 on contig 000000F on LG12. This brought the final total to 17 misassemblies.

The *M. mbenjii* x *A. koningsi* map typically anchored more of the *M. zebra* assembly contigs, and in a more accurate order (i.e. greater collinearity with *O. niloticus*), than did the other three maps. This is likely due to the fact that the *M. mbenjii* x *A. koningsi* map had both more F_2 individuals and more map markers than the other three Lake Malawi cichlid maps, giving it the highest resolution. Anchoring with the other three maps resulted in anchoring of more contigs on LG2, LG9, LG18,

LG20 (see Table 3). However, the map that produced the longest anchored LG did not always appear to be the most accurate. To determine this accuracy, each *M. zebra* LG (anchored with each of the four maps) was aligned to the anchored *O. niloticus* assembly and compared (Additional File E). The *M. zebra* x *M. mbenjii* map was chosen to anchor LG9 as it showed the most similar ordering relative to the *O. niloticus* assembly (Additional File E). The *M. zebra* x *M. mbenjii* map was also chosen to anchor LG11 as the other three maps showed large putative structural differences (Additional File E and also seen in the recombination maps, presented below). LG20 was best represented by the *M. mbenjii* x *A. baenschi* map based on alignment to *O. niloticus*, overall size and by ordering of markers in the recombination maps. Thus, the final M_zebra_UMD2 anchoring used three of the four maps to assign, order and orient contigs. The *L. fuelleborni x Tropheops 'red cheek'* map was not used in the final anchoring but did help confirm many misassemblies and provided information on structural differences. Several LGs have slightly different overall sizes than when the assembly was anchored with just a single map (e.g. LG3 changed from 37,717,154bp to 37,309,556bp, Table 2). This is due to the fact that several small contigs are assigned to different LGs by the four different maps. Although the final *M. zebra* anchoring is based on a combination of the four different maps, no contigs were represented multiple times in the final anchoring.

An anchoring analysis that sequentially chained together the anchored assemblies from all four Lake Malawi cichlid maps resulted in a slightly longer anchored assembly (833Mbp total compared to 760Mbp for M_zebra_UMD2).

However, the ordering of contigs in this combined anchored assembly was far less accurate (when aligned to *O. niloticus*) and so it was not used. There was only a single contig longer than 1Mbp (“000254F”) that was not anchored by at least one map.

Minimal inter-chromosomal differences among Lake Malawi cichlid genomes

The process of anchoring the M_zebra_UMD2 assembly using the four genetic maps also allowed us to look for large structural differences among the six species used to generate the maps. Specifically, we looked for p-contigs that were assigned to different LGs in any of the four maps. Table 4 provides the list of the 9 p-contigs that were assigned to a different LG by at least one map and which represent putative inter-chromosomal rearrangements.

Seven of these nine contigs are anchored to a different LG in one of the maps by only a single marker. It is difficult to determine if these represent true inter-chromosomal differences with such little evidence. Even when all nine contig anchoring differences are considered, it amounts to only 10.1Mbp of total inter-chromosomal differences between the species used to generate the maps. It is possible that there are some other significant inter-chromosomal differences that we did not detect in the unanchored portion of the genome. If they do exist, they are likely to be highly repetitive portions of these genomes that could not be assembled into the long contigs that can be accurately anchored.

Table 4. Putative inter-chromosomal differences as identified by map anchoring comparison. The number of markers aligned to each contig for each LG is indicated in (*N*). 'NA' indicates that a particular map had no markers aligned to that contig.

contig name	contig size	*Mz.* x *Mb.* map LG (160 F_2)	*Lf.* x *Tr.* map LG (262 F_2)	*Mb.* x *Ak.* map LG (331 F_2)	*Mb.* x *Ab.* map LG (161 F_2)	Notes
000084F_pilon|quiver	2,383,905	LG1 (1)	LG3 (3)	LG3 (6)	LG3 (3)	
000105F_pilon|quiver_1_1312536	1,312,536	NA	LG10a (1)	LG2 (1)	LG2 (3)	
000201F_pilon|quiver	1,489,552	LG3 (1)	*LG1 (3)*	LG3 (3)	LG3 (1)	
000223F_pilon|quiver	1,452,516	LG8 (4)	LG8 (8)	LG3 (2)	LG8 (4)	repetitive markers on LG3
000256F_pilon|quiver	1,241,607	LG20 (1)	LG20 (1)	NA	LG9 (1)	
000414F_pilon|quiver	805,874	LG5 (1)	LG5 (1)	NA	LG3 (1)	
000521F_pilon|quiver	566,343	LG15 (2)	NA	LG17 (1)	NA	repetitive marker on LG17
000541F_pilon|quiver	515,490	NA	LG2 (1)	LG3 (1)	NA	
000671F_pilon|quiver	374,096	LG23 (1)	NA	LG23 (1)	LG22 (1)	

Localization of centromeric repeats and karyotype differences

The location of centromeres is key to understanding structural rearrangements in the karyotype. Figure 1 shows the karyotype of *O. niloticus* and *Metriaclima lombardoi* (a species closely related to *M. zebra).* The *O. niloticus* SATA consensus repeat [54] is common to the centromeres of many East African cichlid [16], and closely matches the satellite repeats identified in a recent analysis of centromeres across many taxa [55].

Oreochromis and *Metriaclima* diverged 17-28 million years ago [56]. Their karyotypes each have 22 chromosome pairs, as do the majority of African cichlids, but *O. niloticus* has 1 to 3 meta-submetacentric and 19 to 21 subtelo-acrocentric chromosomes according to two previous karyotypes [16,57], whereas *M. zebra* has six meta-submetacentric and 16 subtelo-acrocentric chromosomes. The chromosomes in Figure 1 have been ordered by type and then by size but only LG3 and LG7 have been assigned to the karyotypes. BAC and additional marker sequences have been used for specific labeling of chromosomes in each species [37,58], but correspondence of chromosomes between species has not been established.

In order to understand the structural basis for these differences in karyotype, we constructed and visualized whole genome alignments of M_zebra_UMD2 and O_niloticus_UMD_NMBU (Additional File E). Figure 2 shows the LG23 alignment of *M. zebra* and *O. niloticus*. Placement of centromere repeats identify a large structural rearrangement on LG23 that shows that this chromosome is subtelo-acrocentric in *O. niloticus*, but meta-submetacentric in *M. zebra.*

Centromere repeats were not assembled on every chromosome for either *M. zebra* and *O. niloticus*. However, on chromosomes where centromere repeats were placed in both assemblies, and a large structural difference was observed, we were able to identify centromere repositioning events, including acrocentric/metacentric changes on LG3, LG16, LG17, and LG23. Although we were not able to identify the centromeres in both genome assemblies, similar rearrangement events suggest possible acrocentric/metacentric changes on LG2, LG6, LG20, and/or LG22 as well (Additional File E).

The whole genome alignment comparisons of *M. zebra* and *O. niloticus* also identified a number of large intra-chromosomal structural rearrangements that do not directly involve the centromere. On LG2 there are two large rearrangements of ~15Mbp and ~20Mbp (Additional File E). The largest single structural change appears on LG19 where there is a ~23Mbp rearrangement between *M. zebra* and *O. niloticus*. A similar ~20Mbp rearrangement is present on LG20. There is an ~11Mbp rearrangement at one end of LG22 that may be associated with another change in centromere location, although the centromere was not localized on LG22 in either assembly.

Perhaps the most diverged chromosome in terms of size, structure and repeat content is LG3. The karyotype of *O. niloticus* LG3 is much larger and more repetitive than the corresponding LG3 in Lake Malawi cichlids (Figure 1 and [16,57]). Additional File F shows an F_{ST} comparison of the *O. aureus* male versus female pools described in [14]. There is a very wide region of sex-patterned differentiation in *O. aureus* on LG3 from ~40Mbp to 85Mbp. The large karyotype of LG3 in *O. niloticus* reflects both this large region of differentiation associated with the sex-determination locus (>40Mbp) as well as the vast amounts of repetitive sequence that have accumulated in this region.

Variation in recombination rate among species

To compare the rates and patterns of recombination across the chromosomes, each set of map markers was aligned to the corresponding assembly and their recombination map positions plotted against physical distance. Male and female recombination in *O. niloticus* is plotted against the O_niloticus_UMD_NMBU assembly in Additional File G. Typically, the *O. niloticus* chromosomes are characterized by low recombination on the ends of chromosomes and higher recombination in the middle of chromosomes. Each of the *O. niloticus* chromosomes show a difference in recombination between males and females. The typical pattern is higher recombination in the females than the males. However, LG6 and large parts of LG4, LG9, LG20, and LG22 show higher recombination in males than females. LG3 and LG23 are both known sex determination chromosomes in tilapias [44,59], and each deviates from the normal recombination patterns. On LG3, there is very low recombination for ~70Mbp. On LG23 there is a ~28Mbp region of greatly reduced recombination.

Likewise, the markers in the four Lake Malawi genetic recombination maps were aligned to the final M_zebra_UMD2 assembly and their recombination map positions were plotted against physical distance. Figure 3 highlights the comparison of the four Lake Malawi genetic recombination maps relative to the M_zebra_UMD2 anchored assembly for four chromosomes. Additional File H contains plots for the other chromosomes. Similar to the *O. niloticus* chromosomes, many Lake Malawi chromosomes show low recombination on the ends of chromosomes and higher recombination in the middle of chromosomes, with several notable exceptions that are indicative of structural changes. In the Lake Malawi maps (Additional File H) there is a region of low recombination for the first ~15Mb of LG2 that corresponds with a large structural rearrangement relative to *O. niloticus* (Additional File E). On LG7 (Figure 3) the usual

pattern of low recombination at the ends of the chromosomes is observed in all four maps, but there is also a region of low recombination in the middle of the chromosome (at ~30Mbp in M_zebra_UMD2), near several smaller scale rearrangements relative to *O. niloticus* (Additional File E). An XY sex determination locus has been identified in this region of LG7 in many Lake Malawi species [30,60]. There is also evidence of large structural rearrangements on LG9 in all four Lake Malawi crosses, as evidenced by both the whole genome alignment and recombination map comparisons (Additional Files E and H). There appears to be a ~2Mbp inversion on LG10 (relative to *O. niloticus*) that is associated with lowered recombination around 20Mbp in M_zebra_UMD2 (Additional Files E and H). LG11 (Figure 3) follows the typical recombination pattern for the *M. zebra* x *M. mbenjii* map, but there appears to be a large 15Mbp inversion in the genus *Aulonocara*, inferred from a large region of complete recombination suppression found in both the *M. mbenjii* x *A. koningsi* and *M. mbenjii* x *A. baenschi* maps. This likely corresponds to another sex locus as has been suggested in a recent analysis of many sand-dwelling Lake Malawi cichlids [61]. Previous studies would also suggest that the *Metriaclima* species of these crosses likely contributed an XX allele [30] and the *Aulonocara* species likely contributed a heterozygous XY sex determining allele, but this has yet to be determined. The *L. fuelleborni* x *Tropheops 'red cheek'* map also shows a large, but different, rearrangement on LG11 when compared to *O. niloticus.* LG15 has a region of lower recombination in the middle that is also associated with structural rearrangements relative to *O. niloticus* (Additional Files E and H). There is a large structural rearrangement on LG20 present in each of the four anchored assemblies that is also associated with a large (~15Mbp) region of low recombination (Figure 3 and Additional Files E and H). Each of the four maps shows high recombination from 0-15Mbp and then much lower recombination to the end of LG23, although the *M. zebra* x *M. mbenjii* map

does not show as much reduction in recombination than the other three maps (Figure 3). The centromere of LG23 is placed at 30.1Mbp and is in the middle of the region of low recombination.

Major structural rearrangements of ancient cichlid chromosomes

We also aligned the O_niloticus_UMD_NMBU assembly to the recently published "HSOK" *O. latipes* medaka assembly [39]. *O. niloticus* has 22 chromosome pairs, while the medaka HSOK genome has 24 chromosome pairs. Table 5 is a comparison of cichlid chromosomes and medaka HSOK chromosomes.

Table 5. Correspondence between *O. niloticus* and *O. latipes* chromosomes. Alignment lengths are provided for chromosomes with large fusion/translocation events.

O_niloticus_UMD_NMBU chromosome	Primary medaka HSOK chromosome (alignment length)	Secondary medaka HSOK chromosome (alignment length)
LG1	3	
LG2	10	
LG3	18	
LG4	8	
LG5	5	
LG6	1	
LG7	6 (32Mbp)	12 (31Mbp)
LG8	19	
LG9	20	
LG10	14	
LG11	16	
LG12	9	
LG13	15	
LG14	13	
LG15	24 (31Mbp)	4 (5Mbp)
LG16	21	
LG17	23 (23Mbp)	4 (12Mbp)
LG18	17	
LG19	22	
LG20	7	
LG22	11	
LG23	2 (23Mbp)	4 (17Mbp)

We identified several large chromosome rearrangements that occurred in a cichlid ancestor. Tilapia LG7, the second largest chromosome (Table 1), is comprised of medaka chromosomes 6 and 12 in their entirety (Figure 4). This indicates a fusion of these ancestral chromosomes in cichlids relative to medaka, as had been previously suggested [38]. Tilapia LG23, the third largest chromosome (Table 1), is comprised of medaka chromosome 2 in its entirety and 17Mbp, or roughly half, of medaka chromosome 4 (Figure 5). The other half of medaka chromosome 4 was likely translocated onto LG15 and LG17. While the remaining 18 chromosomes have undergone extensive intra-chromosomal rearrangements, they have largely maintained a correspondence to individual medaka chromosomes over the course of the 120 million years of evolution since the last common ancestor of these species.

While LG3 is the largest tilapia chromosome (Table 1), it surprisingly does not show any evidence of a chromosomal fusion or translocation event. Tilapia LG3 aligns well to medaka chromosome 18 along the first ~30Mbp of LG3, and the remainder of LG3 aligns to medaka chromosome 18 with much less contiguity.

Figure 6 provides a summary of the major structural features in the evolution of cichlid chromosomes including recombination rates, putative centromeres, karyotype differences, fusions and large inversions greater than 6Mbp. The details of each of these chromosomal features can be found in Additional Files E, G and H.

Linkage disequilibrium

There is significant linkage disequilibrium (LD) over extended physical distances in the tilapia GST® population (see Methods), as shown in Figure 4 and Figure 5. As expected, the regions of low recombination near the ends of the chromosome show the highest levels of

linkage disequilibrium. Large blocks of LD are also evident around the centromere on LG15 (Additional File G), and in the low recombination regions associated with the ancestral chromosome fusions on LG7 (Figure 4) and LG23 (Figure 5).

Repeat landscape of the *Metriaclima zebra* assembly

The M_zebra_UMD2 assembly is 35% repetitive, similar to the O_niloticus_UMD1 assembly which is 37% repetitive [14]. Figure 7 shows the repeat landscape for the *M. zebra* and *O. niloticus* assemblies. While the *O. niloticus* genome assembly does have a slightly larger total quantity of annotated repeats, the *M. zebra* genome assembly has a noticeably larger amount of recent TE insertions (sequence divergence < 2%). To further test that this difference was not an artifact of the two different assembly processes, we assembled the *M. zebra* PacBio reads at the same 44X coverage as the *O. niloticus* assembly. A comparison of the read length distribution of the 44X subsampled *M. zebra* read dataset and the original 44X *O. niloticus* read dataset is provided in Additional File I. This subsampled 44X *M. zebra* assembly was performed with the same parameters, using the same version of Canu as was performed for the O_niloticus_UMD1 assembly. RepeatMasker was subsequently run on this assembly and the pattern of more recent insertion in *M. zebra* relative to *O. niloticus* was even more pronounced (Additional File J). The reason it is more pronounced is likely due to differences in the output of repetitive regions between the FALCON and Canu assemblers.

Three TE families account for most of the difference in the recent TE activity between the two species. Recent insertions (defined as 0-1% sequence divergence) of the class II DNA transposon superfamily Tc1-Mariner make up 0.5% of the total O_niloticus_UMD1 assembly but make up 1.3% of the M_zebra_UMD2 assembly. Recent insertions of another class II DNA transposon superfamily hAT make up 0.15% of the O_niloticus_UMD1 but make up 0.45% of

the M_zebra_UMD2 assembly. Recent insertions of the class I retrotransposon superfamily, LINE-Rex-Babar, make up 0.2% of the O_niloticus_UMD1 assembly, but make up 0.6% of the M_zebra_UMD2 assembly. Other TE superfamilies show smaller increases in *M. zebra* as well. This indicates that *M. zebra*, and perhaps Lake Malawi cichlids in general, have experienced more recent TE expansion than the *O. niloticus* lineage.

The insertion locations (with respect to gene structure) of these three most abundant TE superfamilies were categorized by defining promoters as either 1kb or 15kb upstream of transcriptional start sites and summarized (Additional File K). The LINE-Rex and DNA-TcMar superfamilies both have an increased amount of TE insertion in the 15kb promoter regions of *M. zebra* compared to *O. niloticus* (1,422 and 338 respectively), although there are fewer DNA-hAT elements present in the *M. zebra* promoters compared to *O. niloticus*. There is an increase of these recent TE superfamilies in intronic and intergenic regions, with the LINE-Rex elements having the largest increase in intronic regions (1,376 additional intronic insertions) and DNA-hAT having the largest increase in intergenic regions of *M. zebra* compared to *O. niloticus*. Similar overall patterns of insertion exist when considering a 1kb promoter, except for DNA-TcMar where slightly fewer 1kb promoter insertions were found in *M. zebra* than in *O. niloticu*s.

Overall, the amount of TEs assembled has increased from the original Illumina-only based *M. zebra* assembly [5], to the moderate PacBio coverage gap-filled M_zebra_UMD1 assembly [13], to the high PacBio coverage M_zebra_UMD2 assembly. Additional File L provides a comparison of repeat landscapes for each of these three *M. zebra* assemblies. The overall number of TEs, and particularly the most recently inserted TEs, are better represented as the assemblies improve. The African Cichlid-specific AFC-SINEs and AFC-LINEs [62], have been assembled in greater length as well. For example, the ~7.1kb “L1-1_AFC” LINE was

assembled into 2,874 copies (across 1.29Mbp) in the original M_zebra_v0 assembly, 1,350 copies (across 1.66Mbp) in the M_zebra_UMD1 assembly and 2,295 copies (across 4.77Mbp) in the new M_zebra_UMD2 assembly.

Genome completeness and annotation

Benchmarking Universal Single-Copy Orthologs (BUSCO) [63,64] was used to assess the completeness of the new *M. zebra* genome assembly. 2,586 complete vertebrate BUSCOs were searched and 2,465 (95.3%) complete BUSCOs were found, of which 71 (2.7%) were duplicated and 2,394 were single-copy. Only 82 (3.2%) were reported as fragmented, and just 39 (1.5%) BUSCOs were reported as missing.

The M_zebra_UMD2 assembly was annotated using the NCBI RefSeq annotation pipeline for eukaryotic genomes [53]. Table 6 shows the improvement in gene annotation for the new M_zebra_UMD2 assembly relative to the previous version of the *M. zebra* assembly [5,13].

Table 6. Annotation improvement of the M_zebra_UMD2 assembly gathered from RefSeq annotation reports [65,66].

Feature	M_zebra_UMD1	M_zebra_UMD2	Difference (%)
**Genes and pseudogenes**	27,328	32,471	5,143 (18.8%)
protein-coding	24,290	25,898	1,608 (6.6%)
non-coding	2,468	5,149	2,681 (108.6%)
pseudogenes	443	1,238	795 (179.5%)
**mRNAs**	44,123	46,160	2,037 (4.6%)
fully-supported	41,957	43,159	1,202 (2.9%)
partial	1,184	655	-529 (-44.7%)
with filled gaps	796	246	-550 (-69.1%)
known RefSeq (NM_)	9	12	3 (33.3%)
model RefSeq (XM_)	44,114	46,148	2,034 (4.6%)
**Non-coding RNAs**	3,192	6,209	3,017 (94.5%)
fully-supported	2,228	4,047	1,819 (81.6%)
model RefSeq (XR_)	2,518	4,851	2,333 (92.7%)
**CDSs**	44,263	46,358	2,095 (4.7%)
fully-supported	41,957	43,159	1,202 (2.9%)
partial	1,055	654	-401 (-38.0%)
with major corrections	358	478	120 (33.5%)
known RefSeq (NP_)	9	12	3 (33.3%)
model RefSeq (XP_)	44,127	46,161	2,034 (4.6%)

Discussion

Anchoring to produce chromosome-scale assemblies

The genetic recombination maps and whole genome alignment comparisons to the *O. niloticus* assembly were very useful for identifying large and mostly inter-chromosomal misassemblies in the new *M. zebra* assembly. A 40kb Illumina jumping library was also used in this process to determine if disagreements between the maps and the assembly were true misassemblies, errors in the maps, or structural differences between samples. It is likely that several misassemblies still remain in the final M_zebra_UMD2 anchoring. However, these potential misassemblies are probably only present on smaller contigs where there were not enough markers to detect misassembly events. Only one contig longer than 1Mbp was not anchored by two or more markers from one of the four Lake Malawi maps. Therefore, any possible remaining misassemblies are likely to involve smaller contigs. A high-density map of *M. zebra* would be a useful resource for future studies.

Patterns of continuity in genome assemblies

The longest contigs tend to be anchored in the middle of chromosomes and in regions where there is greater recombination. The ends of chromosomes, typically in regions of lower recombination, tend to have smaller contigs. Perhaps the clearest example of this is on LG13 (Additional File E and Additional File H). On LG7, smaller contigs appear in the middle of the chromosome where there is also a reduction in recombination uncharacteristic of most other chromosomes. Smaller contigs likely correspond to regions with a large fraction of repetitive sequence that lead to a more fragmented assembly. These regions have likely accumulated large TE arrays that are not spanned by even the longest of the reads in our datasets. It is known that

TEs accumulate in regions of suppressed recombination [67]. These chromosomal regions with smaller contigs also tend to have more structural rearrangements relative to *O. niloticus*, which suggests an important role for transposable elements in formation of the rearrangements. The role of various transposable element families in the formation of genomic rearrangements has been previously demonstrated in a variety of organisms [68–72]. This pattern could also be caused by ambiguities in the maps due to there being fewer recombination events and therefore less map resolution in these regions. There are also fewer markers used to anchor smaller contigs that may also contribute to this pattern. Longer read lengths and alternative mapping technologies, such as optical mapping and Hi-C, may complement the genetic recombination maps and be useful for defining the structure of these regions in finer detail.

Patterns of recombination in *O. niloticus*

Several patterns are evident in the recombination maps for *O. niloticus*. First, though the pattern of recombination is generally similar in males and females, the level of recombination in females is generally higher than in males. The total female map length is 1,641 cM, while the male map is only 1,321 cM. The sex differences in recombination rate of *O. niloticus* are smaller than observed in salmonids [73–76], stickleback [77], Japanese flounder [78], and zebrafish [79]. Second, the pattern of recombination on each chromosome is usually sigmoidal, with relatively little recombination over about 5Mb at the ends of each chromosome. The highest levels of recombination are found in the middle of each chromosome. This pattern is exactly opposite the pattern observed in stickleback and catfish, where recombination is highest at the ends of the chromosomes [80,81].

These patterns of recombination have implications for the pattern of linkage disequilibrium (LD) along each chromosome, which varies significantly across the genome. Blocks of LD are much longer in the regions of low recombination (Figure 4, Figure 5, Additional File G), such as near the ends of each chromosome. Regions of low recombination tend to accumulate repetitive transposable elements [67]. These regions are also likely to experience episodes of genetic hitchhiking, which will alter the pattern of genetic differentiation among populations across the genome, as shown in stickleback [77,80]. The extent of LD impacts the probability of fixation of adaptive variants and may affect the probability that a given chromosomal segment can evolve into a new sex chromosome [77]. Interestingly, extensive LD is present on LG3 in *O. niloticus*. One evolutionary interpretation of this finding is that high LD on LG3 predated, and facilitated evolution of, the LG3 sex chromosome present in *O. aureus* [44]. Alternatively, recombination suppression may have evolved as a result of sex-chromosome-associated evolution at LG3; in this scenario, the lineage leading to *O. niloticus* may have had, and subsequently lost, the dominant LG3 sex determination allele, but the traces of sex chromosome evolution remains in the genome.

Patterns of recombination in Lake Malawi cichlids

The four genetic maps of Lake Malawi cichlids show the same general pattern of recombination as *O. niloticus*. Again, the pattern of recombination on most Lake Malawi chromosomes is characterized by low recombination at the ends of the chromosomes and high recombination in the middle of the chromosomes. The several exceptions all indicate lineage-specific, intra-chromosomal rearrangements among the Lake Malawi species.

Perhaps the most striking difference between these four maps is a large (~19Mbp) putative inversion on LG11 in *Aulonocara*, as evidenced by the lack of recombination in the *M. mbenjii* x *A. koningsi* and *M. mbenjii* x *A. baenschi* maps (Figure 3). This putative inversion on LG11 likely corresponds to the same LG11 region recently reported to be associated with bower building behavior in sand-dwelling cichlids [61]. Large putative inversions and regions of low or no recombination are also evident on LG2, LG9, and LG20 (Figure 3 and Additional File H). As additional genetic maps of other African cichlids are developed, this framework can be used to see what additional variation in recombination and structure exists, and what can be learned from it.

Patterns of recombination on sex chromosomes

Sex chromosomes typically accumulate inversions that reduce recombination between the sex determining gene and linked sexually antagonistic alleles [82]. The strain of *O. niloticus* used to generate the genome assembly contigs [14] has an XY sex determination locus on LG1 [32,83]. The strain of *O. niloticus* used to generate the map [23] and anchor those contigs to chromosomes has an XY sex determination locus on LG23 [84]. We observed reduced recombination in males relative to females adjacent to the sex locus at 34.5Mbp on *O. niloticus* LG23 (Additional File G). As previously mentioned, LG3 carries a ZW sex locus in several species of *Oreochromis* [14,44], but not in the *O. niloticus* line assembled here. The ~70Mbp sex interval (Additional File F) is associated with the large reduction in recombination of both males and females (Additional File G). We also observed significant differences in recombination between the sexes on LG7, LG11, LG14 and LG15. An XY sex locus has been identified on LG14 in *O. mossambicus* [85], and XY sex loci have been identified on LG7 [30,60] and LG11

(unpublished) in Lake Malawi cichlids. Notably, alleles of the LG7 XY sex determination system segregate in three of the four Lake Malawi crosses, (the *M. mbenjii* x *A. baenschi* cross is unknown) (50,76 and unpublished results). However, LG7 shows relatively low recombination suppression compared to some other chromosomes. Recombination is reduced in the middle of LG7, centered at ~32Mbp, but this is not associated with the centromere (located at 61Mbp). While this region is near the LG7 XY sex determination interval, the overall shape of recombination on LG7 is likely the result of the chromosome fusion event that occurred in the cichlid ancestor (Figure 4 and discussed below). As discussed for *Oreochromis* above, it is unclear whether recombination suppression or sex determination evolved first at this locus. It should also be noted that there is a single marker in this region that appears out of order in the *M. zebra* x *M. mbenjii* map, perhaps indicating a structural difference (Additional File H and Figure 3). Further investigation will be needed to determine if other regions of the genome that display large differences in sex-specific recombination are associated with previously identified and/or novel sex determination loci.

Conservation of ancient synteny

Synteny is remarkably conserved among even distantly related teleosts [40,87]. Medaka show few inter-chromosomal rearrangements since shortly after the fish-specific whole genome duplication more than 300 MY ago [35]. Our whole genome alignment of tilapia to medaka supports the previously reported findings that the syntenic organization of teleost genomes is largely stable. The ancestral teleost chromosome number was 24 pairs [40]. In cichlids, where 22 chromosome pairs is most common [17], we find evidence for two large fusion events on LG7 and LG23 (Figure 4 and 5). Clearly, the variation in diploid number observed in other cichlid

species implies that there have been additional inter-chromosomal rearrangements, but we predict these will be simple fission/fusion events and not the result of scrambling of these ancient syntenic relationships.

The patterns of recombination across these particular chromosomes provide additional evidence of fusion and translocation events (Figure 4 and Figure 5). There are large deviations from the slope of the recombination curves located precisely where these fusion and translocation events have occurred. This also suggests that the pattern of recombination evolves slowly, as these oddly shaped recombination patterns have persisted for at least 15 million years since the divergence of the common ancestor of *O. niloticus* and the Lake Malawi species. Interestingly, although LG3 is the longest *O. niloticus* chromosome and has an odd pattern of recombination, LG3 does not seem to be the result of a chromosome fusion event. This lends support to the hypothesis that size of LG3 is due to accumulated repetitive sequences after LG3 became a sex chromosome, and that this sex chromosome signature and associated recombination suppression persists in *O. niloticus* even following loss of the LG3 sex determination system.

There are many examples of large-scale (>2Mbp) intra-chromosomal rearrangements between *O. niloticus* and Lake Malawi cichlids, as well as rearrangements evident among the Lake Malawi species. In some cases, the anchoring of the *M. zebra* assembly using each map showed the same large structural rearrangement relative to *O. niloticus* for each map (see LG2, LG19, LG20 in Additional File E). This suggests that these rearrangements happened prior to the Lake Malawi radiation, or are specific to *O. niloticus*. In other cases, there are large structural differences relative to *O. niloticus* that are different among the four maps (LG12, Additional File E), which suggests that these rearrangements occurred during the radiation in Lake Malawi. For

example, on LG11, the *M. zebra* x *M. mbenjii* map is mostly colinear with *O. niloticus*, but the other three maps show a large rearrangement and some differences in the order of this rearrangement. LG9 of *M. zebra* was particularly difficult to anchor with the *M. mbenjii* x *A. koningsi* map (Table 3). Additional work is needed to better define the structure of these chromosomes in each lineage.

Evolution of centromere position and sequence

Long-read sequencing has made it possible to assemble centromere repeats [88–90]. A recent study of centromere evolution in medaka provides an example of the role of centromere evolution in speciation [39]. The study showed that the centromere position of many medaka chromosomes has remained unchanged among *Oryzias* species in both acrocentric and non-acrocentric chromosomes. In other chromosomes, the position of centromeres did change and sometimes these chromosomes underwent major structural rearrangements involving other chromosomes. Alignment of the O_niloticus_UMD_NMBU assembly to these new medaka assemblies showed that cichlids have a different set of conserved and variable chromosomes compared to medaka. Additionally, the medaka study showed that centromere sequence repeats were more conserved in the chromosomes that remained acrocentric than in chromosomes that switched between acro- and non-acrocentric or that were non-acrocentric. Assembly and placement of cichlid centromere repeats in multiple species will provide insight into centromere evolution at the sequence level. Are there differences in centromere sequence/rate of evolution between acrocentric and non-acrocentric chromosomes? Are these differences great enough to create meiotic incompatibilities in hybrids? Are the positions of centromeres conserved across many species? This study provides a starting point to answer these questions.

Evolutionary patterns of African cichlid karyotypes

The karyotypes of *O. niloticus* and *M. zebra* in Figure 1 show that there have been at least 5 changes from subtelo-acrocentric to meta-submetacentric chromosomes. The clearest example of this is the 15Mbp rearrangement on LG23 (Figure 2). The ONSATA (*Oreochromis niloticus* satellite A repeat) and the TZSAT (*Tilapia zillii* satellite repeat) satellite sequences [91] have not been explicitly shown to be centromeric binding sequences, but rather are highly associated with the centromeres via *in situ* hybridization [16]. We were able to identify these ONSATA and TZSAT centromere-containing repeats on both the *M. zebra* and *O. niloticus* assemblies in just over half of the chromosomes (LGs 3, 4, 5, 7, 8, 9, 11, 13, 14, 16, 17, 19, 23). It is possible that these ONSATA and TZSAT repeat sequences may be present in other portions of the chromosome, or that some of them have been assembled incorrectly. Indeed, there are several chromosomes where the ONSATA and TZSAT repeats were identified in multiple distant locations along the chromosome in one or both assemblies (LG6, LG16, LG17, LG19).

Two of the chromosomes with identifiable karyotype changes have also been shown to harbor sex-determining loci in African cichlids. One is the previously mentioned XY sex determination region in *O. niloticus* on LG23 [84] and the ZW sex determination region on LG3 in *O. aureus* (Additional File F) (13), which corresponds to a low recombination region in male and female *O. niloticus*. The assembled and anchored chromosomes support the karyotypes (Figure 1) since the largest *O. niloticus* assembled chromosome is LG3 and the largest *M. zebra* chromosome is LG7 (Table 1 and Table 3). We suggest that LG3 expanded in the *O. niloticus* lineage by the accumulation of a large amount of TEs and segmental duplications, likely while linked to sex determination in a basal *Oreochromis* [14]. It is not clear if this apparent runaway

elongation of LG3 in *Oreochromis* is due to suppressed recombination of a sex-determination locus or some other mechanism. Additional genome assemblies of similar quality in related *Oreochromis* species should allow for further refinement of the evolutionary history of this large sex chromosome in the Oreochromini.

There is also a large (~28Mbp) region of greatly reduced recombination on LG23 in the *O. niloticus* map, as well as in each of the four Lake Malawi maps. LG23 is also the second largest anchored chromosome in the *M. zebra* assembly and third largest chromosome in the *O. niloticus* assembly. It is possible that this arm of LG23 is accumulating TEs similar to LG3, but at an earlier stage. There is an XY sex determination locus on LG23 in *O. niloticus* [59,84], and in at least one species of Lake Victoria cichlid [92], which may be contributing to changes in the size and rate of recombination on this chromosome. Three scenarios may explain these observations: 1) LG23 is an ancient sex chromosome, and though lost in the Malawi lineage, associated recombination suppression remains in Lake Malawi cichlids; 2) The LG23 sex determination locus is indeed segregating in Lake Malawi cichlids but has yet to be identified and described; 3) The recombination pattern on LG23 is not due to sex-chromosome-associated evolution but has been maintained by unknown factors in both lineages.

While many chromosomes have shown extensive rearrangement, it should also be noted that several chromosomes have undergone very little change since the divergence of *M. zebra* and *O. niloticus*. Other than relatively small structural changes at the ends of chromosomes, conserved synteny seems to have been maintained across the entire length of LGs 13, 14, 17 and 18 (Additional File E). It is possible that selective pressures have acted to maintain the synteny of these chromosomes. Since 20% of the *M. zebra* and 10% of the *O. niloticus* genome assemblies remain unanchored, future studies may provide additional structural insights. For

example, LG9 in *M. zebra* remains under-anchored. Future *in situ* and physical mapping studies should confirm these results in *O. niloticus* and *M. zebra*. Our work will greatly inform fine-scale cytogenetic studies aimed at characterizing intra-chromosomal differences among cichlid species.

Recent transposable element expansion in *M. zebra*

TEs have been shown to modulate gene regulatory networks, especially when they insert in regulatory promoters and introns [68,69]. In cichlids, recent evidence has shown that AFC-SINE indels in *cis*-regulatory regions of genes are associated with innovative cichlid phenotypes such as egg-spots [93]. A deletion that may be TE-mediated is responsible for controlling the expression of the *SWS2A* opsin [94]. It is likely that other AFC-specific and TE-mediated mutations have contributed to the diverse phenotypes of African cichlids. Therefore, it is important that these TE insertion events are well represented in genome assemblies.

This study has found that *M. zebra* has a higher number of recent TE insertions (sequence divergence < 2%) than *O. niloticus* (Figure 7 and Additional File J) and that many recent TE insertions occur in both promoter and intron regions (Additional File K). It remains to be seen if these recent TEs have been co-opted to alter gene regulatory networks and have played a large role in generating phenotypic diversity of African cichlids.

Since the *O. niloticus* assembly is 43.4Mbp longer than the *M. zebra* assembly, it is possible that the rate of recent TE insertions is even greater than we have quantified here. We present this finding with several caveats. It is possible that the two species have divergent patterns of insertion across the genome. We previously suggested *O. niloticus* contains larger clusters of repeat arrays that are experiencing recent insertions [14]. These very long arrays do not seem to be present at the same frequency in the *M. zebra* genome. It is possible that many

recent TE insertions in *O. niloticus* were not assembled completely and remain hidden in these large arrays. Differences in effective population size (N_e) between the two species may also account for differences in rate of TE accumulation, as larger populations will be able to purge deleterious insertions more efficiently. Other unknown technical factors may also have contributed to the difference that we have described. Future comparisons of additional samples and species assembled using the same sequencing coverage and assembly software/parameters will help to more accurately quantify the recent TE expansion in African Great Lake cichlids.

Diploid assembly

We present the new *M. zebra* assembly in both haploid and diploid representations. The majority of current genomics tools assume a haploid reference assembly and all subsequent analyses are based on this haploid representation. The use of multiple diploid assemblies will be required to capture population-level patterns of heterozygosity and complex structural variation. The genome assemblies reported here should therefore be considered the beginning of a larger effort to properly represent cichlid genomes. A study of *Arabidopsis thaliana* and *Vitis vinifera* (Cabernet Sauvignon) showed that the phased diploid assemblies produced by FALCON-unzip improved identification of haplotype structure and heterozygous structural variation [8]. Sequencing and assembly of F_1 in cattle has also been shown to recover these complex regions better and may be the way forward for assembly of diploid genomes [95]. Graph genome representations [96,97] have been shown to improve variant calling in complex regions such as the human leukocyte antigen (HLA) [98], major histocompatibility complex (MHC) [99] and centromeres [100]. Additional long-read diploid assemblies will be able to better represent genetic variation, particularly in regions of complex variation which current long read assemblies are beginning to span [89].

Potential implications

This study highlights the evolutionary insights that can be gained using a comparison of high-quality chromosome-scale genome assemblies, genetic recombination maps and cytogenetics across multiple related and, in this case, rapidly evolving species. It further illustrates the need for high-quality, chromosome-scale genome assemblies for answering many basic biological questions. This study illustrates the structural changes that can occur in the genomes of a rapidly evolving clade. It will be interesting to make comparisons to other radiations in the tree of life, both large and small. This study provides a wide-angle view of African cichlid genome history (summarized in Figure 6) and demonstrates how these high-quality resources can be used for many different types of evolutionary genomic analyses. As additional high-quality cichlid genomes are generated, this study will provide the foundation for comparisons of structural variation, recombination, cytogenetics, and repetitive sequences across the cichlid phylogeny. Many new questions have been generated here. How do the structural changes of African cichlid genomes compare to other groups? Is the pattern of few inter-chromosomal, but many intra-chromosomal differences seen here found in additional Lake Malawi genera as well as other radiations in Lake Tanganyika and Lake Victoria? Are these patterns of recombination observed across the majority of cichlids? Are any deviations from these typical recombination patterns related to specific phenotypic traits or sex chromosome history? How have these chromosomes evolved structurally? We look forward to the new dawn in cichlid genomics.

Methods

O. niloticus SNP array map, misassembly detection and new anchoring

Offspring (n=689) and parents from 41 full-sib families belonging to the 20th, 24th and 25th generations of the GST® strain were analyzed using a custom 57K SNP Axiom® Nile Tilapia Genotyping Array [23]. SNPs classified as "PolyHighRes" or "No-MinorHom" by Axiom Analysis Suite (Affymetrix, Santa Clara, USA), and having a minor-allele frequency ≥ 0.05, and call rate ≥ 0.85 were used in genetic map construction (n= 40,548). Lep-MAP2 [101] was used to order these SNPs into linkage groups in a stepwise process beginning with SNPs being assigned to linkage groups using the 'SeparateChromosomes' command. LOD thresholds were adjusted until 22 linkage groups were generated, which correspond with the *O. niloticus* karyotype. Unassigned SNPs were subsequently added to linkage groups using the 'JoinSingles' command and a more relaxed LOD threshold, and ordered within each linkage group using the 'OrderMarkers' command.

Sequence flanking each SNP (2 x 35nt) was used to precisely position 40,190 SNPs to the O_niloticus_UMD1 assembly (NCBI accession MKQE00000000) and thereby integrate the linkage and physical maps. This revealed 22 additional contig misassemblies (i.e. contigs containing SNPs from different LGs) that were not detected in the original anchoring for O_niloticus_UMD1. These contigs were subsequently broken. Linkage information was subsequently used to order and orientate contigs and build sequences for 22 Nile tilapia LGs in the new O_niloticus_UMD_NMBU assembly following the previous cichlid nomenclature [5,14,58,102].

LD results ($r^2 > 0.97$) presented in Figure 4, Figure. 5 and Additional file G, were produced in PLINK2 version 1.90b3w [103] using the pedigree described above and SNP-positions given in [103].

PacBio Sequencing of *M. zebra*

The previous version of the *M. zebra* assembly, M_zebra_UMD1 [13], included 16.5X PacBio sequencing (25 SMRT cells using the P5-C3 chemistry) on an PacBio RS II machine [13]. An additional library was prepared using the same Qiagen MagAttract HMW DNA extraction and Blue Pippin pulse-field gel electrophoresis size selection. An additional 60 SMRT cells (using the P6-C4 chemistry) were sequenced on the same PacBio RS II at the University of Maryland Genomics Resource Center as the previous 16.5X P5-C3 data. These P6-C4 SMRT cells comprised ~48.5X coverage to bring combined total to ~65X coverage.

M. zebra diploid genome assembly

The 65X PacBio reads were assembled using FALCON-integrate/FALCON_unzip (*version 0.4.0*) [8]. The following parameters were used for the '*fc_run.py*' assembly step:

length_cutoff = 9000

length_cutoff_pr = 9000

pa_HPCdaligner_option = -v -dal128 -H10000 -M60 -t16 -e.70 -l2000 -s100 -k14 -h480 -w8

ovlp_HPCdaligner_option = -v -dal128 -H10000 -M60 -t32 -h1024 -e.96 -l1000 -s100 -k24

falcon_sense_option = --output_multi --min_idt 0.70 --min_cov 4 --max_n_read 350 --n_core 5

overlap_filtering_setting = --max_diff 100 --max_cov 150 --min_cov 0 --bestn 10 --n_core 18

This was followed by the unzip step (‘*fc_unzip.py*’) and quiver polishing of the diploid assembly with the ‘*fc_quiver.py*’ assembly step.

Polishing of the *M. zebra* diploid genome assembly

The diploid assembly described above includes a PacBio polishing (quiver) step. However, there were also Illumina reads available for *M. zebra* from the first version of the assembly [5]. Trimming and filtering of the raw *M. zebra* Illumina reads are described for the previous version of the assembly [13]. The trimmed and filtered fragment library corresponded to 30.1X coverage and the trimmed and filtered 2-3kb library corresponded to 32.6X coverage for a total of 62.7X Illumina coverage. These Illumina reads were aligned to the diploid assembly with BWA mem [104] (*version 0.7.12-r1044*). Pilon [105] (*version 1.22*) was run supplying the fragment library with the ‘*--frags*' option, the 2-3kb library with the ‘*--jumps*' option and the following options: ‘*--diploid --fix bases --mindepth 10 --minmq 1 --minqual 1 --nostrays*'.

This intermediate, Illumina-polished assembly was then polished again with the PacBio reads using SMRT-Analysis [106] (*version 2.3.0.140936*) using the 65X raw PacBio reads. First, each SMRT cell was separately aligned to the intermediate polished assembly using pbalign (*version 0.2.0.138342*) with the ‘*--forQuiver*' flag. Next, cmph5tools.py (*version 0.8.0*) was used to merge and sort (with the ‘*--deep*’ flag) the pbalign .h5 output files for each SMRT cell.

Finally, Quiver (*GenomicConsensus version 0.9.2* and *ConsensusCore version 0.8.8*) was run on the merged and sorted pbalign output to produce an initial polished assembly.

Detecting misassemblies in *M. zebra*

To detect misassemblies present in the intermediate polished assembly, several datasets were analyzed and compared. This included four genetic maps: A genetic map with 834 markers generated from RAD genotyping of 160 F_2 individuals from a cross of *M. zebra* and *M. mbenjii* [50]; a genetic map with 946 markers generated from RAD genotyping of 262 F_2 individuals from a cross of *Labeotropheus fuelleborni* and *Tropheops 'red cheek'* [51]; a genetic map of 2,553 markers generated from RAD genotyping of 331 F_2 individuals from a cross of *M. mbenjii* and *Aulonocara koningsi* (cross and map construction details in separate Methods section); a genetic map of 1,217 markers generated from RAD genotyping of 161 F_2 individuals from a cross of *M. mbenjii* and *A. baenschi* (cross and map construction details in separate Methods section).

The markers for each of the four maps were aligned to the intermediate polished assembly using BWA mem [104] (*version 0.7.12-r1044*) and a separate SAM file was generated. Chromonomer [107] (*version 1.05*) was run for each map using these respective SAM files and map information as input. Chromonomer detected contigs in the intermediate assembly that were mapped to multiple linkage groups.

To narrow the location of these identified misassemblies, the Illumina 40kb mate-pair library from the first *M. zebra* assembly [5] was aligned to the intermediate assembly. The raw PacBio reads were aligned using BLASR [108] (version 1.3.1.127046) with the following parameters: *'-minMatch 8 -minPctI- dentity 70 -bestn 1 -nCandidates 10 -maxScore -500 -nproc*

40 -noSplitSubreads –sam'. Regions of abnormal coverage in the PacBio read alignments as well as abnormal clone coverage in the 40kb mate-pair were identified for most potential misassemblies identified by the genetic maps. These misassembly regions were manually inspected using these alignments in IGV [109]. Additionally, RefSeq [53] (*release 76*) *M. zebra* transcripts were aligned to the intermediate assembly using GMAP [110] (*version 2015-07-23*) and RepeatMasker [111] repeat annotations were considered when defining the exact location of a misassembly break.

One additional misassembly was identified during the comparison of linkage maps (next section) and was subsequently broken using the same process as above.

***M. zebra* assembly anchoring**

The same four genetics maps used above for misassembly detection were also used for anchoring the assembly contigs (after breaking) into the final set of linkage groups. Chromonomer [107] (*version 1.05*) was run on each of these four genetic maps to anchor the polished and misassembly corrected contigs. BWA mem (*version 0.7.12-r1044)* was used to create the input SAM file by aligning each respective map marker sequences to these contigs. Gaps of 100bp were placed between anchored contigs. To accomplish the anchoring with multiple maps, the markers for each of those respective maps and LGs were used with Chromonomer as described above.

***M. zebra* repeat annotation**

RepeatModeler [112] (*version open-1.0.8*) was first used to identify and classify *de novo* repeat families present in the final anchored assembly. These *de novo* repeats were combined

with the RepBase-derived RepeatMasker libraries [113]. RepeatMasker [111] (*version open-4.0.5*) was run on the final anchored assembly using NCBI BLAST+ (*version 2.3.0+*) as the engine ('*-e ncbi*') and specifying the combined repeat library ('*-lib*'). The more sensitive slow search mode ('*-s*') was used. The repeat landscape was generated with the RepeatMasker '*calcDivergenceFromAlign.pl*' and '*createRepeatLandscape.pl*' utility scripts.

The use of the 'genomation' package [114] within R (*version 3.4.1*) was used to determine the overlap of the RepeatMasker annotated elements DNA/TcMar, DNA/hAT, and LINE/Rex with the NCBI RefSeq gene models for both *M. zebra* and *O. niloticus*.

M. zebra BUSCO genome-completeness analysis

BUSCO (*version 3.0.2*) was run on the M_zebra_UMD2 anchored assembly in the genome mode (*-m geno*) and compared against the vertebrate BUSCO set ('vertebrata_odb9').

Whole genome alignment of *M. zebra* to *O. niloticus*

The final anchored M_zebra_UMD2 assembly was aligned to the O_niloticus_UMD_NMBU assembly using the '*nucmer*' program of the MUMmer package [115] (*version 3.1*). The default *nucmer* parameters were used and the raw *nucmer* alignments were filtered using the '*delta-filter*' program with the following options: '*-o 50 -l 50 -1 -i 10 -u 10*'. These filtered alignments were converted to a tab-delimited set of coordinates using the '*show-coords*' program with the following options: '*-I 10 -L 5000 -l -T -H*'. This set of coordinates was then visualized using Ribbon [116] and used to generate the images in Additional File E.

Whole genome alignment of *M. zebra* to medaka

The HSOK medaka genome assembly version 2.2.4 was downloaded from http://utgenome.org/medaka_v2/#!Assembly.md and corresponds to NCBI accession (GCA_002234695.1). Similar to the M_zebra_UMD2 comparison, O_niloticus_UMD_NMBU was aligned to the medaka HSOK genome with *nucmer*. The '*delta-filter'* settings were adjusted to *'-1 -l 50 -i 50 -u 50*' to account for the increased divergence between the two more distantly related species. The '*show-coords*' settings were also adjusted to '*-I 50 -L 50 -l -T -H*'. Alignments were again viewed with Ribbon to identify putative chromosome fusion and translocation events and used to generate the part of the images in Figure 4 and Figure 5.

Summary figure

KaryoplotR [117] was used to generate the chromosome images, recombination curves, and large rearrangements in Figure 6. The kpPlotLoess function was used to generate the recombination curves as LOESS smoothed lines using the markers for each respective map. A span of 0.17 and an interval of 0.1 was used for each curve.

Declarations

List of abbreviations

AFC – African cichlid specific repetitive element.

BUSCO – Benchmarking Universal Single-Copy Orthologs.

cM – Centimorgan.

LD – Linkage disequilibrium.

LG – Linkage group.

LINE – Long interspersed nuclear element.

LGs – Linkage groups.

N50 – Shortest contig/scaffold/read/sequence length at 50% of the genome/read set.

NG50 – Shortest contig/scaffold/read/sequence length at 50% of the estimated genome/read set size.

ONSATA – *Oreochromis niloticus* satellite A repeat.

ONSATB – *Oreochromis niloticus* satellite B repeat

PacBio – Pacific Biosciences.

RAD – Restriction site associated DNA.

RefSeq – NCBI Reference Sequence Database.

SMRT – Single Molecule, Real-Time.

TE – Transposable element.

TZSAT – *Tilapia zillii* satellite repeat.

Animal care

Animal procedures were conducted in accordance with University of Maryland IACUC Protocol #R-10-74.

Consent for publication

Not applicable.

Competing interests

The authors declare that they have no competing interests.

Funding

This work was supported by the United States Department of Agriculture under project number MD.W-2014-05906 to TDK, the National Science Foundation under grant number DEB-1143920 to TDK, the National Institutes of Health project R01-EY024639 to KLC and the Beckman Young Investigator Award from the Arnold and Mabel Beckman Foundation to RBR.

Author's contributions

MAC, TDK, and KLC conceived the study. TDK carried out HMW DNA extraction. MAC carried out computational analyses. RJ, ECM, SPN, RBR and SL performed genetic map construction. MAC and SL integrated the tilapia linkage map with the assembly. WJG organized map data for anchoring. MAC and TDK wrote the manuscript. All authors read and approved the manuscript.

Acknowledgements

We acknowledge all members of the UMD cichlid labs for their countless discussions on this project. We thank Luke Tallon and Naomi Sengamalay at the Genomics Resource Center, Institute for Genome Sciences for providing a high quality PacBio library and sequence reads. We acknowledge the University of Maryland supercomputing resources (www.it.umd.edu/hpcc/) made available in conducting the research reported in this paper. We thank Silje Karoliussen and Matthew Kent for genotyping and quality filtering of the Nile tilapia 58K SNP-array data. We thank the NCBI Eukaryotic Genome Annotation team, particularly Françoise Thibaud-Nissen, for their helpful feedback and guidance during the genome annotation process. We thank many individuals in the cichlid community for their patience while we developed these resources.

Author's information

Not applicable.

Availability of supporting data and materials

The *O. niloticus* Whole Genome Shotgun project has been deposited at DDBJ/ENA/GenBank under the accession MKQE00000000 (O_niloticus_UMD1). The version described in this paper is version MKQE02000000 (O_niloticus_UMD_NMBU). The *M. zebra* Whole Genome Shotgun project has been deposited at DDBJ/ENA/GenBank under the accession AGTA00000000. The version described in this paper is version AGTA05000000.

Endnotes

Not applicable.

References

1. Kocher TD. Adaptive evolution and explosive speciation: the cichlid fish model. Nat Rev Genet. 2004;5:288–98.
2. Malinsky M, Challis RJ, Tyers AM, Schiffels S, Terai Y, Ngatunga BP, et al. Genomic islands of speciation separate cichlid ecomorphs in an East African crater lake. Science. 2015;350:1493–8.
3. Malinsky M, Svardal H, Tyers AM, Miska EA, Genner MJ, Turner GF, et al. Whole-genome sequences of Malawi cichlids reveal multiple radiations interconnected by gene flow. Nat Ecol Evol. Springer US; 2018;2:1940–55.
4. Meier JI, Sousa VC, Marques DA, Selz OM, Wagner CE, Excoffier L, et al. Demographic modelling with whole-genome data reveals parallel origin of similar *Pundamilia* cichlid species after hybridization. Mol Ecol. 2017;26:123–41.
5. Brawand D, Wagner CE, Li YI, Malinsky M, Keller I, Fan S, et al. The genomic substrate for adaptive radiation in African cichlid fish. Nature. 2014;513:375–81.
6. Bradnam KR, Fass JN, Alexandrov A, Baranay P, Bechner M, Birol İ, et al. Assemblathon 2: evaluating de novo methods of genome assembly in three vertebrate species. Gigascience. 2013;2.
7. Korlach J. Returning to more finished genomes. Genomics Data. 2014;2:46–8.
8. Chin C-S, Peluso P, Sedlazeck FJ, Nattestad M, Concepcion GT, Clum A, et al. Phased

diploid genome assembly with single molecule real-time sequencing. Nat Methods. 2016;13:1050–4.
9. Koren S, Walenz BP, Berlin K, Miller JR, Bergman NH, Phillippy AM. Canu: Scalable and accurate long-read assembly via adaptive κ-mer weighting and repeat separation. Genome Res. 2017;27:722–36.
10. Chaisson MJP, Huddleston J, Dennis MY, Sudmant PH, Malig M, Hormozdiari F, et al. Resolving the complexity of the human genome using single-molecule sequencing. Nature. Nature Publishing Group; 2014;517:608–11.
11. Gordon D, Huddleston J, Chaisson MJP, Hill CM, Kronenberg ZN, Munson KM, et al. Long-read sequence assembly of the gorilla genome. Science. 2016;352.
12. Zimin A V., Puiu D, Hall R, Kingan S, Clavijo BJ, Salzberg SL. The first near-complete assembly of the hexaploid bread wheat genome, *Triticum aestivum*. Gigascience. 2017;
13. Conte MA, Kocher TD. An improved genome reference for the African cichlid, *Metriaclima zebra*. BMC Genomics. BMC Genomics; 2015;16:724.
14. Conte MA, Gammerdinger WJ, Bartie KL, Penman DJ, Kocher TD. A high quality assembly of the Nile Tilapia (*Oreochromis niloticus*) genome reveals the structure of two sex determination regions. BMC Genomics. BMC Genomics; 2017;18:341.
15. Feldberg E, Ivan J, Porto R, Antonio L, Bertollo C. Chromosomal changes and adaptation of cichlid fishes during evolution. AL Val BG Kapoor (eds), Fish Adapt. 2003;285–309.
16. Ferreira IA, Poletto AB, Kocher TD, Mota-Velasco JC, Penman DJ, Martins C. Chromosome evolution in African cichlid fish: contributions from the physical mapping of repeated DNAs. Cytogenet Genome Res. 2010;129:314–22.
17. Poletto AB, Ferreira IA, Cabral-de-Mello DC, Nakajima RT, Mazzuchelli J, Ribeiro HB, et al. Chromosome differentiation patterns during cichlid fish evolution. BMC Genet. 2010;11:50.
18. Carbone L, Alan Harris R, Gnerre S, Veeramah KR, Lorente-Galdos B, Huddleston J, et al. Gibbon genome and the fast karyotype evolution of small apes. Nature. 2014;513:195–201.
19. Damas J, O’Connor R, Farré M, Lenis VPE, Martell HJ, Mandawala A, et al. Upgrading short-read animal genome assemblies to chromosome level using comparative genomics and a universal probe set. Genome Res. 2017;27:875–84.
20. Lewin HA, Larkin DM, Pontius J, O’Brien SJ. Every genome sequence needs a good map. Genome Res. 2009;19:1925–8.
21. Hellmann I, Ebersberger I, Ptak SE, Pääbo S, Przeworski M. A neutral explanation for the correlation of diversity with recombination rates in humans. Am J Hum Genet. 2003;72:1527–35.
22. Wolf JBW, Ellegren H. Making sense of genomic islands of differentiation in light of speciation. Nat Rev Genet. Nature Publishing Group; 2017;18:87–100.
23. Joshi R, Árnyasi M, Lien S, Gjøen HM, Alvarez AT, Kent M. Development and Validation of 58K SNP-Array and High-Density Linkage Map in Nile Tilapia (*O . niloticus*). Front Genet. 2018;9:1–15.
24. Begun DJ, Aquadro CF. Levels of naturally occurring DNA polymorphism correlate with recombination rates in D. melanogaster. Nature. 1992;356:519–20.
25. Kulathinal RJ, Bennett SM, Fitzpatrick CL, Noor MAF. Fine-scale mapping of recombination rate in *Drosophila* refines its correlation to diversity and divergence. Proc Natl Acad Sci. 2008;105:10051–6.
26. Charlesworth D. Evolution of recombination rates between sex chromosomes. Phil Trans R Soc B. 2017;372:20160456.

27. Stapley J, Feulner PGD, Johnston SE, Santure AW, Smadja CM. Variation in recombination frequency and distribution across eukaryotes: Patterns and processes. Philos Trans R Soc B Biol Sci. 2017;372:20160455.
28. Gante HF, Matschiner M, Malmstrøm M, Jakobsen KS, Jentoft S, Salzburger W. Genomics of speciation and introgression in Princess cichlid fishes from Lake Tanganyika. Mol Ecol. 2016;
29. Werren JH. Selfish genetic elements, genetic conflict, and evolutionary innovation. Proc Natl Acad Sci U S A. 2011;108 Suppl:10863–70.
30. Ser JR, Roberts RB, Kocher TD. Multiple interacting loci control sex determination in Lake Malawi cichlid fish. Evolution (N Y). 2010;64:486–501.
31. Roberts RB, Ser JR, Kocher TD. Sexual conflict resolved by invasion of a novel sex determiner in Lake Malawi cichlid fishes. Science. 2009;326:998–1001.
32. Gammerdinger WJ, Conte MA, Acquah EA, Roberts RB, Kocher TD. Structure and decay of a proto-Y region in Tilapia, *Oreochromis niloticus*. BMC Genomics. 2014;15:975.
33. Gammerdinger WJ, Conte MA, Baroiller J-F, D'Cotta H, Kocher TD. Comparative analysis of a sex chromosome from the blackchin tilapia, *Sarotherodon melanotheron*. BMC Genomics. 2016;17:808.
34. Clark FE, Conte MA, Ferreira-Bravo IA, Poletto AB, Martins C, Kocher TD. Dynamic sequence evolution of a sex-associated B chromosome in lake Malawi cichlid fish. J Hered. 2017;108:53–62.
35. Kasahara M, Naruse K, Sasaki S, Nakatani Y, Qu W, Ahsan B, et al. The medaka draft genome and insights into vertebrate genome evolution. Nature. 2007;447:714–9.
36. Roberts NB, Juntti SA, Coyle KP, Dumont BL, Stanley MK, Ryan AQ, et al. Polygenic sex determination in the cichlid fish. BMC Genomics. 2016;1–13.
37. Mazzuchelli J, Kocher TD, Yang F, Martins C. Integrating cytogenetics and genomics in comparative evolutionary studies of cichlid fish. BMC Genomics. 2012;13.
38. Liu F, Sun F, Li J, Xia JH, Lin G, Tu RJ, et al. A microsatellite-based linkage map of salt tolerant tilapia (*Oreochromis mossambicus* x *Oreochromis spp.*) and mapping of sex-determining loci. BMC Genomics. 2013;14:1–14.
39. Ichikawa K, Tomioka S, Suzuki Y, Nakamura R, Doi K, Yoshimura J, et al. Centromere evolution and CpG methylation during vertebrate speciation. Nat Commun. 2017;8:1833.
40. Amores A, Catchen J, Nanda I, Warren W, Walter R, Schartl M, et al. A RAD-tag genetic map for the platyfish (*Xiphophorus maculatus*) reveals mechanisms of karyotype evolution among teleost fish. Genetics. 2014;197:625–41.
41. Takahashi K, Terai Y, Nishida M, Okada N. A novel family of short interspersed repetitive elements (SINEs) from cichlids: the patterns of insertion of SINEs at orthologous loci support the proposed monophyly of four major groups of cichlid fishes in Lake Tanganyika. Mol Biol Evol. 1998;15:391–407.
42. Takahashi K, Okada N. Mosaic structure and retropositional dynamics during evolution of subfamilies of short interspersed elements in African cichlids. Mol Biol Evol. 2002;19:1303–12.
43. Chuong EB, Elde NC, Feschotte C. Regulatory activities of transposable elements: From conflicts to benefits. Nat Rev Genet. 2017;18:71–86.
44. Lee B, Hulata G, Kocher TD. Two unlinked loci controlling the sex of blue tilapia (*Oreochromis aureus*). Heredity (Edinb). 2004;92:543–9.
45. Gregory TR. Animal Genome Size Database [Internet]. 2016. Available from: http://www.genomesize.com
46. Hedrick PW. Genetics of Populations, Fourth Edition. Jones and Bartlett Publishers,

Sudbury, MA; 2011.
47. Hill WG, Robertson A. Linkage disequilibrium in finite populations. Theor Appl Genet. 1968;38:226–31.
48. Ohta T, Kimura M. Linkage disequilibrium at steady state determined by random genetic drift and recurrent mutation. Genetics. 1969;63:229.
49. Husemann M, Nguyen R, Ding B, Danley PD. A genetic demographic analysis of Lake Malawi rock-dwelling cichlids using spatio-temporal sampling. Mol Ecol. 2015;24:2686–701.
50. O'Quin CT, Drilea AC, Conte MA, Kocher TD. Mapping of pigmentation QTL on an anchored genome assembly of the cichlid fish , *Metriaclima zebra*. BMC Genomics. 2013;14:1.
51. Albertson RC, Powder KE, Hu Y, Coyle KP, Roberts RB, Parsons KJ, et al. Genetic basis of continuous variation in the levels and modular inheritance of pigmentation in cichlid fishes. Mol Ecol. 2014;23:5135–50.
52. Nandamuri SP, Conte MA, Carleton KL. Multiple trans QTL and one cis -regulatory deletion are associated with the differential expression of cone opsins in African cichlids. BMC Genomics. BMC Genomics; 2018;1–17.
53. O'Leary NA, Wright MW, Brister JR, Ciufo S, Haddad D, McVeigh R, et al. Reference sequence (RefSeq) database at NCBI: Current status, taxonomic expansion, and functional annotation. Nucleic Acids Res. 2016;44:D733–45.
54. Franck JPC, Kornfield I, Wright JM. The utility of SATA satellite dna sequences for inferring phylogenetic relationships among the three major genera of tilapiine cichlid fishes. Mol Phylogenet Evol. 1994;3:10–6.
55. Melters DP, Bradnam KR, Young HA, Telis N, May MR, Ruby JG, et al. Comparative analysis of tandem repeats from hundreds of species reveals unique insights into centromere evolution. Genome Biol. BioMed Central Ltd; 2013;14:R10.
56. Kumar S, Stecher G, Suleski M, Hedges SB. TimeTree: A Resource for Timelines, Timetrees, and Divergence Times. Mol Biol Evol. 2017;34:1812–9.
57. Ferreira IA, Martins C. Physical chromosome mapping of repetitive DNA sequences in Nile tilapia *Oreochromis niloticus*: Evidences for a differential distribution of repetitive elements in the sex chromosomes. Micron. 2008;39:411–8.
58. Guyon R, Rakotomanga M, Azzouzi N, Coutanceau JP, Bonillo C, D'Cotta H, et al. A high-resolution map of the Nile tilapia genome: a resource for studying cichlids and other percomorphs. BMC Genomics. 2012;13:222.
59. Eshel O, Shirak A, Weller JI, Hulata G, Ron M. Linkage and Physical Mapping of Sex Region on LG23 of Nile Tilapia (*Oreochromis niloticus*). G3 Genes, Genomes, Genet. 2012;2:35–42.
60. Peterson EN, Cline ME, Moore EC, Roberts NB, Roberts RB, Moore EC. Genetic sex determination in Astatotilapia calliptera, a prototype species for the Lake Malawi cichlid radiation. Sci Nat. The Science of Nature; 2017;104:41.
61. York RA, Patil C, Abdilleh K, Johnson Z V., Conte MA, Genner MJ, et al. Behavior-dependent cis regulation reveals genes and pathways associated with bower building in cichlid fishes. Proc Natl Acad Sci. 2018;115:E11081–90.
62. Terai Y, Takahashi K, Okada N. SINE cousins: the 3'-end tails of the two oldest and distantly related families of SINEs are descended from the 3' ends of LINEs with the same genealogical origin. Mol Biol Evol. 1998;15:1460–71.
63. Simão FA, Waterhouse RM, Ioannidis P, Kriventseva E V., Zdobnov EM. BUSCO: Assessing genome assembly and annotation completeness with single-copy orthologs.

Bioinformatics. 2015;31:3210–2.
64. Waterhouse RM, Seppey M, Simao FA, Manni M, Ioannidis P, Klioutchnikov G, et al. BUSCO applications from quality assessments to gene prediction and phylogenomics. Mol Biol Evol. 2018;35:543–8.
65. Maylandia zebra Annotation Report [Internet]. [cited 2018 May 23]. Available from: https://www.ncbi.nlm.nih.gov/genome/annotation_euk/Maylandia_zebra/103/
66. Maylandia zebra Annotation Report [Internet]. [cited 2018 May 23]. Available from: https://www.ncbi.nlm.nih.gov/genome/annotation_euk/Maylandia_zebra/104/
67. Dolgin ES, Charlesworth B. The effects of recombination rate on the distribution and abundance of transposable elements. Genetics. 2008;178:2169–77.
68. Bourque G, Burns KH, Gehring M, Gorbunova V, Seluanov A, Hammell M, et al. Ten things you should know about transposable elements. Genome Biology; 2018;1–12.
69. Bennetzen JL, Wang H. The contributions of transposable elements to the structure, function, and evolution of plant genomes. Annu Rev Plant Biol. 2014;65:505–30.
70. Carvalho CMB, Lupski JR. Mechanisms underlying structural variant formation in genomic disorders. Nat Rev Genet. 2016;17:224.
71. Deininger PL, Moran J V., Batzer MA, Kazazian HH. Mobile elements and mammalian genome evolution. Curr Opin Genet Dev. 2003;13:651–8.
72. Ade C, Roy-Engel AM, Deininger PL. Alu elements: An intrinsic source of human genome instability. Curr Opin Virol [Internet]. Elsevier B.V.; 2013;3:639–45. Available from: http://dx.doi.org/10.1016/j.coviro.2013.09.002
73. Sakamoto T, Danzmann RG, Gharbi K, Howard P, Ozaki A, Khoo SK, et al. A microsatellite linkage map of rainbow trout (*Oncorhynchus mykiss*) characterized by large sex-specific differences in recombination rates. Genetics. 2000;155:1331–45.
74. Moen T, Hoyheim B, Munck H, Gomez-Raya L. A linkage map of Atlantic salmon (*Salmo salar*) reveals an uncommonly large difference in recombination rate between the sexes. Anim Genet. 2004;35:81–92.
75. Gharbi K, Gautier A, Danzmann RG, Gharbi S, Sakamoto T, Høyheim B, et al. A linkage map for brown trout (*Salmo trutta*): Chromosome homeologies and comparative genome organization with other salmonid fish. Genetics. 2006;172:2405–19.
76. Rexroad CE, Palti Y, Gahr SA, Vallejo RL. A second generation genetic map for rainbow trout (*Oncorhynchus mykiss*). BMC Genet. 2008;9:1–14.
77. Sardell JM, Cheng C, Dagilis AJ, Ishikawa A, Kitano J, Peichel CL, et al. Sex differences in recombination in sticklebacks. G3 Genes, Genomes, Genet. 2018;g3-200166.
78. Castaño-Sánchez C, Fuji K, Ozaki A, Hasegawa O, Sakamoto T, Morishima K, et al. A second generation genetic linkage map of Japanese flounder (Paralichthys olivaceus). BMC Genomics. 2010;11:554.
79. Singer A, Perlman H, Yan Y, Walker C, Corley-Smith G, Brandhorst B, et al. Sex-specific recombination rates in zebrafish (*Danio rerio*). Evol Heal Dis. 2002;160:649–57.
80. Roesti M, Moser D, Berner D. Recombination in the threespine stickleback genome - Patterns and consequences. Mol Ecol. 2013;22:3014–27.
81. Zeng Q, Fu Q, Li Y, Waldbieser G, Bosworth B, Liu S, et al. Development of a 690 K SNP array in catfish and its application for genetic mapping and validation of the reference genome sequence. Sci Rep. 2017;7:1–14.
82. Charlesworth B. The evolution of sex chromosomes. Science (80-). 1991;251:1030–3.
83. Cnaani A, Lee BY, Zilberman N, Ozouf-Costaz C, Hulata G, Ron M, et al. Genetics of sex

determination in tilapiine species. Sex Dev. 2008;2:43–54.
84. Li M, Sun Y, Zhao J, Shi H, Zeng S, Ye K, et al. A tandem duplicate of Anti-Müllerian Hormone with a missense SNP on the Y chromosome is essential for male sex determination in Nile Tilapia, Oreochromis niloticus. PLoS Genet. 2015;11:1–23.
85. Gammerdinger WJ, Conte MA, Sandkam BA, Penman DJ, Kocher TD. Characterization of sex chromosomes in three deeply diverged species of Pseudocrenilabrinae (Teleostei: Cichlidae). Hydrobiologia. Springer International Publishing; 2018;6:1–12.
86. O'Quin CT. The genetic basis of pigment pattern differentiation in Lake Malawi African cichlids. Thesis Dissertation [Internet]. University of Maryland; 2014. Available from: http://hdl.handle.net/1903/15212
87. Guyomard R, Boussaha M, Krieg F, Hervet C, Quillet E. A synthetic rainbow trout linkage map provides new insights into the salmonid whole genome duplication and the conservation of synteny among teleosts. BMC Genet. 2012;13:1–12.
88. Sevim V, Bashir A, Chin CS, Miga KH. Alpha-CENTAURI: Assessing novel centromeric repeat sequence variation with long read sequencing. Bioinformatics. 2016;32:1921–4.
89. Jain M, Koren S, Quick J, Rand AC, Sasani TA, Tyson JR, et al. Nanopore sequencing and assembly of a human genome with ultra-long reads. Nat Biotechnol. 2018;36:338.
90. Jain M, Olsen HE, Turner DJ, Stoddart D, Bulazel K V., Paten B, et al. Linear assembly of a human centromere on the Y chromosome. Nat Biotechnol. 2018;36:321–3.
91. Franck J, Wright J, McAndrew B. Genetic variability in a family of satellite DNAs from tilapia (Pisces: Cichlidae). Genome. 1992;35:719–25.
92. Feulner PGD, Schwarzer J, Haesler MP, Meier JI, Seehausen O. A dense linkage map of Lake Victoria cichlids improved the *Pundamilia* genome assembly and revealed a major QTL for sex-determination. G3 Genes, Genomes, Genet. 2018;8:2411–20.
93. Santos ME, Braasch I, Boileau N, Meyer BS, Sauteur L, Böhne A, et al. The evolution of cichlid fish egg-spots is linked with a cis-regulatory change. Nat Commun. 2014;5:5149.
94. Schulte JE, O'Brien CS, Conte MA, O'Quin KE, Carleton KL. Interspecific variation in Rx1 expression controls opsin expression and causes visual system diversity in African cichlid fishes. Mol Biol Evol. 2014;31:2297–308.
95. Koren S, Rhie A, Walenz BP, Dilthey AT, Bickhart DM, Kingan SB, et al. *De novo* assembly of haplotype-resolved genomes with trio binning. Nat Biotechnol. 2018;36:1174.
96. Novak AM, Hickey G, Garrison E, Blum S, Connelly A, Dilthey A, et al. Genome graphs. bioRxiv. 2017;101378.
97. Paten B, Novak AM, Eizenga JM, Garrison E. Genome graphs and the evolution of genome inference. Genome Res. 2017;27:665–76.
98. Eggertsson HP, Jonsson H, Kristmundsdottir S, Hjartarson E, Kehr B, Masson G, et al. Graphtyper enables population-scale genotyping using pangenome graphs. Nat Genet. 2017;49:1654–60.
99. Dilthey A, Cox C, Iqbal Z, Nelson MR, McVean G. Improved genome inference in the MHC using a population reference graph. Nat Genet. 2015;47:682–8.
100. Miga KH, Newton Y, Jain M, Altemose N, Willard HF, Kent WJ. Centromere reference models for human chromosomes X and Y satellite arrays. 2014;697–707.
101. Rastas P, Calboli FCF, Guo B, Shikano T, Merilä J. Construction of ultradense linkage maps with Lep-MAP2: Stickleback F2 recombinant crosses as an example. Genome Biol Evol. 2015;8:78–93.
102. Lee B-Y, Lee W-J, Streelman JT, Carleton KL, Howe AE, Hulata G, et al. A second-

generation genetic linkage map of tilapia (*Oreochromis spp.*). Genetics. 2005;170:237–44.
103. Chang CC, Chow CC, Tellier LCAM, Vattikuti S, Purcell SM, Lee JJ. Second-generation PLINK: Rising to the challenge of larger and richer datasets. Gigascience. 2015;4:7.
104. Li H. Aligning sequence reads , clone sequences and assembly contigs with BWA-MEM. arXiv. 2013;00:1–2.
105. Walker BJ, Abeel T, Shea T, Priest M, Abouelliel A, Sakthikumar S, et al. Pilon: An integrated tool for comprehensive microbial variant detection and genome assembly improvement. PLoS One. 2014;9:e112963.
106. PacificBiosciences/SMRT-Analysis [Internet]. [cited 2014 May 5]. Available from: https://github.com/PacificBiosciences/SMRT-Analysis
107. Catchen J, Amores A. Chromonomer [Internet]. Available from: http://catchenlab.life.illinois.edu/chromonomer/
108. Chaisson MJ, Tesler G. Mapping single molecule sequencing reads using basic local alignment with successive refinement (BLASR): application and theory. BMC Bioinformatics. 2012;13:238.
109. Thorvaldsdóttir H, Robinson JT, Mesirov JP. Integrative Genomics Viewer (IGV): High-performance genomics data visualization and exploration. Brief Bioinform. 2013;14:178–92.
110. Wu TD, Watanabe CK. GMAP: a genomic mapping and alignment program for mRNA and EST sequences. Bioinformatics. 2005;21:1859–75.
111. Smit, AFA, Hubley, R & Green P. RepeatMasker Open-4.0 [Internet]. 2010. Available from: www.repeatmasker.org
112. Smit, AFA, Hubley R. RepeatModeler Open-1.0 [Internet]. 2010. Available from: www.repeatmasker.org
113. Bao W, Kojima KK, Kohany O. Repbase Update, a database of repetitive elements in eukaryotic genomes. Mob DNA. 2015;6:11.
114. Akalin A, Franke V, Vlahoviček K, Mason CE, Schübeler D. Genomation: A toolkit to summarize, annotate and visualize genomic intervals. Bioinformatics. 2015;31:1127–9.
115. Kurtz S, Phillippy A, Delcher AL, Smoot M, Shumway M, Antonescu C, et al. Versatile and open software for comparing large genomes. Genome Biol. 2004;5:R12.
116. Nattestad M, Chin C-S, Schatz MC. Ribbon: Visualizing complex genome alignments and structural variation. bioRxiv. 2016;0344:82123.
117. Gel B, Serra E. KaryoploteR: An R/Bioconductor package to plot customizable genomes displaying arbitrary data. Bioinformatics. 2017;33:3088–90.

Figure legends

Figure 1. A) Chromosome mapping of SATA satellite DNA in *O. niloticus* reproduced and modified with permission from [16]. The SATA sequences are labelled in yellow against the background staining with propidium iodide. B) Giemsa-stained karyograms of the Lake Malawi *Metriaclima lombardoi* reproduced and modified with permission from [34]. LG3 in *O. niloticus* (A) and LG7 in *Metriaclima* (B) are labeled based on [37].

Figure 2. Comparative alignment of LG23 in *M. zebra* and *O. niloticus*. Centromere repeats in each assembly are indicated by large black triangles. Anchored contigs in each assembly are shown as red arrows indicating the orientation of each contig.

Figure 3. Comparison of the four genetic maps relative to M_zebra_UMD2 for LG7, LG11, LG20 and LG23. Maps for all LGs are provided in Additional File H.

Figure 4. O_niloticus_UMD_NMBU LG7 is an ancient cichlid-specific fusion corresponding to medaka HSOK 12 and 6. Female (red) and male *O. niloticus* recombination curves are shown along with linkage disequilibrium ($r^2 > 0.97$) in black. Alignment of LG7 to medaka HSOK 12 and 6 are shown on the bottom.

Figure 5. O_niloticus_UMD_NMBU LG23 is an ancient cichlid-specific fusion corresponding to medaka HSOK 2 and part of medaka HSOK 4. Female (red) and male *O. niloticus* recombination curves are shown along with linkage disequilibrium ($r^2 > 0.97$) in black. Alignment of LG7 to medaka HSOK 12 and 6 are shown on the bottom.

Figure 6. Summary of large structural changes in African cichlid genomes. (a) Chromosome fusion events on LG7 and LG23. (b) Expansion of repetitive LG3 in the *Oreochromis* lineage likely in conjunction with its role as ZW sex chromosome. (c) Putative inversions in *Aulonocara* on LG11 and LG20. Chromosomes that have undergone a large (> 6Mb) structural change are displayed. Other chromosomes that have not undergone a large change in the 7 cichlid species studied are not shown. Likely changes in meta-/sub-metacentric ("m/sm") and subtelomeric/acrocentric ("st/a") chromosomes from Malawi and *O. niloticus* are labeled. Recombination rates are shown as LOESS smoothed curves. Male and female recombination rate curves are shown for *O. niloticus*. Typical recombination rate curves for Lake Malawi cichlids are usually represented by the *M. mbenjii* x *A. koningsi* map. Recombination curves in crosses involving *Aulonocara* are shown for LG11 and LG20 to highlight large differences in recombination on those particular chromosomes. Several rearrangements, such as LG2, are more complex than depicted in this figure. Refer to Additional File E for detailed whole genome alignments and Additional Files G and H for detailed recombination plots. Divergence times were obtained from [56].

Figure 7. Comparison of the repeat landscape in the *M. zebra* and *O. niloticus* genome assemblies.

Additional files

Additional File A – Read length distribution of the 65X coverage *M. zebra* PacBio reads.

Additional File B - Size distribution of the M_zebra_UMD2 assembled haplotigs and theoretical recombination rate for several different effective population sizes.

Additional File C – M_zebra_UMD2 FALCON p-contigs where markers from two or more different LGs maps aligned, indicating a potential inter-LG misassembly.

Additional File D – Screenshot of IGV view to inspect potential misassemblies. In this example, a misassembly on this contig was confirmed at position 420,665 (indicated by the white arrows). The top red box shows the portion of the contig that is being visualized. LG17 markers aligned at 186kb and 308kb, while LG10a markers aligned at 760kb and 1.6Mbp as indicated by the red arrows. The top two tracks below that are the read coverage plots for the PacBio read alignments against the diploid and haploid sets of contigs. There is a sharp decrease in PacBio read coverage at the misassembly location. The track below shows 40kb mate-pair alignments and also shows no clone coverage at the location of the misassembly.

Additional File E – *M. zebra* assembly contigs anchored with each of the 4 maps and aligned to O_niloticus_UMD_NMBU (indicated as black on bottom with contigs in red for each panel). Centromeres indicated with black triangles. Contigs are represented as red lines above each respective assembly.

Additional File F – (a) F_{ST} comparison of male and female *O. aureus* LG3 ZW. (b) *O. niloticus* recombination curve of LG3 from Additional File G.

Additional File G – *O. niloticus* recombination curves for females (red) and males (blue). Centromere repeats are displayed as green triangles where applicable. X-axis represent the location along the anchored LG. Left Y-axis represents linkage disequilibrium (black points, $r^2 > 0.97$) and right Y-axis shows the map location for each marker.

Additional File H – Comparison of recombination in the four Lake Malawi genetic maps. LGs from maps that needed to be reversed from their original published order are indicated in Additional File M. The detected misassembly on LG12 is included on page 13 of this file.

Additional File I – Histogram of read length distributions for the 44X coverage PacBio read sets from *M. zebra* and *O. niloticus*. These read sets were used for the closer comparison of recent repeats between the two species.

Additional File J – Comparison of the repeat landscape in the *M. zebra* and *O. niloticus* genome assemblies using same assembly parameters and 44X coverage PacBio data. Note that the Y-axis is different for the two repeat landscapes.

Additional File K – Spreadsheet of TE insertion locations by defining promoter regions as either 1kbp or 15kbp.

Additional File L – Comparison of the repeat landscape in the three *M. zebra* assembly versions.

Additional File M – Table of the orientation of Lake Malawi recombination maps for each LG. The forward and reverse orientation information of each map was used to generate recombination plots in the same orientation for Additional File H.

Click here to access/download;Figure;Figure1_karyotypes.pdf

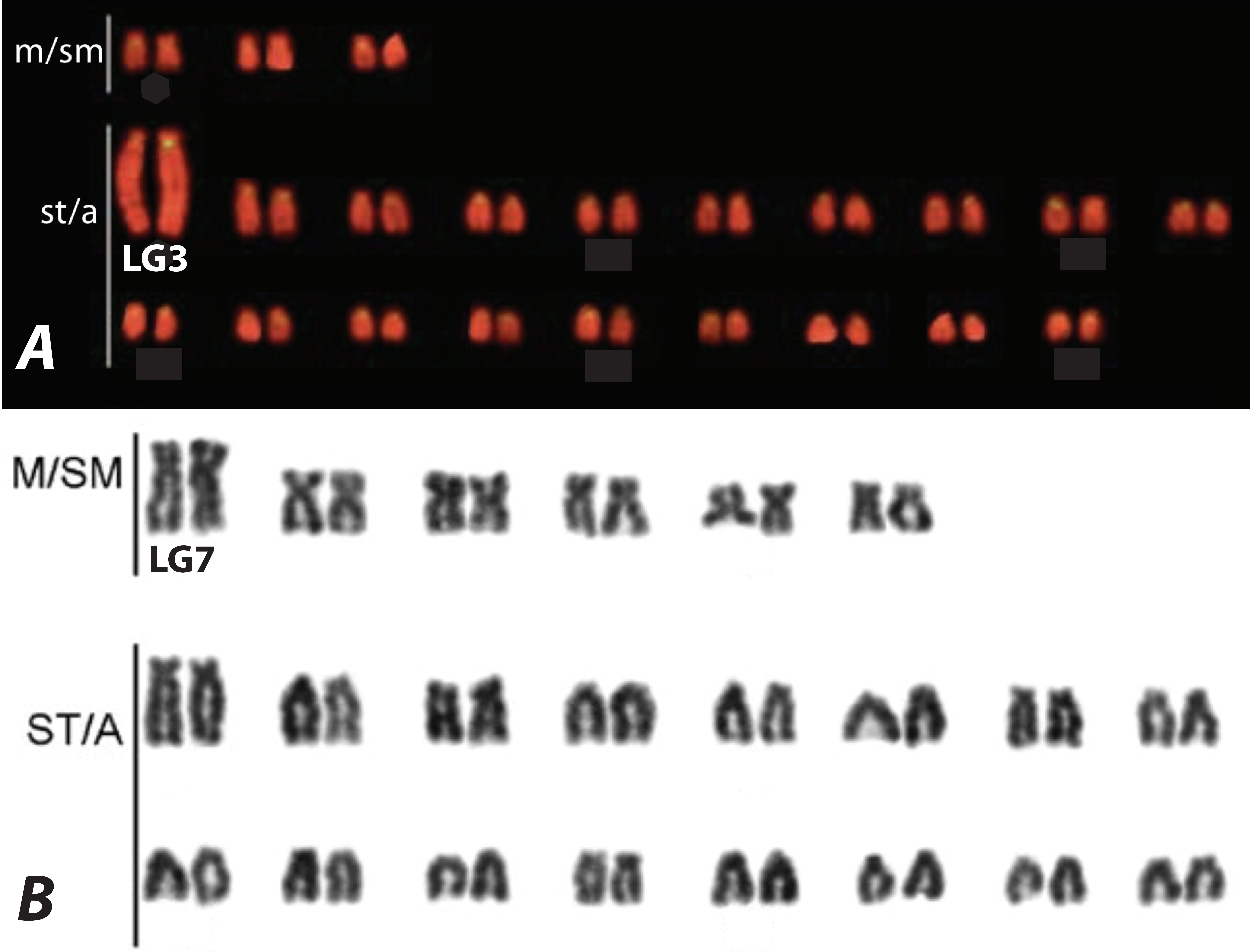

Figure2

Click here to access/download;Figure;Figure2_LG23_comparison.png

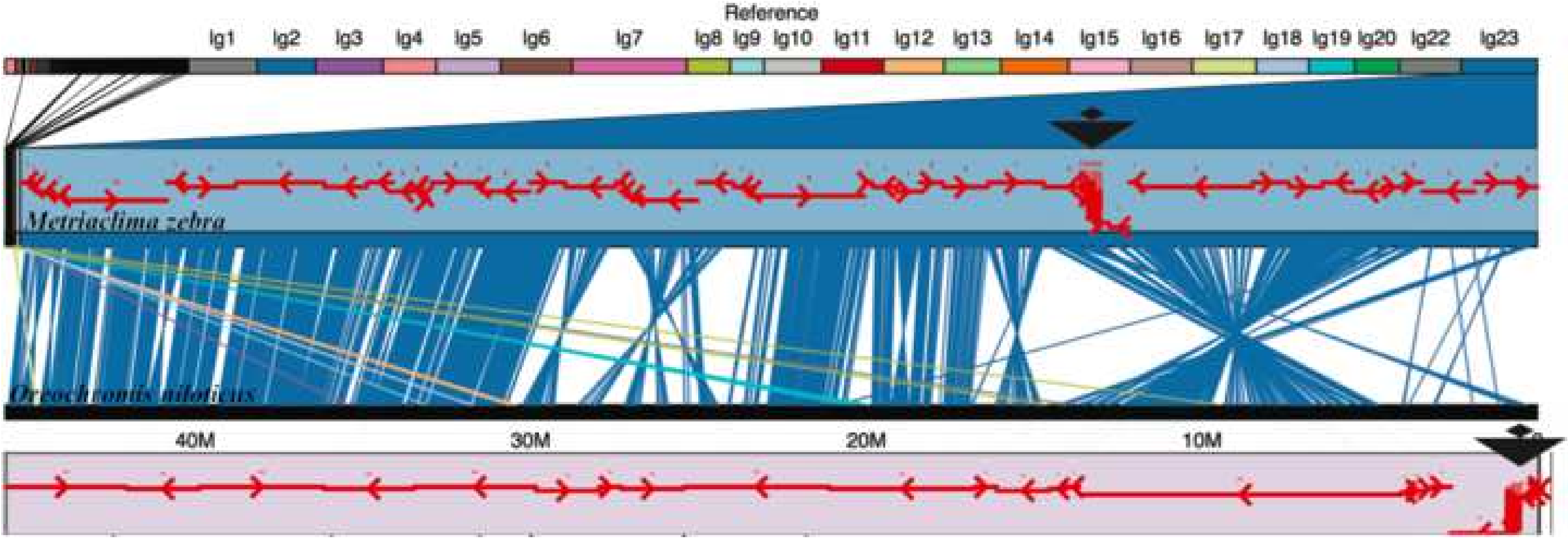

Figure3

Click here to access/download;Figure;Figure3_select_Malawi_recombination.pdf

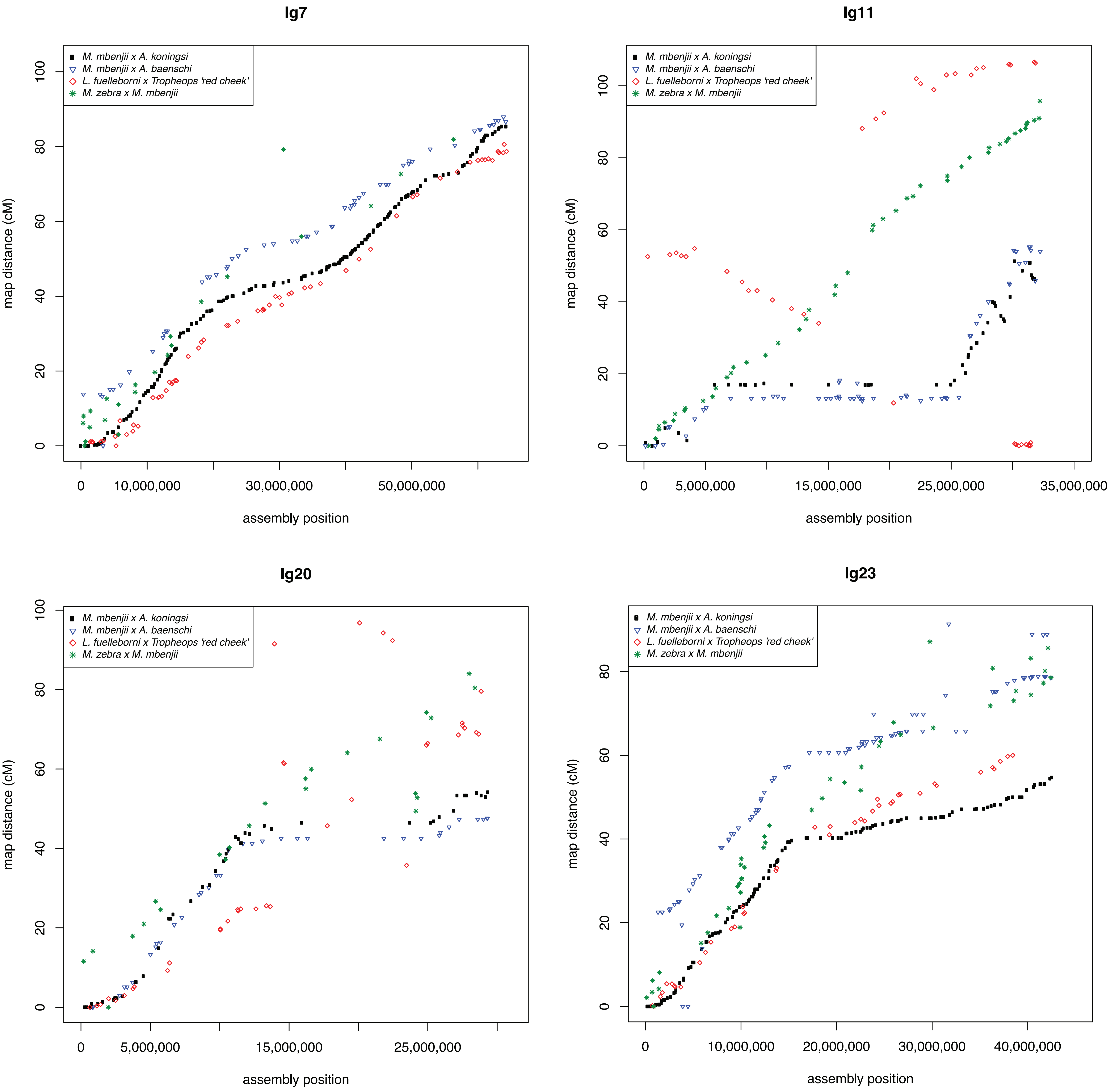

Click here to access/download;Figure;Figure4_LG7_fusion.pdf

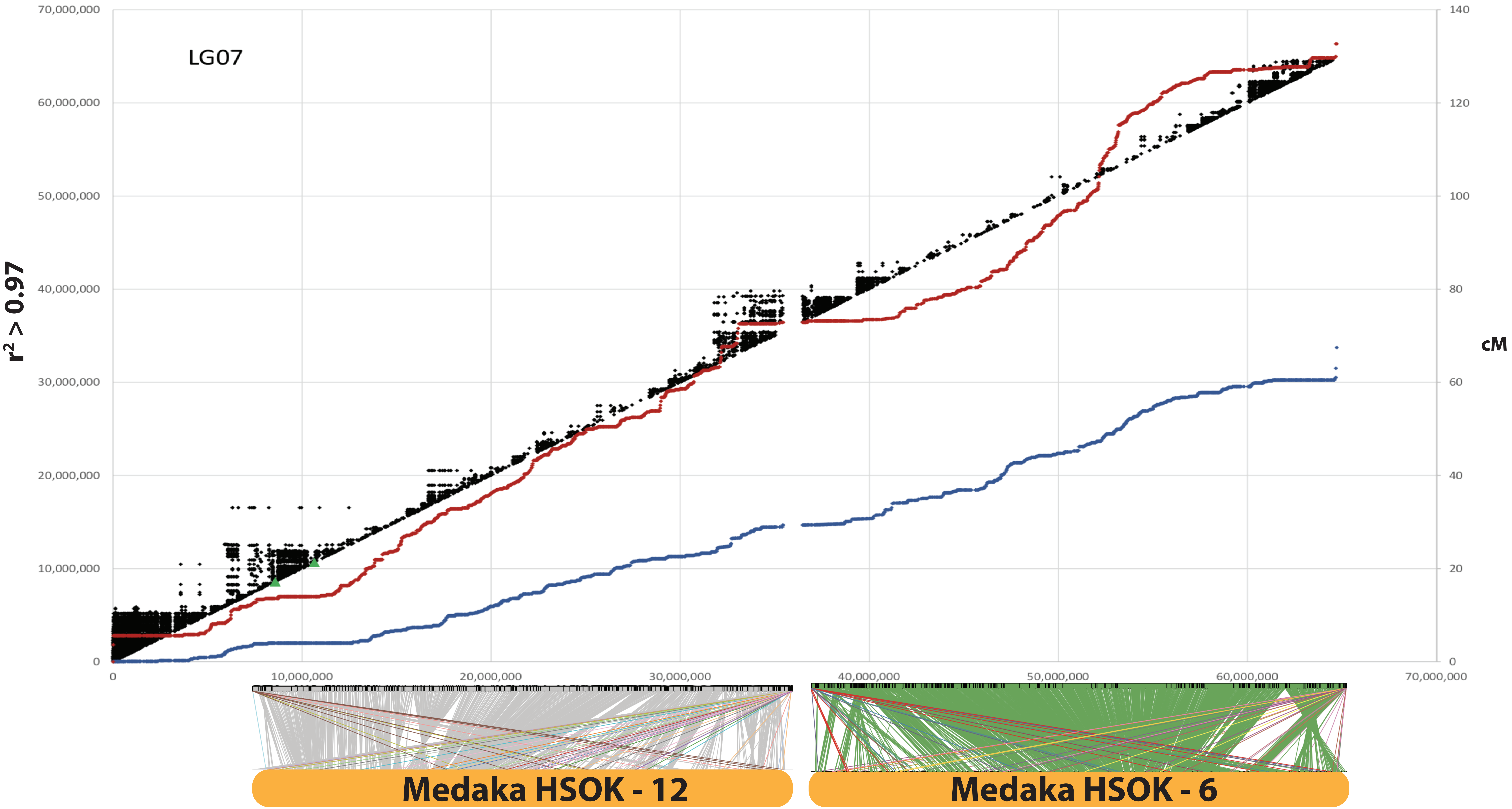

Figure5

Click here to access/download;Figure;Figure5_LG23_fusion.pdf

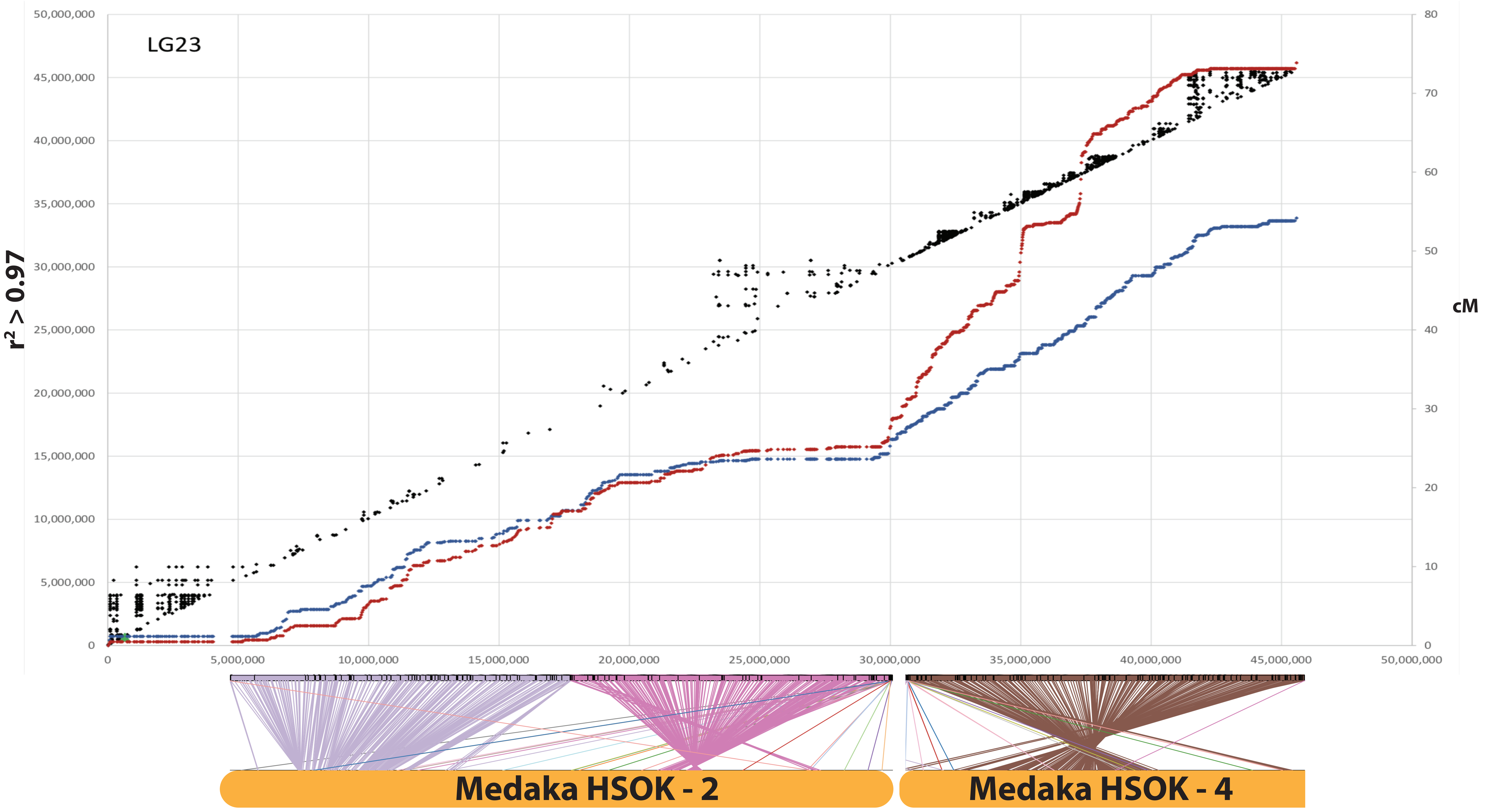

Click here to access/download;Figure;Figure6_summary_of_structural_changes.pdf

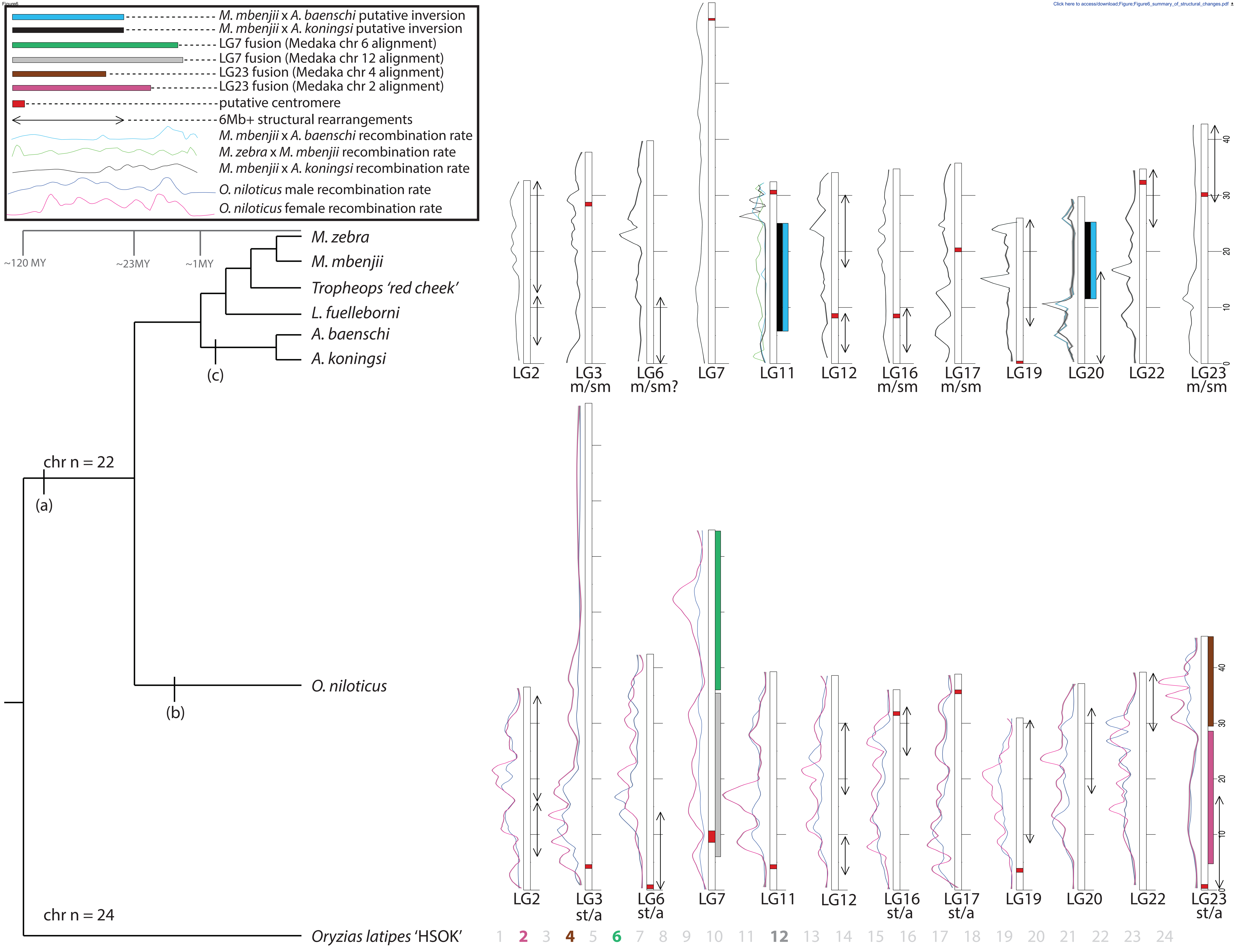

Figure7

Click here to access/download;Figure;Figure7_Repeat_landscape_comparison.pdf

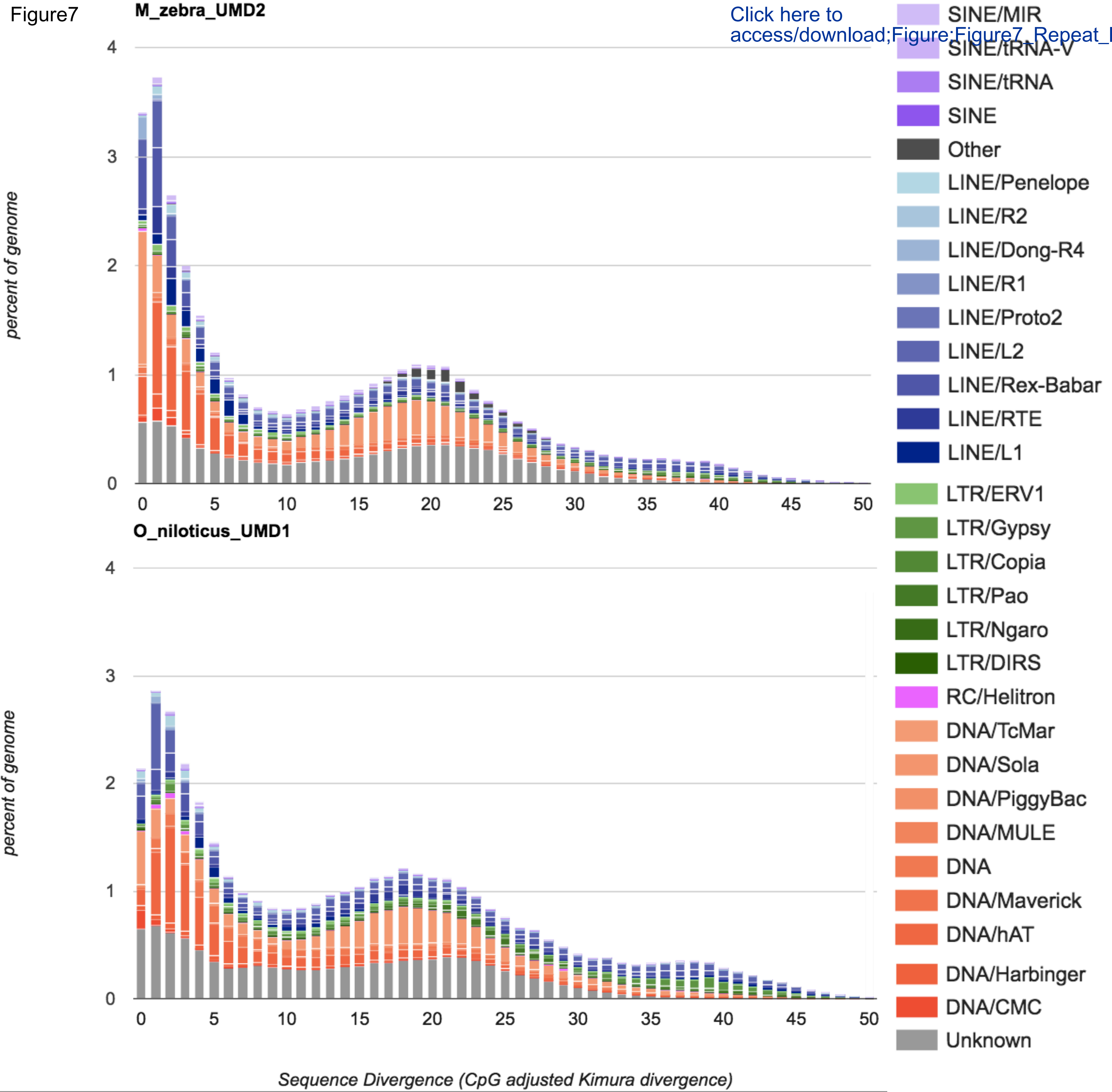

Click here to access/download
Supplementary Material
AdditionalFileA_M_zebra_65X_combined_chems_reads.
hist.pdf

Click here to access/download
Supplementary Material
AdditionalFileB_LD_and_haplotig_distribution_black_hist_redo.pdf

Click here to access/download
Supplementary Material
AdditionalFileC_chromonomer_misassembly_compariso
n.xlsx

Click here to access/download
Supplementary Material
AdditionalFileD_contig_000146F_1_1162490.pdf

AdditionalFileE

Click here to access/download
Supplementary Material
AdditionalFileE_whole_chromosome_alignments.pdf

AdditionalFileF

Click here to access/download
Supplementary Material
AdditionalFileF_LG3_Fst_recombination.pdf

AdditionalFileG

Click here to access/download
Supplementary Material
AdditionalFileG_tilapia_recombination_plots.pdf

Click here to access/download
Supplementary Material
AdditionalFileH_Lake_Malawi_recombination_plots.pdf

AdditionalFileI

Click here to access/download
Supplementary Material
AdditionalFileI_44X_read_length_distribution_comparison.pdf

Click here to access/download
Supplementary Material
AdditionalFileJ_TE_landscape_comparison.pdf

Click here to access/download
Supplementary Material
AdditionalFileK_TE_insertion_comparison_promoter_comparison.xlsx

Click here to access/download
Supplementary Material
AdditionalFileL_TE_landscape_M_zebra_assemblies.pdf

Click here to access/download
Supplementary Material
AdditionalFileM_map_orientation_comparison.xlsx

Response_to_Reviewers

Click here to access/download;Personal Cover;Response_to_reviewers.docx

We thank both reviewers for their careful reading of our manuscript. We have worked to significantly shorten and tighten the text. We detail our response to each of their specific comments in the paragraphs below.

Reviewer #1:

This paper by Conte and colleagues describes two new chromosome-scale genomic assemblies of African cichlids. The authors used multiple genetic maps to anchor contigs from high-coverage PacBio sequencing and correct misassemblies. Based on these two high-quality genomes and the genetic maps, the authors performed comprehensive comparative analyses of recombination landscapes, large-scale chromosomal rearrangements, and transposable element insertions. The paper presents extensive genomic resources, which will be valuable for future studies in the field.

However, the manuscript in its current form is highly descriptive and many parts of the paper are repetitive and very tedious to read. I'm convinced that the appeal of the paper for a broader readership could be improved considerably by shortening the main text and putting a focus on the biologically interesting aspects. The purely descriptive details could be presented more effectively in tables and figures or additional supporting materials. For instance, instead of the lengthy description of rearrangements, regions of unusual recombination, and putative sex determination loci, I would like to see a carefully designed summary figure, which provides the reader with a good overview of these events in the two genomes.

In its current form, many interesting aspects are buried in large amounts of text that provide information of little biological relevance. Most importantly, the discussion part should be written much more concisely, as it still largely descriptive and repeats most of the information that was already provided in the results section. Here, the authors should refrain from discussing every single aspect of their results and rather focus on the biological interpretation of the most interesting findings of the study.

Minor comments:
Page 4, line 15: Define "indel" here.

We have added this definition.

Page 4, line 52: Provide reference for PacBio sequencing.

We have added this reference.

Page 10, Table 1: Improve the labelling of the table. It is not immediately clear that the numbers represent base pairs.

We have improved the labeling of this table.

Page 11, line 42: "relatively complete" compared to what?

We have edited this sentence to clarify.

Page 11, line 35: The description here seems to imply that the final p-contigs are not phased. The p-contigs are phased within the borders of their associated haplotigs. Please provide a more detailed explanation of p-contigs and haplotigs here.

We have modified this sentence to remove the wrong implication and have added a more detailed explanation of p-contigs and haplotigs.

Page 11, lines 42-55: This part is unclear and should be rewritten. What are "theoretical sizes of heterozygous regions"? Do you mean the theoretical expectation of the distribution of distances between heterozygous sites? The associated Additional File A is completely unclear and needs a much more detailed explanation and legend, e.g. what is the relationship between the two x-axes (length in base pairs and recombination rate) or the two y-axes (frequency and E(r2))? How do you derive information about the completeness of haplotigs from this graph?

The axes of File A were poorly labelled, and our conclusions a little overextended. We have revised the text, figure and legend to improve the presentation of these results. The main point is that the size distribution of the homozygous regions in the genome assembly closely matches the expected size distribution of homozygous regions in an individual drawn from a population with an Ne of 1,000 – 2,000 individuals. We no longer make any claims about the completeness of the genome assembly from this graph.

Page 12, line 55: Provide full genus name for A. koningsi.

We have added this.

Page 13, Table 3: Why does the total length differ for the four different maps, given that it includes both anchored and unanchored contigs?

We had added a sentence that clarifies this.

Page 15, lines 34-42: Given that the anchoring is based on a combination of four different maps, is it possible that certain contigs are represented multiple times in the final assembly?

We have added a sentence that addresses this question.

Page 16, lines 4-47: Given that all genetic maps are from inter-species crosses, what are the expectations for inter-chromosomal rearrangements that are only present in one of the two species? It seems unlikely, that the given approach would have power to detect rearrangements in such cases.

The reviewer is correct that we would be unlikely to detect inter-chromosomal rearrangements in interspecific crosses among Lake Malawi species. Recombination in such regions in hybrids would likely produce inviable gametes or offspring, effectively suppressing recombination. So, such rearrangements would produce only the weak signal of reduced recombination through these regions, which we would not likely detect.

Page 16 line 34: "… at most 1% of these Lake Malawi genomes is affected by inter-chromosomal rearrangements …"

We have deleted this sentence. While we believe it is likely true, based on the limited amount of inter-chromosomal rearrangement between tilapia and Metriaclima, we currently do not have the data to support this statement.

Page 23, lines 46-48: This sentence doesn't make sense without a distance qualifier, i.e. significant linkage disequilibrium over extended physical distances.

We have added this qualifier.

Page 26, line 59: "Only one contig longer than 1Mbp was not anchored …"

We have modified this sentence.

Page 27, line 7: "Contigs in the M_zebra_UMD2 assembly were primarily anchored with" or "The M_zebra_UMD2 anchoring was primarily performed with"

We have changed this sentence.

Page 27, lines 46-48: The suggested link between TEs and chromosomal rearrangements seems a bit far-fetched. It appears more likely to me that low recombination is facilitating the enrichment of both TEs and rearrangements due to reduced Ne and therefore reduced efficacy of selection against slightly deleterious events in these regions.

We agree that there is an enrichment of TEs and rearrangements in regions of low recombination due to reduced efficacy of selection against slightly deleterious events in these regions and had already included a citation supporting that idea (Dolgin et al 2008). However, the link between TEs and chromosomal rearrangements is not far-fetched, and many studies have demonstrated this. We have added a sentence with several citations to support our claim.

Page 27, line 56: Not clear what is meant by "orthogonal mapping technologies" here. Alternative mapping technologies?

We have clarified this sentence.

Page 28, line 51: Genetic differentiation between what?

We have added to this sentence to be more explicit.

Page 30, line 33: Linkage group information is missing for the sex determination locus.

We have added this.

Page 30, lines 45-49: Rather the alleles of the sex determination system segregate in three crosses.

We have modified this sentence.

Page 32, lines 36-44: The connection between lack of evidence for a chromosome fusion event on LG3 and the accumulation of repetitive elements is not clear.

We have added some text to clarify this connection.

Page 34, line 29: What are "centromere-containing repeats" and does this refer to the ONSATA and TZSAT satellite sequences in the next sentence? Please rephrase this part.

We have rearranged and modified a sentence to make this clear.

Page 37, line 38: "will be able to purge"

This has been incorporated.

Page 40, line 9: Incomplete sentence

This sentence has been modified.

Page 40, line 29: Omit "that"

This sentence has been modified.

Page 40, line 44: Check reference. Reference to PLINK software doesn't make sense in this context.

We are unsure what the reviewer means, as one of the many things that PLINK can do is report LD statistics as described.

Reviewer #2:

General comments:

With their high speciation rates and remarkable phenotypic diversity, African cichlid fishes serve as a model for studying a broad range of evolutionary processes. In this manuscript, the authors use two high-quality genome assemblies and five genetic maps to investigate large scale structural variation, changes in broad-scale patterns of recombination, and to compare transposable element contents of the two assemblies. I think that the work is generally well executed and provides valuable new insights. The manuscript is also clearly written and the arguments are easy to follow. My only critical comment related to the current text is its repetitiveness, with the same statements often occurring in several places (see examples below). Especially the discussion is very long and, even though it offers some additional interpretation and explanations, it reads largely as a restatement of the analyses and the results.

Overall, I congratulate the authors on their work. I have some (relatively minor) comments and suggestions that the authors should consider before publication.

Specific comments/suggestions:

Data Description - this could be expanded, especially considering the GigaScience journal's focus. For example, for the new PacBio reads, I would be interested to see the read length distribution which is a crucial factor in genome assembly, especially influencing its contiguity and the types of repetitive elements that can be assembled. Perhaps the two "new genetic maps" should also have a brief description here? I don't

know what the GigaScience journal policy is, but this work is not fully reproducible until the "new genetic maps" are published. I highlighted this in the comments to the Editor.

We have added a figure of the read length distribution as well as several statistics that provides more information when evaluating the genome assembly as the reviewer suggests. We have also added a brief description of the two new genetic maps. The new genetic maps themselves have already been uploaded to the GigaScience FTP to make the work reproducible.

Analyses
Page 10 - "The anchored assembly of LG3 is 54.7% repetitive, compared to repeat rate of 37% genome-wide" Perhaps a clarification of what is meant "repetitive" would be useful. Do the numbers quoted by the authors refer to the portions of the chromosomes masked by the RepeatMasker software?

We have modified this sentence to clarify this point.

Page 11 -"To measure the completeness of the haplotigs, the theoretical sizes of heterozygous regions under null expectations of recombination rates and effective population sizes were compared to the size distribution of the haplotigs. Additional File A shows the…" I don't understand this analysis and the associated "Additional File A". What exactly are the "haplotigs"? What determines their boundaries? Perhaps the authors should elaborate, and/or at the very least provide a reference to the theory that predicts how large the haplotigs should be.

See response to Reviewer #1 above. The theory on this point is explained on page 540 (Figure 9.8) of Hedrick's "Genetics of Populations" textbook (4th edition). The theory is attributed to Hill and Robertson 1968 (TAG 38:226-231) and Ohta and Kimura 1969 (Genet. Res. 13:47).

Page 24 - "…on the same compute cluster…"
I am not sure how the identity of the compute cluster could affect genome assembly. Read length and quality are two more likely factors that are not mentioned.

We have added an additional file that provides a comparison of the read length distributions of both datasets used in this particular analysis as well as some additional sentences to explain our inferences based on this comment.

Page 24 - "M. zebra genome assembly has a noticeably larger amount of recent TE insertions."   Would be interesting to know where these insertions localised. For example, how many of these localised in gene promoters.

We have performed this requested analysis and provided the results as an additional file as well as providing additional text in the results and discussion.

Discussion
Page 27 - "optical mapping, may be needed to resolve the structure of these regions in finer detail." Again, perhaps longer reads that can span the repeats would help?

We have modified this sentence to include longer reads as a possibility.

Page 29 - "…suggesting the inversion may represent an evolved difference between the Metriaclima and Aulonocara lineages."   I am not sure what exactly is meant by "an evolved difference". Isn't any difference between the lineages evolved? I suggest rephrasing this…

We have reworded this.

Page 30 - "…suggesting that the rearrangement occurred in the Lake Malawi ancestor and has maintained reduced recombination in this region across all lineages."
The Malawi species used for the maps represent only one genus of mbuna and Aulonocara - a small subset of Lake Malawi lineages. So I suggest rephrasing this, instead of "Lake Malawi ancestor" something like "ancestor of the Lake Malawi species used in our crosses".

This sentence has been removed in our efforts to remove some of the repetitive regions from our manuscript as the reviewer suggests below.

Page 30 - "…has an XY sex determination locus on [76]"
I think there is a word missing at the end of the sentence.

We have added this.

Some examples of repetitiveness in the current text:

* *on page 10 we learn that LG3 is "the largest and most repetitive chromosome in O. niloticus [15], and is a sex chromosome in the closely related species, O. aureus", then on page 19 we read that "In the related species O. aureus, sex determination is controlled by a locus on LG3 [13,46]. ", then on page 20 we read "LG3, the largest chromosome in O. niloticus", then on page 23 that "LG3 is the largest tilapia chromosome", then on page 29 that LG3 is "where a sex determination locus is located in a sister species, O. aureus.", then on page 31 that this is the most complete assembly of "LG3, the largest chromosome in the O. niloticus karyotype (Figure 1). This chromosome carries a ZW sex locus in several species of Oreochromis [13,46]" on page 35 we read that "On LG3, a WZ sex determination region was previously identified [46] and characterized [13] in the congener species O. aureus" , then "the largest assembled chromosome in O. niloticus is LG3" on the same page. This is all rather tiresome.*
* *A different example: on page 44 is a description of which genetic map was used for anchoring particular LGs in the M. zebra assembly. But this is also shown in Table 3, and also described in even more detail in pages 14 and 15… and then reiterated again (without details) on page 27.*

We have removed this repetition except in one case where it is needed to make a separate point.
