# Supplementary material for: Chromosome-scale assemblies reveal the structural evolution of African cichlid genomes
Source: Gigascience. 2019 Apr 3;8(4):giz030. doi: 10.1093/gigascience/giz030 (PMC6447674; doi:10.1093/gigascience/giz030)

LG1

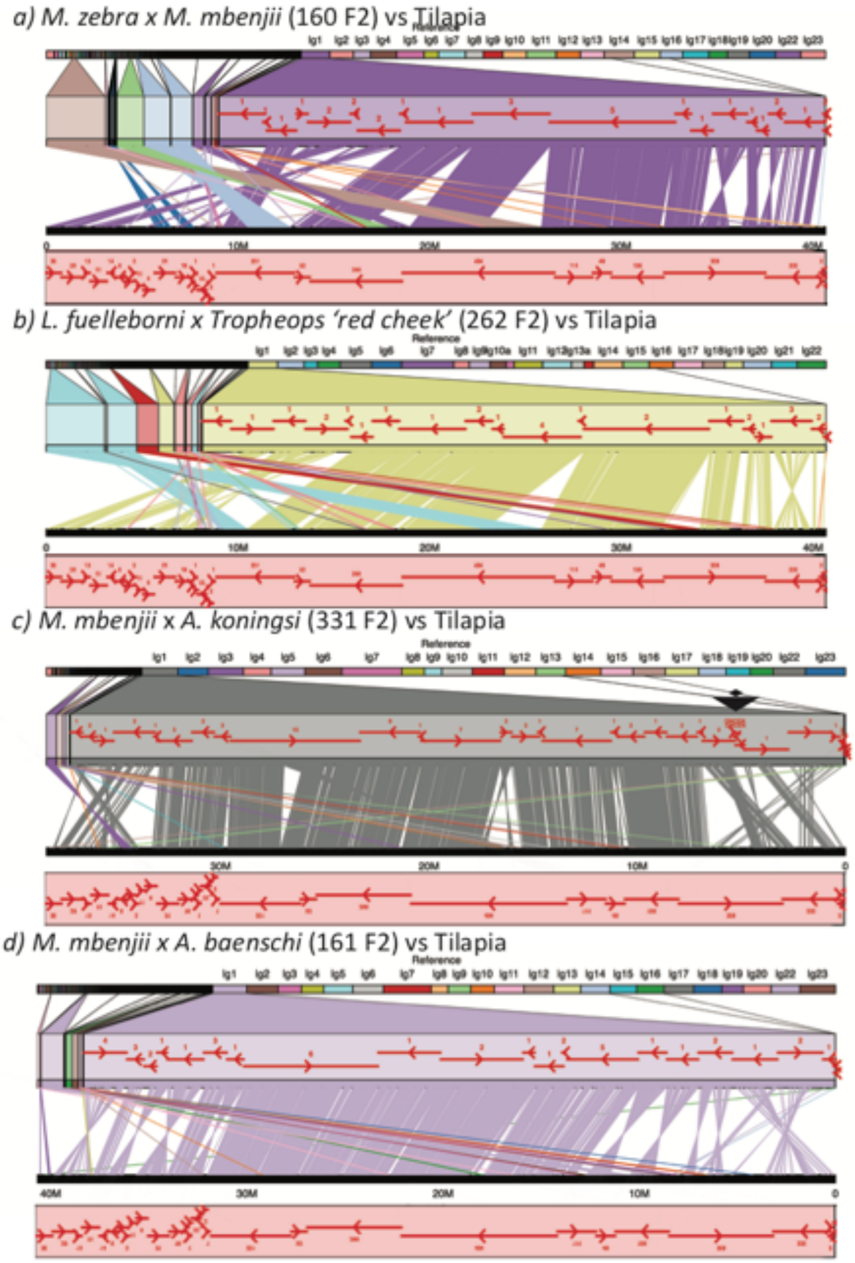
a) M. zebra x M. mbenjii (160 F2) vs Tilapia
b) L. fuelleborni x Tropheops 'red cheek' (262 F2) vs Tilapia
c) M. mbenjii x A. koningsi (331 F2) vs Tilapia
d) M. mbenjii x A. baenschi (161 F2) vs Tilapia
Reference
10M
20M
30M
40M

LG2

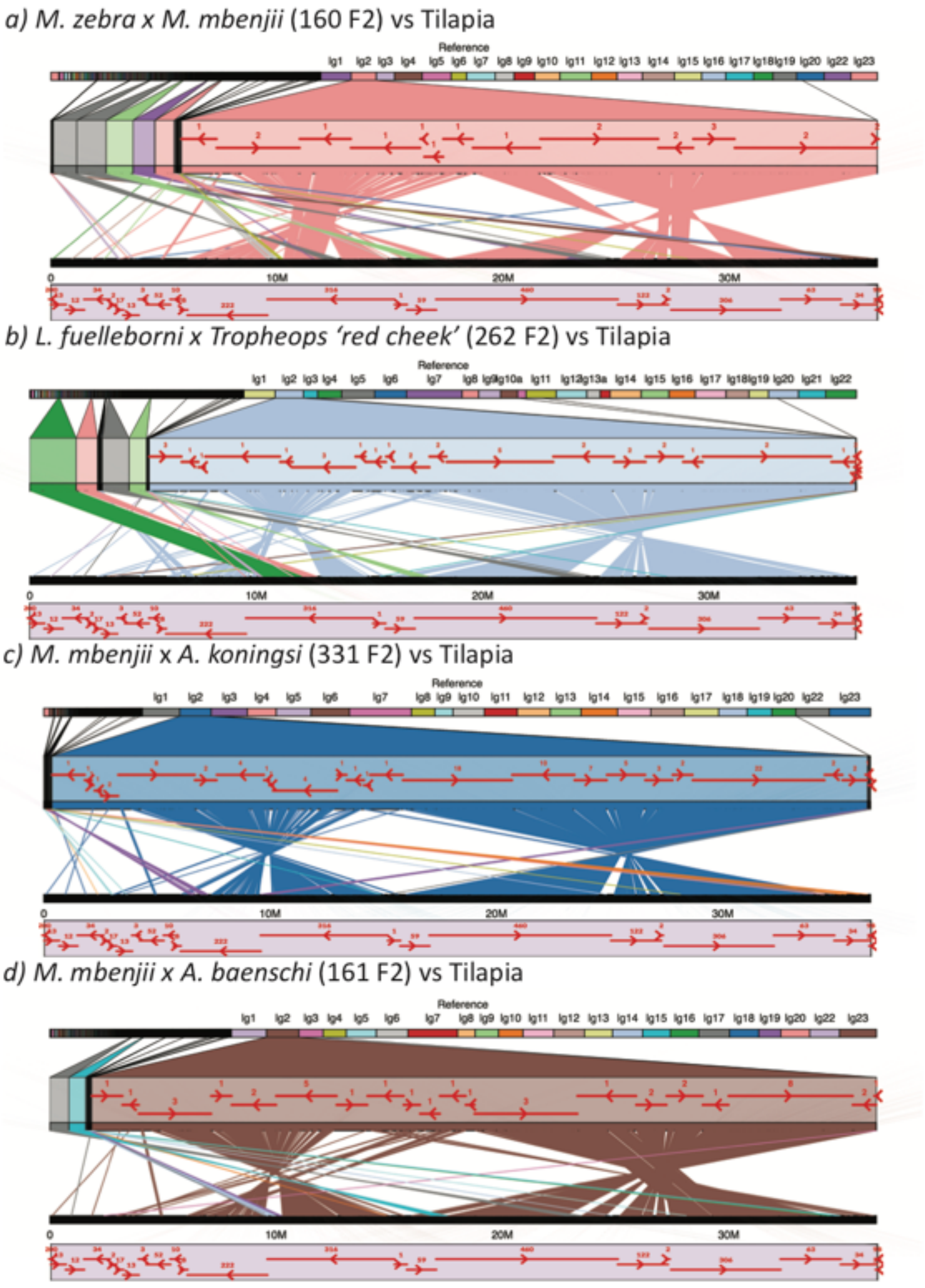
a) M. zebra x M. mbenjii (160 F2) vs Tilapia
Reference
0
10M
20M
30M
b) L. fuelleborni x Tropheops 'red cheek' (262 F2) vs Tilapia
Reference
0
10M
20M
30M
c) M. mbenjii x A. koningsi (331 F2) vs Tilapia
Reference
0
10M
20M
30M
d) M. mbenjii x A. baenschi (161 F2) vs Tilapia
Reference
0
10M
20M
30M

LG3

a) M. zebra x M. mbenjii (160 F2) vs Tilapia

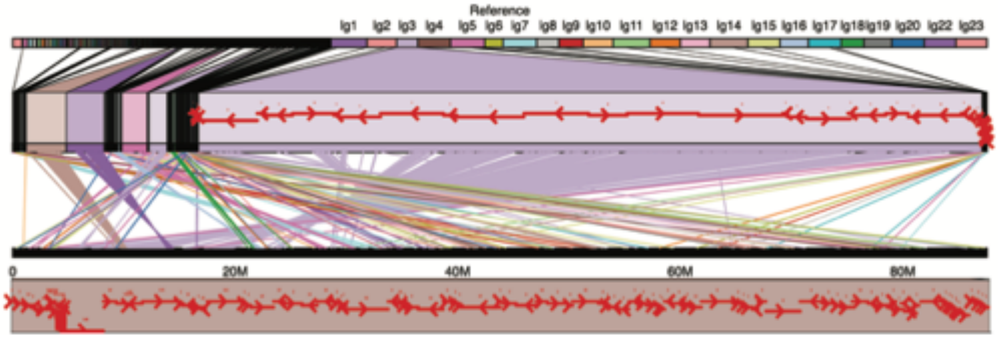

b) L. fuelleborni x Tropheops 'red cheek' (262 F2) vs Tilapia

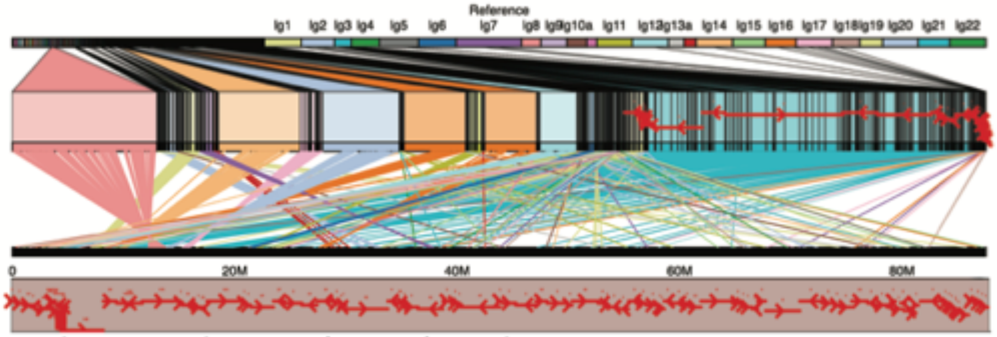

c) M. mbenjii x A. koningsi (331 F2) vs Tilapia

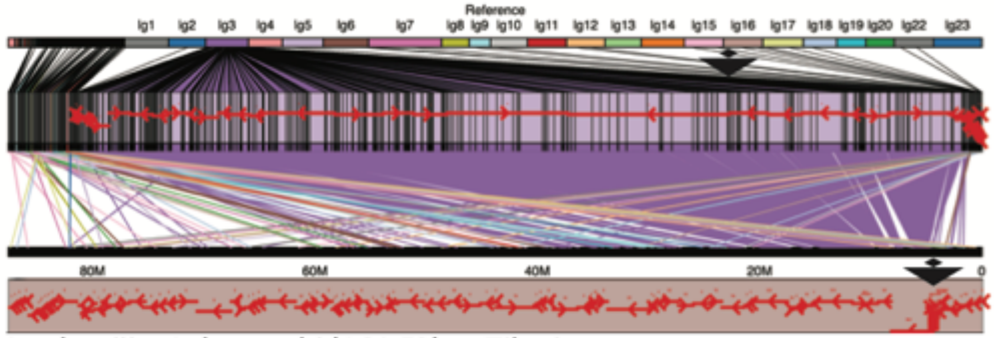

d) M. mbenjii x A. baenschi (161 F2) vs Tilapia

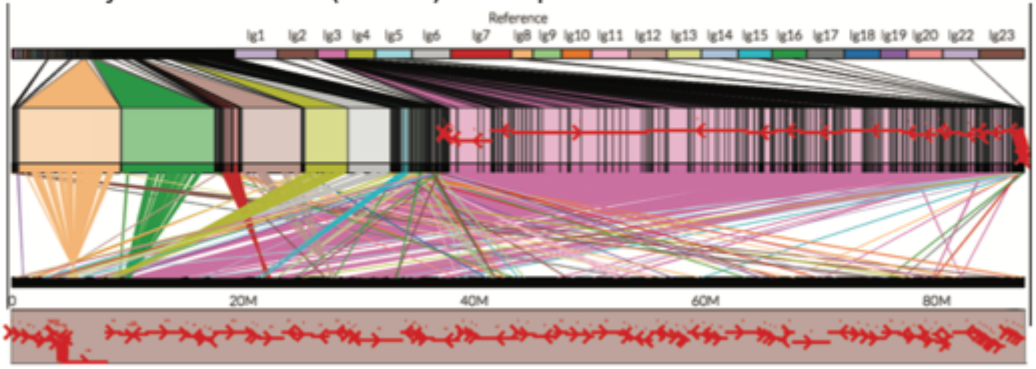

LG4

a) *M. zebra x M. mbenjii* (160 F2) vs Tilapia

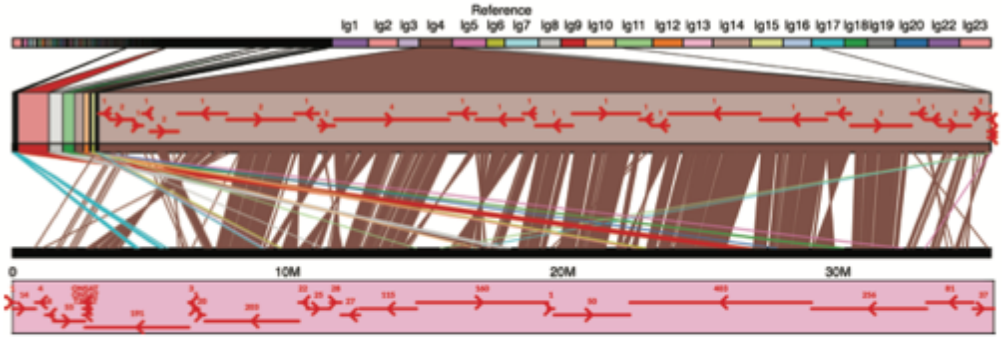

b) *L. fuelleborni x Tropheops 'red cheek'* (262 F2) vs Tilapia

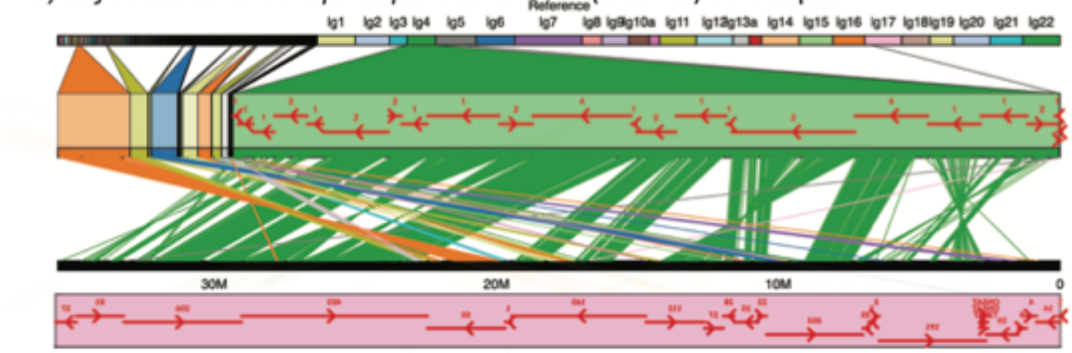

c) *M. mbenjii* x *A. koningsi* (331 F2) vs Tilapia

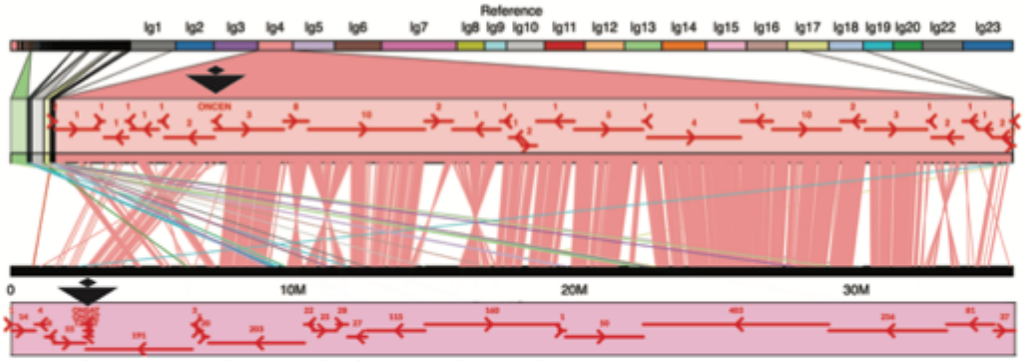

d) *M. mbenjii* x *A. baenschi* (161 F2) vs Tilapia

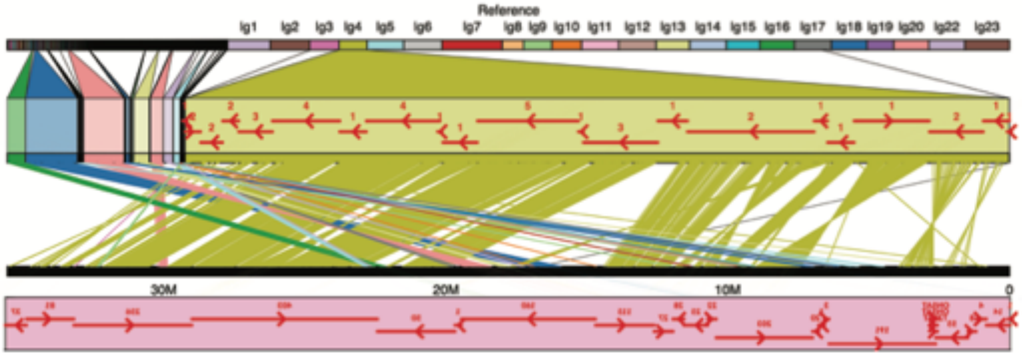

LG5

a) M. zebra x M. mbenjii (160 F2) vs Tilapia

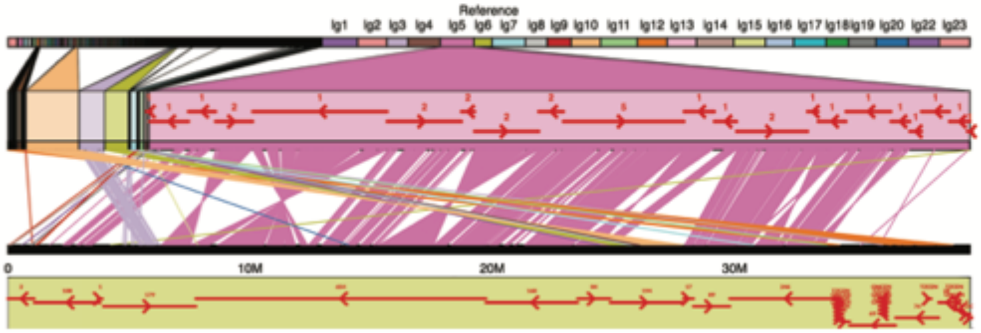

b) L. fuelleborni x Tropheops 'red cheek' (262 F2) vs Tilapia

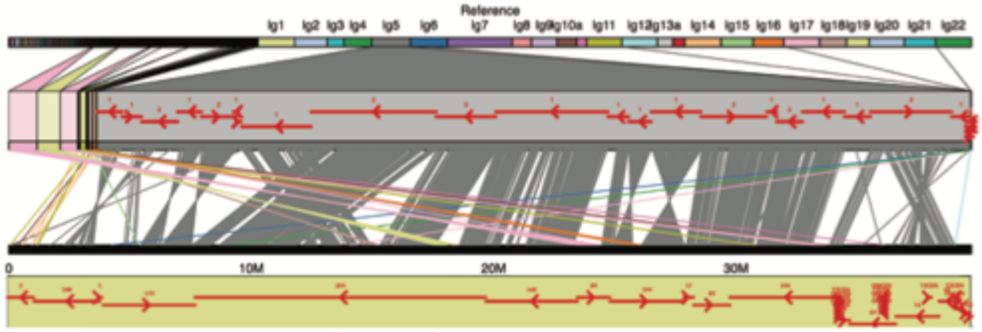

c) M. mbenjii x A. koningsi (331 F2) vs Tilapia

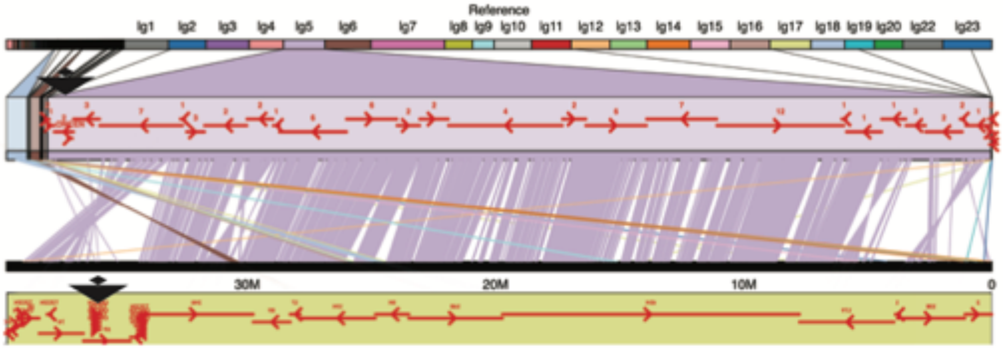

d) M. mbenjii x A. baenschi (161 F2) vs Tilapia

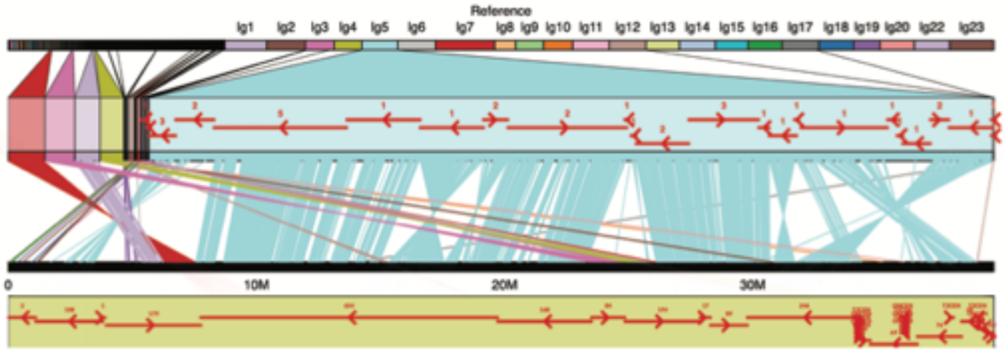

LG6

a) M. zebra x M. mbenjii (160 F2) vs Tilapia

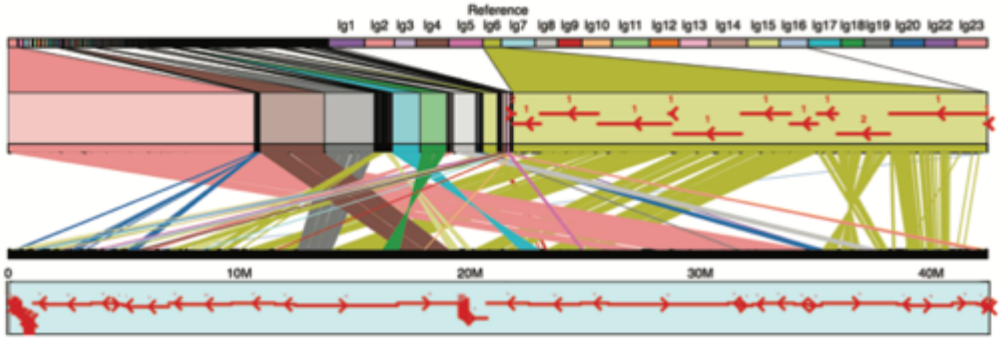

b) L. fuelleborni x Tropheops 'red cheek' (262 F2) vs Tilapia

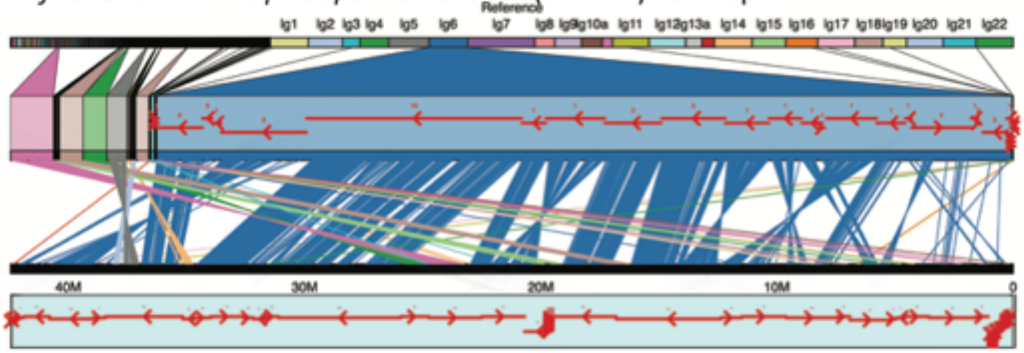

c) M. mbenjii x A. koningsi (331 F2) vs Tilapia

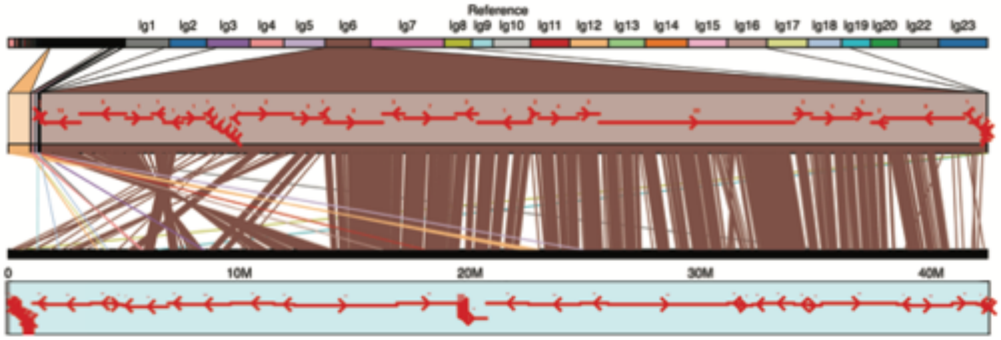

d) M. mbenjii x A. baenschi (161 F2) vs Tilapia

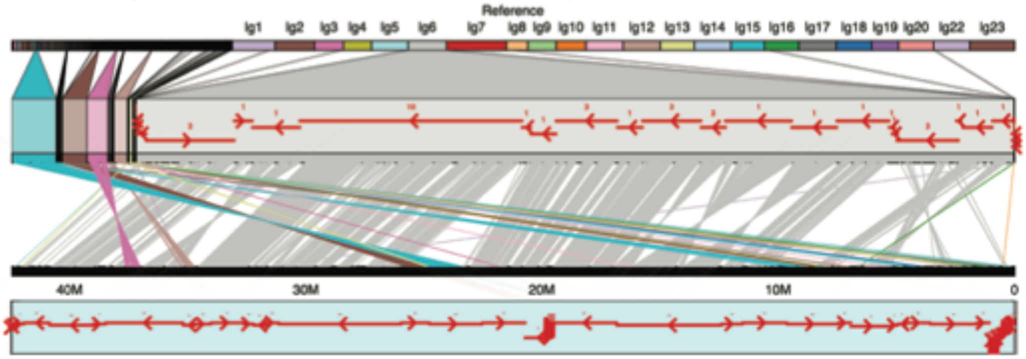

LG7

a) *M. zebra* x *M. mbenjii* (160 F2) vs Tilapia

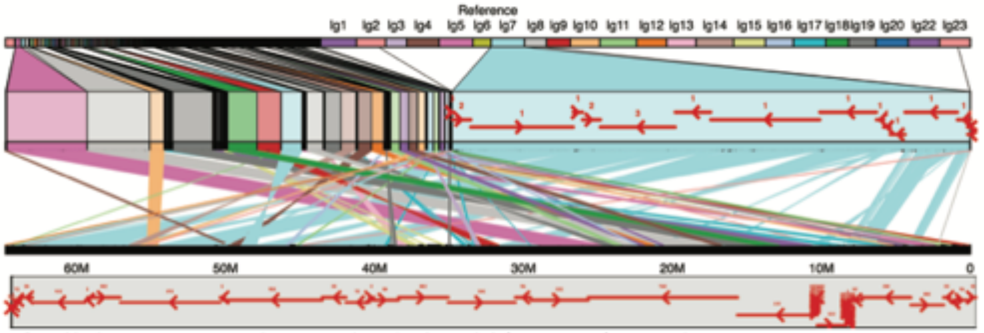

b) *L. fuelleborni* x *Tropheops 'red cheek'* (262 F2) vs Tilapia

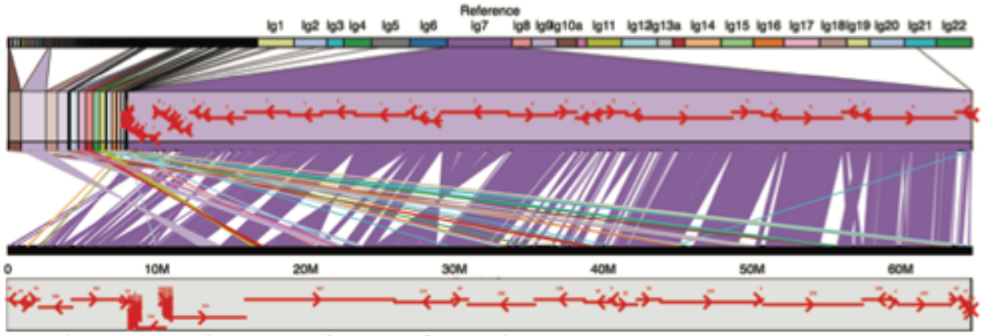

c) *M. mbenjii* x *A. koningsi* (331 F2) vs Tilapia

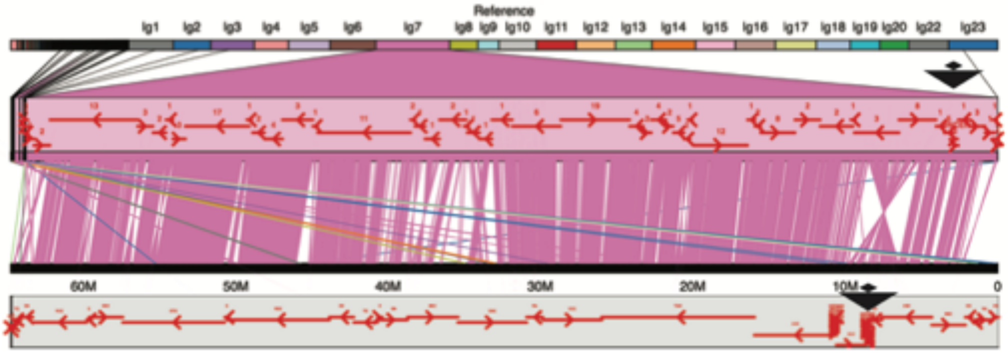

d) *M. mbenjii* x *A. baenschi* (161 F2) vs Tilapia

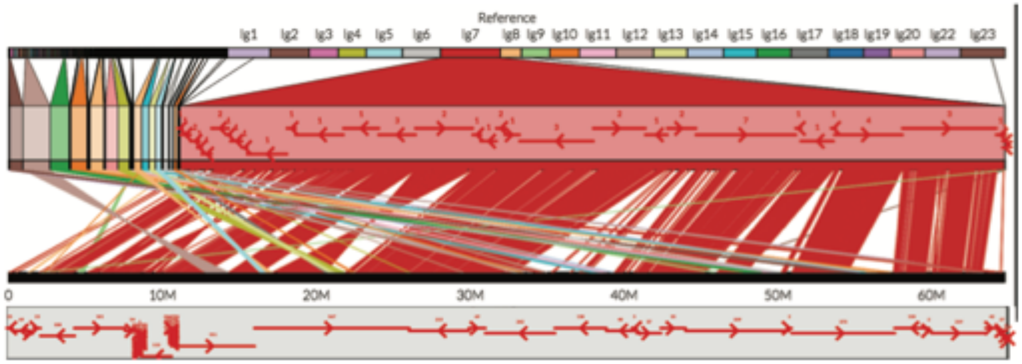

LG8

a) M. zebra x M. mbenjii (160 F2) vs Tilapia

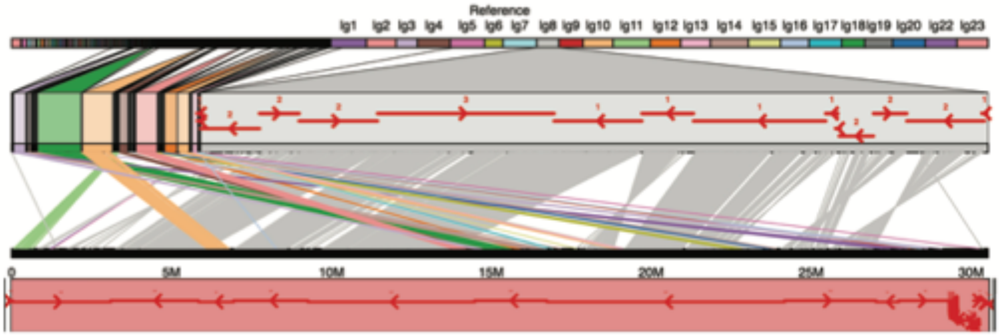

b) L. fuelleborni x Tropheops 'red cheek' (262 F2) vs Tilapia

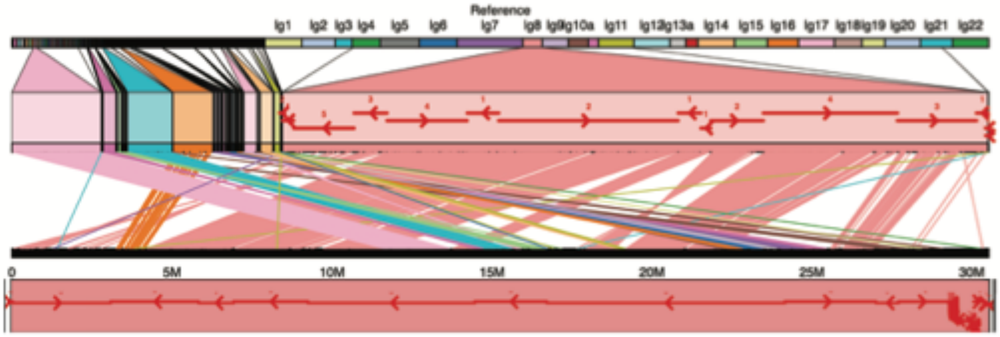

c) M. mbenjii x *A. koningsi* (331 F2) vs Tilapia

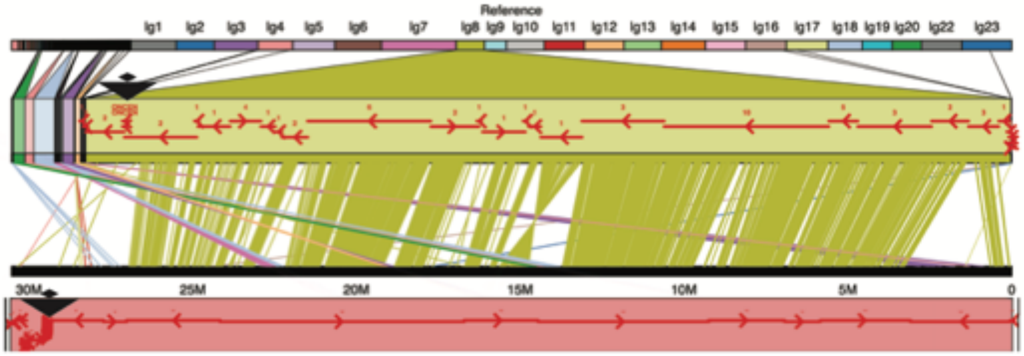

d) M. mbenjii x A. baenschi (161 F2) vs Tilapia

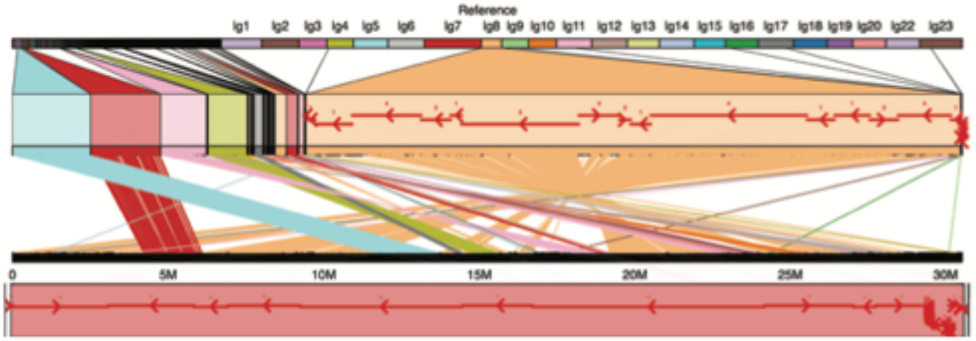

LG9

a) M. zebra x M. mbenjii (160 F2) vs Tilapia

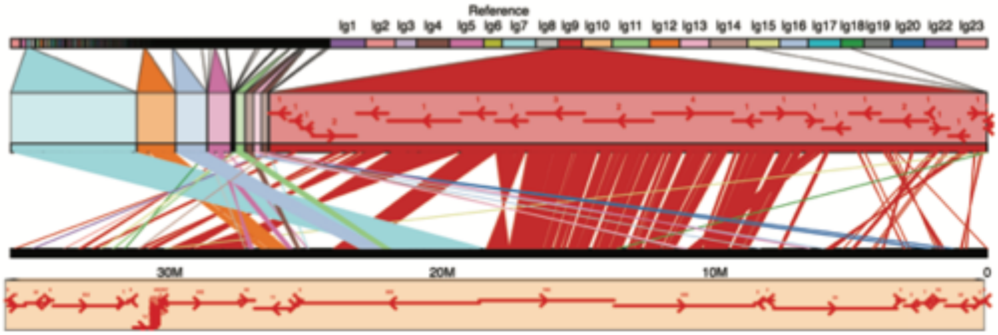

b) L. fuelleborni x Tropheops 'red cheek' (262 F2) vs Tilapia

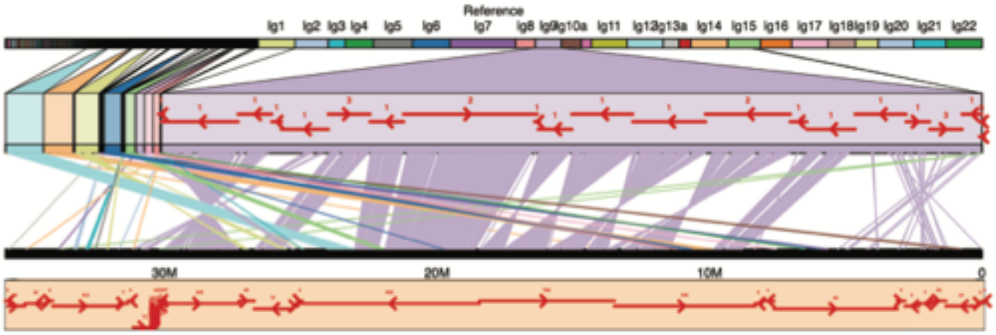

c) M. mbenjii x *A. koningsi* (331 F2) vs Tilapia

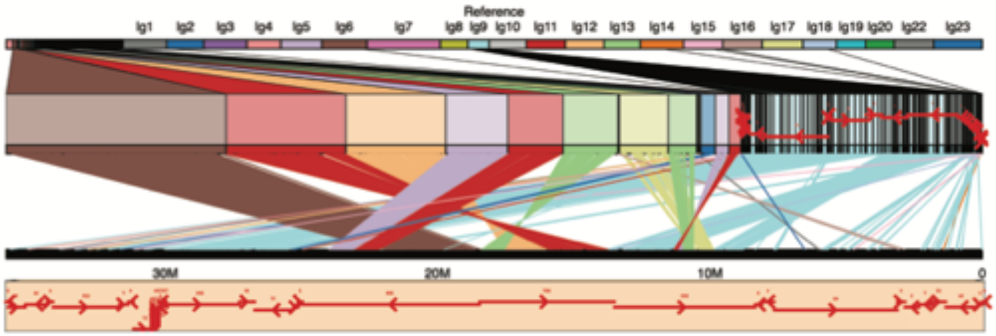

d) M. mbenjii x A. baenschi (161 F2) vs Tilapia

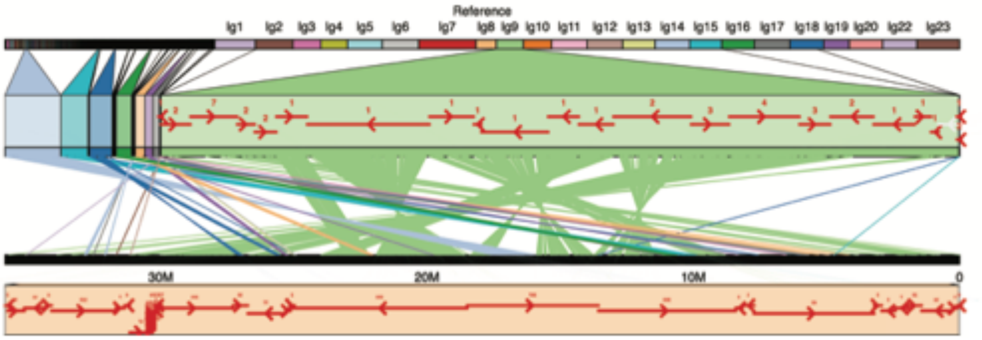

LG10

a) M. zebra x M. mbenjii (160 F2) vs Tilapia

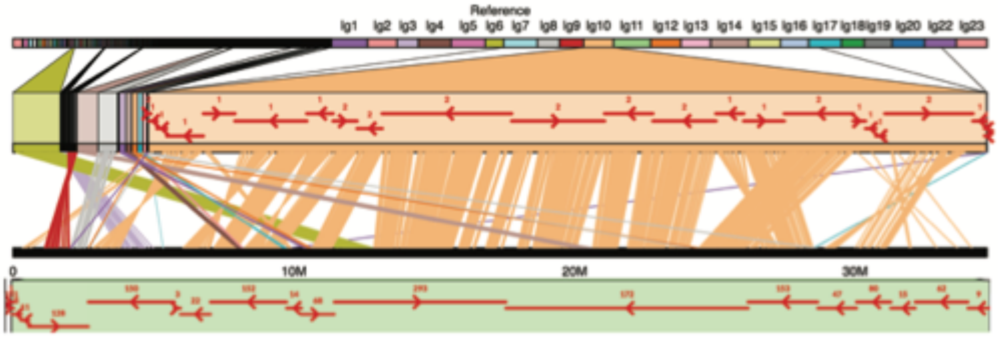

b) L. fuelleborni x Tropheops 'red cheek' (262 F2) vs Tilapia

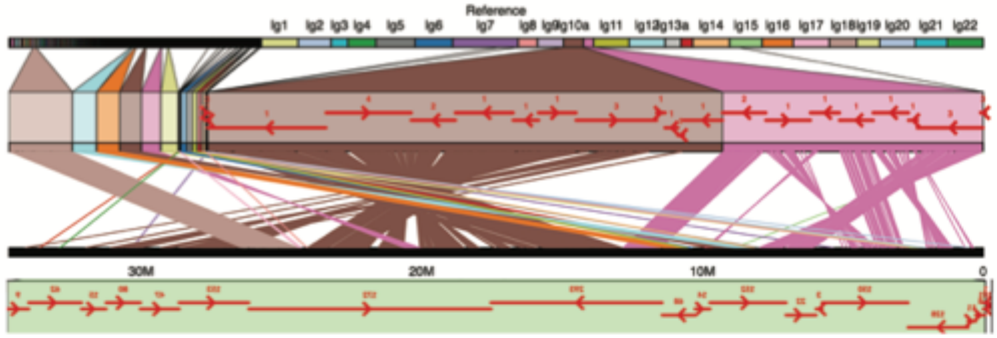

c) M. mbenjii x *A. koningsi* (331 F2) vs Tilapia

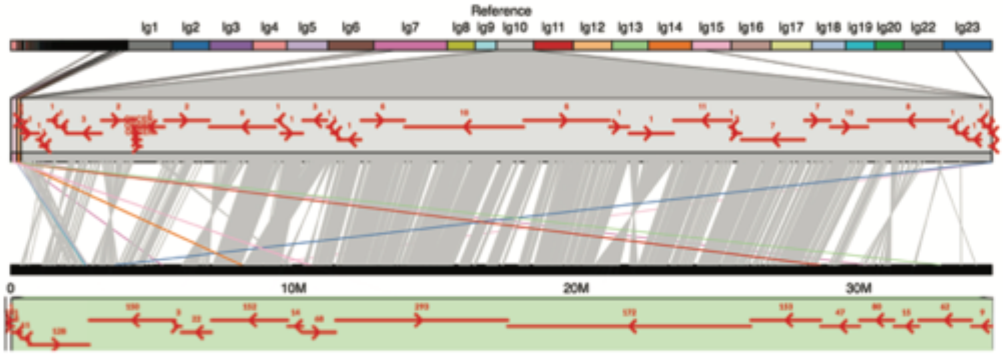

d) M. mbenjii x A. baenschi (161 F2) vs Tilapia

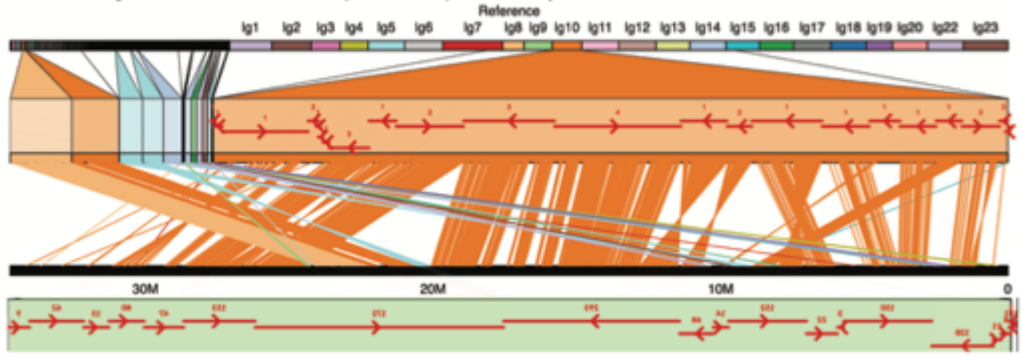

LG11

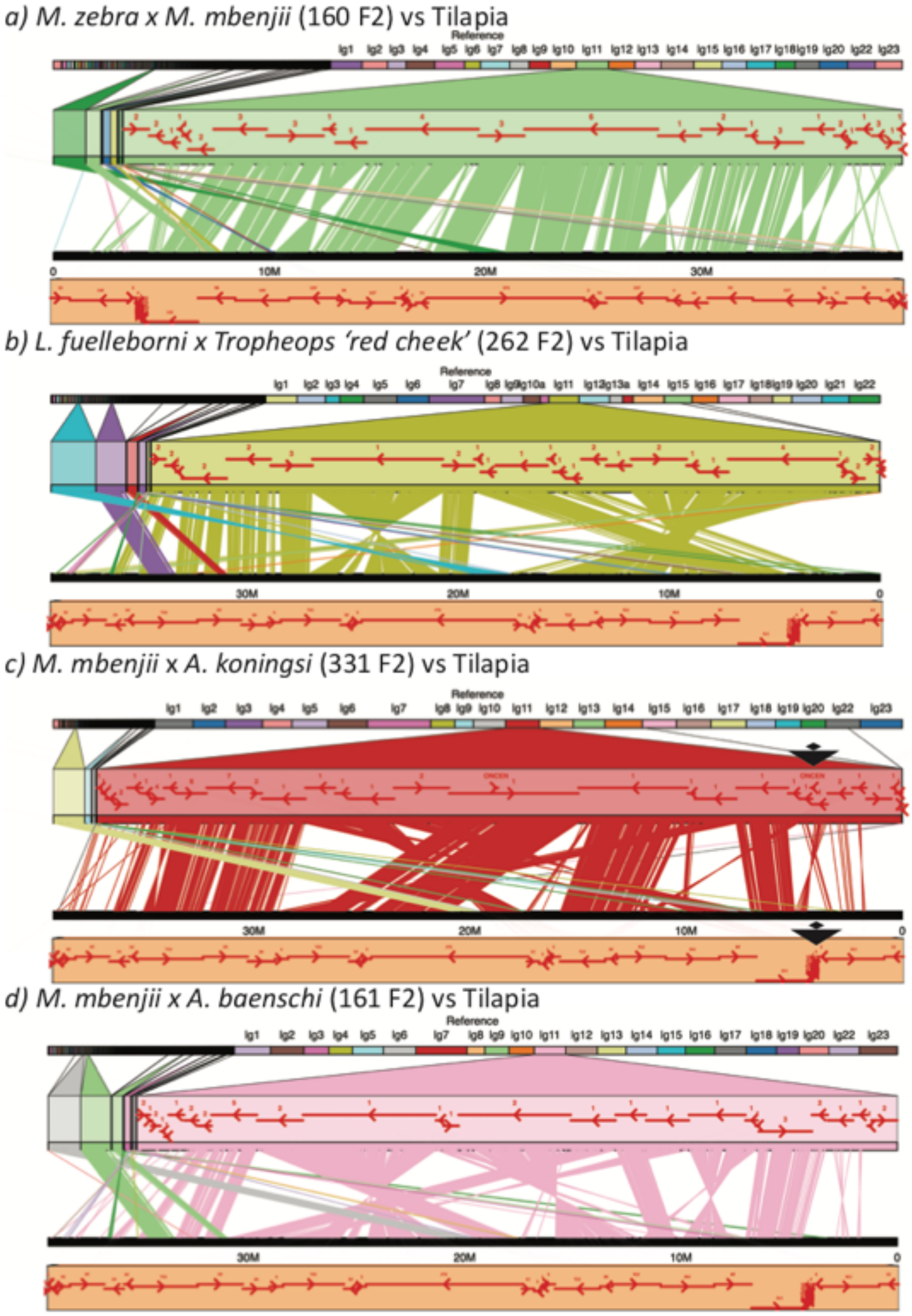

a) M. zebra x M. mbenjii (160 F2) vs Tilapia
Reference
0
10M
20M
30M
b) L. fuelleborni x Tropheops 'red cheek' (262 F2) vs Tilapia
Reference
30M
20M
10M
0
c) M. mbenjii x A. koningsi (331 F2) vs Tilapia
Reference
30M
20M
10M
0
d) M. mbenjii x A. baenschi (161 F2) vs Tilapia
Reference
30M
20M
10M
0

LG12

a) M. zebra x M. mbenjii (160 F2) vs Tilapia

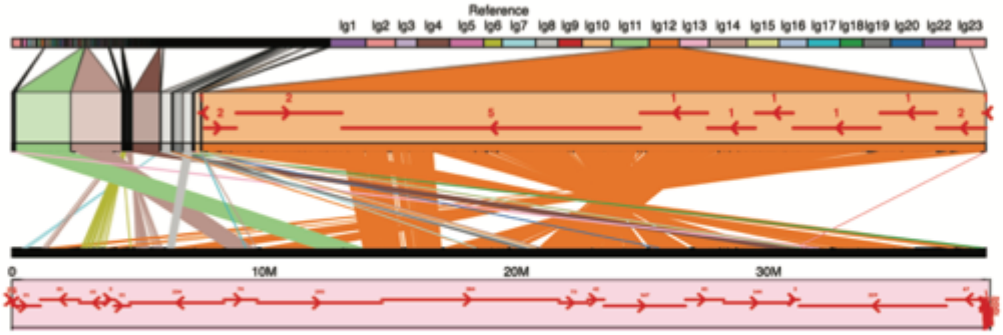

b) L. fuelleborni x Tropheops 'red cheek' (262 F2) vs Tilapia

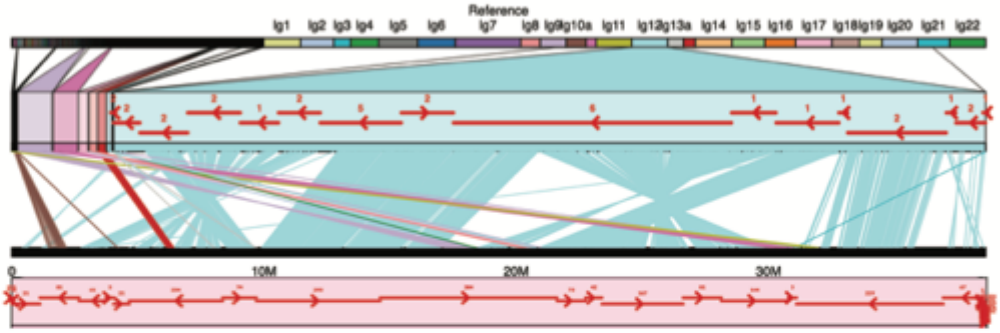

c) M. mbenjii x A. koningsi (331 F2) vs Tilapia

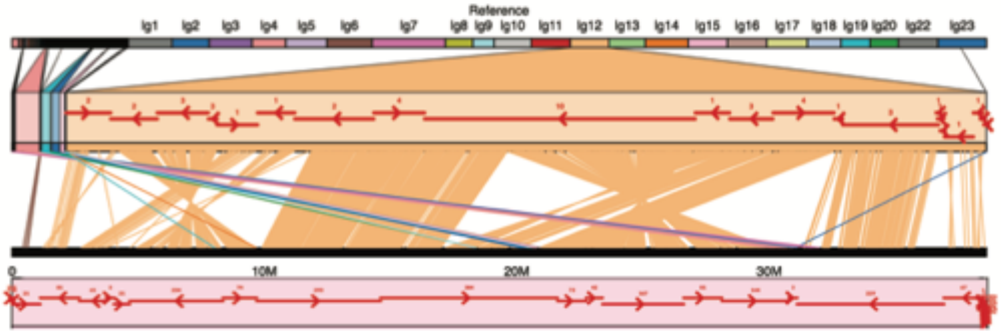

d) M. mbenjii x A. baenschi (161 F2) vs Tilapia

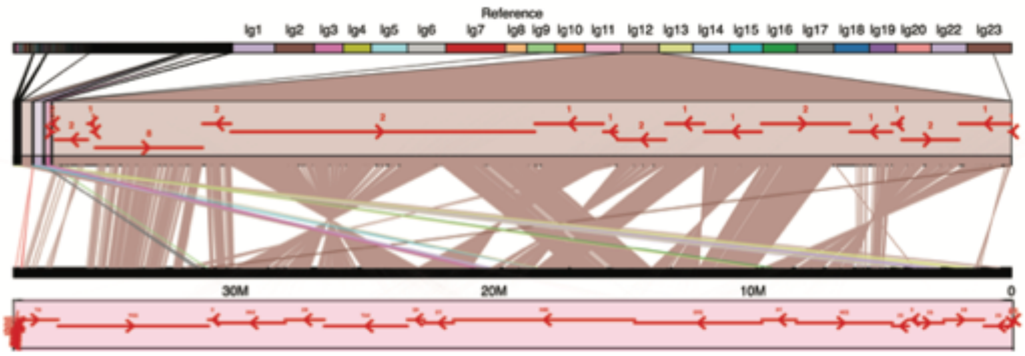

LG13

a) *M. zebra x M. mbenjii* (160 F2) vs Tilapia

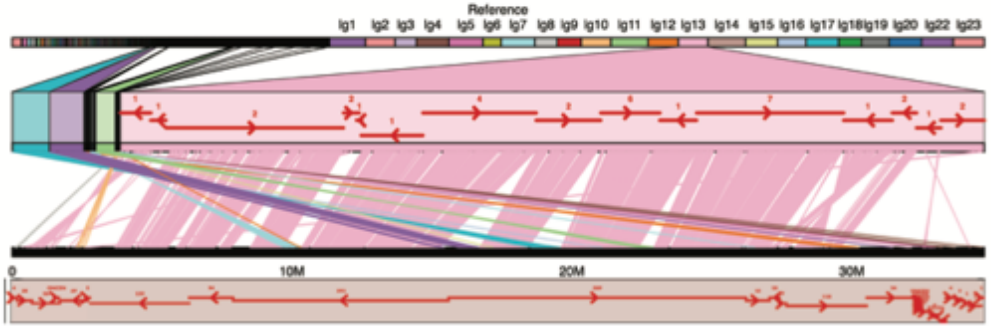

b) *L. fuelleborni x Tropheops 'red cheek'* (262 F2) vs Tilapia

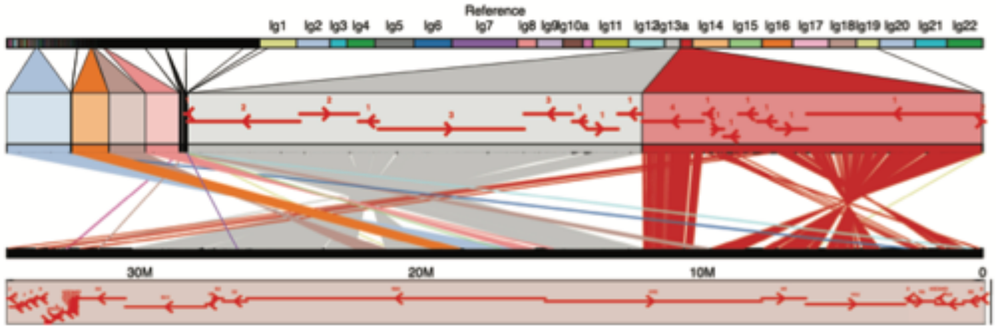

c) *M. mbenjii x A. koningsi* (331 F2) vs Tilapia

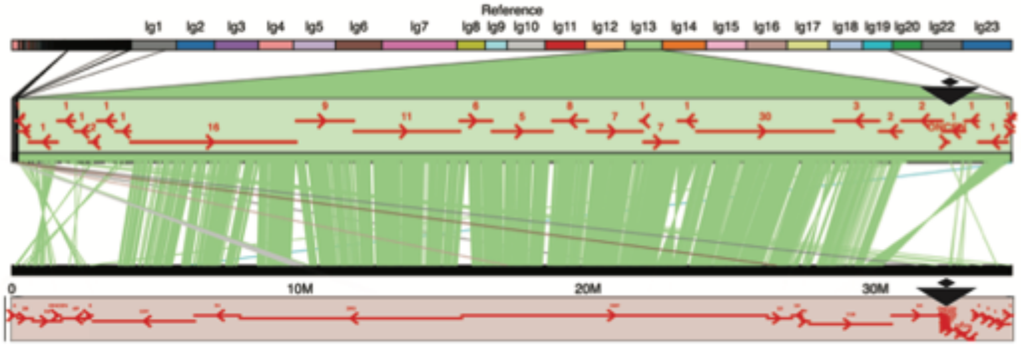

d) *M. mbenjii x A. baenschi* (161 F2) vs Tilapia

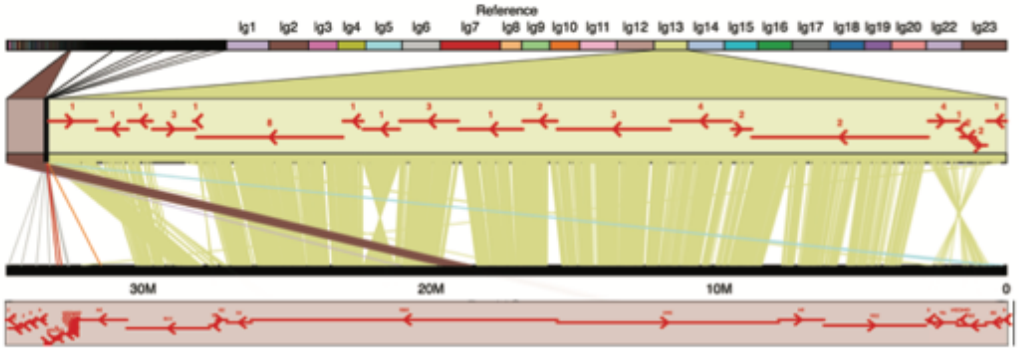

LG14

a) *M. zebra* x *M. mbenjii* (160 F2) vs Tilapia

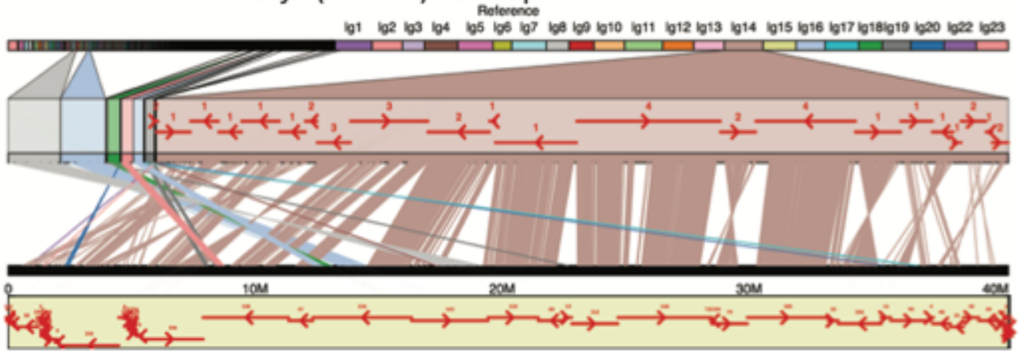

b) *L. fuelleborni* x *Tropheops 'red cheek'* (262 F2) vs Tilapia

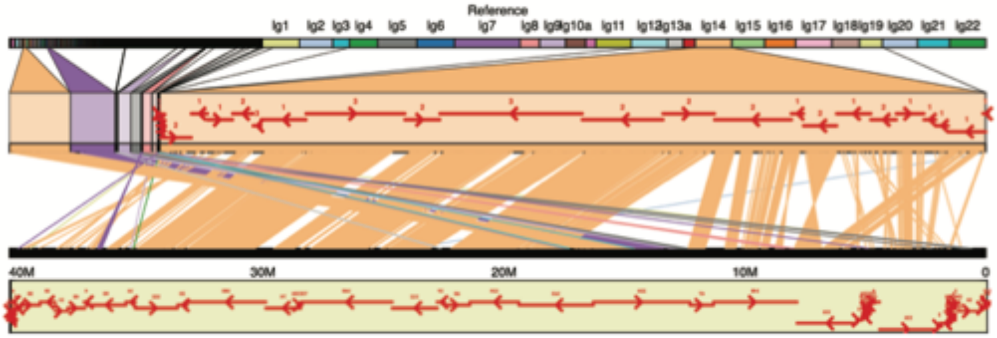

c) *M. mbenjii* x *A. koningsi* (331 F2) vs Tilapia

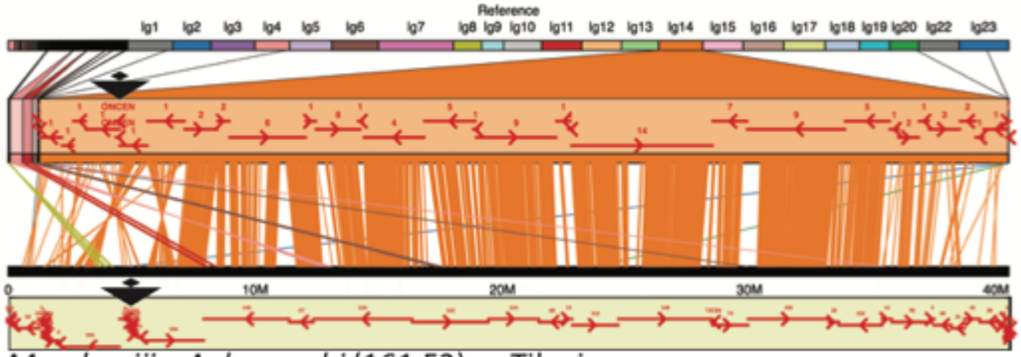

d) *M. mbenjii* x *A. baenschi* (161 F2) vs Tilapia

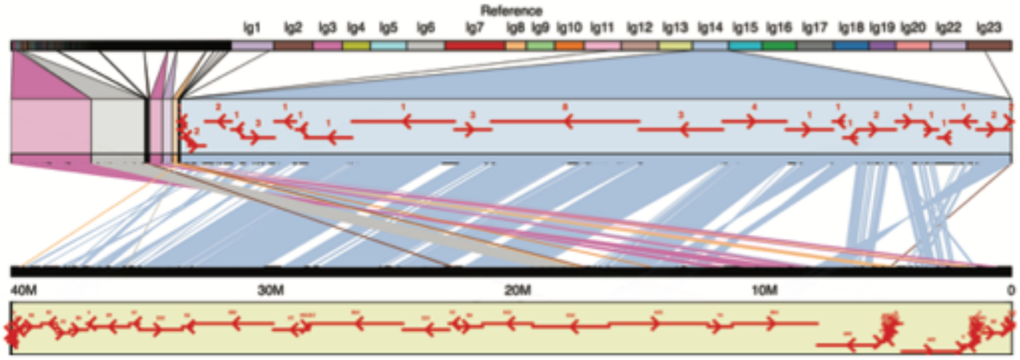

LG15

a) *M. zebra* x *M. mbenjii* (160 F2) vs Tilapia

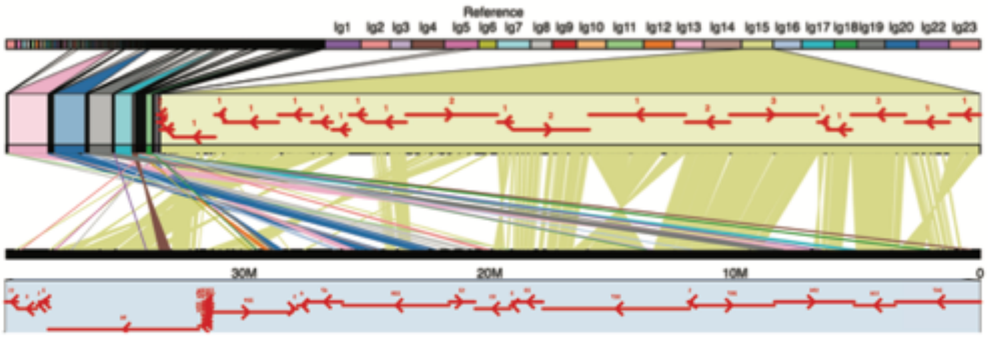

b) *L. fuelleborni* x *Tropheops 'red cheek'* (262 F2) vs Tilapia

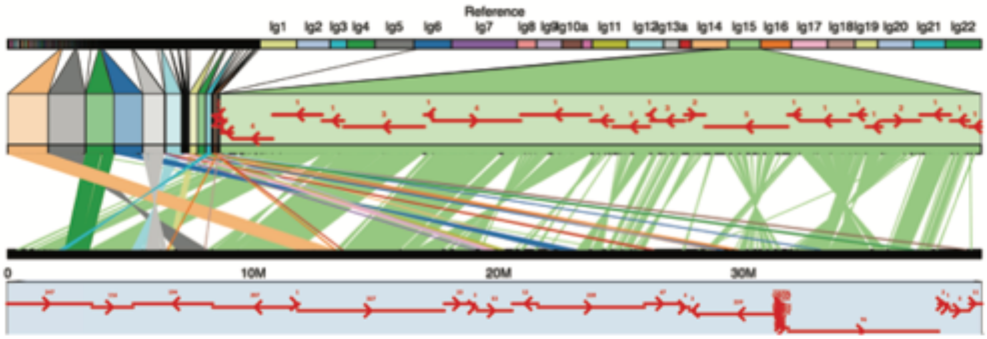

c) *M. mbenjii* x *A. koningsi* (331 F2) vs Tilapia

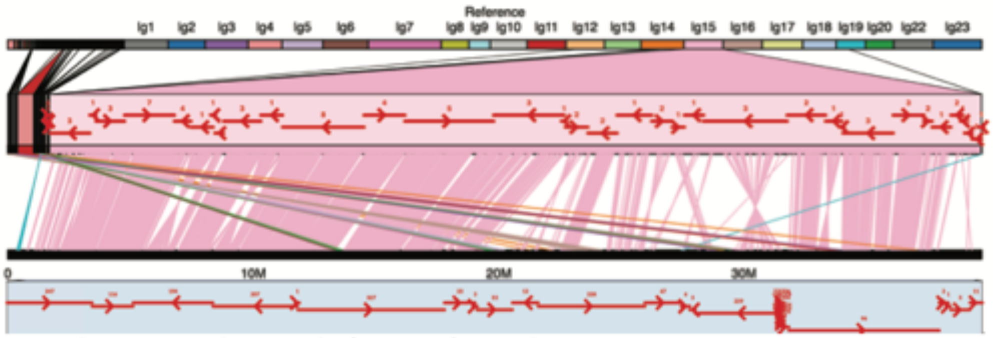

d) *M. mbenjii* x *A. baenschi* (161 F2) vs Tilapia

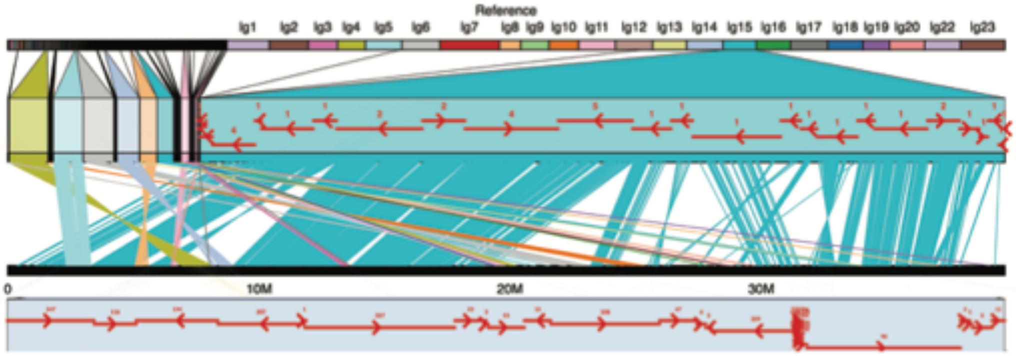

LG16

a) M. zebra x M. mbenjii (160 F2) vs Tilapia

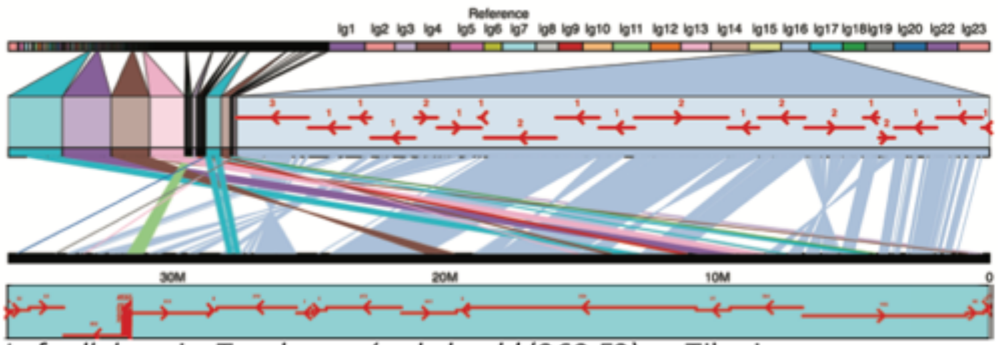

b) L. fuelleborni x Tropheops 'red cheek' (262 F2) vs Tilapia

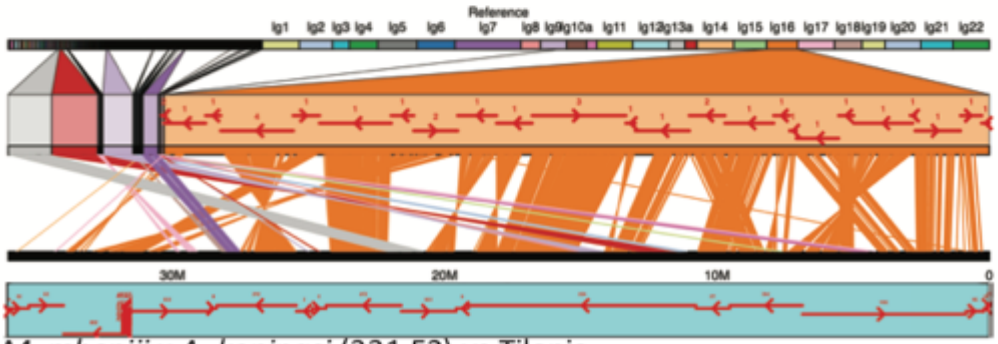

c) M. mbenjii x *A. koningsi* (331 F2) vs Tilapia

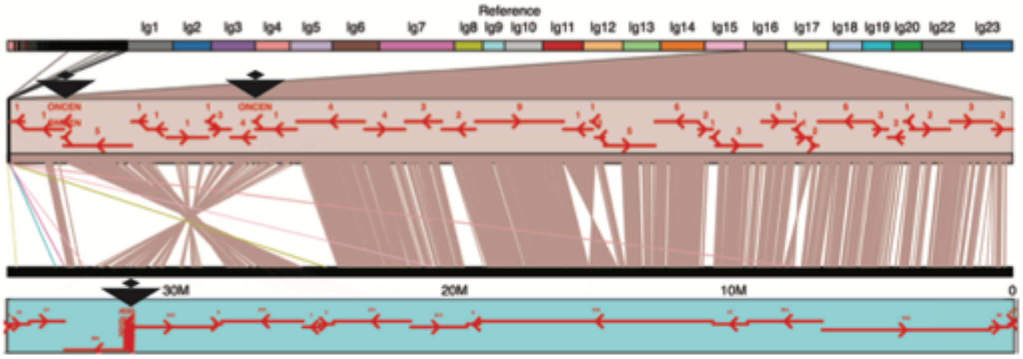

d) M. mbenjii x *A. baenschi* (161 F2) vs Tilapia

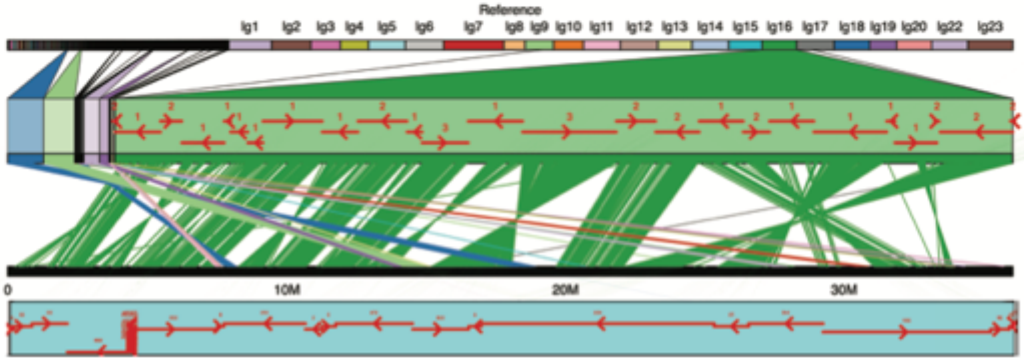

LG17

a) *M. zebra x M. mbenjii* (160 F2) vs Tilapia

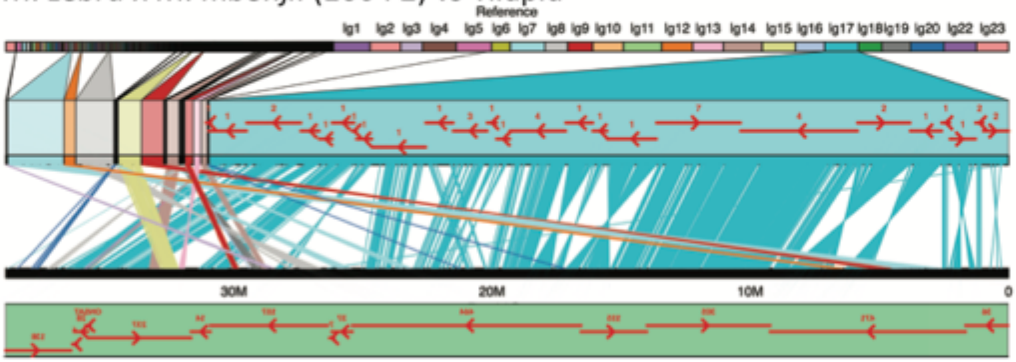

b) *L. fuelleborni x Tropheops 'red cheek'* (262 F2) vs Tilapia

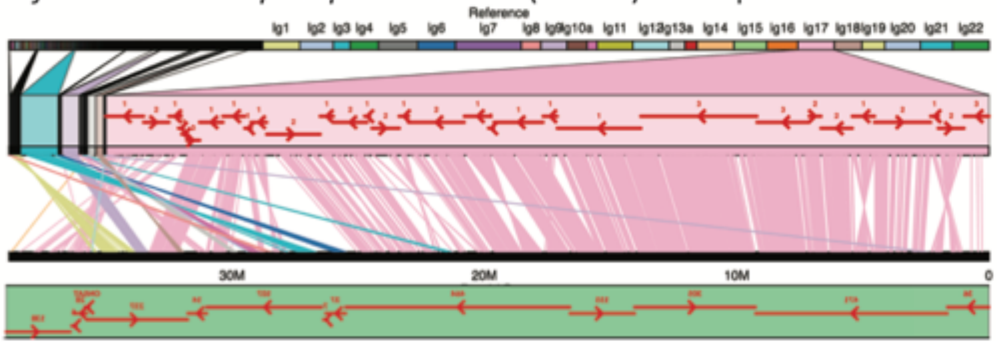

c) *M. mbenjii* x *A. koningsi* (331 F2) vs Tilapia

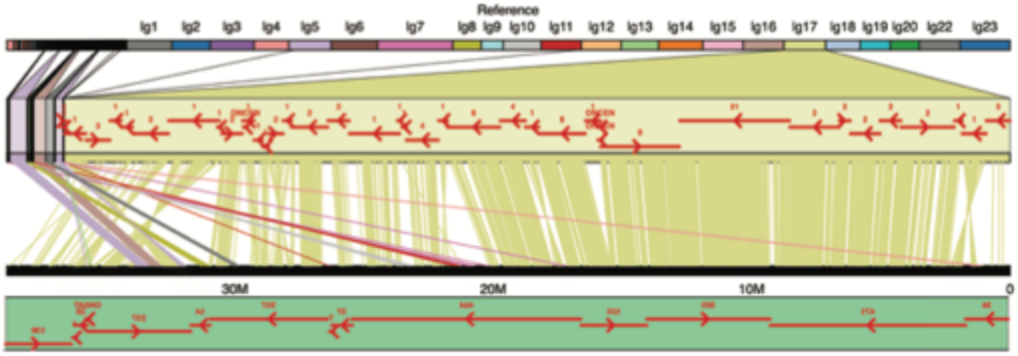

d) *M. mbenjii x A. baenschi* (161 F2) vs Tilapia

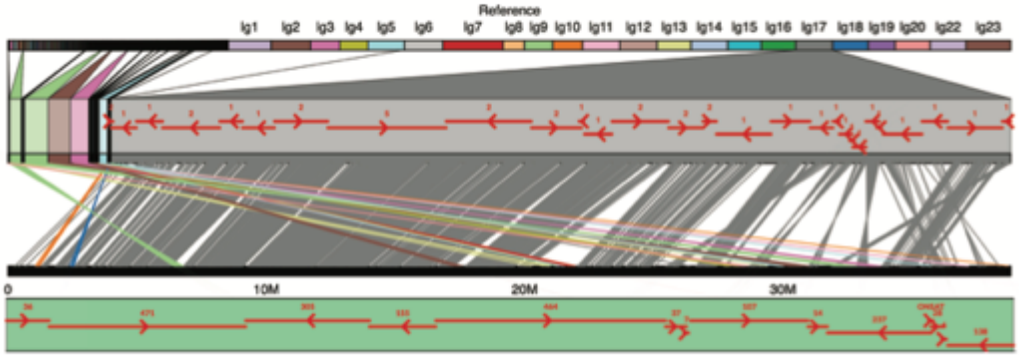

LG18

a) M. zebra x M. mbenjii (160 F2) vs Tilapia

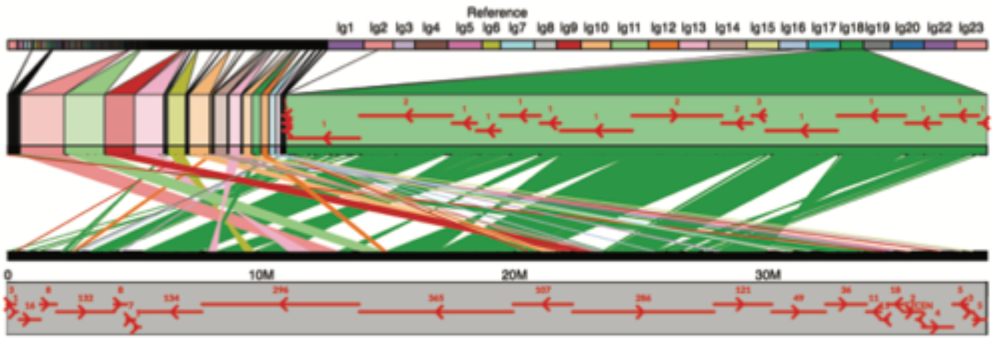

b) L. fuelleborni x Tropheops 'red cheek' (262 F2) vs Tilapia

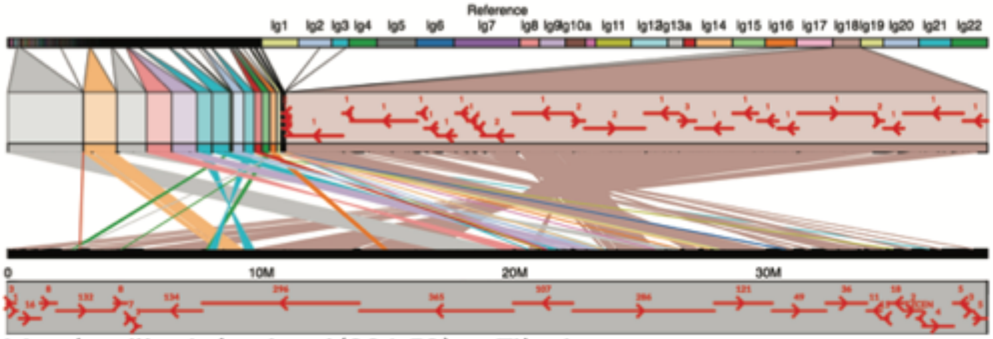

c) M. mbenjii x *A. koningsi* (331 F2) vs Tilapia

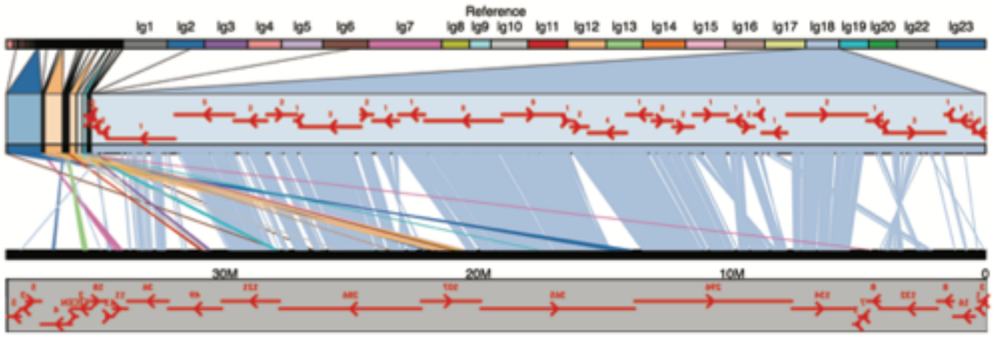

d) M. mbenjii x *A. baenschi* (161 F2) vs Tilapia

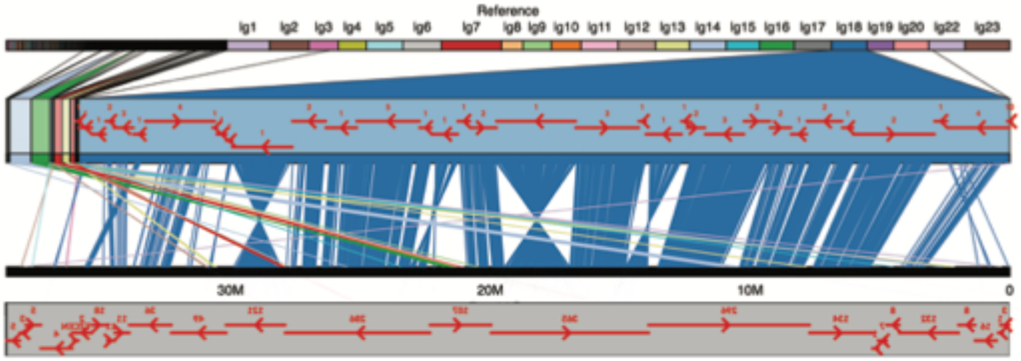

LG19

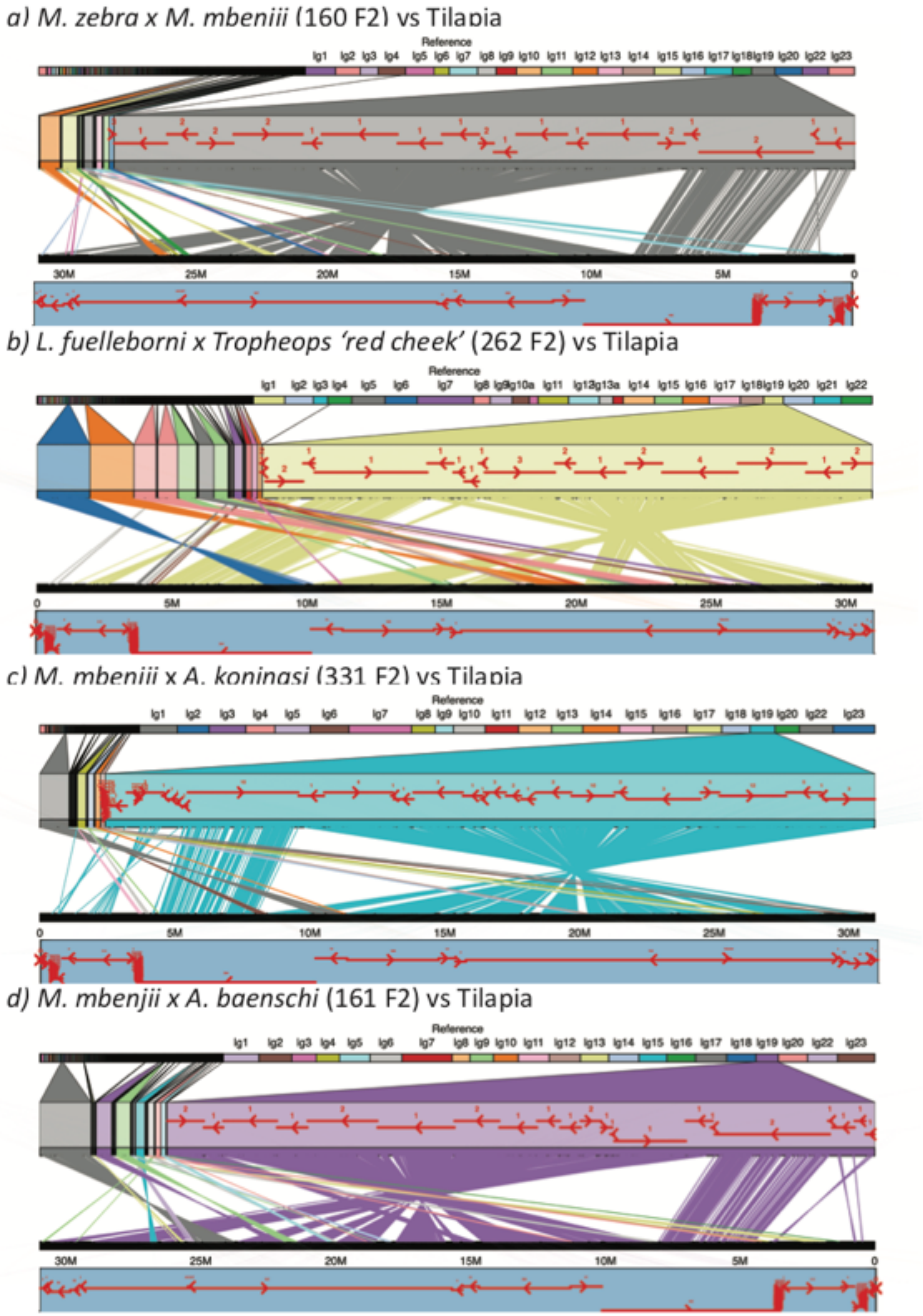
a) M. zebra x M. mbenjii (160 F2) vs Tilapia
Reference
30M
25M
20M
15M
10M
5M
0
b) L. fuelleborni x Tropheops 'red cheek' (262 F2) vs Tilapia
Reference
0
5M
10M
15M
20M
25M
30M
c) M. mbenjii x A. koningsi (331 F2) vs Tilapia
Reference
0
5M
10M
15M
20M
25M
30M
d) M. mbenjii x A. baenschi (161 F2) vs Tilapia
Reference
30M
25M
20M
15M
10M
5M
0

LG20

a) M. zebra x M. mbenjii (160 F2) vs Tilapia

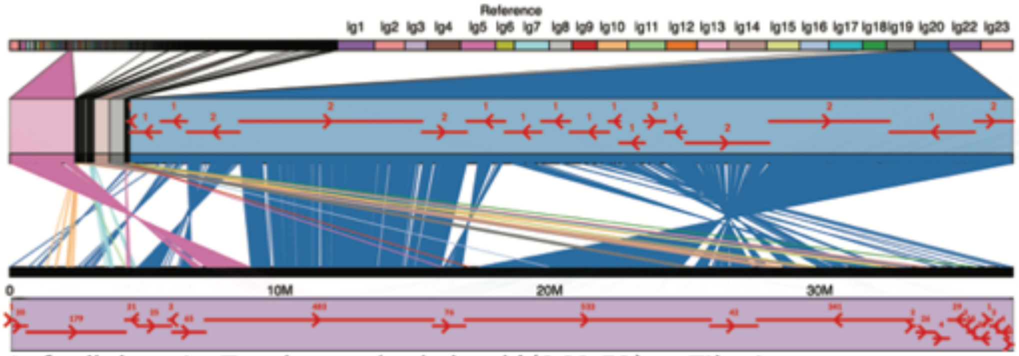

b) L. fuelleborni x Tropheops 'red cheek' (262 F2) vs Tilapia

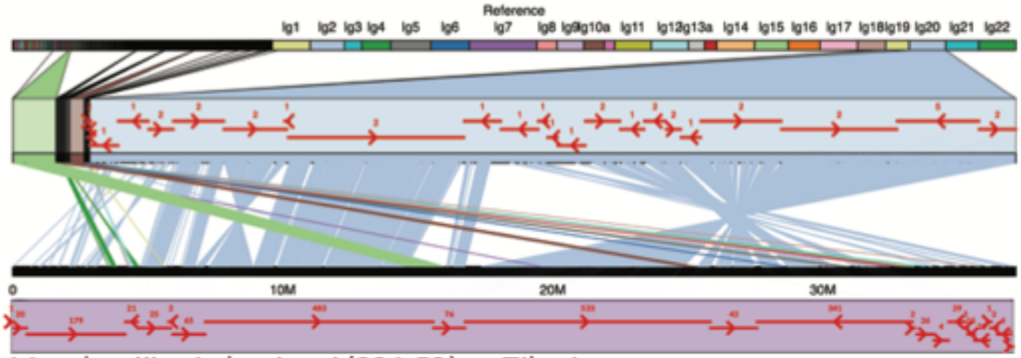

c) M. mbenjii x A. koningsi (331 F2) vs Tilapia

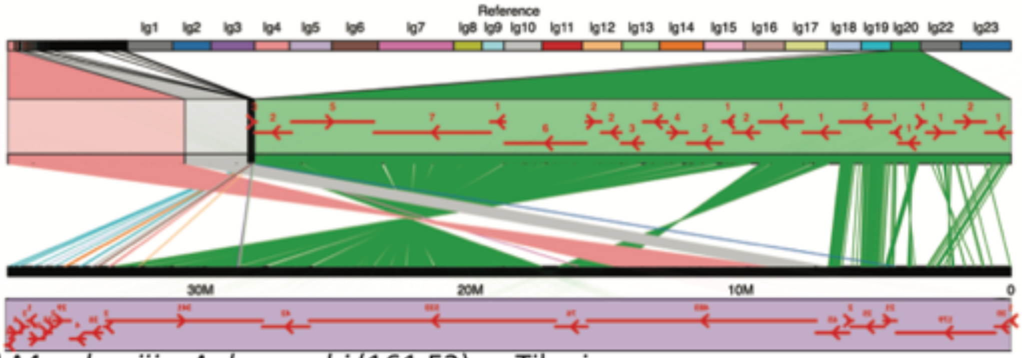

d) M. mbenjii x A. baenschi (161 F2) vs Tilapia

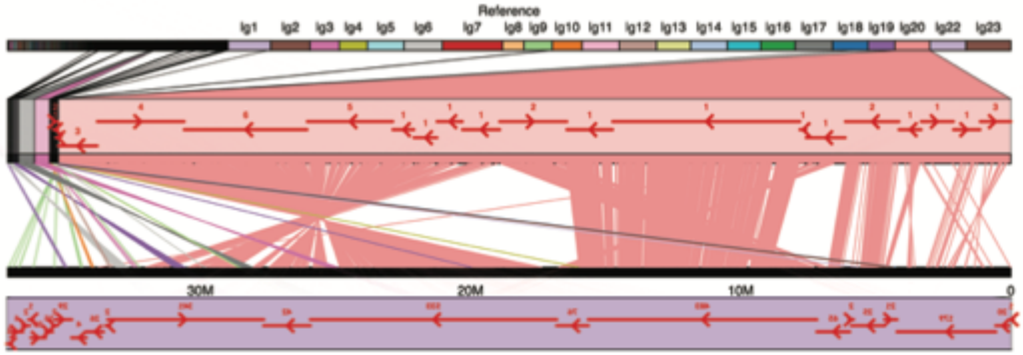

LG22

a) M. zebra x M. mbenjii (160 F2) vs Tilapia

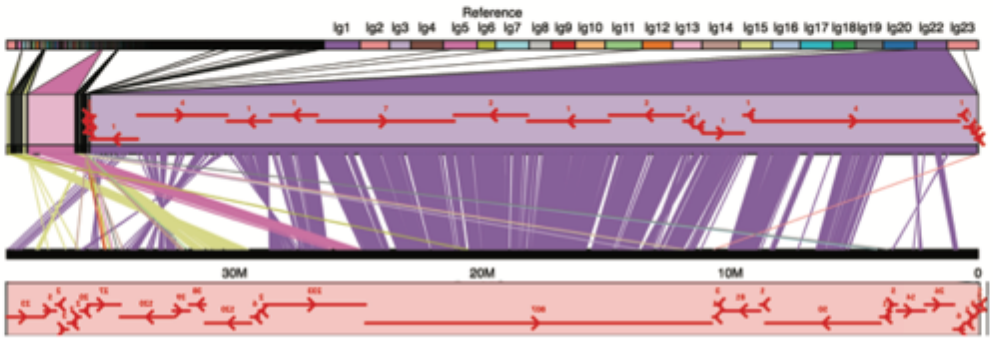

b) L. fuelleborni x Tropheops 'red cheek' (262 F2) vs Tilapia

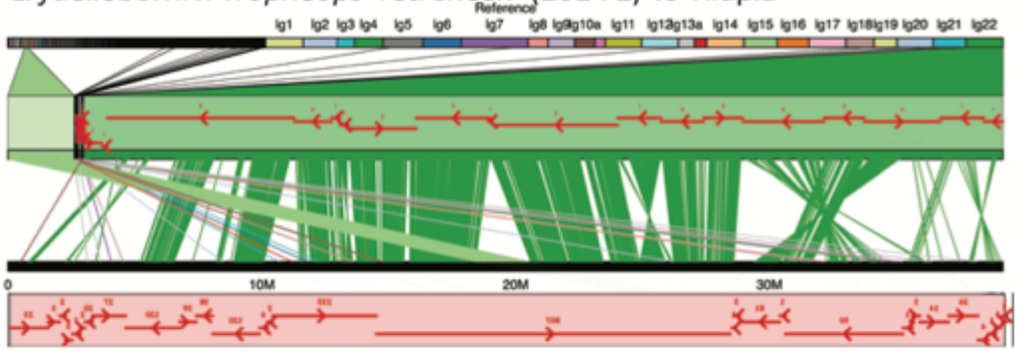

c) M. mbenjii x A. koningsi (331 F2) vs Tilapia

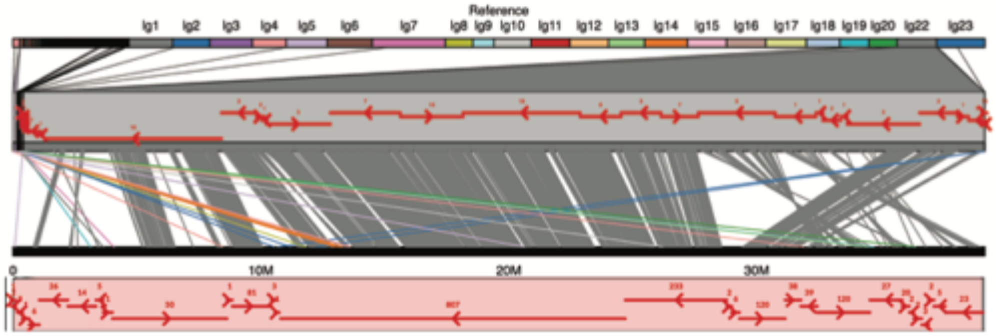

d) M. mbenjii x A. baenschi (161 F2) vs Tilapia

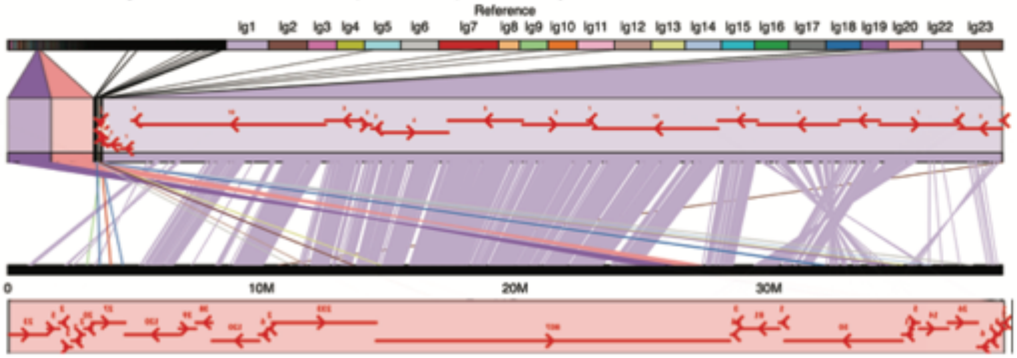

LG23

a) *M. zebra x M. mbenjii* (160 F2) vs Tilapia

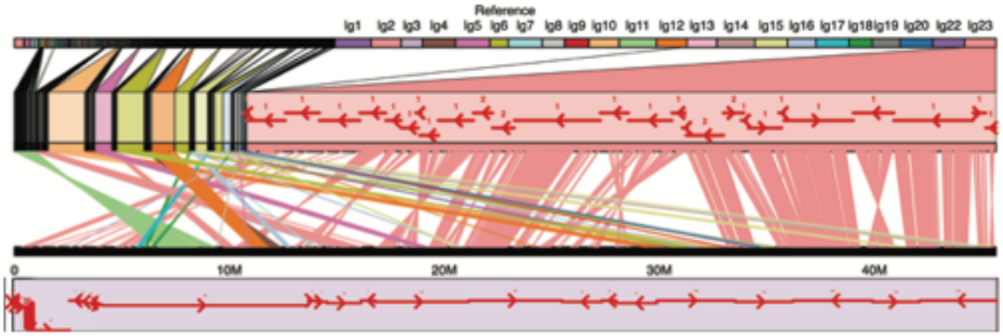

b) *L. fuelleborni x Tropheops 'red cheek'* (262 F2) vs Tilapia

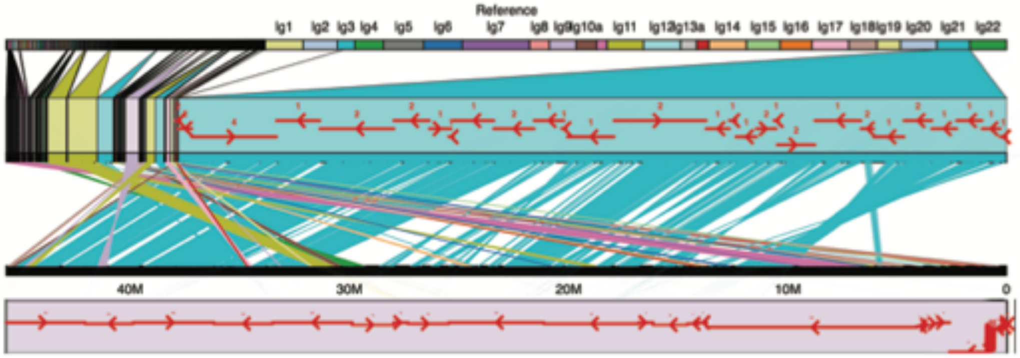

c) *M. mbenjii* x *A. koningsi* (331 F2) vs Tilapia

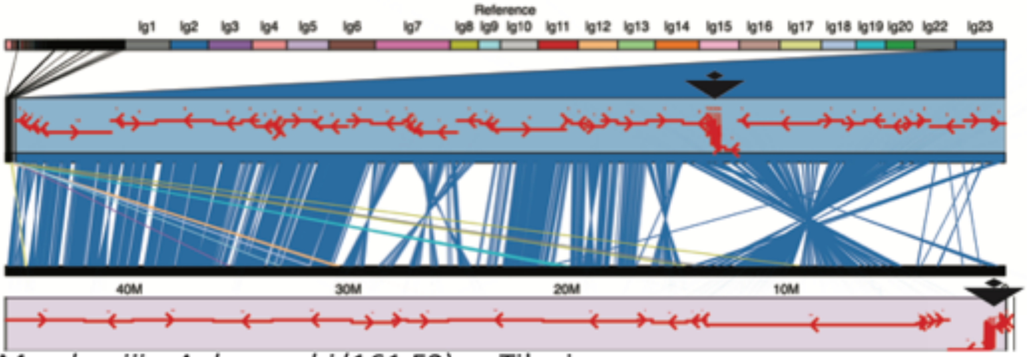

d) *M. mbenjii x A. baenschi* (161 F2) vs Tilapia

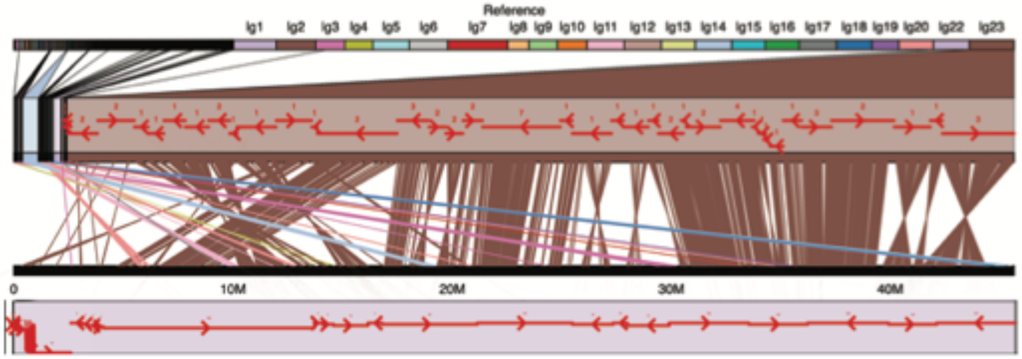

Supplement: Supplement_Files.zip [file giz030_supplement_files.zip › AdditionalFileD_whole_chromosome_alignments.pdf]
