# Supplementary material for: Chromosome-scale assemblies reveal the structural evolution of African cichlid genomes
Source: Gigascience. 2019 Apr 3;8(4):giz030. doi: 10.1093/gigascience/giz030 (PMC6447674; doi:10.1093/gigascience/giz030)
Supplement: Supplement_Files.zip [file giz030_supplement_files.zip › AdditionalFileI_TE_landscape_comparison.pdf]

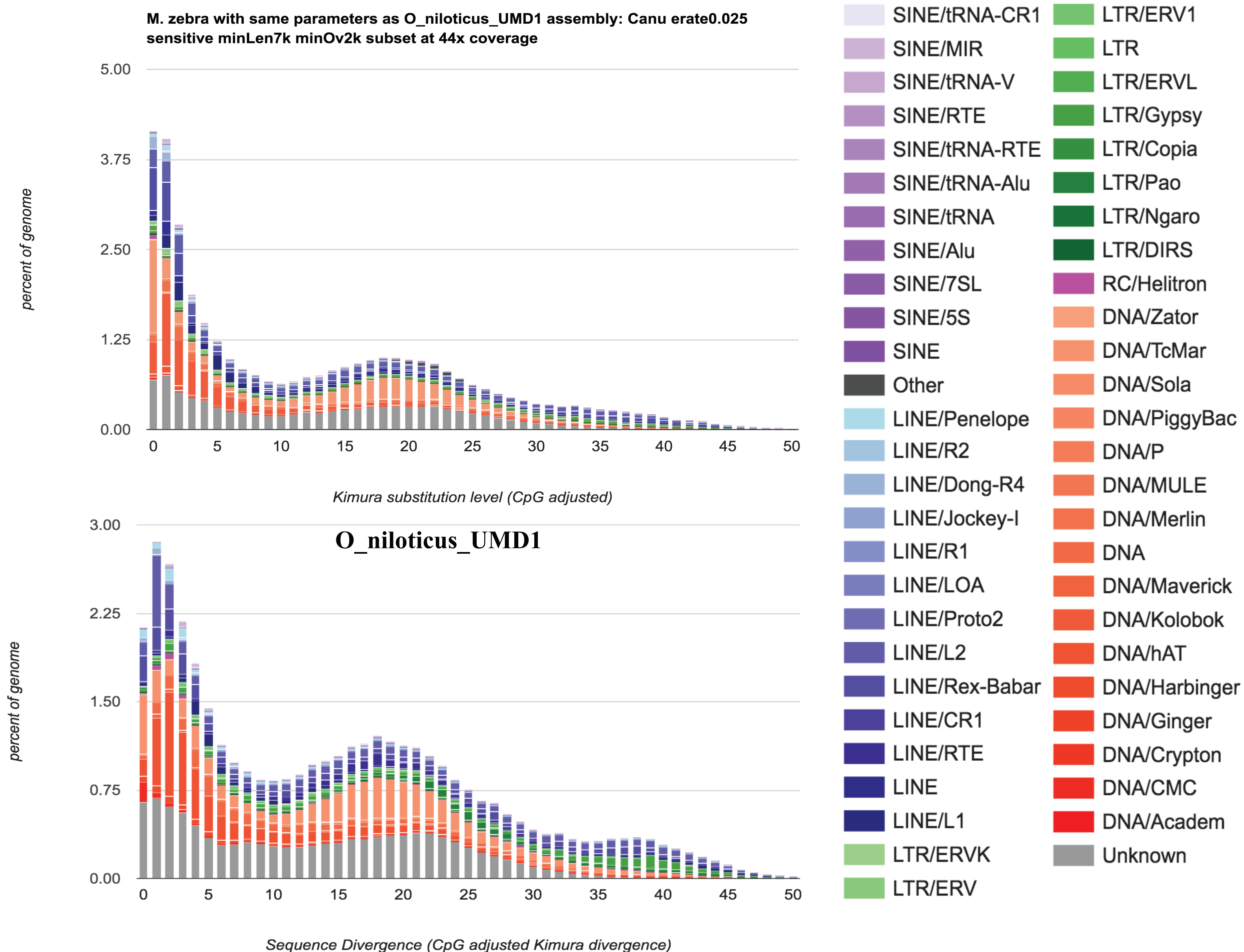

M. zebra with same parameters as O_niloticus_UMD1 assembly: Canu erate0.025 sensitive minLen7k minOv2k subset at 44x coverage
percent of genome
5.00
3.75
2.50
1.25
0.00
0
5
10
15
20
25
30
35
40
45
50
Kimura substitution level (CpG adjusted)
O_niloticus_UMD1
percent of genome
3.00
2.25
1.50
0.75
0.00
0
5
10
15
20
25
30
35
40
45
50
Sequence Divergence (CpG adjusted Kimura divergence)
SINE/tRNA-CR1
SINE/MIR
SINE/tRNA-V
SINE/RTE
SINE/tRNA-RTE
SINE/tRNA-Alu
SINE/tRNA
SINE/Alu
SINE/7SL
SINE/5S
SINE
Other
LINE/Penelope
LINE/R2
LINE/Dong-R4
LINE/Jockey-I
LINE/R1
LINE/LOA
LINE/Proto2
LINE/L2
LINE/Rex-Babar
LINE/CR1
LINE/RTE
LINE
LINE/L1
LTR/ERVK
LTR/ERV
LTR/ERV1
LTR
LTR/ERVL
LTR/Gypsy
LTR/Copia
LTR/Pao
LTR/Ngaro
LTR/DIRS
RC/Helitron
DNA/Zator
DNA/TcMar
DNA/Sola
DNA/PiggyBac
DNA/P
DNA/MULE
DNA/Merlin
DNA
DNA/Maverick
DNA/Kolobok
DNA/hAT
DNA/Harbinger
DNA/Ginger
DNA/Crypton
DNA/CMC
DNA/Academ
Unknown
