# Supplementary figures and images for: Chromosome-scale assemblies reveal the structural evolution of African cichlid genomes
Source: Gigascience. 2019 Apr 3;8(4):giz030. doi: 10.1093/gigascience/giz030 (PMC6447674; doi:10.1093/gigascience/giz030)

**Histogram of 65X coverage M. zebra reads**

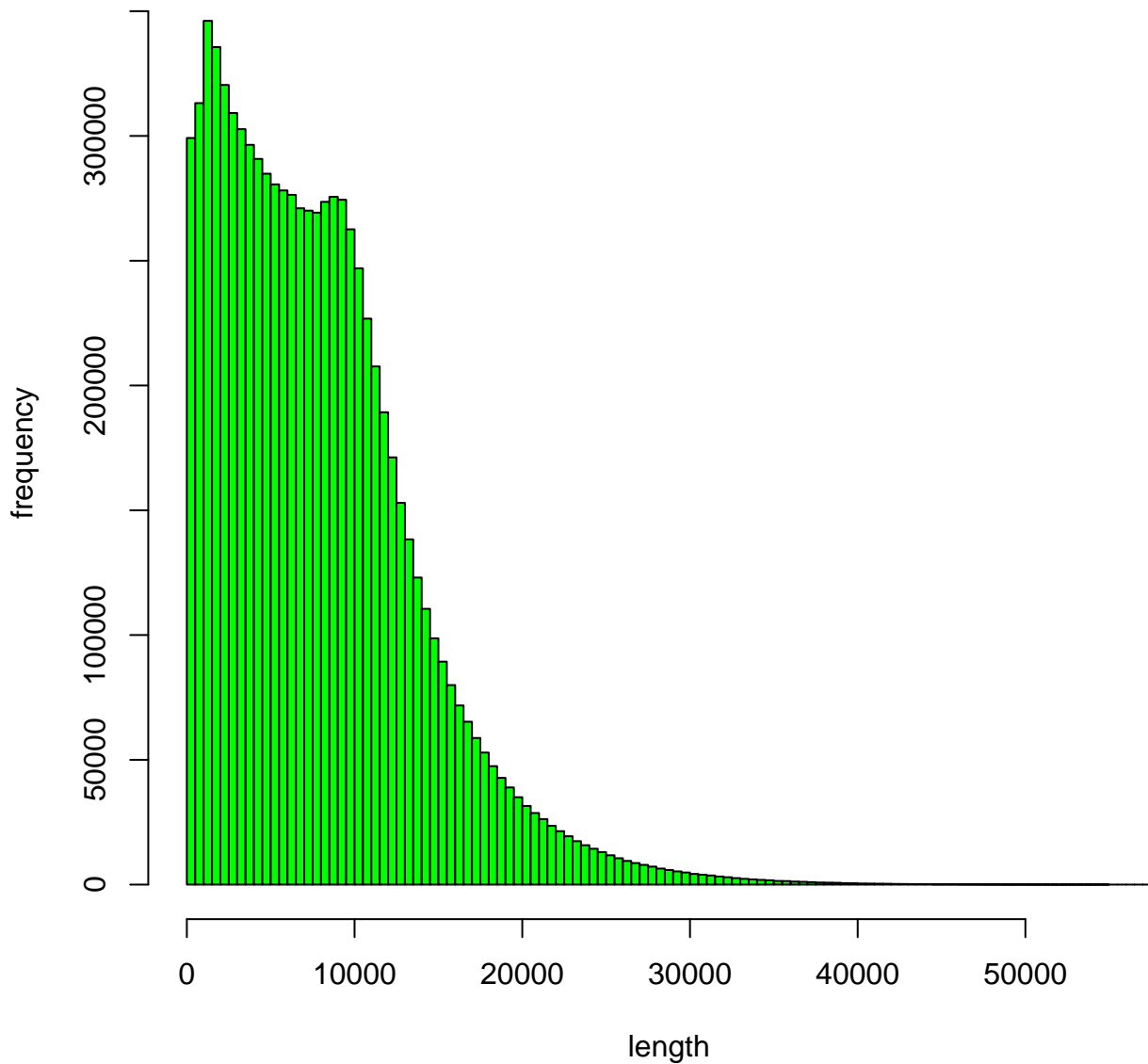

Supplement: Supplement_Files.zip [file giz030_supplement_files.zip › AdditionalFileA_M_zebra_65X_combined_chems_reads.hist.pdf]

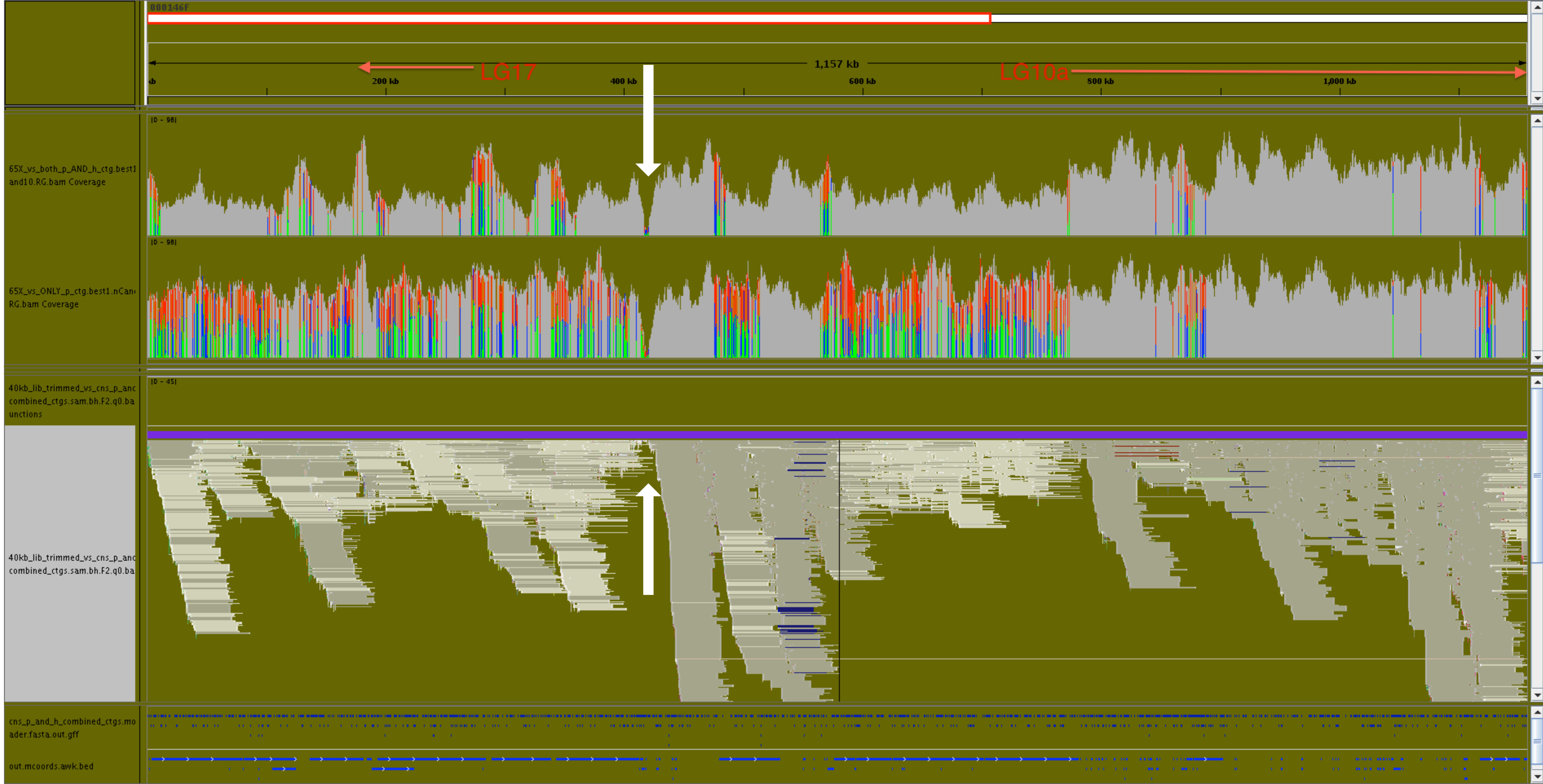

Supplement: Supplement_Files.zip [file giz030_supplement_files.zip › AdditionalFileC_contig_000146F_1_1162490.pdf]

# Linkage Group 3

(a)

*O. aureus*  $F_{ST}$  on *O. niloticus*\_UMD\_NMBU

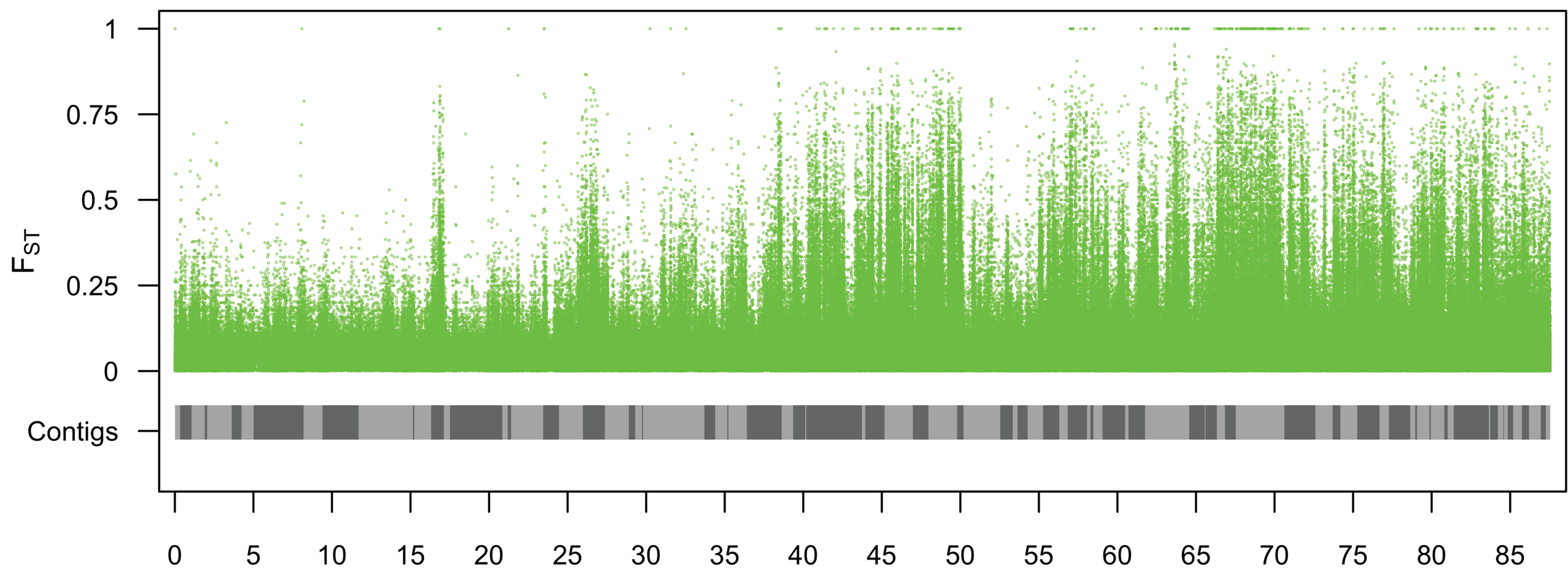

(b)

Position (Mb)

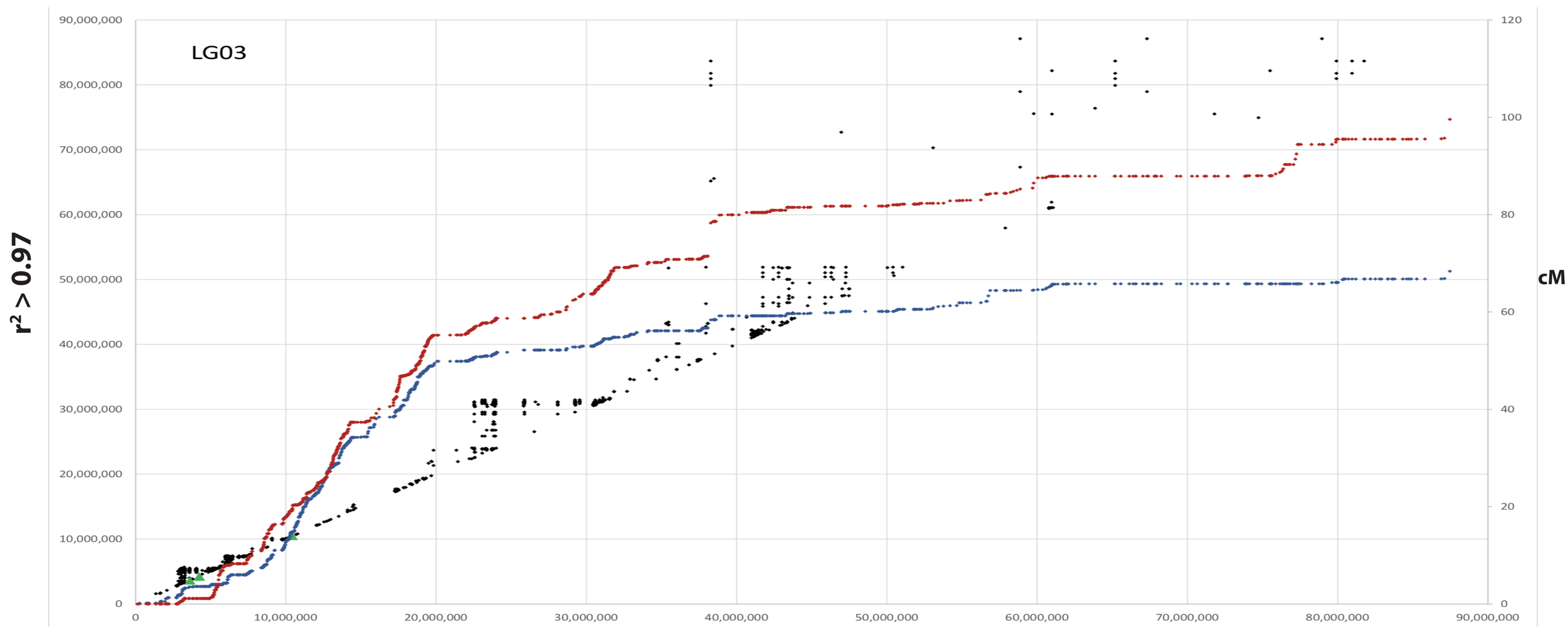

Supplement: Supplement_Files.zip [file giz030_supplement_files.zip › AdditionalFileE_LG3_Fst_recombination.pdf]

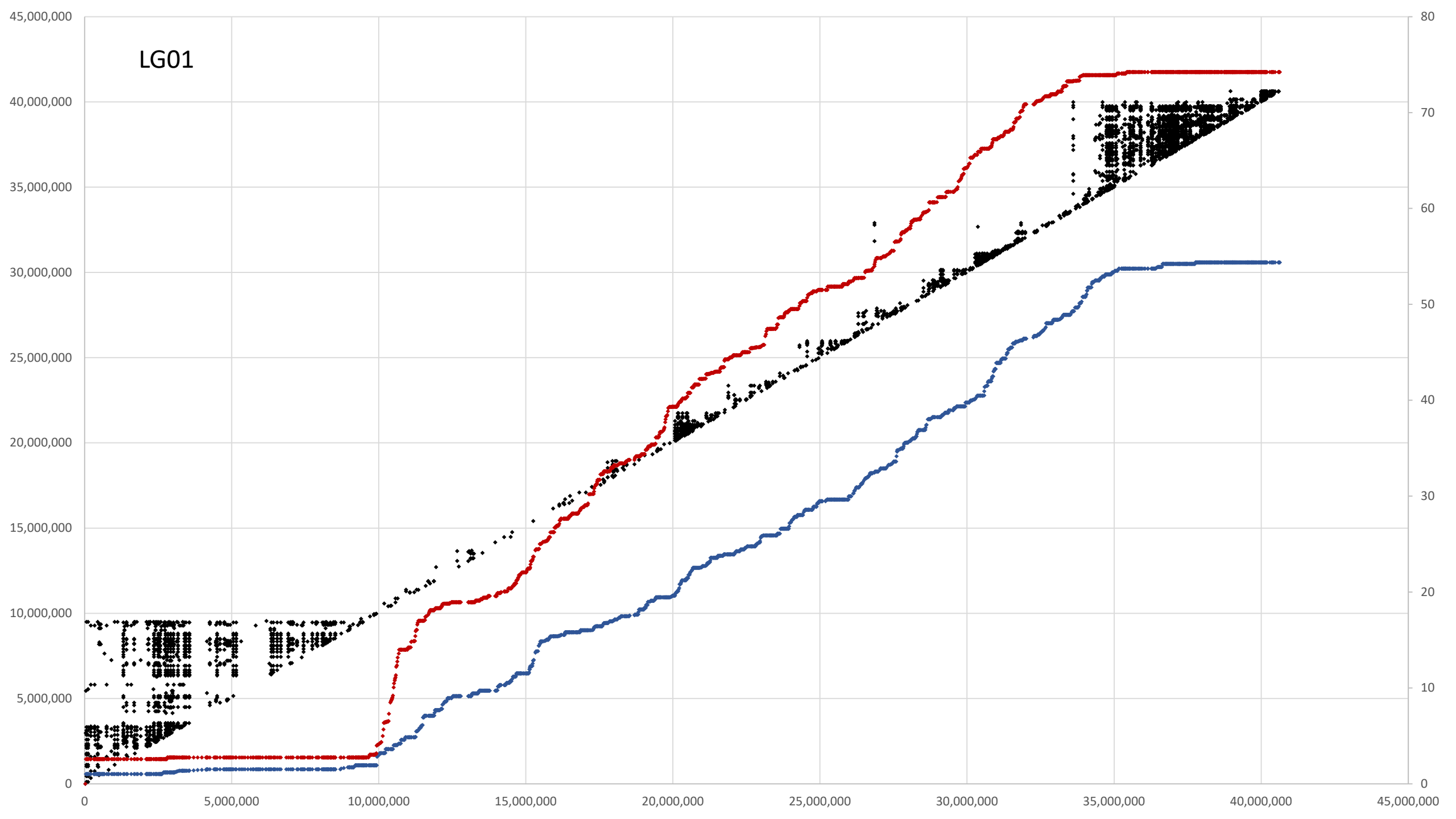

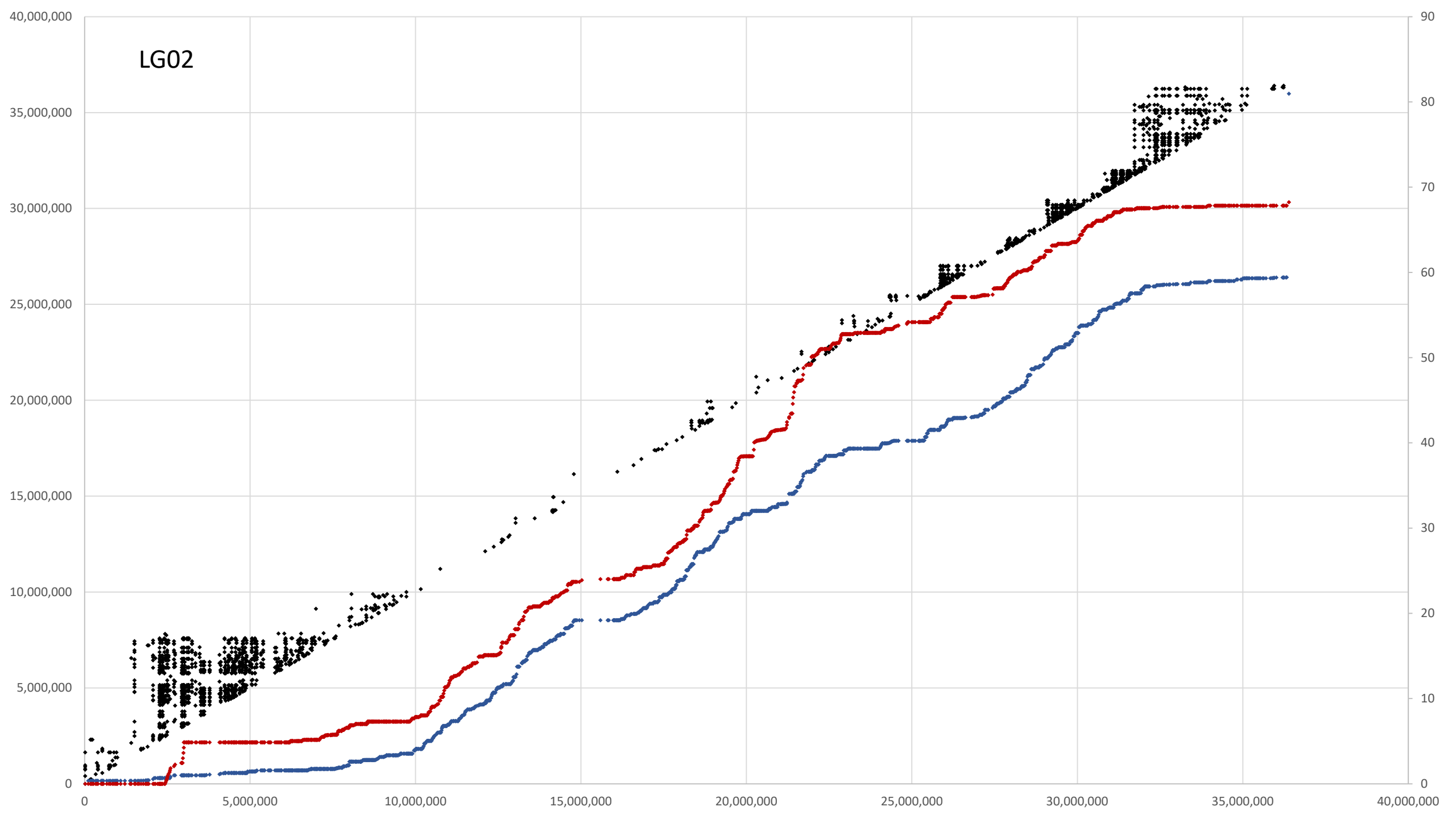

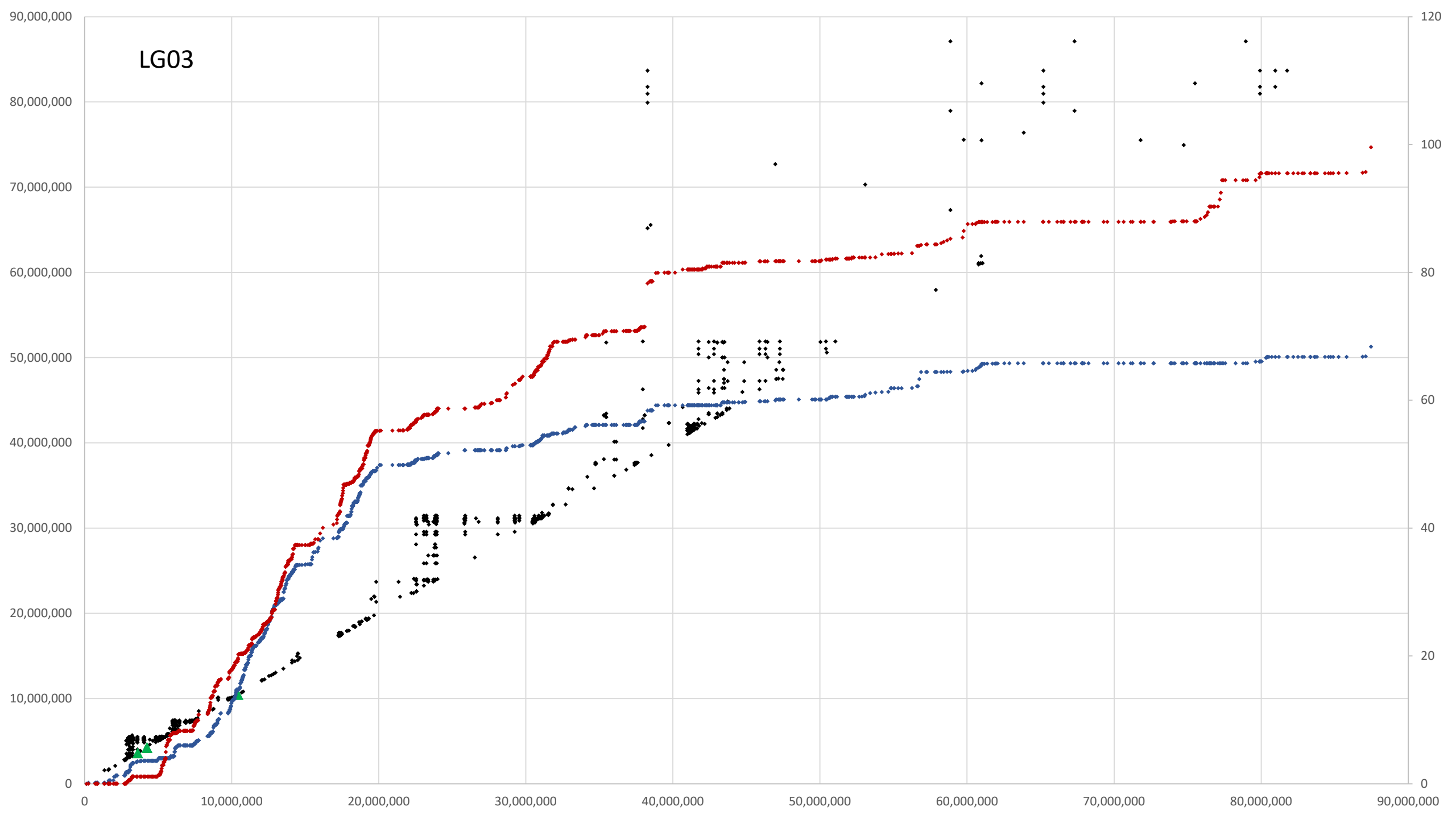

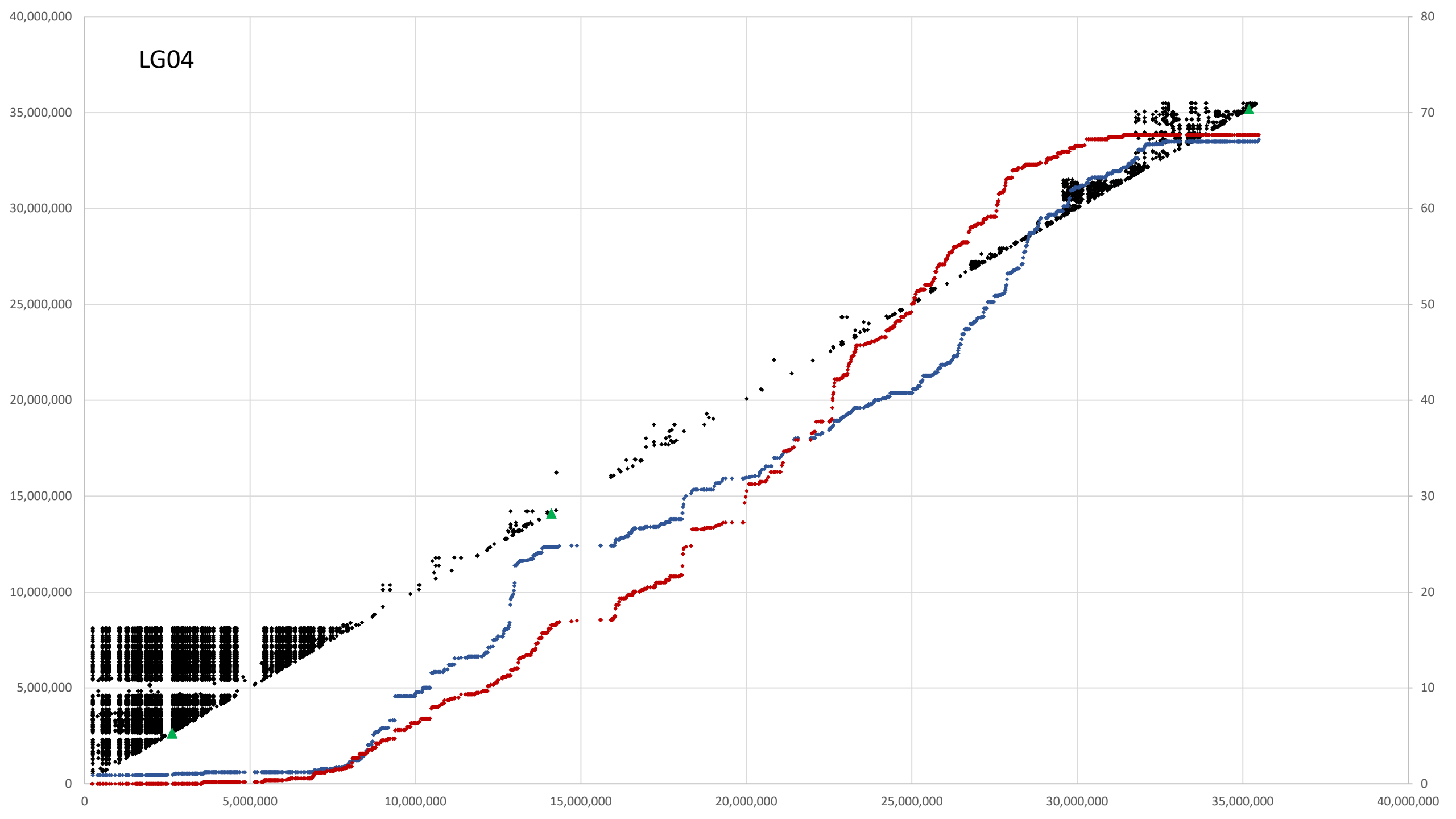

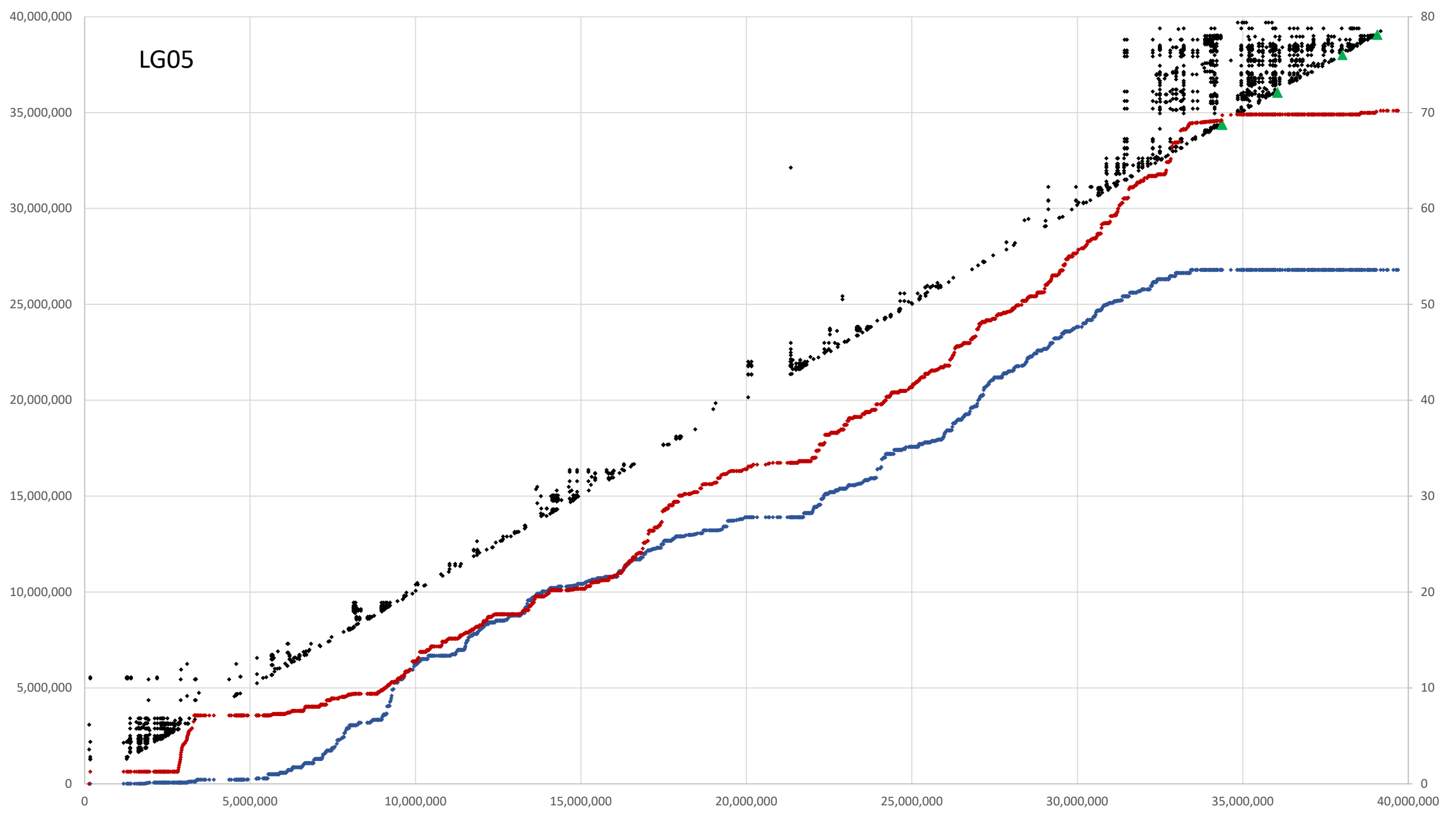

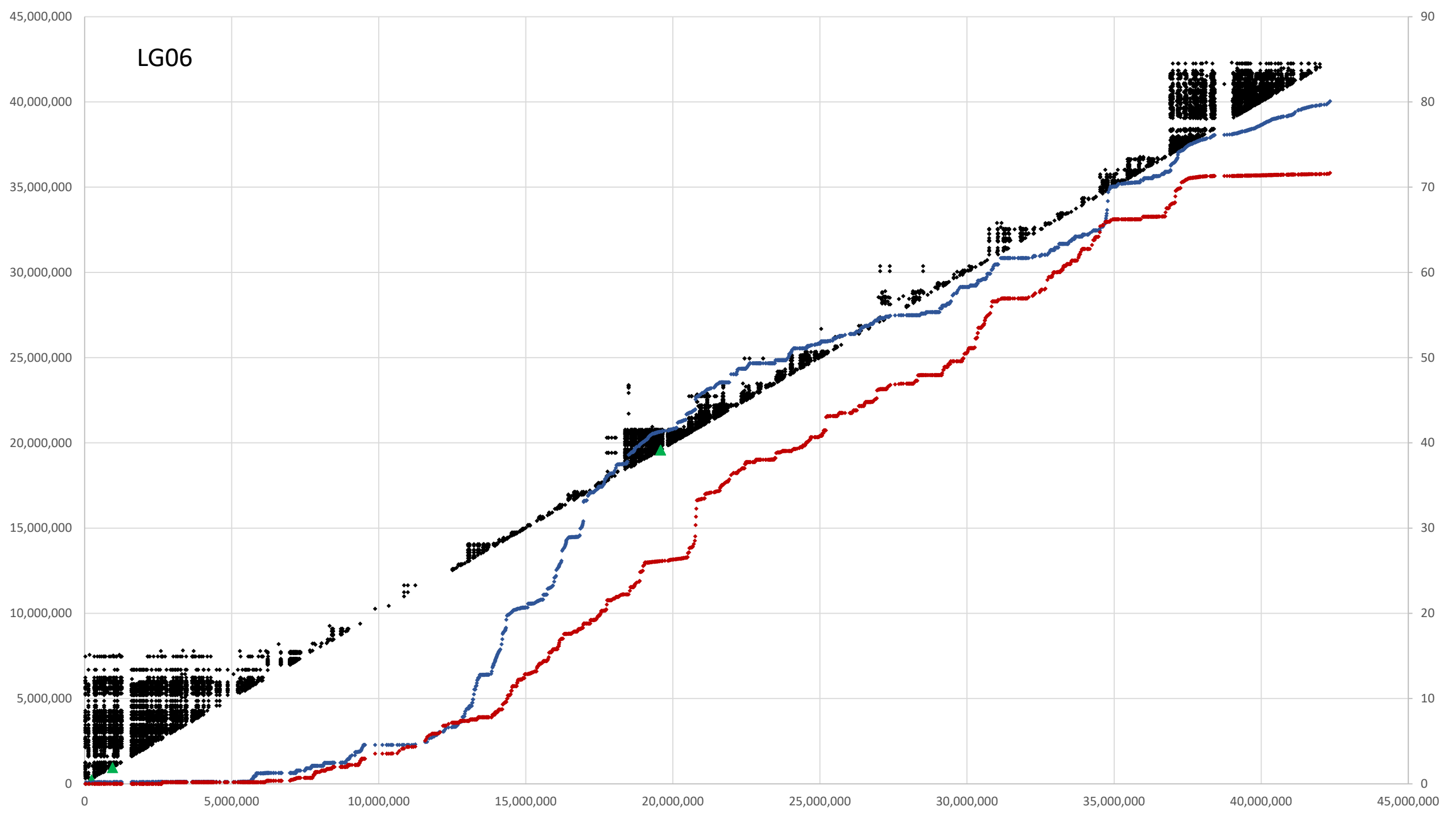

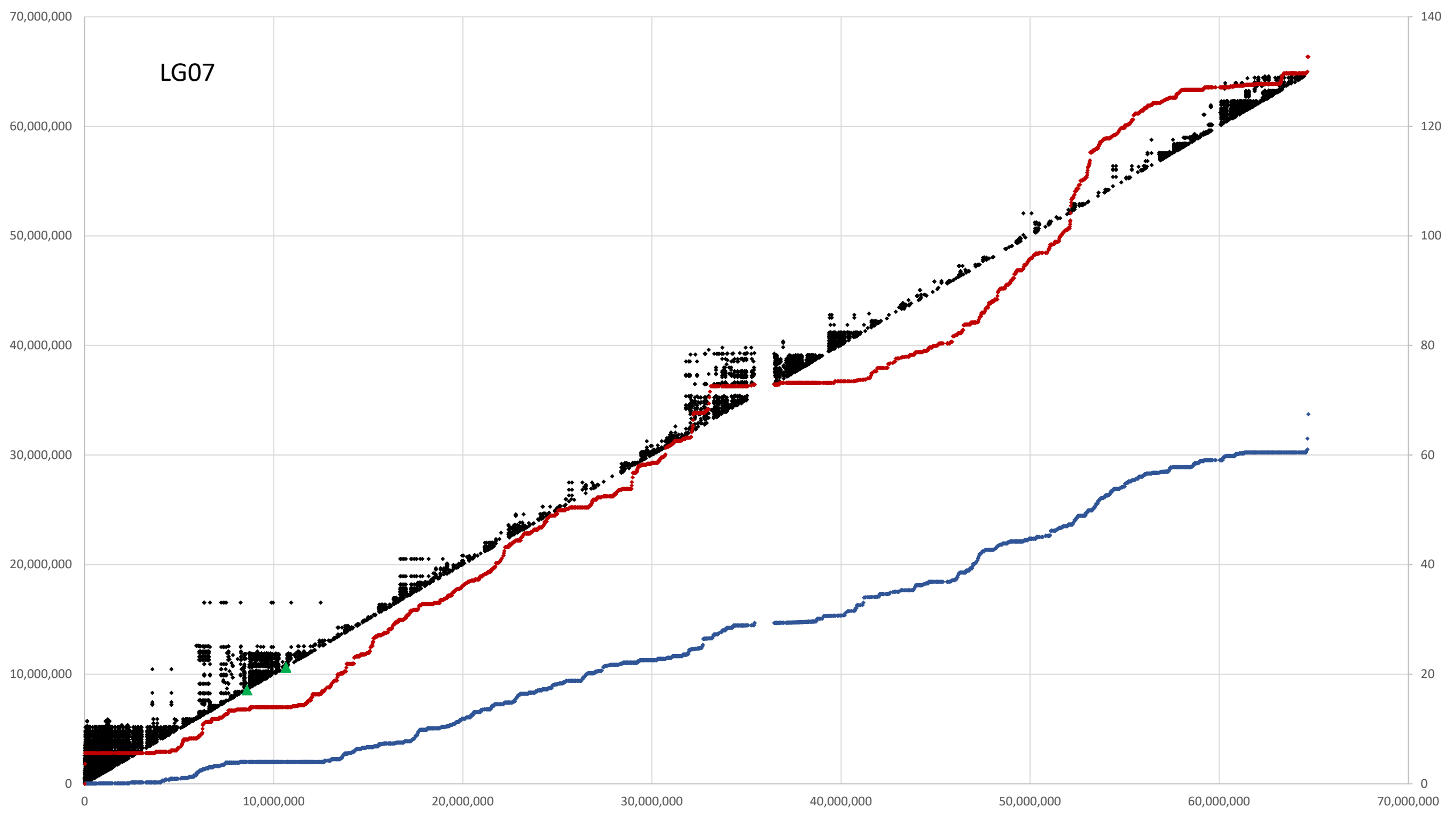

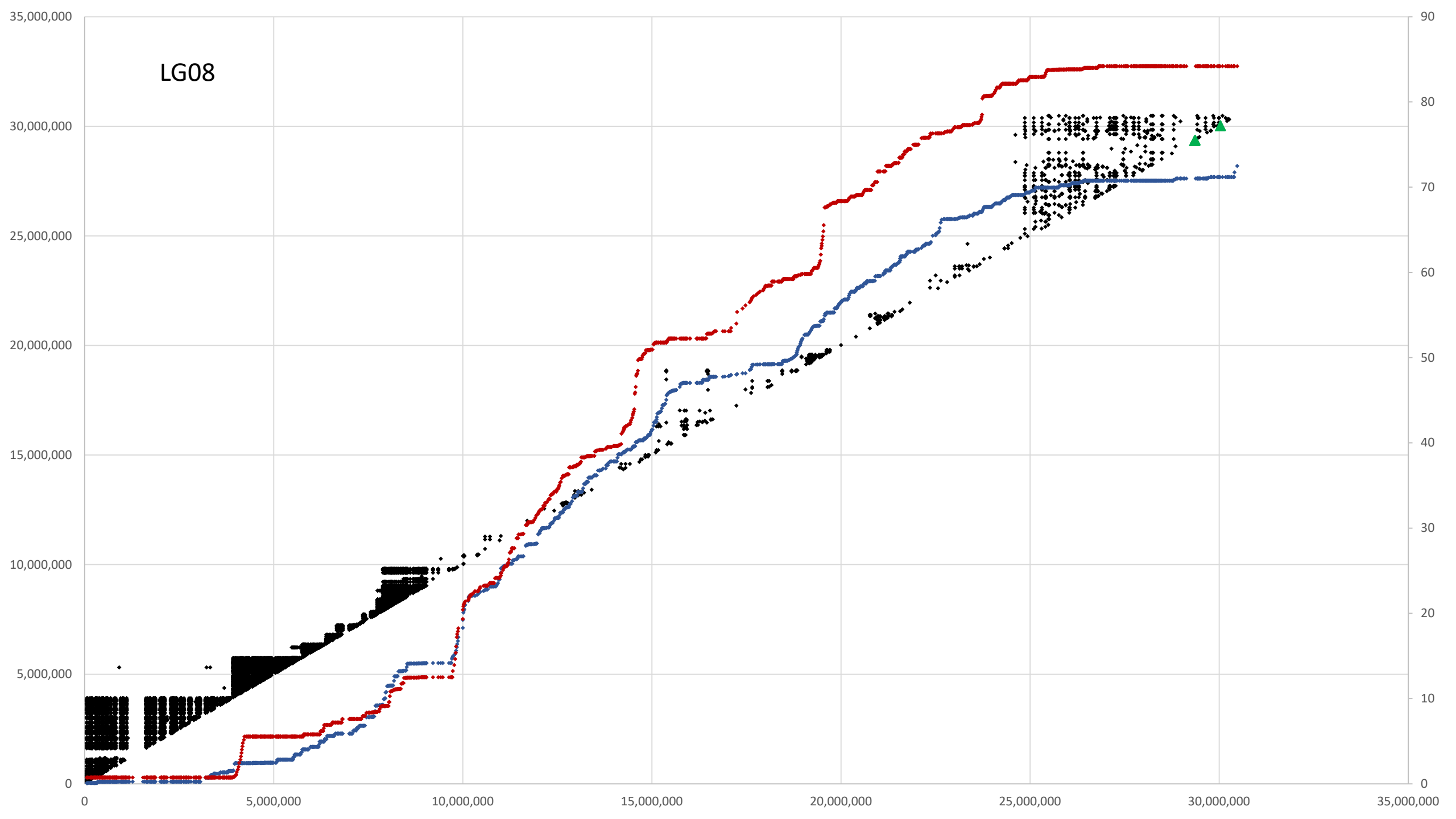

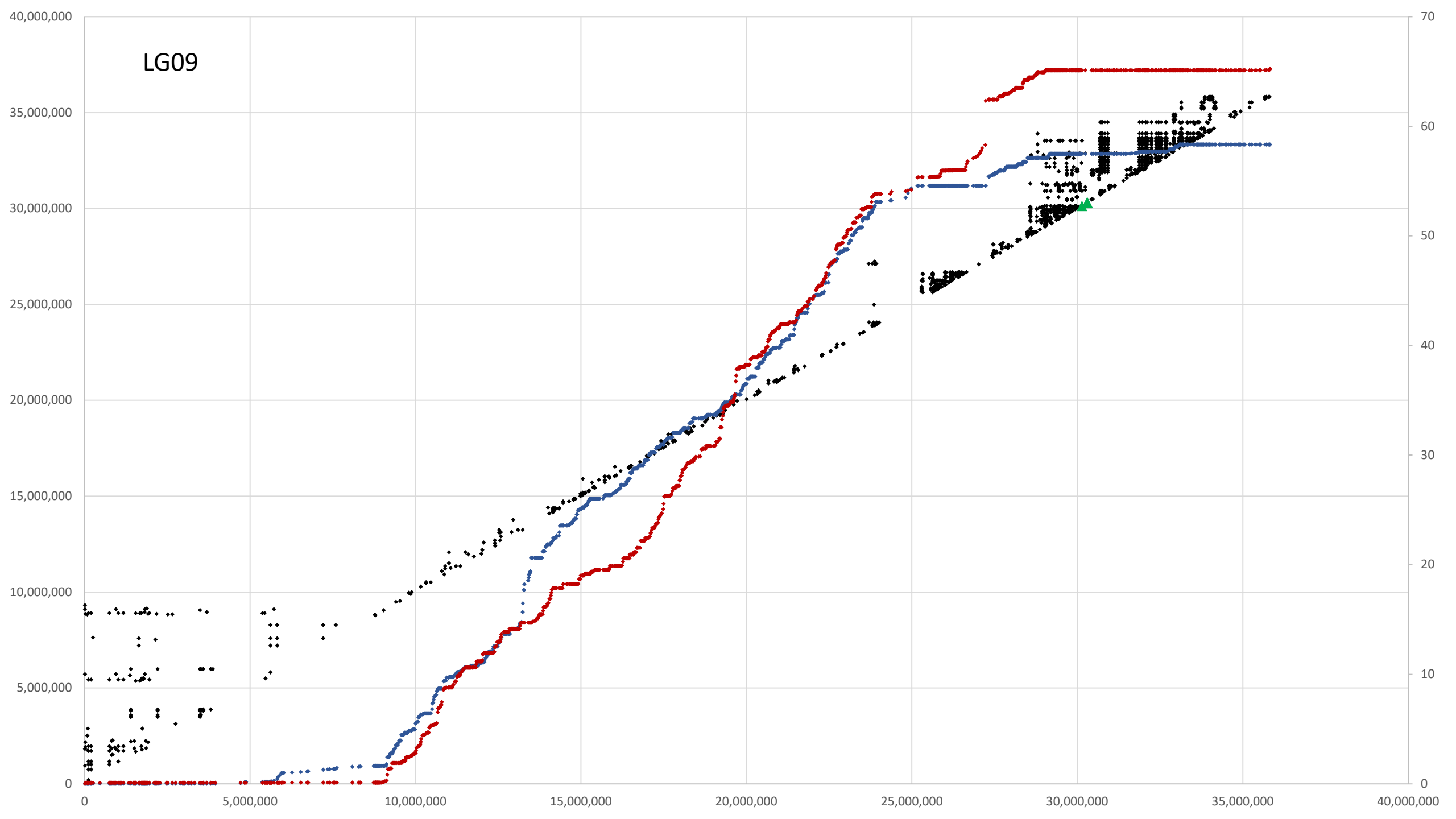

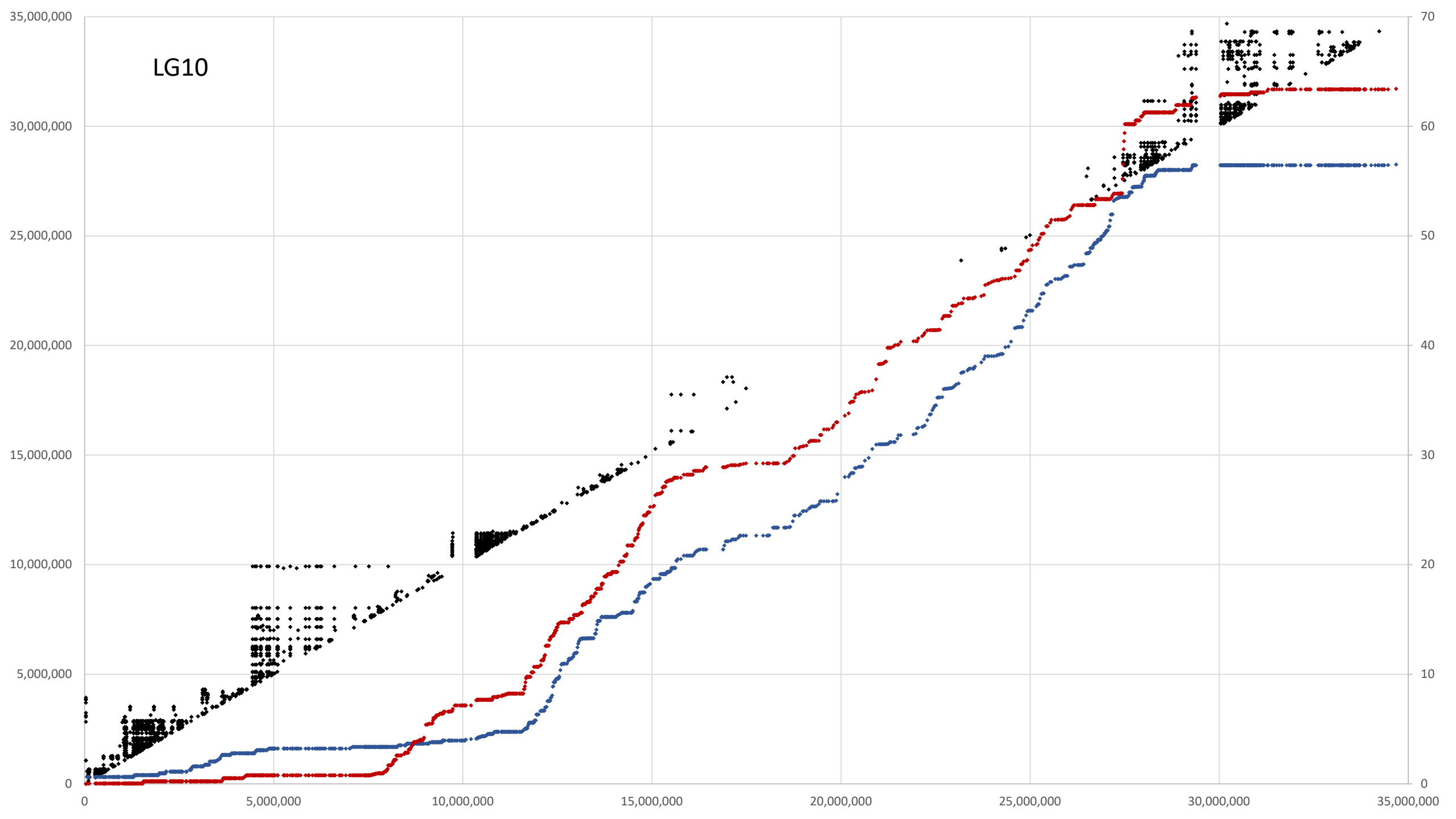

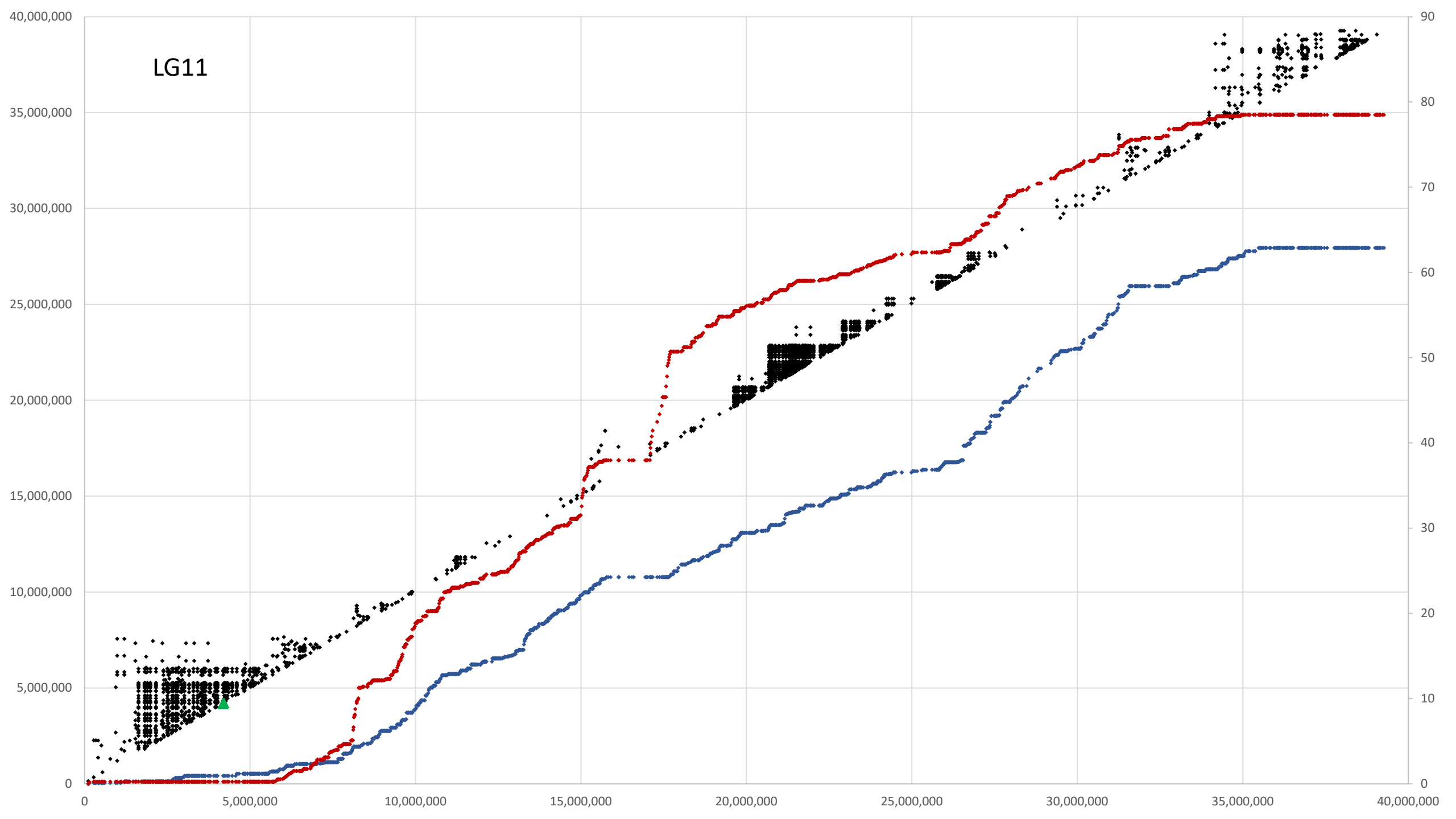

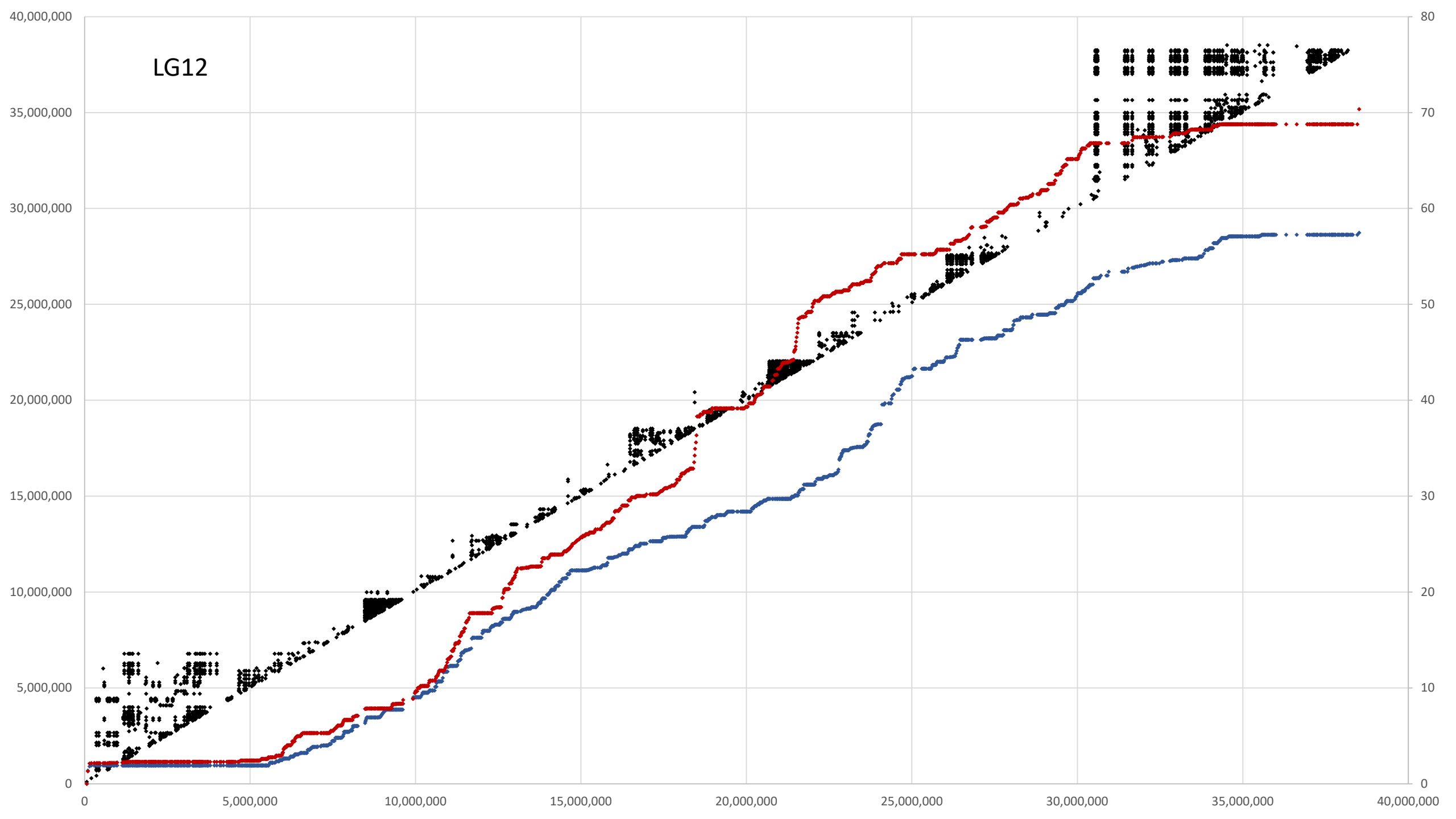

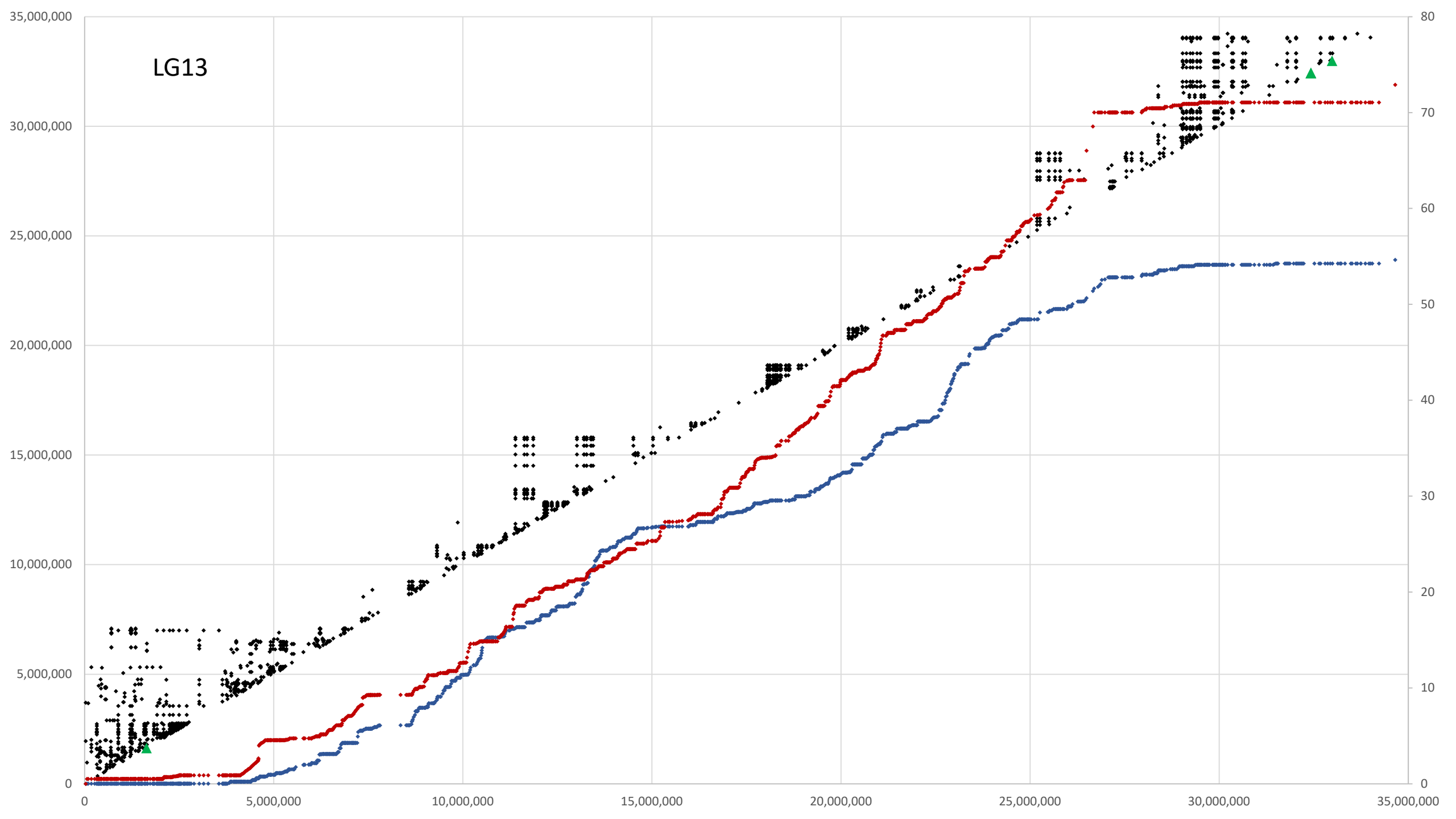

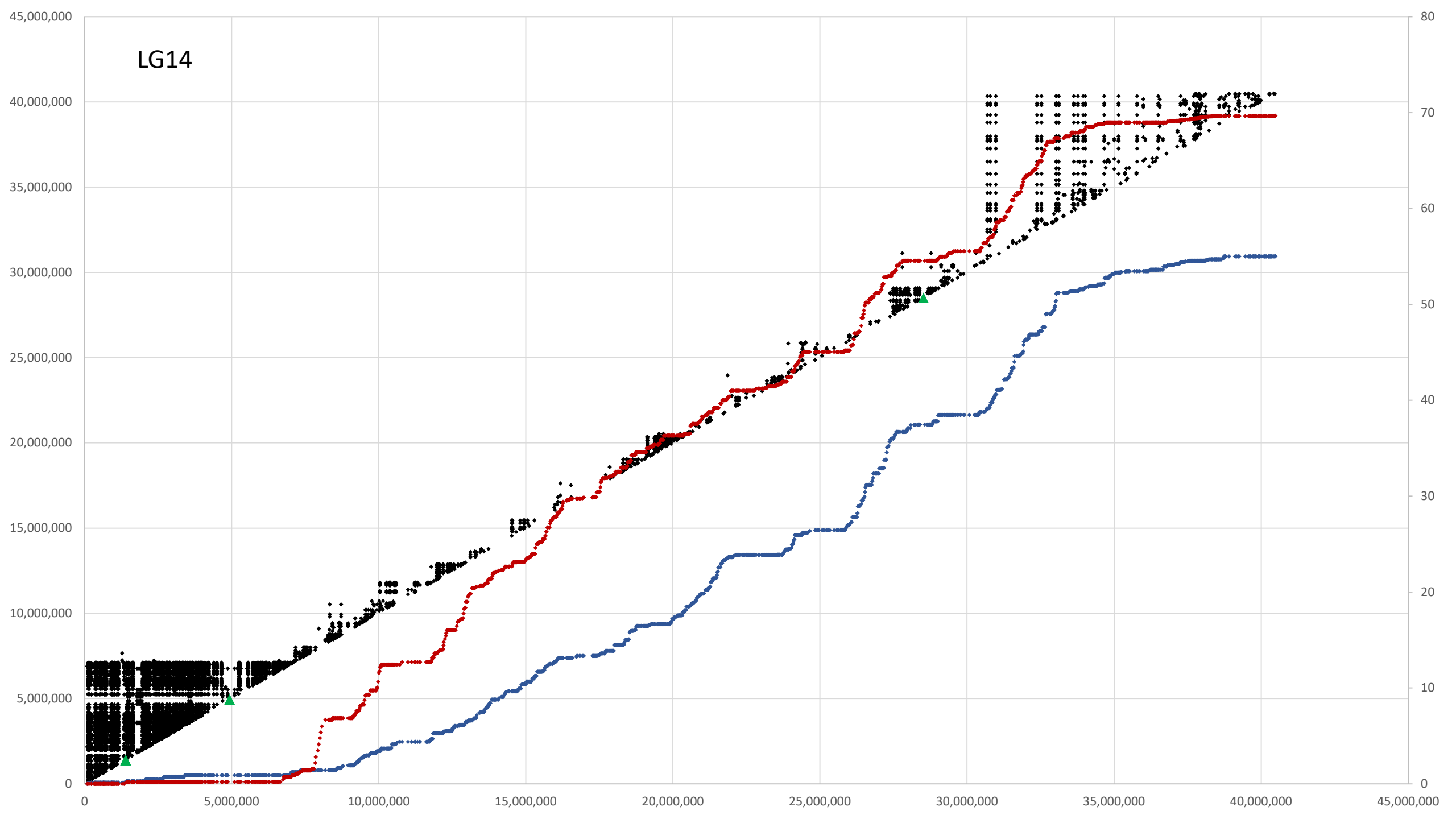

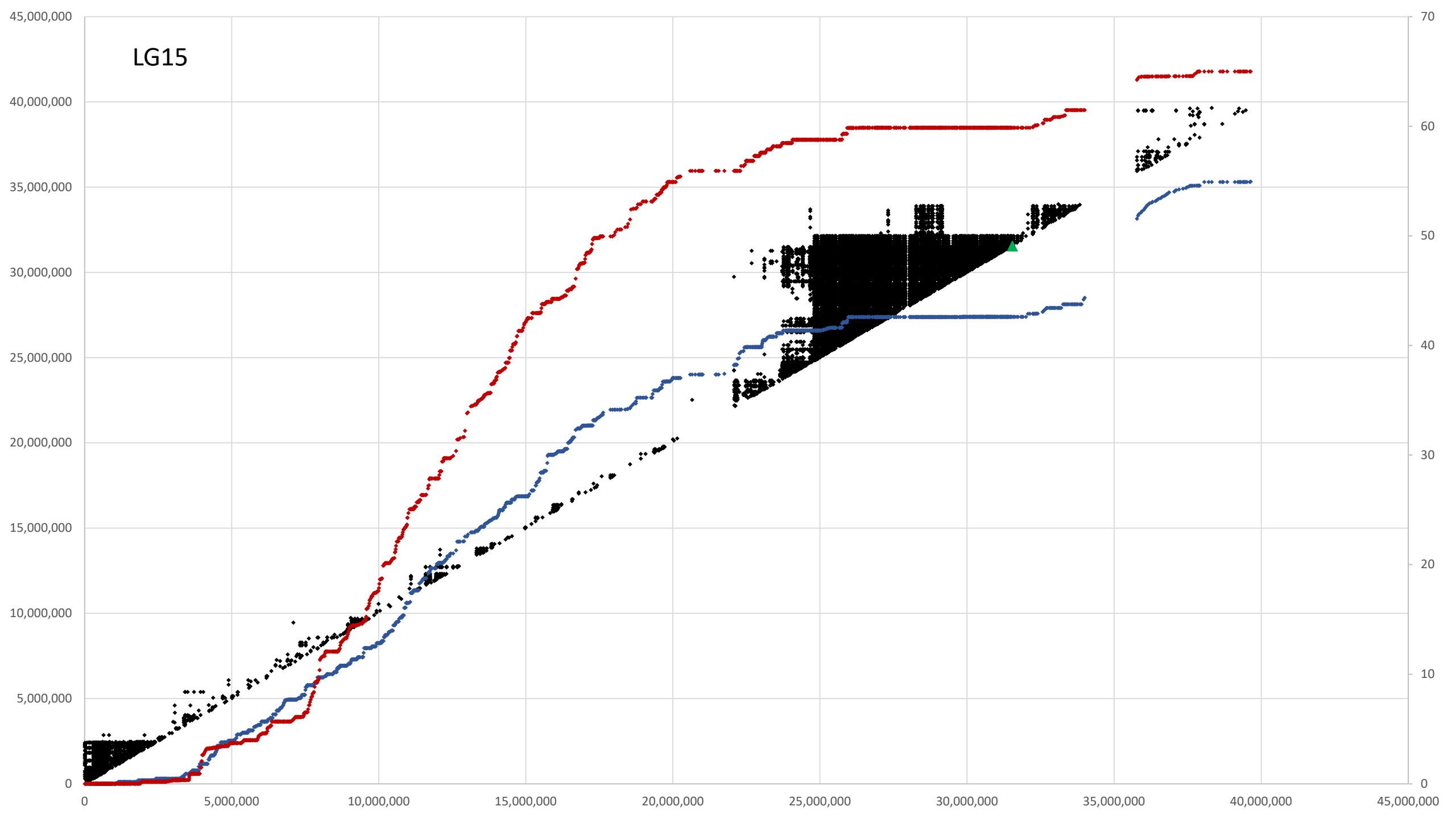

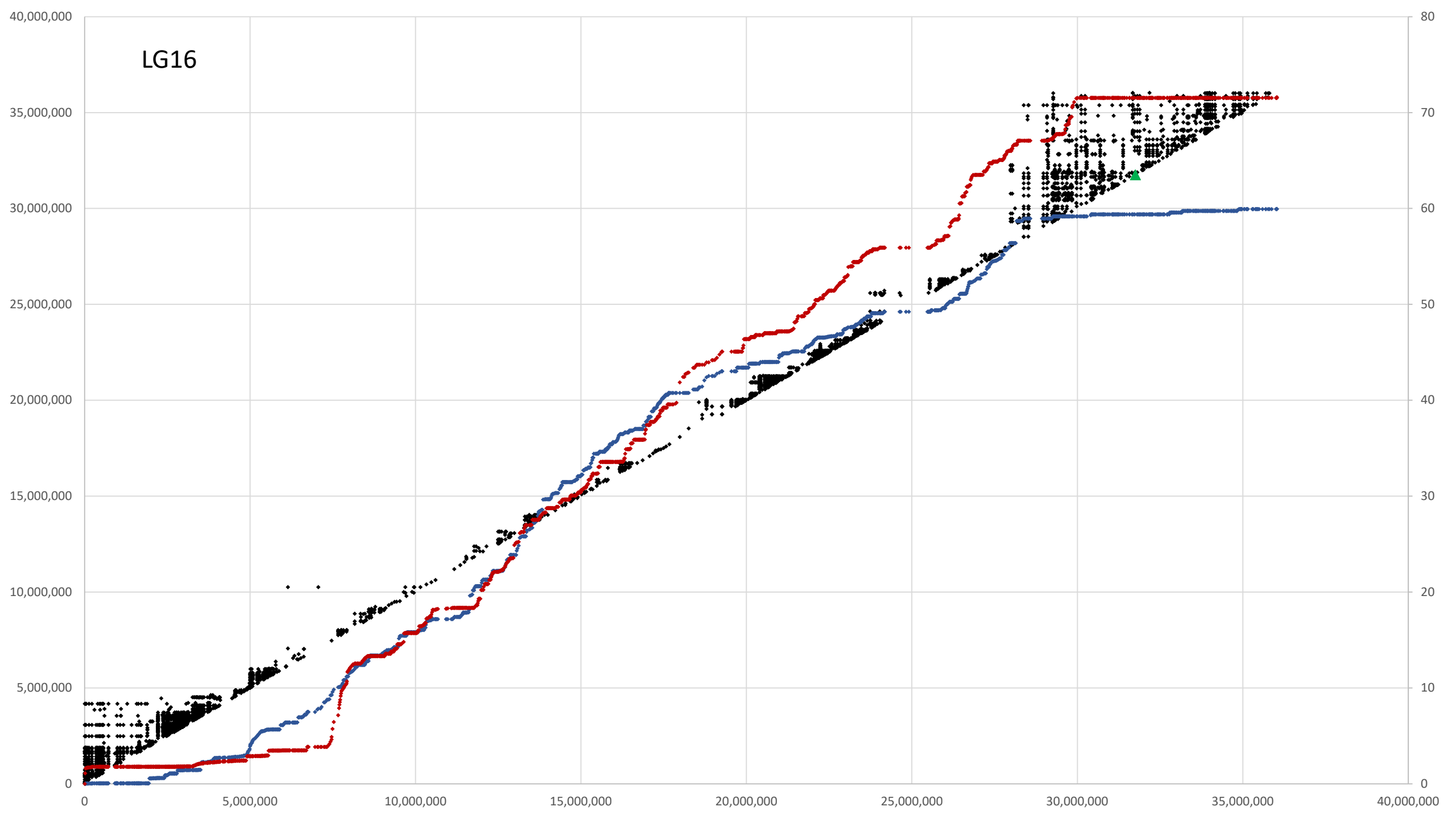

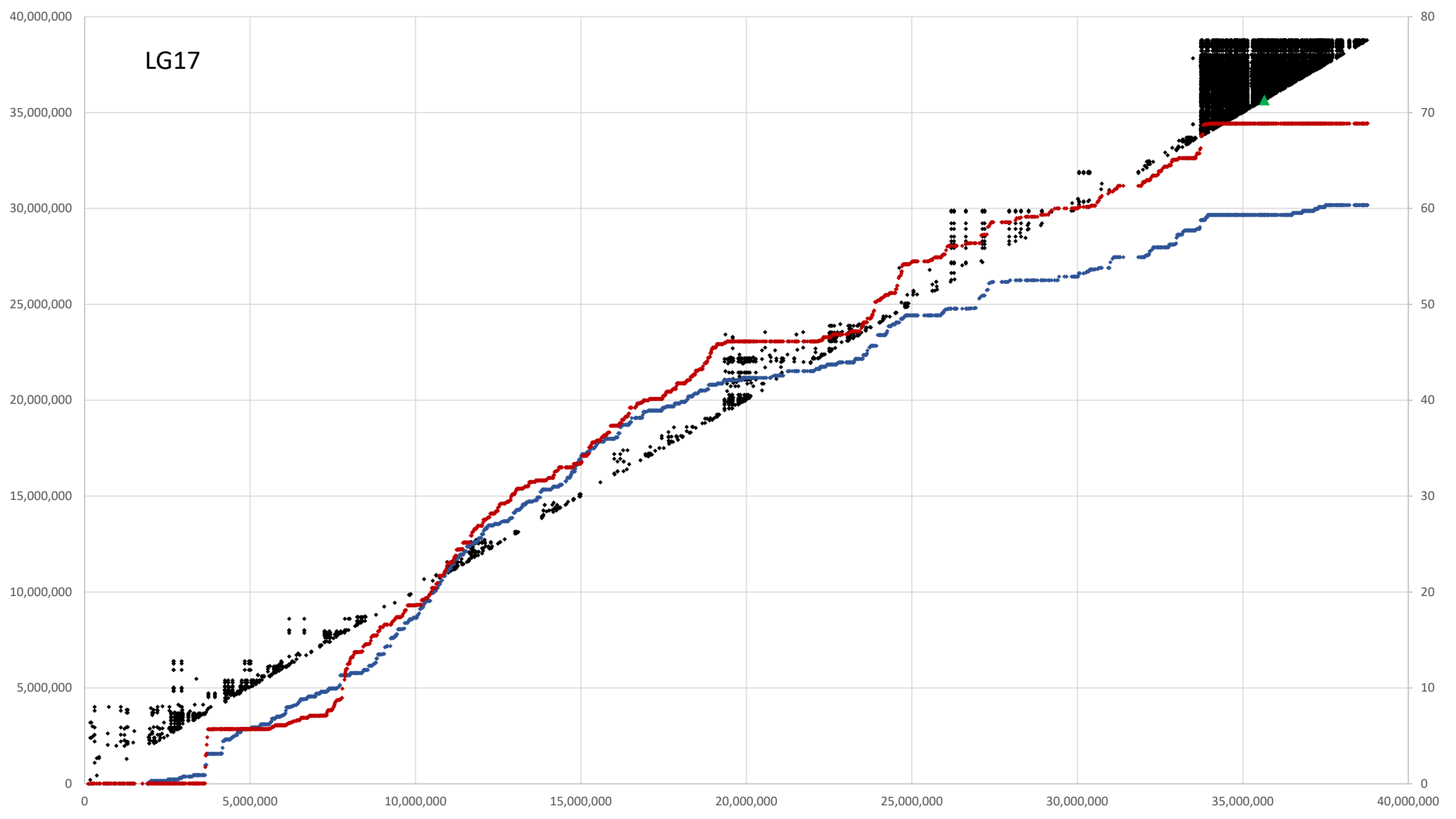

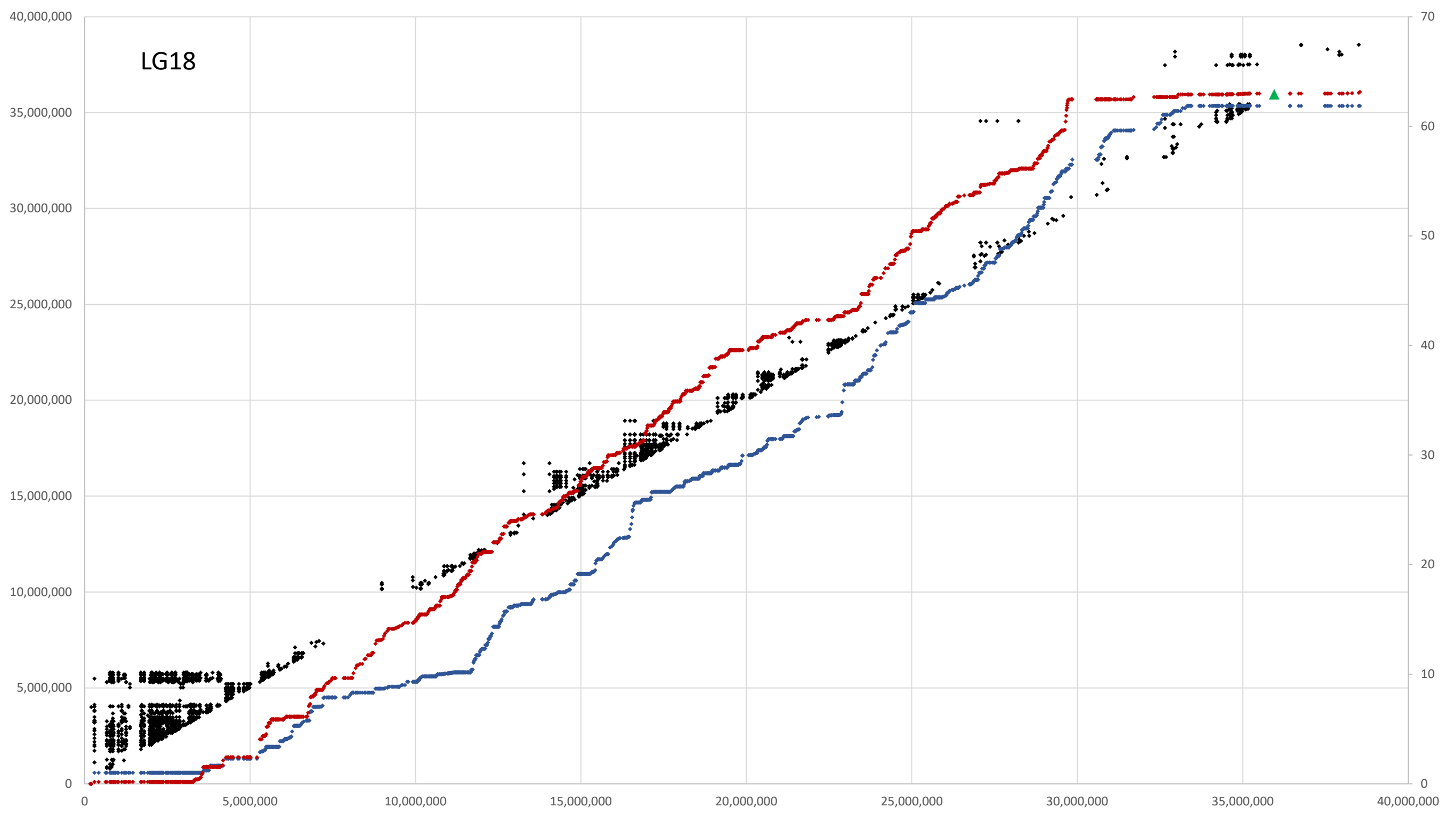

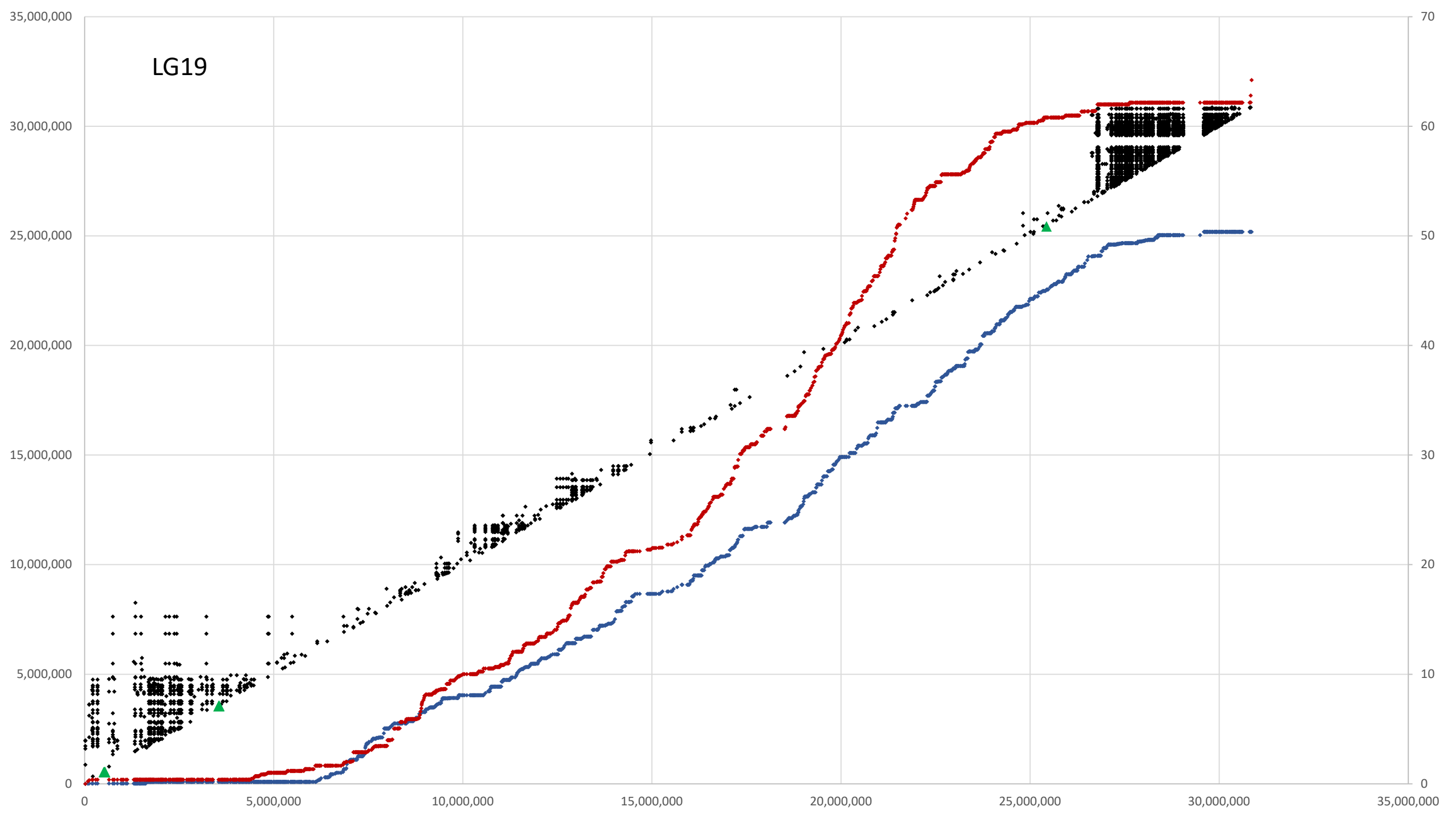

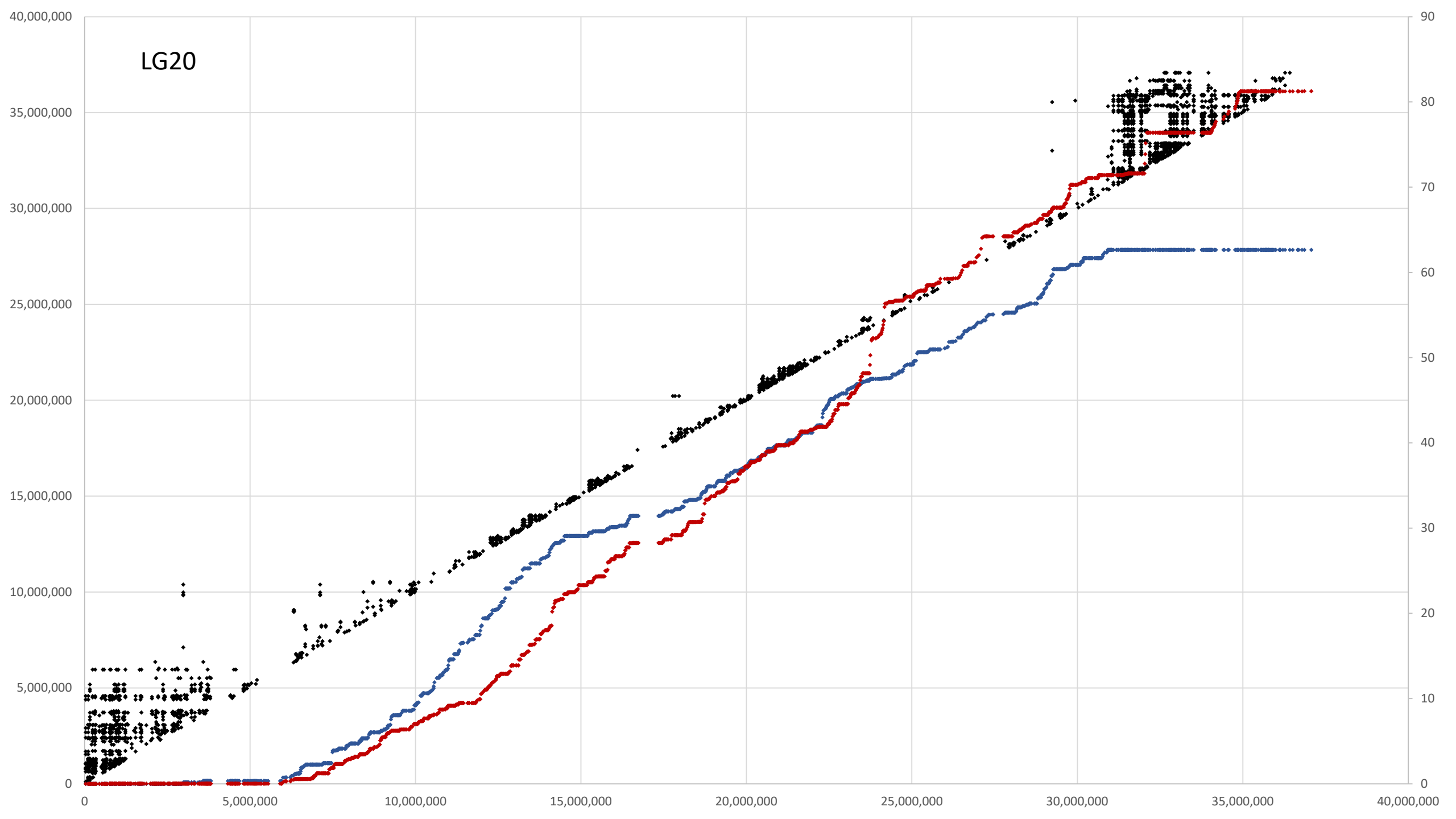

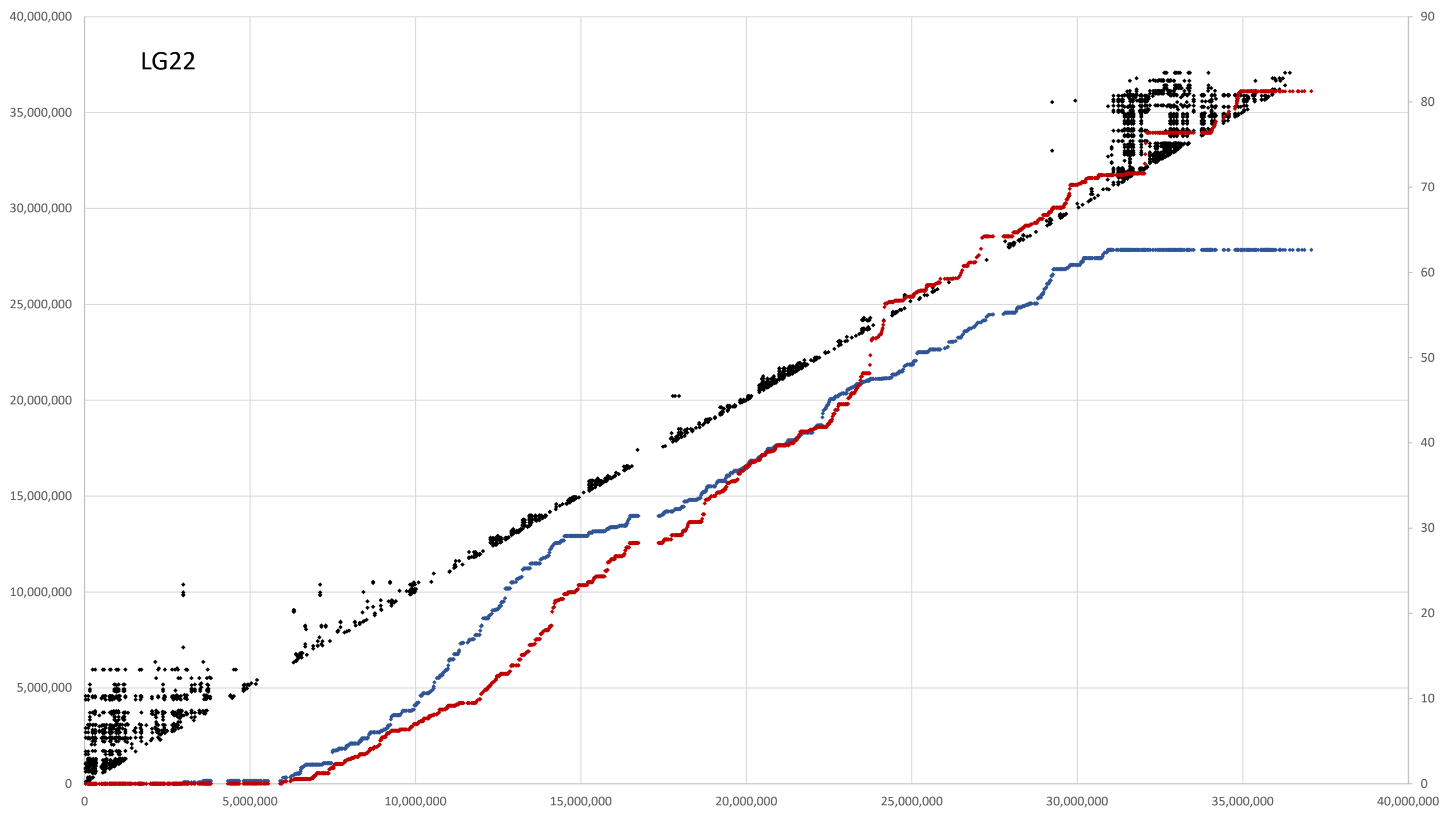

LG23

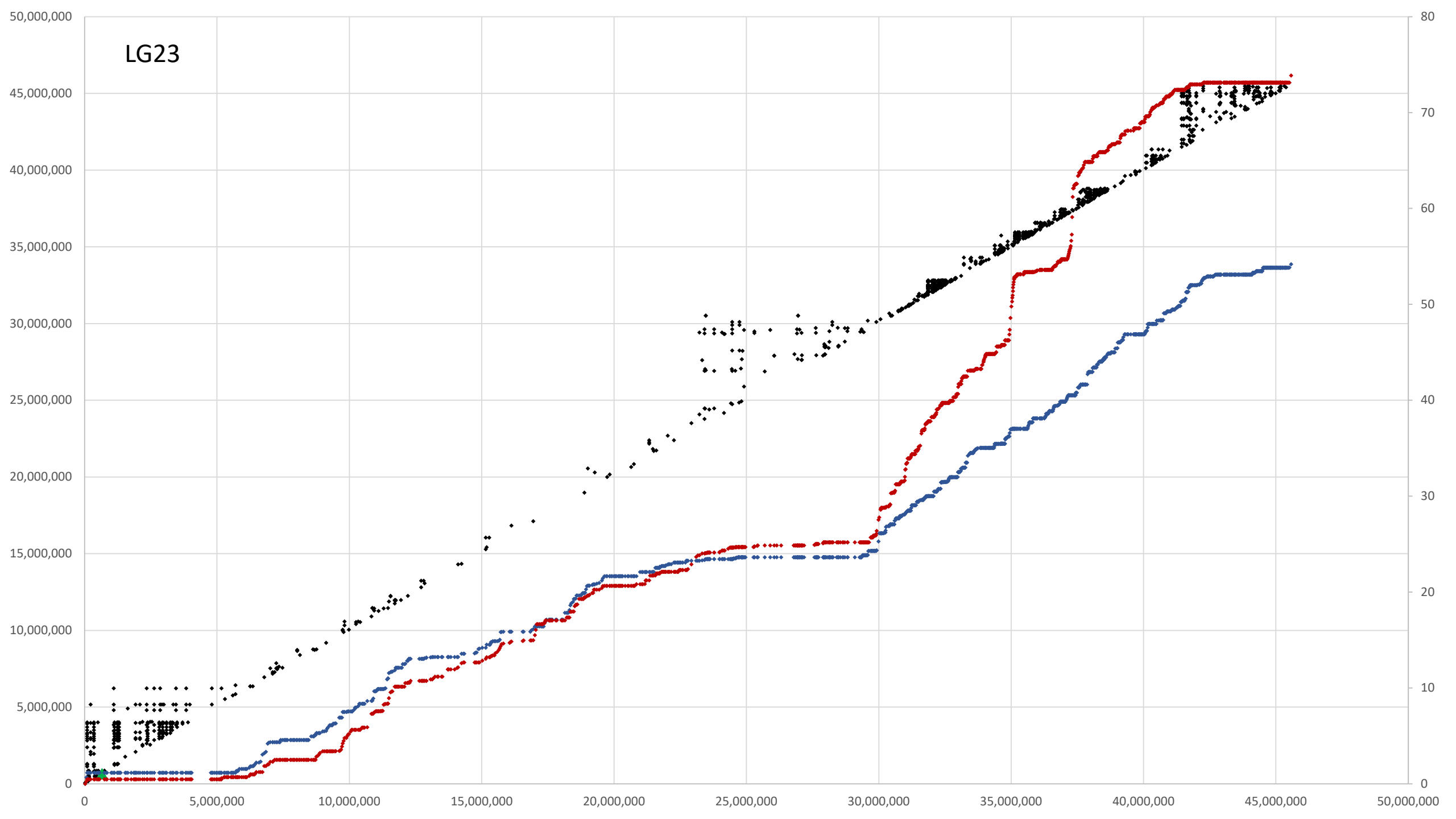

Supplement: Supplement_Files.zip [file giz030_supplement_files.zip › AdditionalFileF_tilapia_recombination_plots.pdf]

lg1

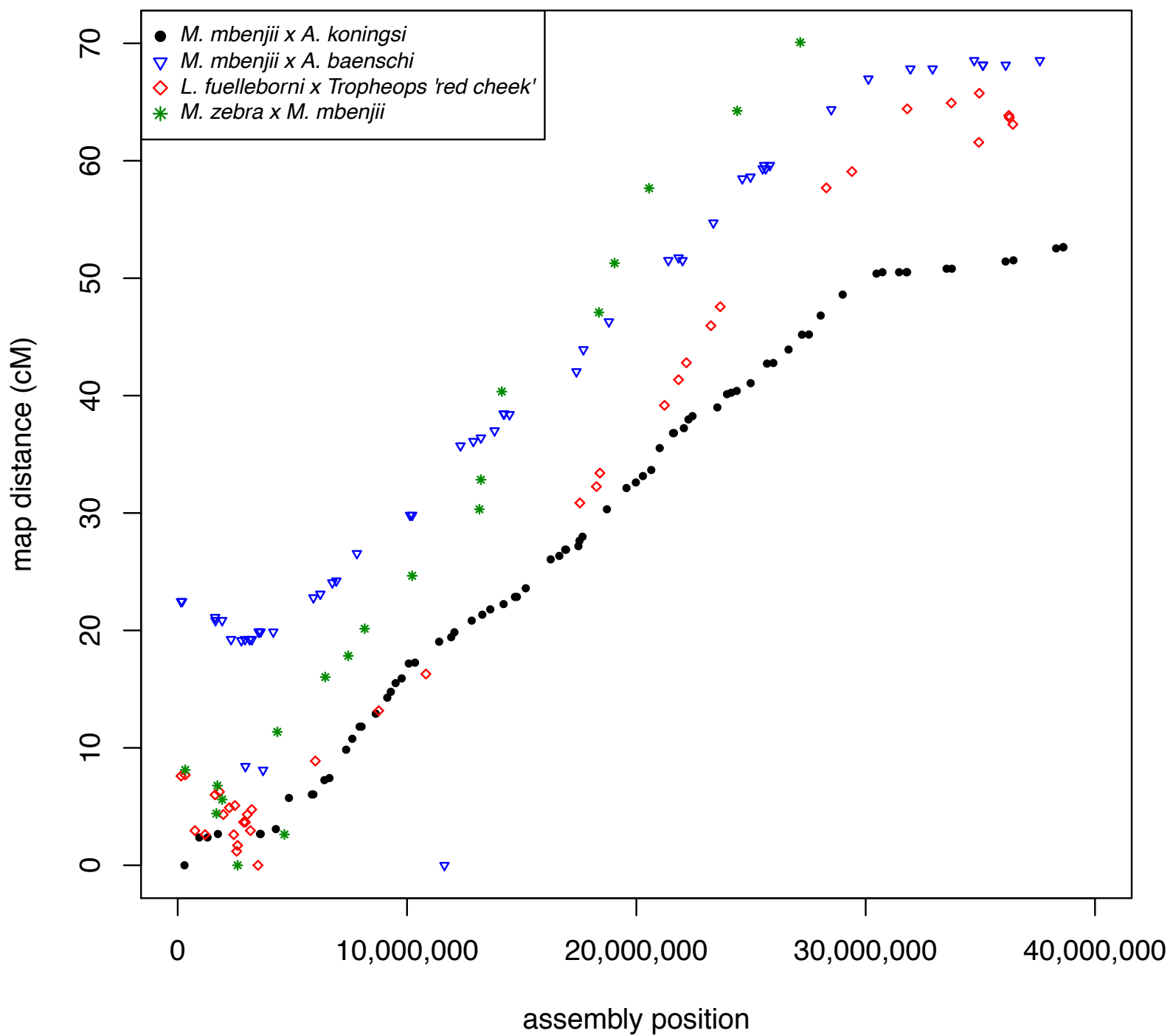

lg2

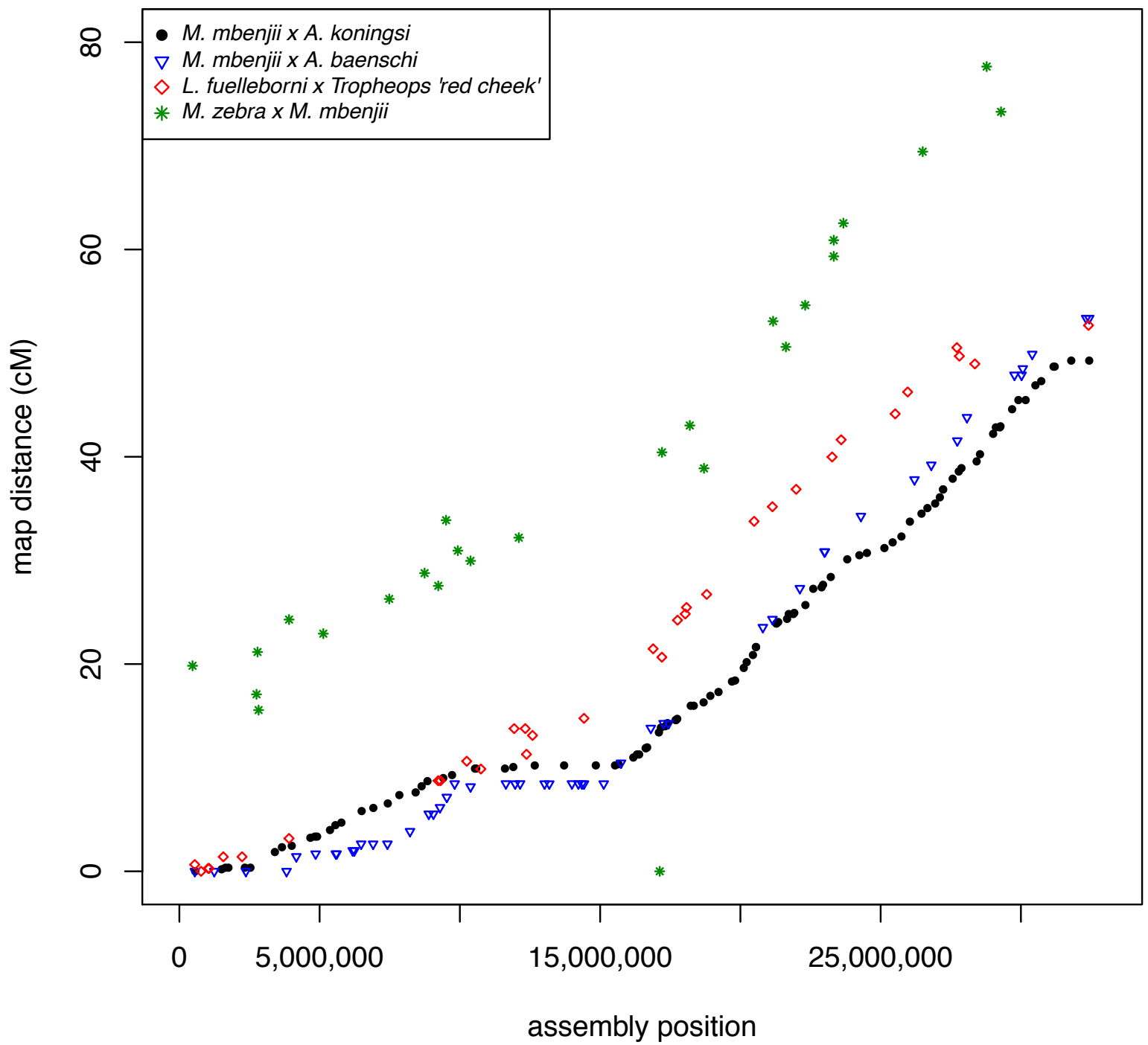

lg3

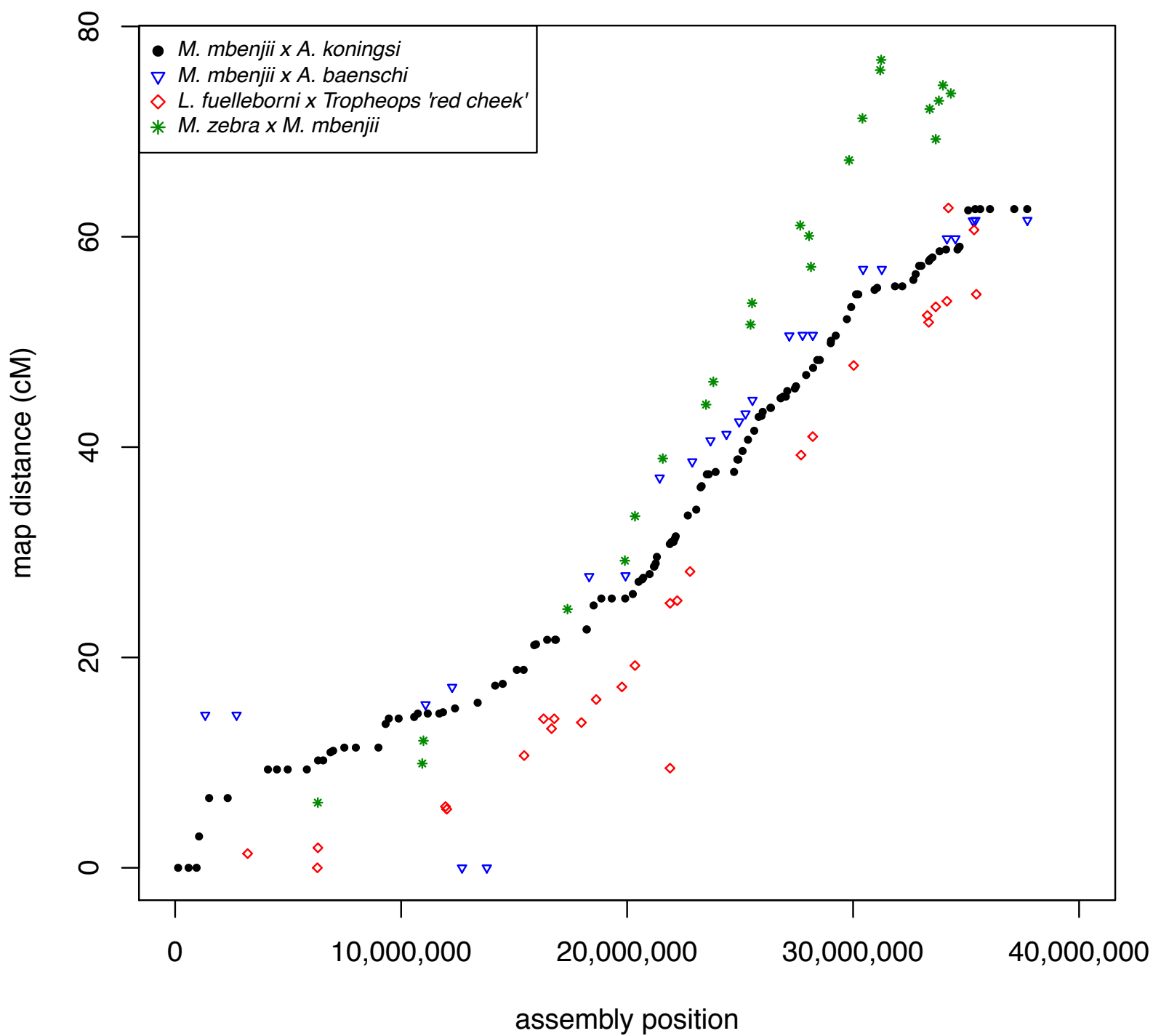

# Ig4

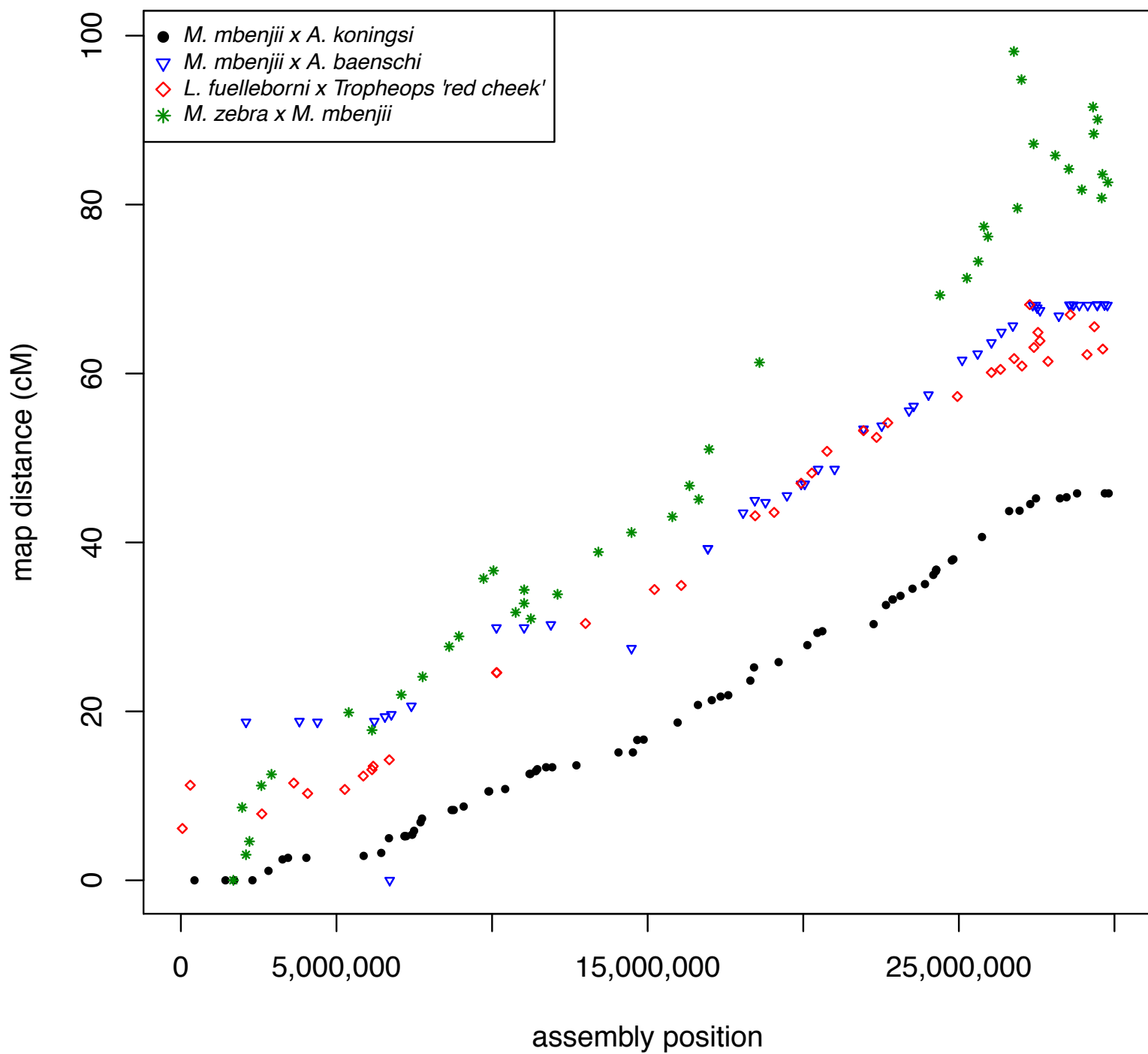

Ig5

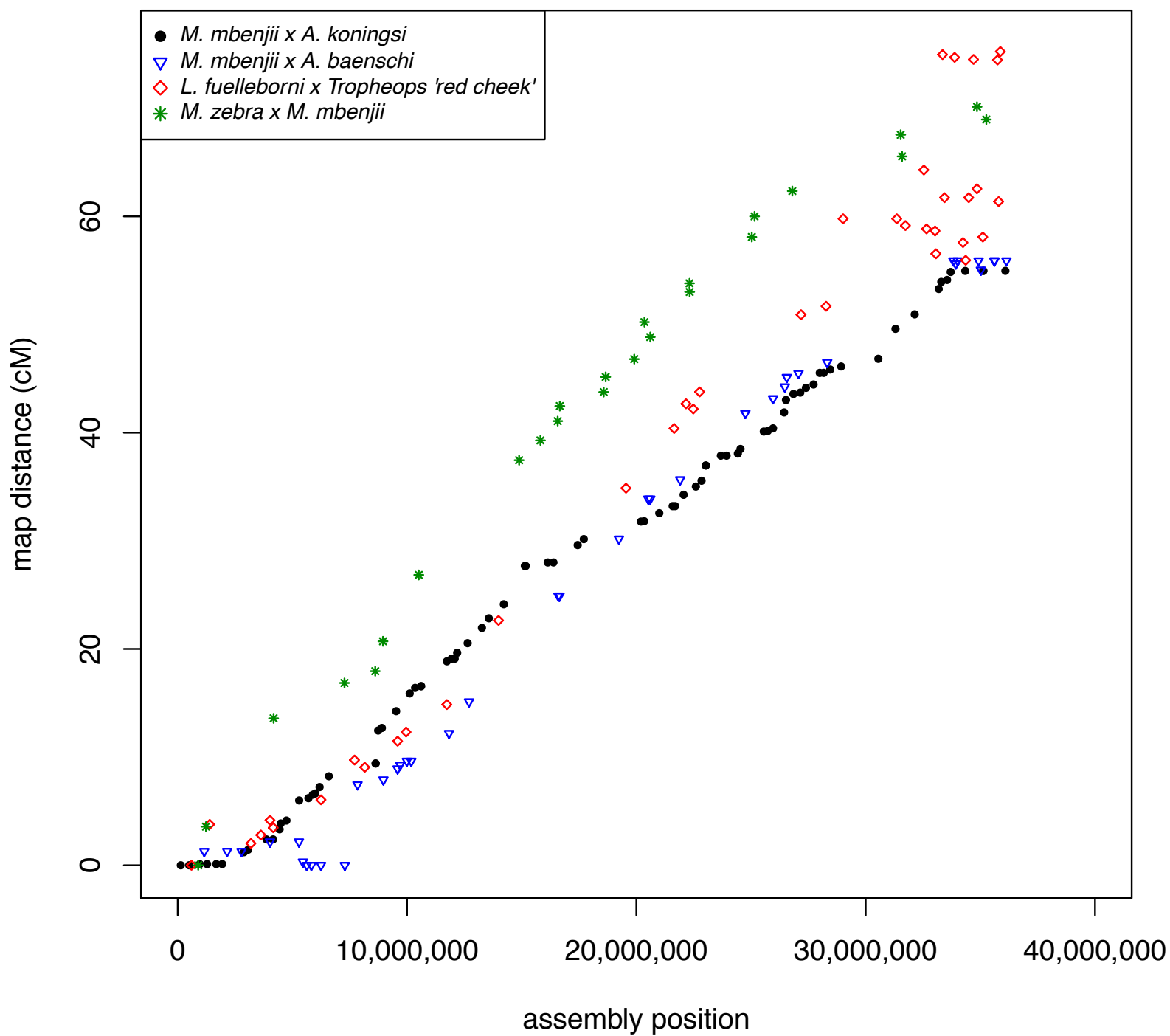

Ig6

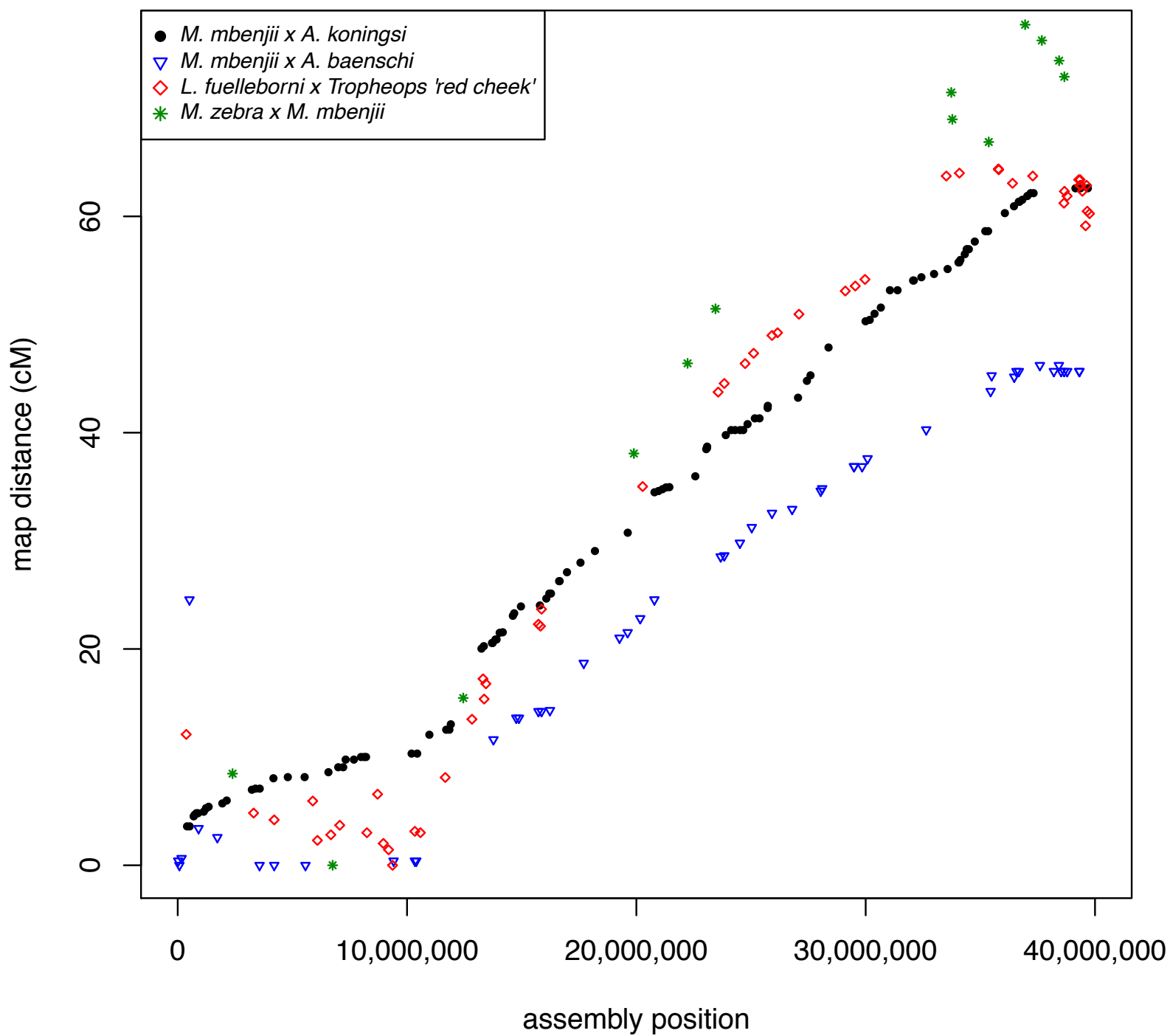

lg7

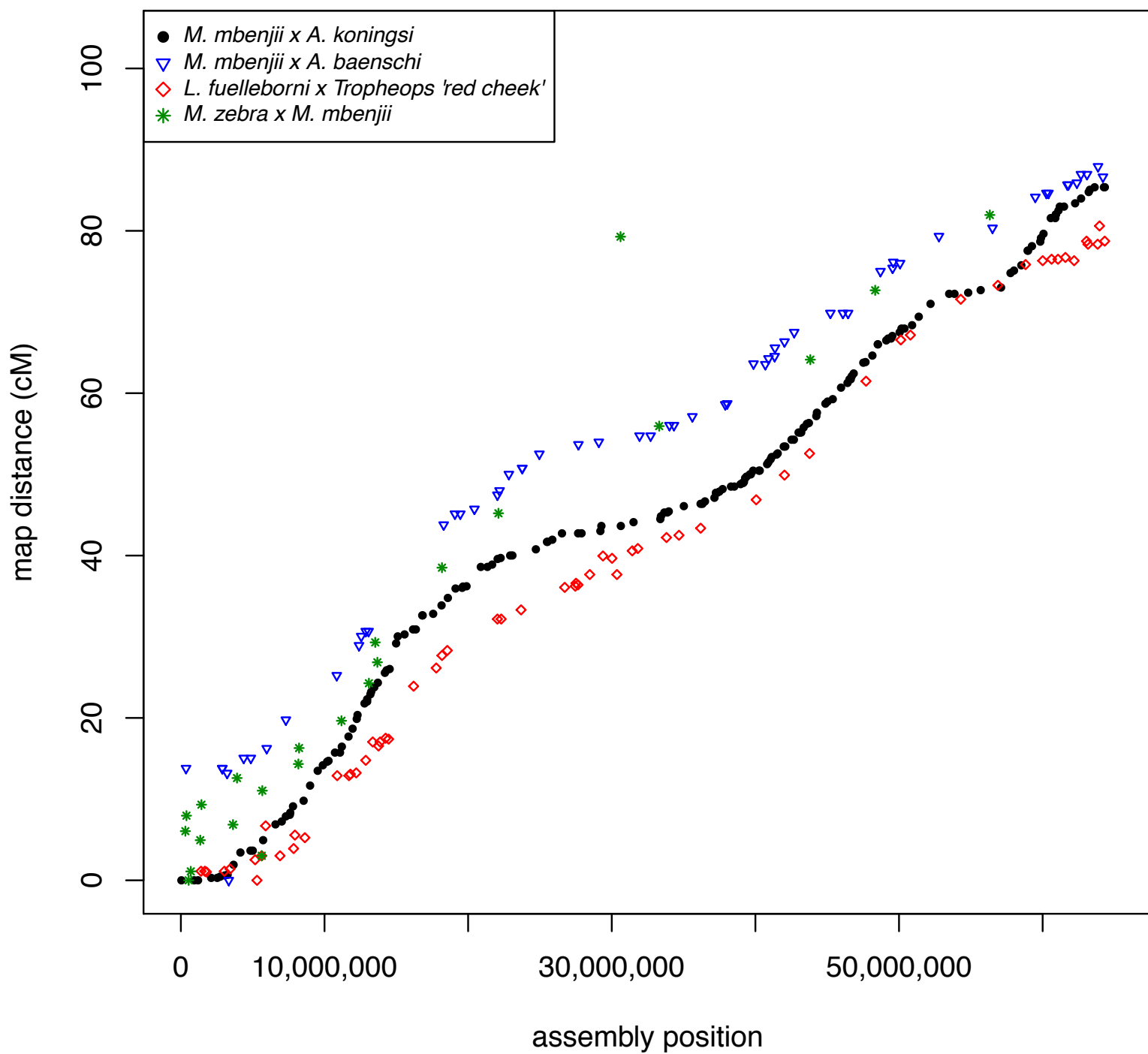

Ig8

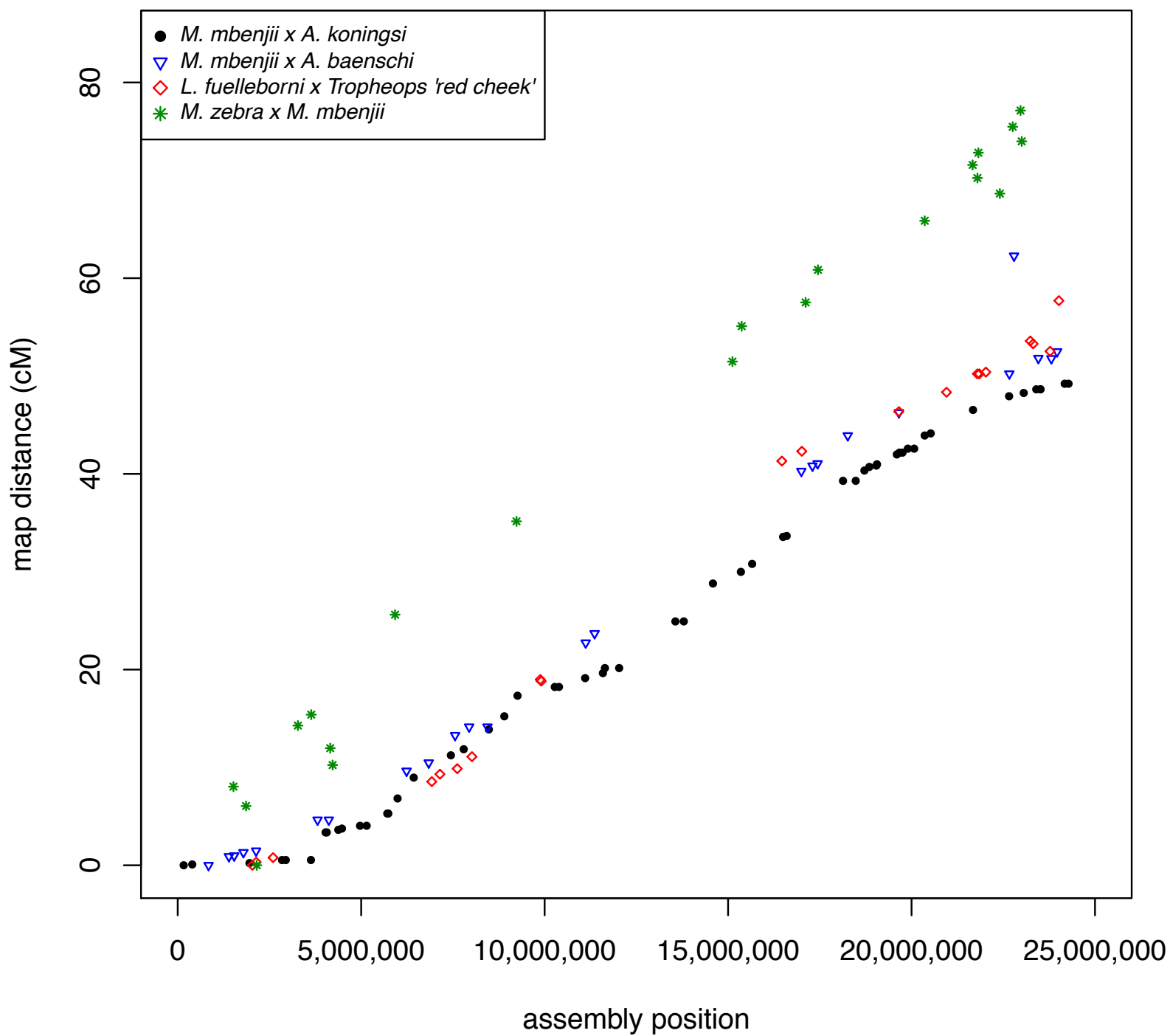

Ig9

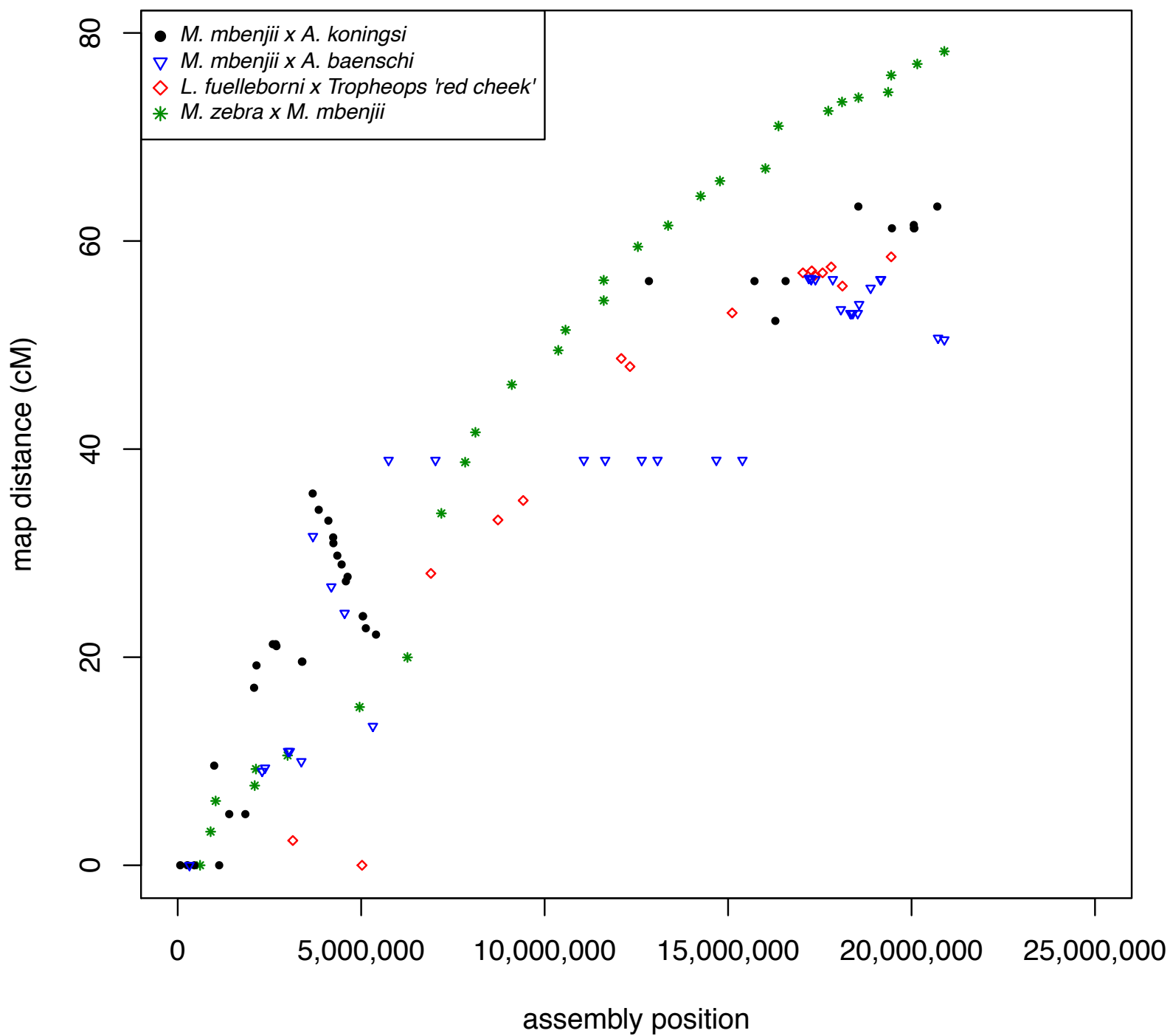

Ig10

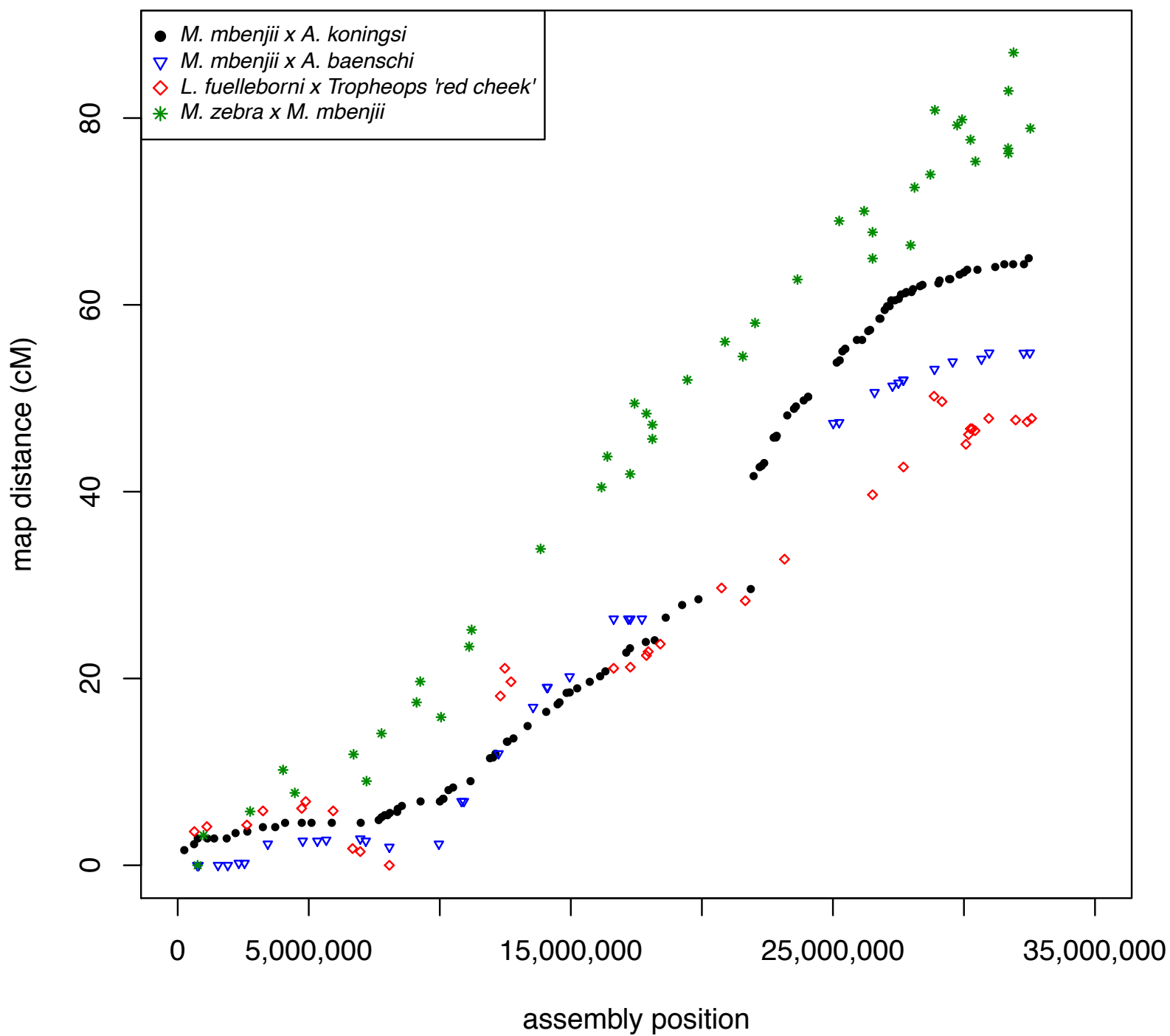

Ig11

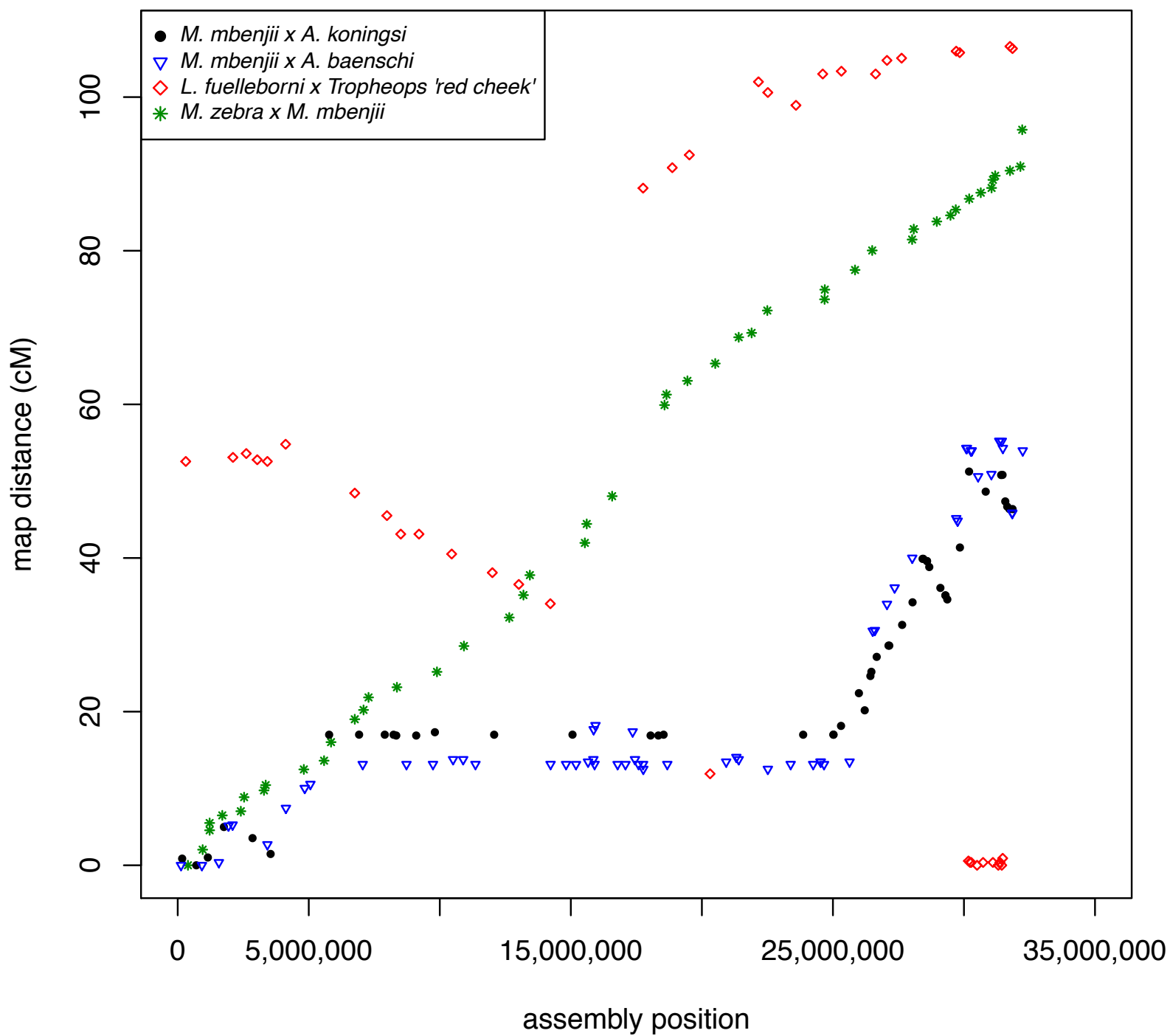

Ig12

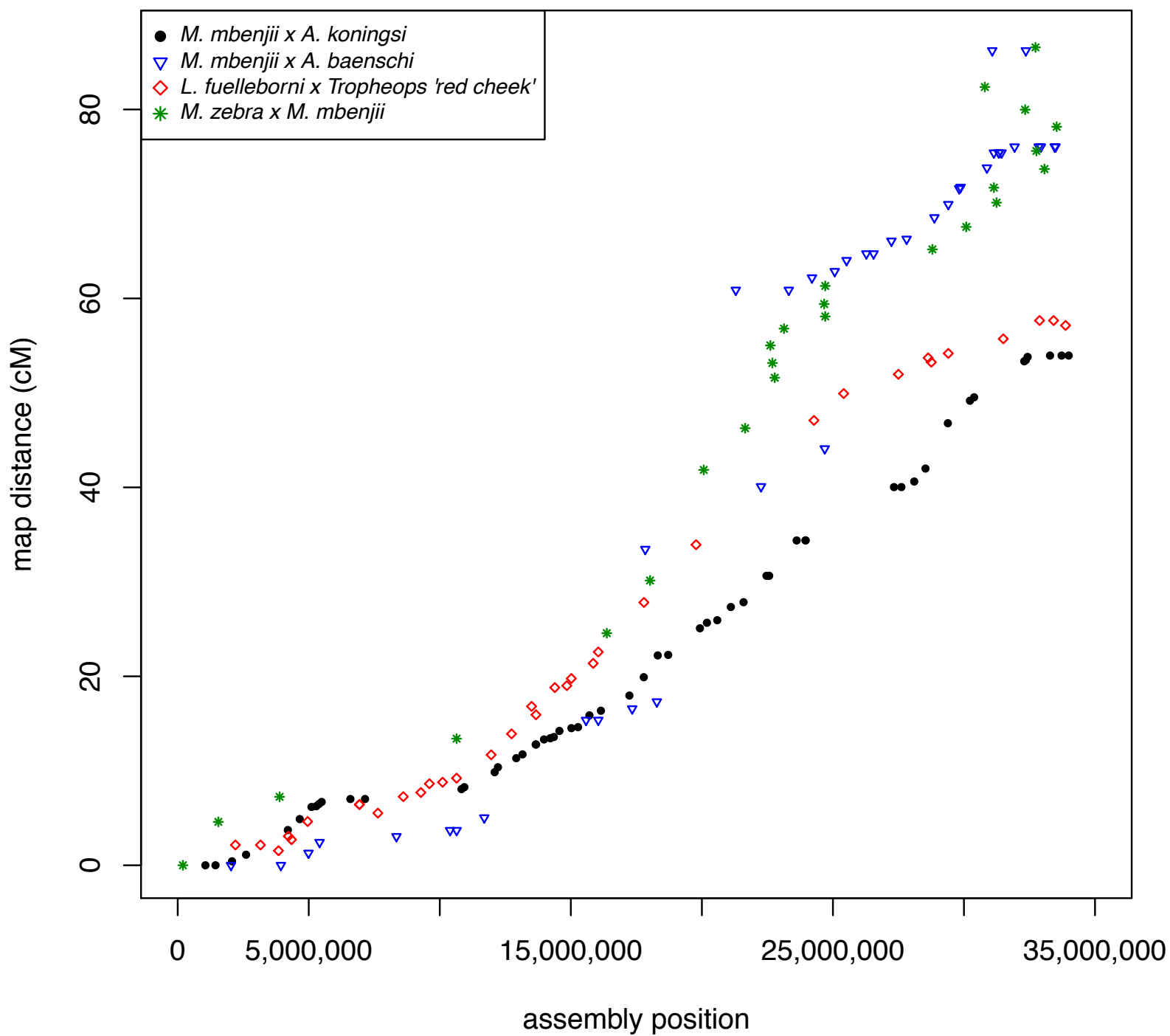

# Ig12

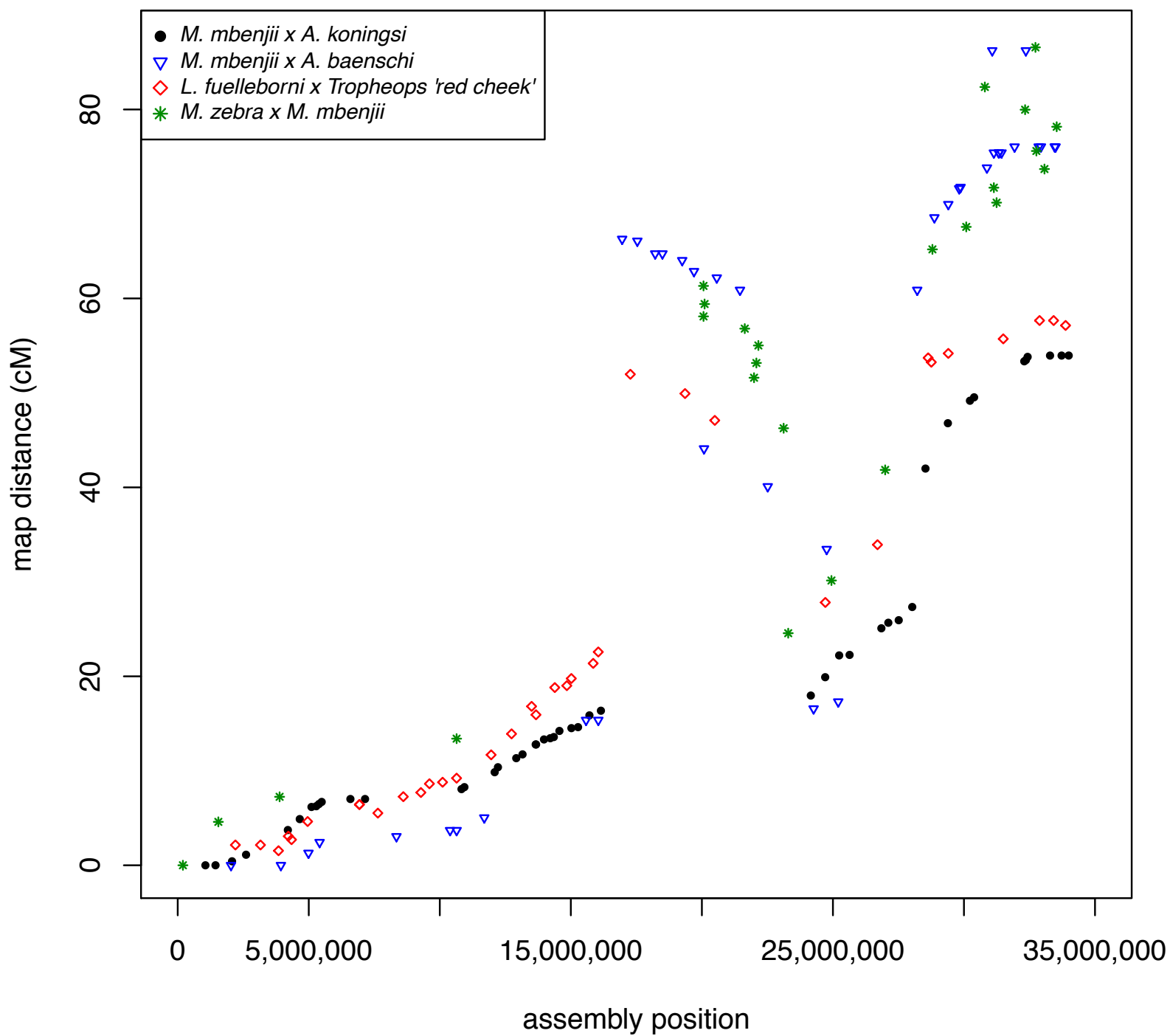

# Ig13

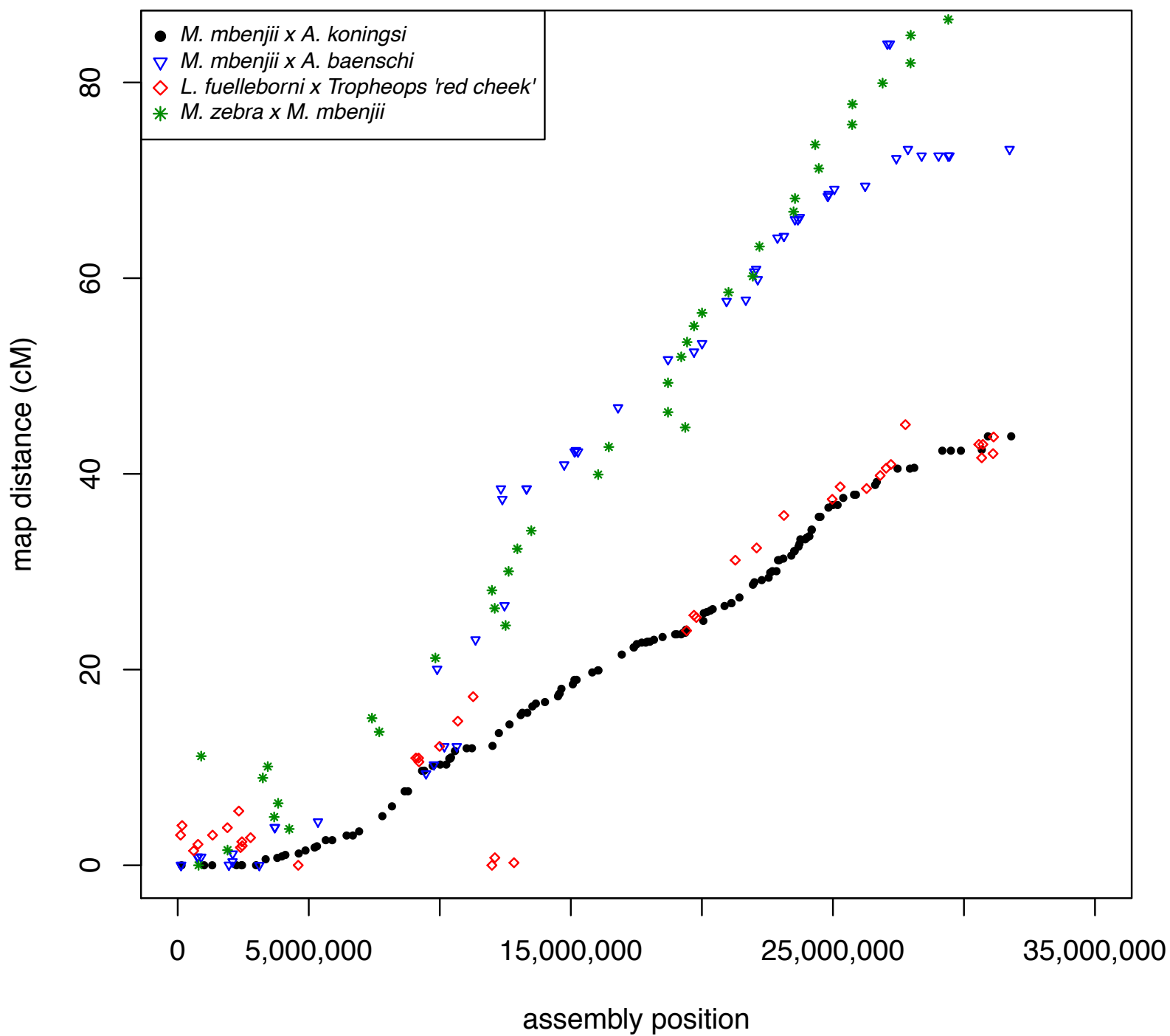

Ig14

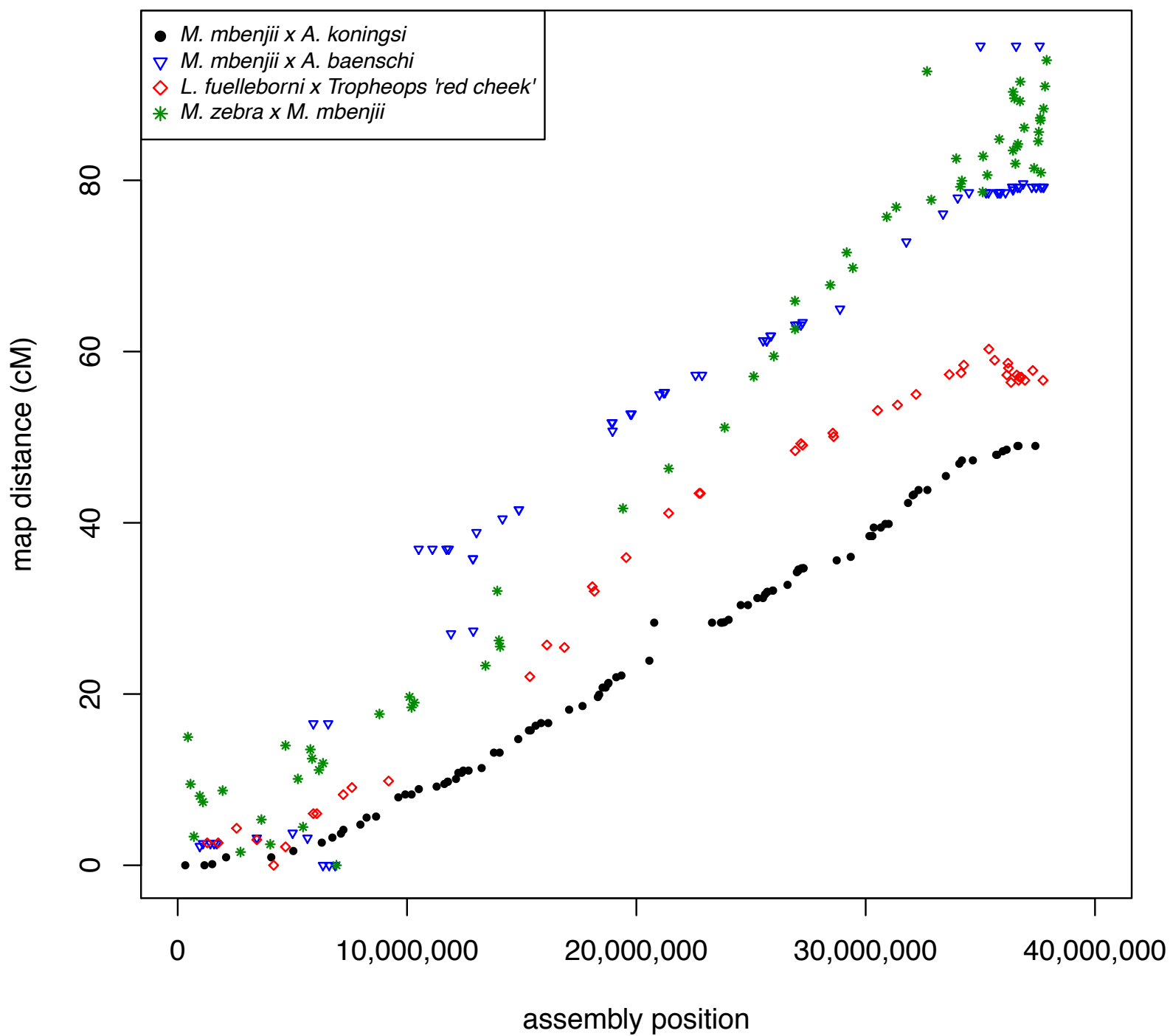

Ig15

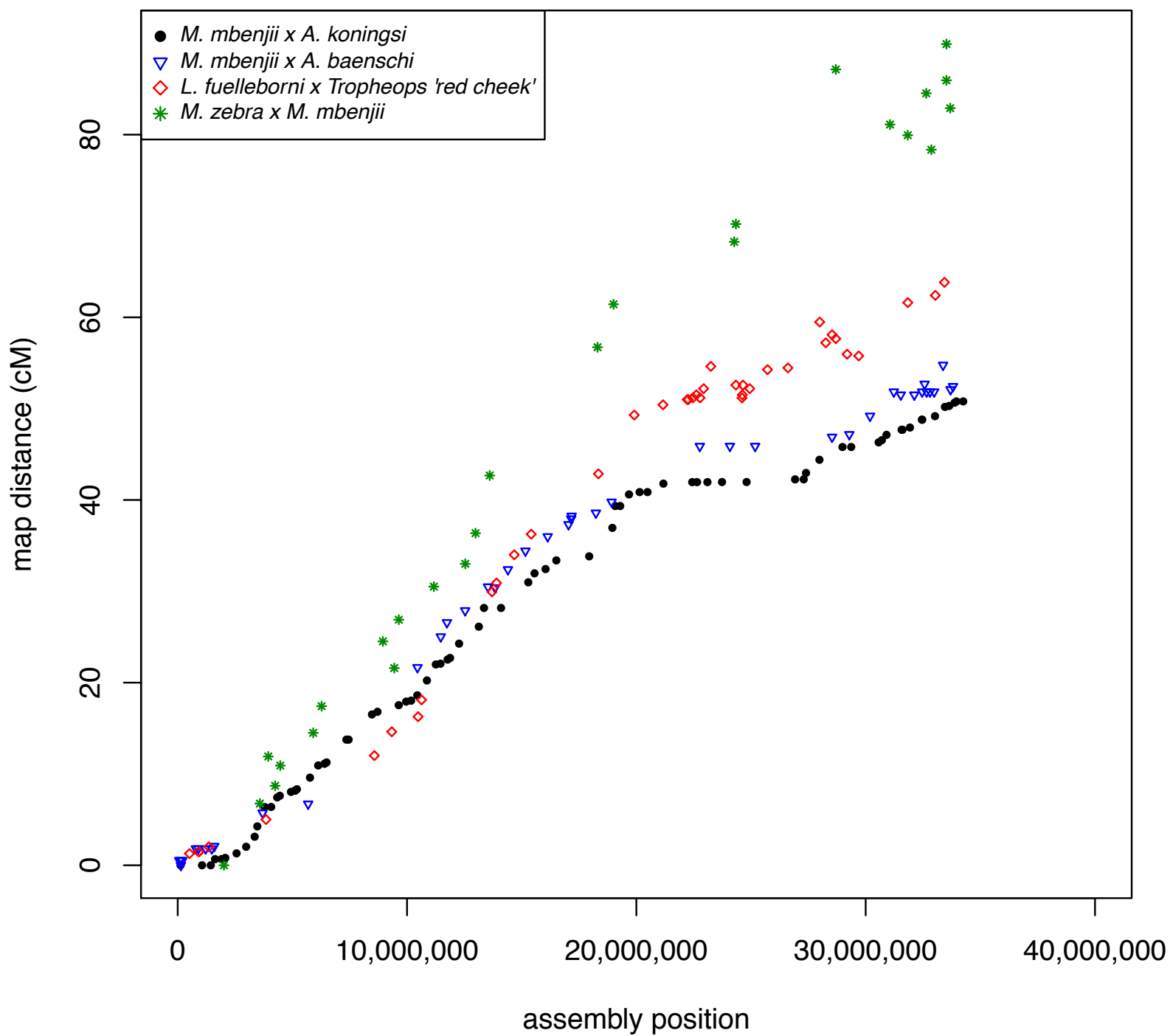

# Ig16

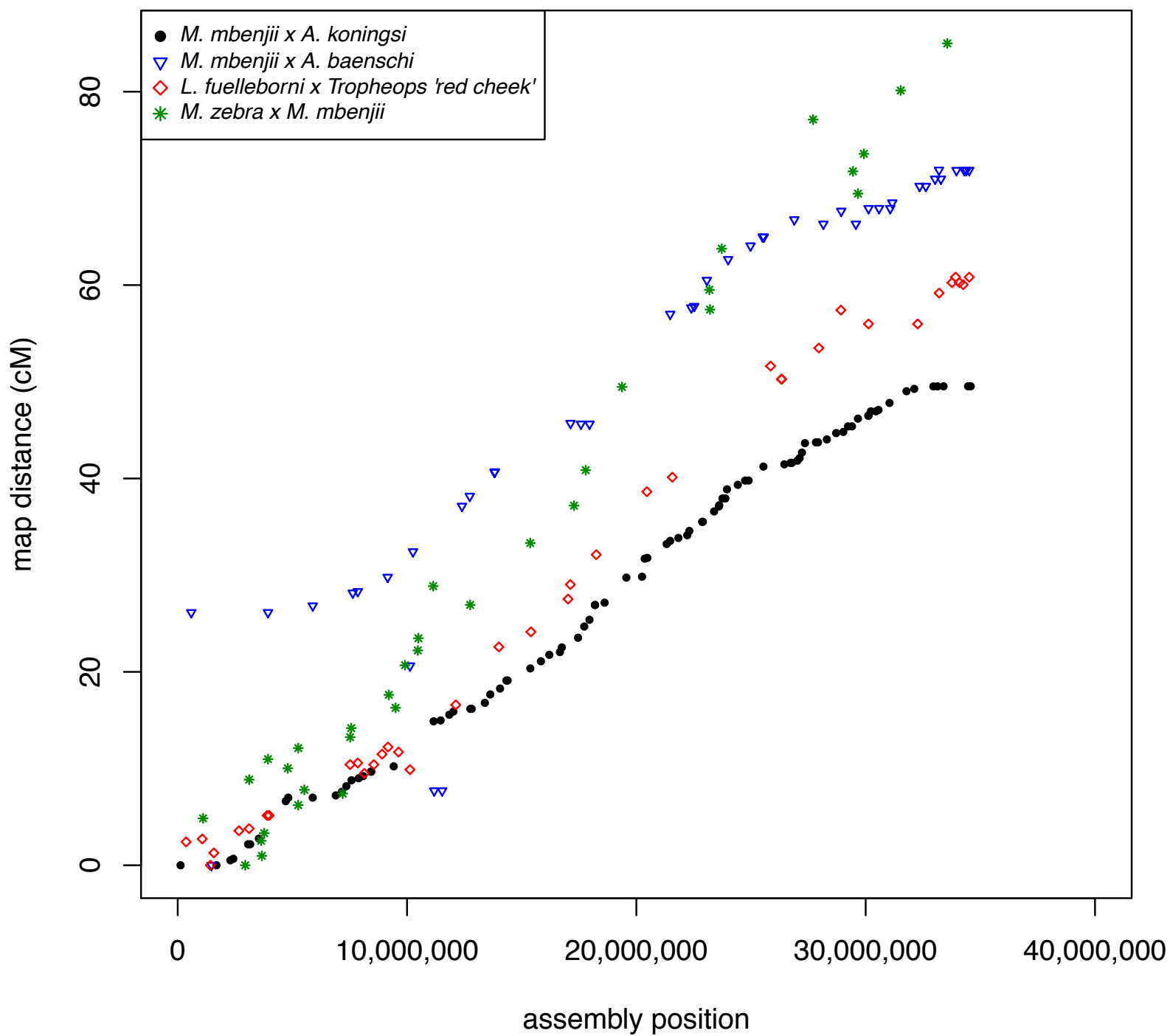

Ig17

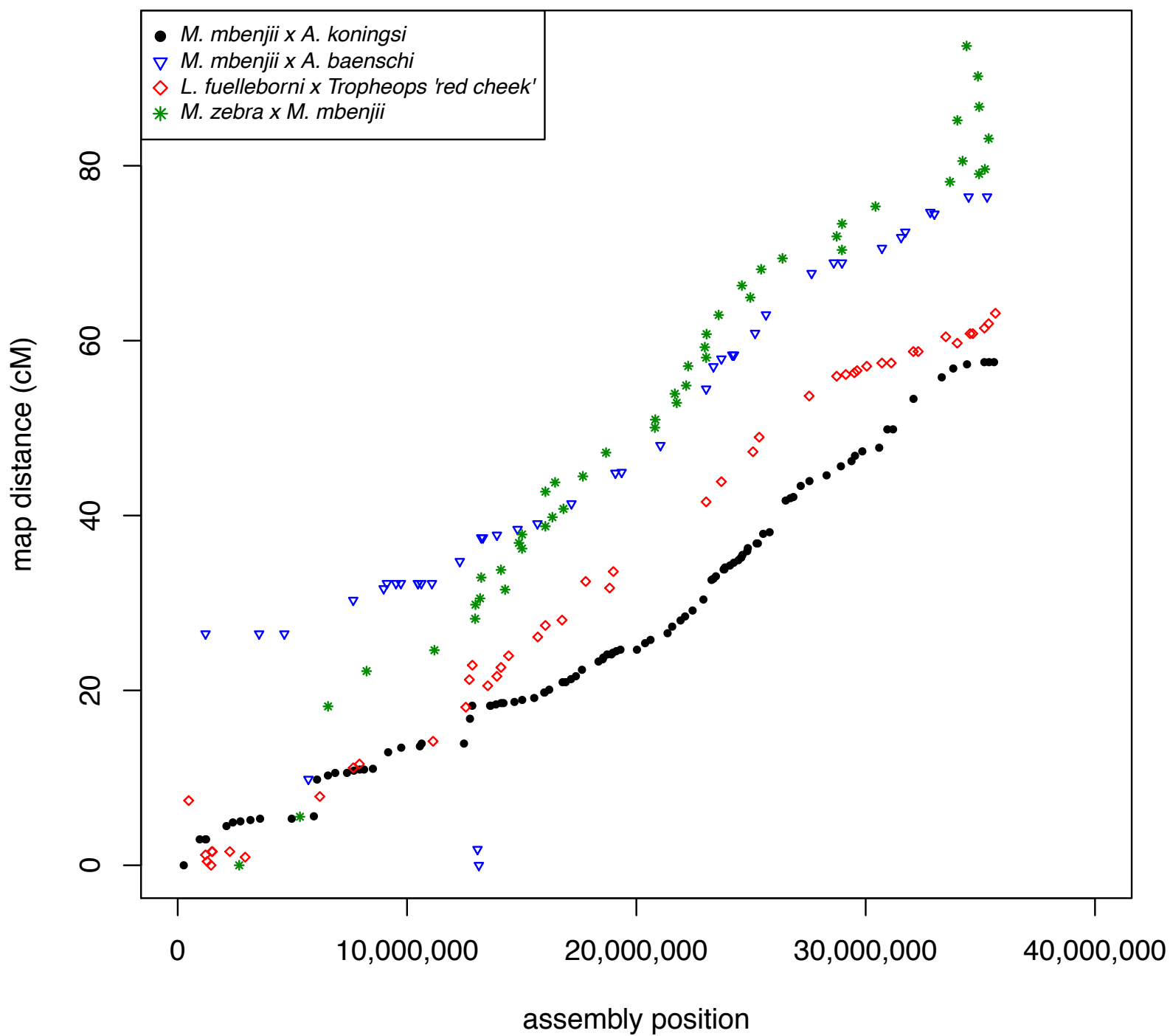

Ig18

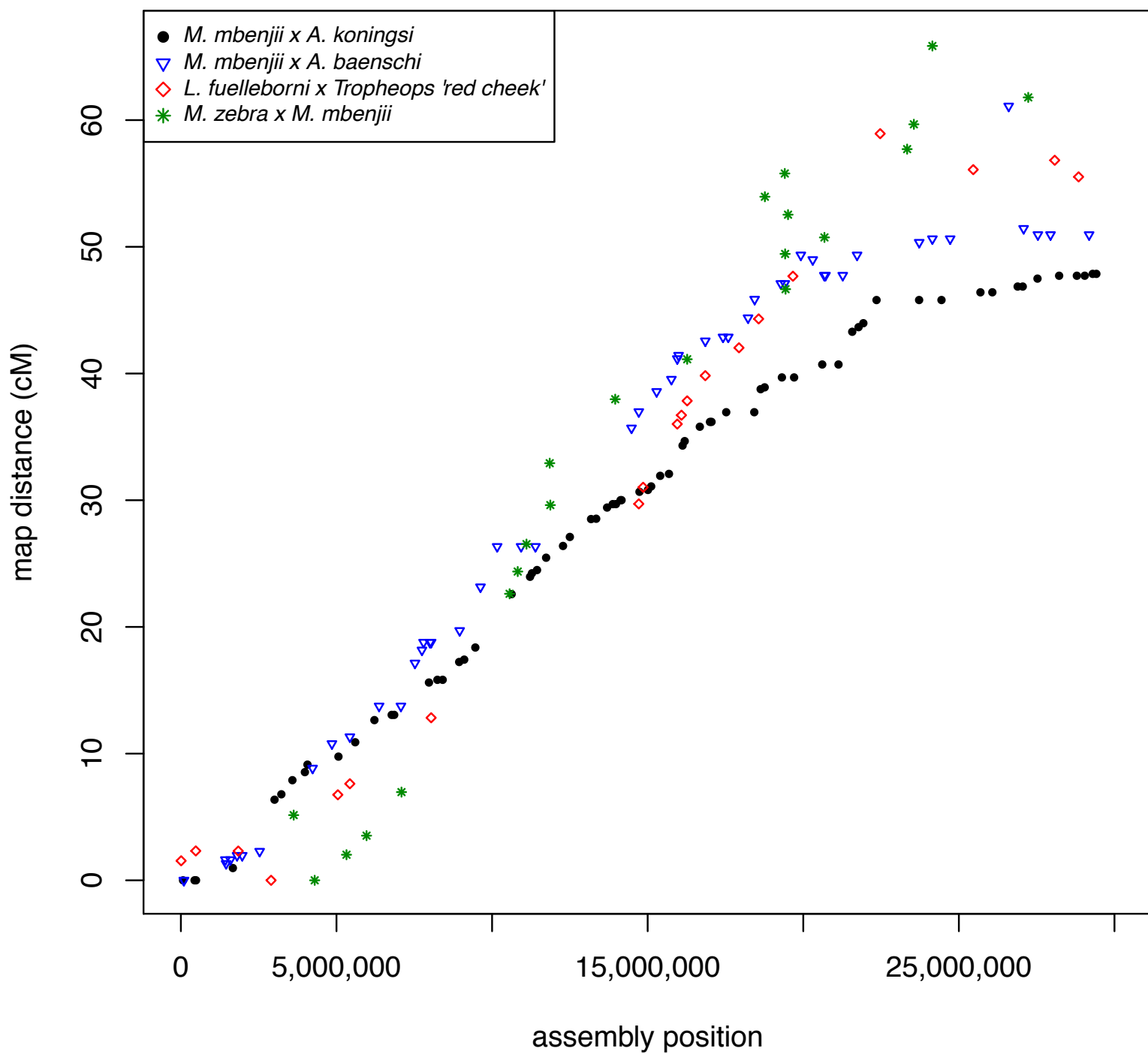

# Ig19

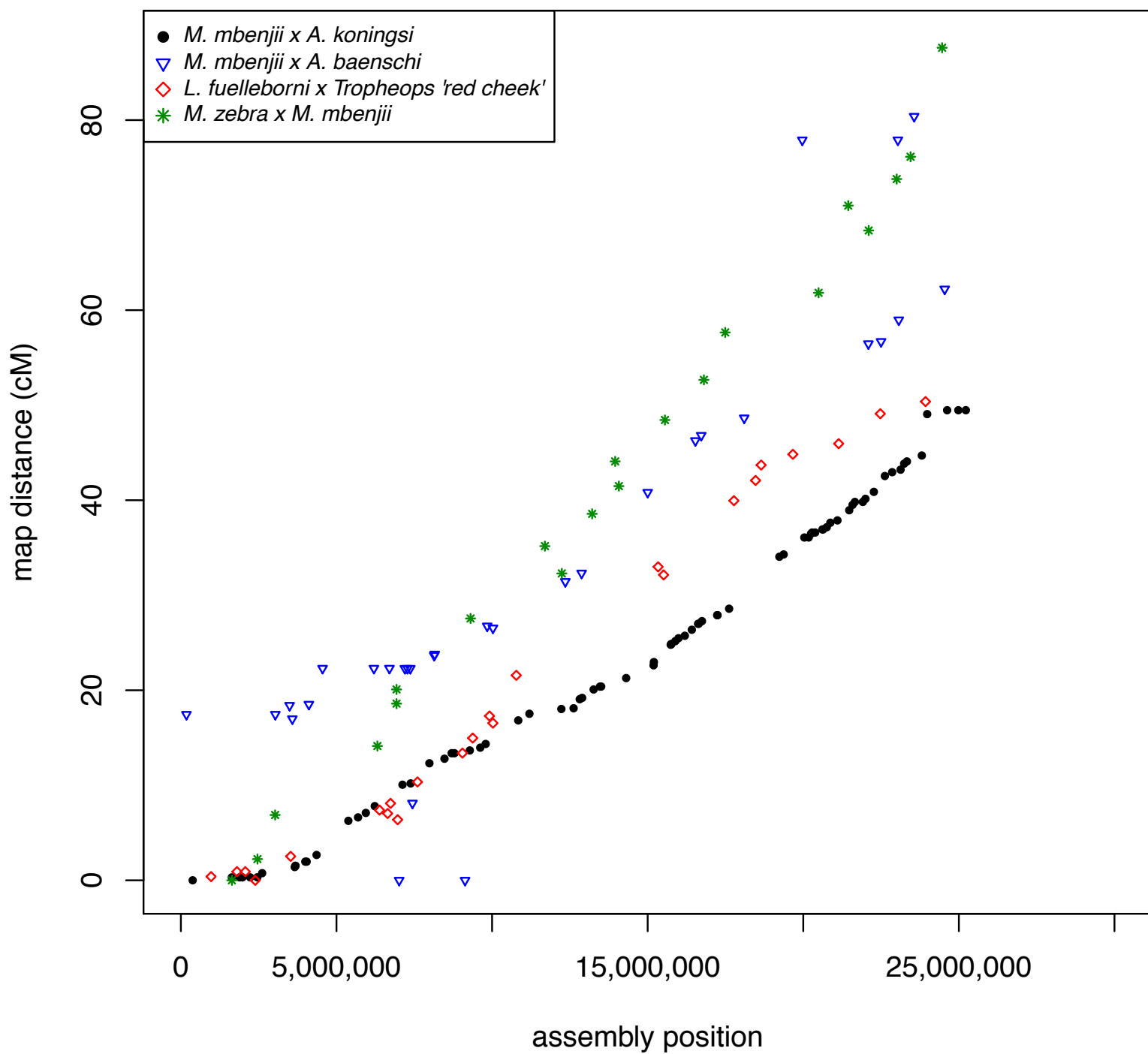

Ig20

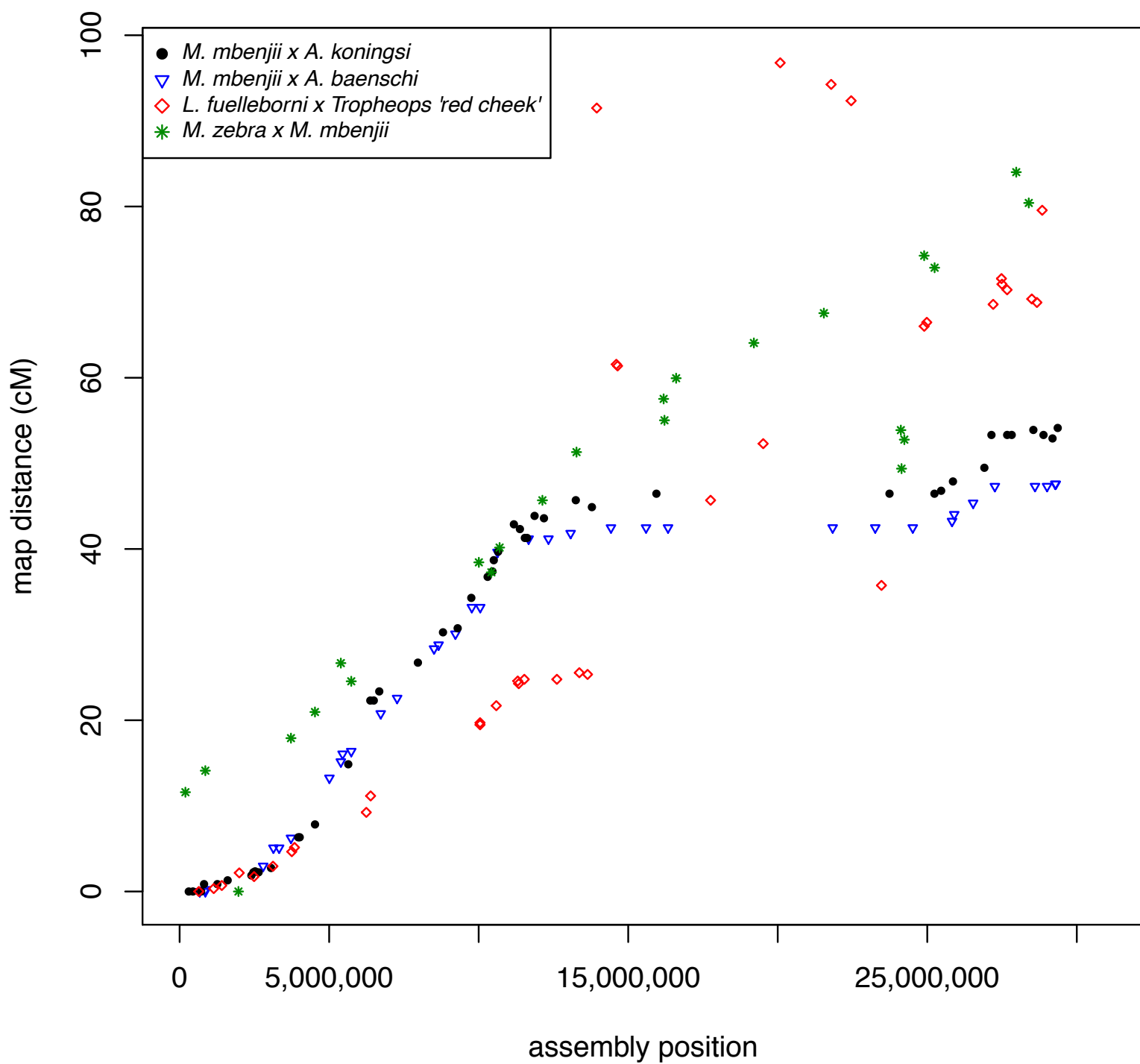

Ig22

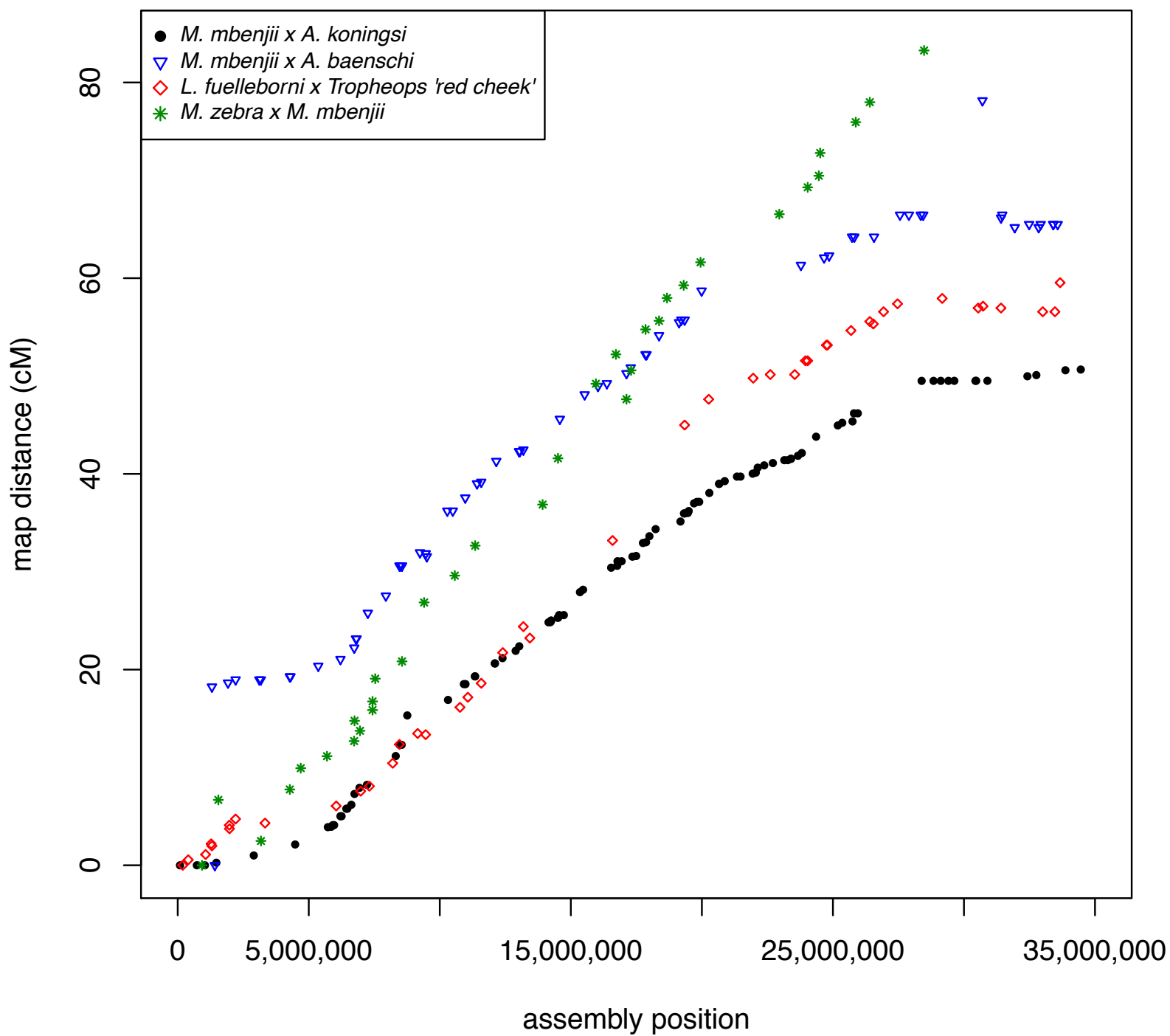

Ig23

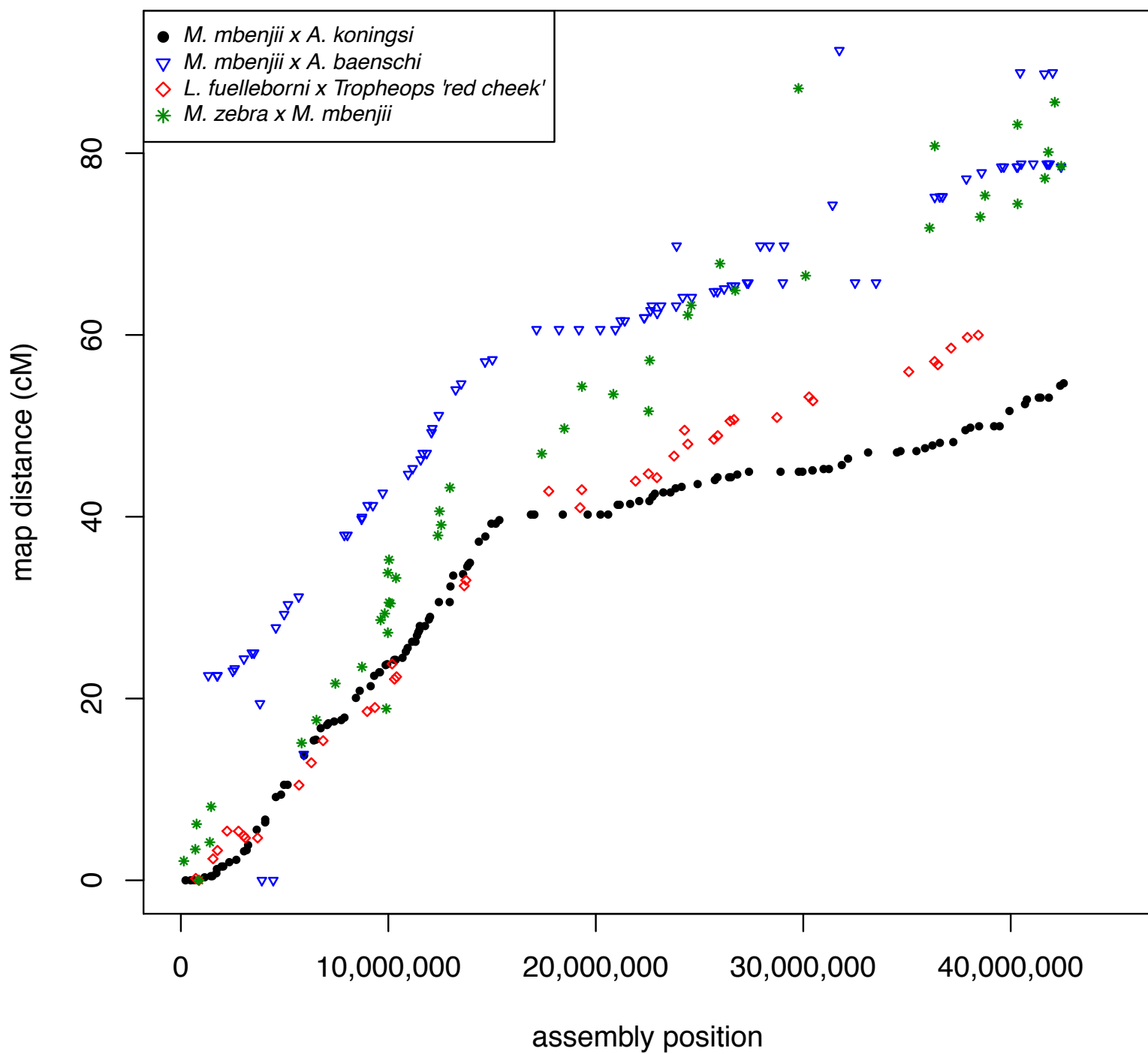

Supplement: Supplement_Files.zip [file giz030_supplement_files.zip › AdditionalFileG_Lake_Malawi_recombination_plots.pdf]

Read length distributions

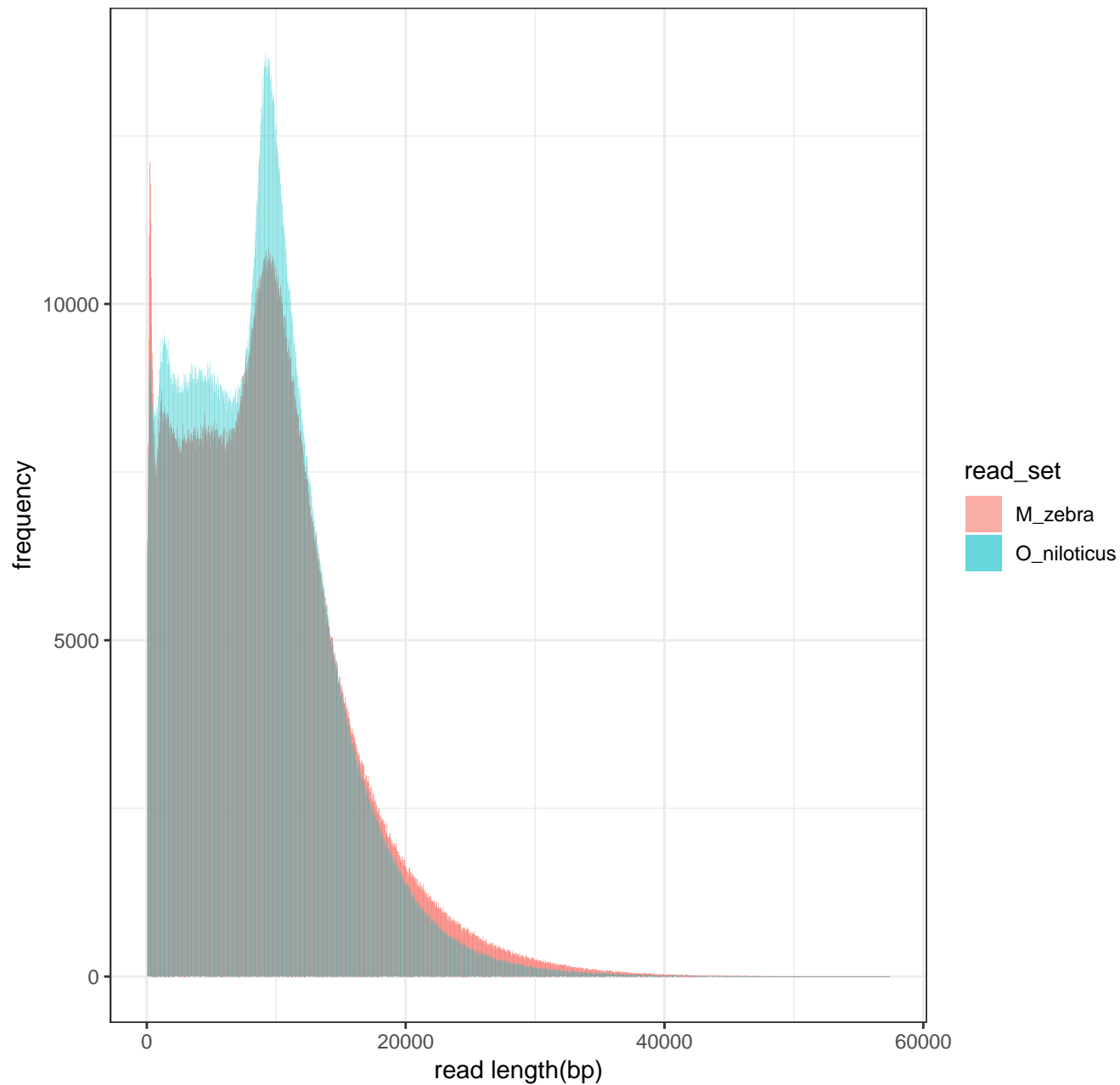

Supplement: Supplement_Files.zip [file giz030_supplement_files.zip › AdditionalFileH_44X_read_length_distribution_comparison.pdf]

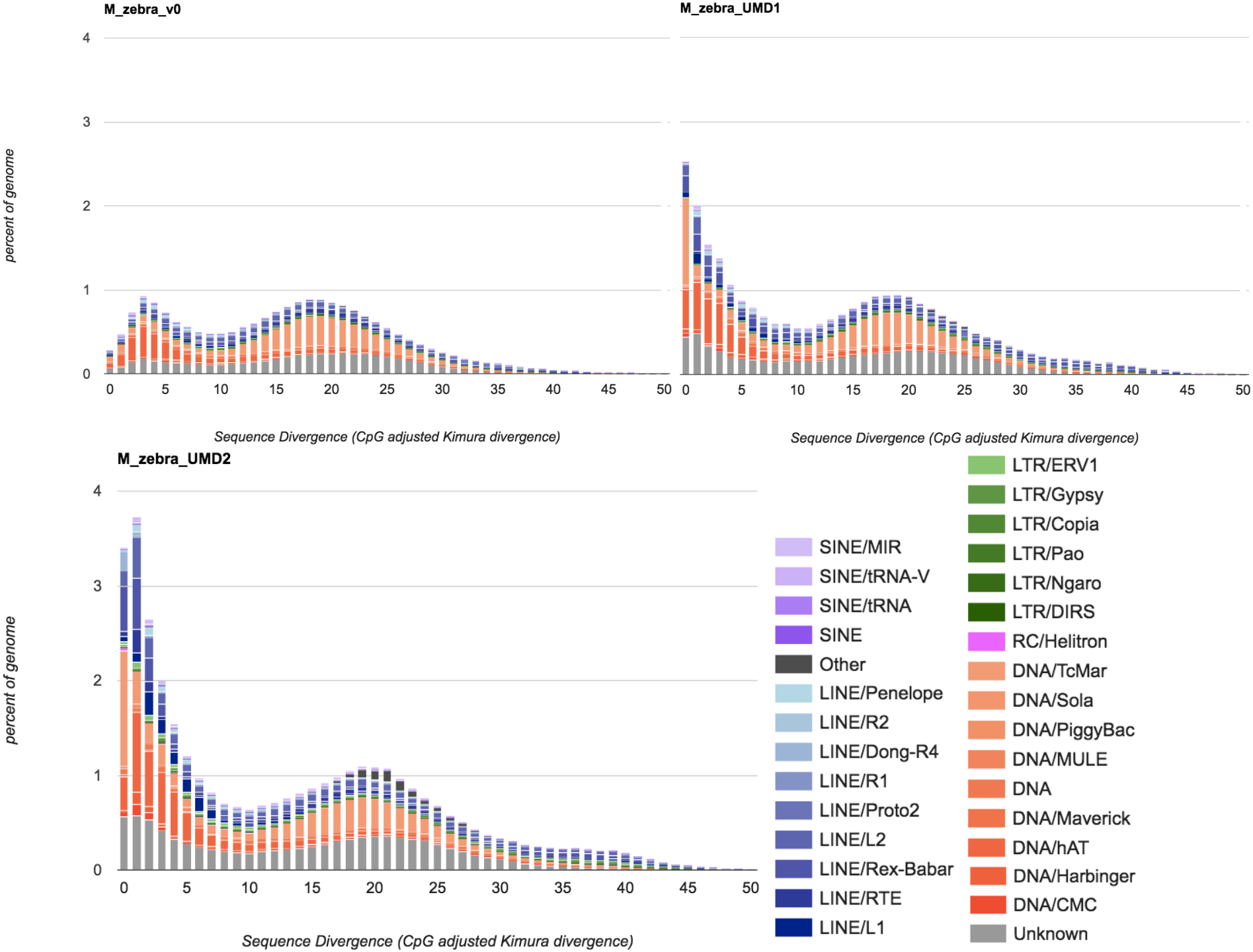

Supplement: Supplement_Files.zip [file giz030_supplement_files.zip › AdditionalFileK_TE_landscape_M_zebra_assemblies.pdf]
