# Supplementary material for: Chromosome-scale assemblies reveal the structural evolution of African cichlid genomes
Source: Gigascience. 2019 Apr 3;8(4):giz030. doi: 10.1093/gigascience/giz030 (PMC6447674; doi:10.1093/gigascience/giz030)
Supplement: Response-to-Reviewer-Comments-Revision-1.pdf [file giz030_response-to-reviewer-comments-revision-1.pdf]

Author's Response To Reviewer Comments

Close

We again thank the reviewers for their careful reviews and thoughtful feedback of our manuscript. We detail our response to each of their specific comments in the paragraphs below.

"Reviewer reports:
Reviewer #1: The authors have carefully revised their manuscript and have addressed most of the issues raised in my previous review (except two points, see below). They have also added a nice summary figure, which greatly helps to get an overview of the major rearrangements detected between the two genome assemblies. The authors have also removed the most obvious redundancies in the discussion section, which considerably improves readability of the paper. However, the discussion hasn't changed much in length and remains largely descriptive. I would have really liked to see a much more concise discussion of the biologically relevant findings, but I leave it up to the editor and authors to decide if the paper would benefit from further shortening.

We feel that we have sufficiently reduced the repetition and although the discussion is long, we feel that it is necessary to make all of the points we wish to make throughout this large study.

Remaining issues:

Page 11, line 42 - page 12, line 17, Additional File B: Unfortunately, the authors' response in the rebuttal letter doesn't help to clarify my previous issues with their approach to analyze the haplotig size distribution. In their rebuttal, the authors are now talking about the size distribution of homozygous regions in the diploid assembly and the expected size distribution of homozygous regions in an individual from a population of a given Ne. In the main text, the description of the figure in Additional File B is as follows:
"Additional File B shows the size distribution of the assembled haplotigs and how it relates to the theoretical recombination rate for several different effective population sizes (Ne)."
Not only is the description of the figure still lacking important details, they also describe the y-axis as the theoretical recombination rate, while the figure is showing E(r2), i.e. a measure of linkage disequilibrium. Apart from this confusing and contradicting description of what Additional Figure B is actually showing, I don't to see the theoretical foundation of this approach. The references provided in the main text don't really help here. As far as I can see, Additional File B compares to completely unrelated properties, i.e. the size distribution of haplotigs and the theoretical decay curves of LD versus physical distance for different effective population sizes (but these should depend on rho=Ne*r). To my knowledge, FALCON-unzip performs a purely physical phasing procedure. Therefore, the extent of LD in a population shouldn't be of any relevance for the extent of phased segments. What determines the sizes of resolved haplotypes blocks (i.e. the haplotig size
distribution) is the distribution of distances between heterozygous sites in the diploid genomes (which is determined by Ne, mutation and recombination rate, as well as the level of inbreeding) compared to the distribution of read lengths and sequencing coverage. Given the insufficient description and figure labelling, I might just not correctly understand the reasoning behind the approach taken by the authors here, but I would strongly suggest omitting this part and figure from the paper as it seems conceptually flawed."

This is a very useful point and we now agree with the reviewer. We have removed this small section and figure from the manuscript accordingly.

"Page 40, line 7-9: "We are unsure what the reviewer means, as one of the many things that PLINK can do is report LD statistics as described."
I was specifically referring to the second part of the sentence: "using the pedigree described above and SNP positions given in [103]." I assume that the SNP positions were not obtained from the paper describing the PLINK software."

We have changed this.

Close
