# Supplementary material for: Chromosome-scale assemblies reveal the structural evolution of African cichlid genomes
Source: Gigascience. 2019 Apr 3;8(4):giz030. doi: 10.1093/gigascience/giz030 (PMC6447674; doi:10.1093/gigascience/giz030)
Supplement: Response-to-Reviewer-Comments_Original-Submission.pdf [file giz030_response-to-reviewer-comments_original-submission.pdf]

Author's Response To Reviewer Comments

Close

We thank both reviewers for their careful reading of our manuscript. We have worked to significantly shorten and tighten the text. We detail our response to each of their specific comments in the paragraphs below.

Reviewer #1:
This paper by Conte and colleagues describes two new chromosome-scale genomic assemblies of African cichlids. The authors used multiple genetic maps to anchor contigs from high-coverage PacBio sequencing and correct misassemblies. Based on these two high-quality genomes and the genetic maps, the authors performed comprehensive comparative analyses of recombination landscapes, large-scale chromosomal rearrangements, and transposable element insertions. The paper presents extensive genomic resources, which will be valuable for future studies in the field.
However, the manuscript in its current form is highly descriptive and many parts of the paper are repetitive and very tedious to read. I'm convinced that the appeal of the paper for a broader readership could be improved considerably by shortening the main text and putting a focus on the biologically interesting aspects. The purely descriptive details could be presented more effectively in tables and figures or additional supporting materials. For instance, instead of the lengthy description of rearrangements, regions of unusual recombination, and putative sex determination loci, I would like to see a carefully designed summary figure, which provides the reader with a good overview of these events in the two genomes.
In its current form, many interesting aspects are buried in large amounts of text that provide information of little biological relevance. Most importantly, the discussion part should be written much more concisely, as it still largely descriptive and repeats most of the information that was already provided in the results section. Here, the authors should refrain from discussing every single aspect of their results and rather focus on the biological interpretation of the most interesting findings of the study.

Minor comments:
Page 4, line 15: Define "indel" here.
We have added this definition.

Page 4, line 52: Provide reference for PacBio sequencing.
We have added this reference.

Page 10, Table 1: Improve the labelling of the table. It is not immediately clear that the numbers represent base pairs.
We have improved the labeling of this table.

Page 11, line 42: "relatively complete" compared to what?
We have edited this sentence to clarify.

Page 11, line 35: The description here seems to imply that the final p-contigs are not phased. The p-contigs are phased within the borders of their associated haplotigs. Please provide a more detailed explanation of p-contigs and haplotigs here.
We have modified this sentence to remove the wrong implication and have added a more detailed explanation of p-contigs and haplotigs.

Page 11, lines 42-55: This part is unclear and should be rewritten. What are "theoretical sizes of heterozygous regions"? Do you mean the theoretical expectation of the distribution of distances between heterozygous sites? The associated Additional File A is completely unclear and needs a much more detailed explanation and legend, e.g. what is the relationship between the two x-axes (length in base pairs and recombination rate) or the two y-axes (frequency and E(r2))? How do you derive information about the completeness of haplotigs from this graph?

The axes of File A were poorly labelled, and our conclusions a little overextended. We have revised the text, figure and legend to improve the presentation of these results. The main point is that the size distribution of the homozygous regions in the genome assembly closely matches the expected size distribution of homozygous regions in an individual drawn from a population with an Ne of 1,000 – 2,000 individuals. We no longer make any claims about the completeness of the genome assembly from this graph.

Page 12, line 55: Provide full genus name for A. koningsi.
We have added this.

Page 13, Table 3: Why does the total length differ for the four different maps, given that it includes both anchored and unanchored contigs?
We had added a sentence that clarifies this.

Page 15, lines 34-42: Given that the anchoring is based on a combination of four different maps, is it possible that certain contigs are represented multiple times in the final assembly?
We have added a sentence that addresses this question.

Page 16, lines 4-47: Given that all genetic maps are from inter-species crosses, what are the expectations for inter-chromosomal rearrangements that are only present in one of the two species? It seems unlikely, that the given approach would have power to detect rearrangements in such cases.
The reviewer is correct that we would be unlikely to detect inter-chromosomal rearrangements in interspecific crosses among Lake Malawi species. Recombination in such regions in hybrids would likely produce inviable gametes or offspring, effectively suppressing recombination. So, such rearrangements would produce only the weak signal of reduced recombination through these regions, which we would not likely detect.

Page 16 line 34: "… at most 1% of these Lake Malawi genomes is affected by inter-chromosomal rearrangements …"
We have deleted this sentence. While we believe it is likely true, based on the limited amount of inter-chromosomal rearrangement between tilapia and Metriaclima, we currently do not have the data to support this statement.

Page 23, lines 46-48: This sentence doesn't make sense without a distance qualifier, i.e. significant linkage disequilibrium over extended physical distances.
We have added this qualifier.

Page 26, line 59: "Only one contig longer than 1Mbp was not anchored …"
We have modified this sentence.

Page 27, line 7: "Contigs in the M_zebra_UMD2 assembly were primarily anchored with" or "The M_zebra_UMD2 anchoring was primarily performed with"
We have changed this sentence.

Page 27, lines 46-48: The suggested link between TEs and chromosomal rearrangements seems a bit far-fetched. It appears more likely to me that low recombination is facilitating the enrichment of both TEs and rearrangements due to reduced Ne and therefore reduced efficacy of selection against slightly deleterious events in these regions.
We agree that there is an enrichment of TEs and rearrangements in regions of low recombination due to reduced efficacy of selection against slightly deleterious events in these regions and had already included a citation supporting that idea (Dolgin et al 2008). However, the link between TEs and chromosomal rearrangements is not far-fetched, and many studies have demonstrated this. We have added a sentence with several citations to support our claim.

Page 27, line 56: Not clear what is meant by "orthogonal mapping technologies" here. Alternative mapping technologies?
We have clarified this sentence.

Page 28, line 51: Genetic differentiation between what?
We have added to this sentence to be more explicit.

Page 30, line 33: Linkage group information is missing for the sex determination locus.

We have added this.

Page 30, lines 45-49: Rather the alleles of the sex determination system segregate in three crosses.
We have modified this sentence.

Page 32, lines 36-44: The connection between lack of evidence for a chromosome fusion event on LG3 and the accumulation of repetitive elements is not clear.
We have added some text to clarify this connection.

Page 34, line 29: What are "centromere-containing repeats" and does this refer to the ONSATA and TZSAT satellite sequences in the next sentence? Please rephrase this part.
We have rearranged and modified a sentence to make this clear.

Page 37, line 38: "will be able to purge"
This has been incorporated.

Page 40, line 9: Incomplete sentence
This sentence has been modified.

Page 40, line 29: Omit "that"
This sentence has been modified.

Page 40, line 44: Check reference. Reference to PLINK software doesn't make sense in this context.
We are unsure what the reviewer means, as one of the many things that PLINK can do is report LD statistics as described.

Reviewer #2:

General comments:
With their high speciation rates and remarkable phenotypic diversity, African cichlid fishes serve as a model for studying a broad range of evolutionary processes. In this manuscript, the authors use two high-quality genome assemblies and five genetic maps to investigate large scale structural variation, changes in broad-scale patterns of recombination, and to compare transposable element contents of the two assemblies. I think that the work is generally well executed and provides valuable new insights. The manuscript is also clearly written and the arguments are easy to follow. My only critical comment related to the current text is its repetitiveness, with the same statements often occurring in several places (see examples below). Especially the discussion is very long and, even though it offers some additional interpretation and explanations, it reads largely as a restatement of the analyses and the results.

Overall, I congratulate the authors on their work. I have some (relatively minor) comments and suggestions that the authors should consider before publication.

Specific comments/suggestions:
Data Description - this could be expanded, especially considering the GigaScience journal's focus. For example, for the new PacBio reads, I would be interested to see the read length distribution which is a crucial factor in genome assembly, especially influencing its contiguity and the types of repetitive elements that can be assembled. Perhaps the two "new genetic maps" should also have a brief description here? I don't know what the GigaScience journal policy is, but this work is not fully reproducible until the "new genetic maps" are published. I highlighted this in the comments to the Editor.

We have added a figure of the read length distribution as well as several statistics that provides more information when evaluating the genome assembly as the reviewer suggests. We have also added a brief description of the two new genetic maps. The new genetic maps themselves have already been uploaded to the GigaScience FTP to make the work reproducible.

Analyses
Page 10 - "The anchored assembly of LG3 is 54.7% repetitive, compared to repeat rate of 37% genome-wide" Perhaps a clarification of what is meant "repetitive" would be useful. Do the numbers quoted by the authors refer to the portions of the chromosomes masked by the RepeatMasker software?
We have modified this sentence to clarify this point.

Page 11 -"To measure the completeness of the haplotigs, the theoretical sizes of heterozygous regions under null expectations of recombination rates and effective population sizes were compared to the size distribution of the haplotigs. Additional File A shows the..." I don't understand this analysis and the associated "Additional File A". What exactly are the "haplotigs"? What determines their boundaries? Perhaps the authors should elaborate, and/or at the very least provide a reference to the theory that predicts how large the haplotigs should be.
See response to Reviewer #1 above. The theory on this point is explained on page 540 (Figure 9.8) of Hedrick's "Genetics of Populations" textbook (4th edition). The theory is attributed to Hill and Robertson 1968 (TAG 38:226-231) and Ohta and Kimura 1969 (Genet. Res. 13:47).

Page 24 - "...on the same compute cluster..."
I am not sure how the identity of the compute cluster could affect genome assembly. Read length and quality are two more likely factors that are not mentioned.
We have added an additional file that provides a comparison of the read length distributions of both datasets used in this particular analysis as well as some additional sentences to explain our inferences based on this comment.

Page 24 - "M. zebra genome assembly has a noticeably larger amount of recent TE insertions." Would be interesting to know where these insertions localised. For example, how many of these localised in gene promoters.
We have performed this requested analysis and provided the results as an additional file as well as providing additional text in the results and discussion.

Discussion
Page 27 - "optical mapping, may be needed to resolve the structure of these regions in finer detail." Again, perhaps longer reads that can span the repeats would help?
We have modified this sentence to include longer reads as a possibility.

Page 29 - "...suggesting the inversion may represent an evolved difference between the Metriaclima and Aulonocara lineages." I am not sure what exactly is meant by "an evolved difference". Isn't any difference between the lineages evolved? I suggest rephrasing this...
We have reworded this.

Page 30 - "...suggesting that the rearrangement occurred in the Lake Malawi ancestor and has maintained reduced recombination in this region across all lineages."
The Malawi species used for the maps represent only one genus of mbuna and Aulonocara - a small subset of Lake Malawi lineages. So I suggest rephrasing this, instead of "Lake Malawi ancestor" something like "ancestor of the Lake Malawi species used in our crosses".
This sentence has been removed in our efforts to remove some of the repetitive regions from our manuscript as the reviewer suggests below.

Page 30 - "...has an XY sex determination locus on [76]"
I think there is a word missing at the end of the sentence.
We have added this.

Some examples of repetitiveness in the current text:
* on page 10 we learn that LG3 is "the largest and most repetitive chromosome in O. niloticus [15], and is a sex chromosome in the closely related species, O. aureus", then on page 19 we read that "In the related species O. aureus, sex determination is controlled by a locus on LG3 [13,46]. ", then on page 20 we read "LG3, the largest chromosome in O. niloticus", then on page 23 that "LG3 is the largest tilapia chromosome", then on page 29 that LG3 is "where a sex determination locus is located in a sister species, O. aureus.", then on page 31 that this is the most complete assembly of "LG3, the largest chromosome in the O. niloticus karyotype (Figure 1). This chromosome carries a ZW sex locus in several species of Oreochromis [13,46]" on page 35 we read that "On LG3, a WZ sex determination region was previously identified [46] and characterized [13] in the congener species O. aureus" , then "the largest assembled chromosome in O. niloticus is LG3" on the same page. This is all rather tiresome.
* A different example: on page 44 is a description of which genetic map was used for anchoring particular LGs in the M. zebra assembly. But this is also shown in Table 3, and also described in even more detail in pages 14 and 15... and then reiterated again (without details) on page 27.
We have removed this repetition except in one case where it is needed to make a separate point.

Close
