# Supplementary material for: Chromosome-scale assemblies reveal the structural evolution of African cichlid genomes
Source: Gigascience. 2019 Apr 3;8(4):giz030. doi: 10.1093/gigascience/giz030 (PMC6447674; doi:10.1093/gigascience/giz030)
Supplement: Reviewer-1-Report-Original-Submission -- Alexander Nater — 9/7/2018 Reviewed [file giz030_reviewer-1-report-original-submission.pdf]

Reviewer Report

Title: Chromosome-scale assemblies reveal the structural evolution of African cichlid genomes

Version: Original Submission **Date:** 9/7/2018

Reviewer name: Alexander Nater

Reviewer Comments to Author:

This paper by Conte and colleagues describes two new chromosome-scale genomic assemblies of African cichlids. The authors used multiple genetic maps to anchor contigs from high-coverage PacBio sequencing and correct misassemblies. Based on these two high-quality genomes and the genetic maps, the authors performed comprehensive comparative analyses of recombination landscapes, large-scale chromosomal rearrangements, and transposable element insertions. The paper presents extensive genomic resources, which will be valuable for future studies in the field. However, the manuscript in its current form is highly descriptive and many parts of the paper are repetitive and very tedious to read. I'm convinced that the appeal of the paper for a broader readership could be improved considerably by shortening the main text and putting a focus on the biologically interesting aspects. The purely descriptive details could be presented more effectively in tables and figures or additional supporting materials. For instance, instead of the lengthy description of rearrangements, regions of unusual recombination, and putative sex determination loci, I would like to see a carefully designed summary figure, which provides the reader with a good overview of these events in the two genomes. In its current form, many interesting aspects are buried in large amounts of text that provide information of little biological relevance. Most importantly, the discussion part should be written much more concisely, as it still largely descriptive and repeats most of the information that was already provided in the results section. Here, the authors should refrain from discussing every single aspect of their results and rather focus on the biological interpretation of the most interesting findings of the study.
Minor comments:
Page 4, line 15: Define "indel" here.
Page 4, line 52: Provide reference for PacBio sequencing.
Page 10, Table 1: Improve the labelling of the table. It is not immediately clear that the numbers represent base pairs.
Page 11, line 42: "relatively complete" compared to what?
Page 11, line 35: The description here seems to imply that the final p-contigs are not phased. The p-contigs are phased within the borders of their associated haplotigs. Please provide a more detailed explanation of p-contigs and haplotigs here.
Page 11, lines 42-55: This part is unclear and should be rewritten. What are "theoretical sizes of heterozygous regions"? Do you mean the theoretical expectation of the distribution of distances between heterozygous sites? The associated Additional File A is completely unclear and needs a much more detailed explanation and legen, e.g. what is the relationship between the two x-axes (length in base pairs and recombination rate) or the two y-axes (frequency and E(r2))? How do you derive information about the completeness of haplotigs from this graph?

Page 12, line 55: Provide full genus name for A. koningsi.
Page 13, Table 3: Why does the total length differ for the four different maps, given that it includes both anchored and unanchored contigs?
Page 15, lines 34-42: Given that the anchoring is based on a combination of four different maps, is it possible that certain contigs are represented multiple times in the final assembly?
Page 16, lines 4-47: Given that all genetic maps are from inter-species crosses, what are the expectations for inter-chromosomal rearrangements that are only present in one of the two species? It seems unlikely, that the given approach would have power to detect rearrangements in such cases.
Page 16 line 34: "… at most 1% of these Lake Malawi genomes is affected by inter-chromosomal rearrangements …"
Page 23, lines 46-48: This sentence doesn't make sense without a distance qualifier, i.e. significant linkage disequilibrium over extended physical distances.
Page 26, line 59: "Only one contig longer than 1Mbp was not anchored …"
Page 27, line 7: "Contigs in the M_zebra_UMD2 assembly were primarily anchored with" or "The M_zebra_UMD2 anchoring was primarily performed with"
Page 27, lines 46-48: The suggested link between TEs and chromosomal rearrangements seems a bit far-fetched. It appears more likely to me that low recombination is facilitating the enrichment of both TEs and rearrangements due to reduced Ne and therefore reduced efficacy of selection against slightly deleterious events in these regions.
Page 27, line 56: Not clear what is meant by "orthogonal mapping technologies" here. Alternative mapping technologies?
Page 28, line 51: Genetic differentiation between what?
Page 30, line 33: Linkage group information is missing for the sex determination locus.
Page 30, lines 45-49: Rather the alleles of the sex determination system segregate in three crosses.
Page 32, lines 36-44: The connection between lack of evidence for a chromosome fusion event on LG3 and the accumulation of repetitive elements is not clear.
Page 34, line 29: What are "centromere-containing repeats" and does this refer to the ONSATA and TZSAT satellite sequences in the next sentence? Please rephrase this part.
Page 37, line 38: "will be able to purge"
Page 40, line 9: Incomplete sentence
Page 40, line 29: Omit "that"
Page 40, line 44: Check reference. Reference to PLINK software doesn't make sense in this context.

Methods

Are the methods appropriate to the aims of the study, are they well described, and are necessary controls included? Choose an item.

Conclusions

Are the conclusions adequately supported by the data shown? Choose an item.

Reporting Standards

Does the manuscript adhere to the journal's guidelines on [minimum standards of reporting?](#) Choose an item.

Choose an item.

Statistics

Are you able to assess all statistics in the manuscript, including the appropriateness of statistical tests used? Choose an item.

Quality of Written English

Please indicate the quality of language in the manuscript: Choose an item.

Declaration of Competing Interests

Please complete a declaration of competing interests, considering the following questions:

- Have you in the past five years received reimbursements, fees, funding, or salary from an organisation that may in any way gain or lose financially from the publication of this manuscript, either now or in the future?
- Do you hold any stocks or shares in an organisation that may in any way gain or lose financially from the publication of this manuscript, either now or in the future?
- Do you hold or are you currently applying for any patents relating to the content of the manuscript?
- Have you received reimbursements, fees, funding, or salary from an organization that holds or has applied for patents relating to the content of the manuscript?
- Do you have any other financial competing interests?
- Do you have any non-financial competing interests in relation to this paper?

If you can answer no to all of the above, write 'I declare that I have no competing interests' below. If your reply is yes to any, please give details below.

I declare that I have no competing interests.

I agree to the open peer review policy of the journal. I understand that my name will be included on my report to the authors and, if the manuscript is accepted for publication, my named report including any attachments I upload will be posted on the website along with the authors' responses. I agree for my report to be made available under an Open Access Creative Commons CC-BY license (http://creativecommons.org/licenses/by/4.0/). I understand that any comments which I do not wish to be included in my named report can be included as confidential comments to the editors, which will not be published.

I agree to the open peer review policy of the journal

To further support our reviewers, we have joined with Publons, where you can gain additional credit to further highlight your hard work (see: https://publons.com/journal/530/gigascience). On publication of this paper, your review will be automatically added to Publons, you can then choose whether or not to claim your Publons credit. I understand this statement.

Yes Choose an item.
