# Supplementary material for: Chromosome-scale assemblies reveal the structural evolution of African cichlid genomes
Source: Gigascience. 2019 Apr 3;8(4):giz030. doi: 10.1093/gigascience/giz030 (PMC6447674; doi:10.1093/gigascience/giz030)
Supplement: Reviewer-1-Report-Revision-1 -- Alexander Nater — 2/1/2019 Reviewed [file giz030_reviewer-1-report-revision-1.pdf]

Reviewer Report

Title: Chromosome-scale assemblies reveal the structural evolution of African cichlid genomes

Version: Revision 1 **Date:** 2/1/2019

Reviewer name: Alexander Nater

Reviewer Comments to Author:

The authors have carefully revised their manuscript and have addressed most of the issues raised in my previous review (except two points, see below). They have also added a nice summary figure, which greatly helps to get an overview of the major rearrangements detected between the two genome assemblies. The authors have also removed the most obvious redundancies in the discussion section, which considerably improves readability of the paper. However, the discussion hasn't changed much in length and remains largely descriptive. I would have really liked to see a much more concise discussion of the biologically relevant findings, but I leave it up to the editor and authors to decide if the paper would benefit from further shortening.

Remaining issues:

Page 11, line 42 - page 12, line 17, Additional File B: Unfortunately, the authors' response in the rebuttal letter doesn't help to clarify my previous issues with their approach to analyze the haplotig size distribution. In their rebuttal, the authors are now talking about the size distribution of homozygous regions in the diploid assembly and the expected size distribution of homozygous regions in an individual from a population of a given Ne. In the main text, the description of the figure in Additional File B is as follows:

"Additional File B shows the size distribution of the assembled haplotigs and how it relates to the theoretical recombination rate for several different effective population sizes (Ne)."

Not only is the description of the figure still lacking important details, they also describe the y-axis as the theoretical recombination rate, while the figure is showing E(r2), i.e. a measure of linkage disequilibrium. Apart from this confusing and contradicting description of what Additional Figure B is actually showing, I don't to see the theoretical foundation of this approach. The references provided in the main text don't really help here. As far as I can see, Additional File B compares to completely unrelated properties, i.e. the size distribution of haplotigs and the theoretical decay curves of LD versus physical distance for different effective population sizes (but these should depend on rho=Ne*r). To my knowledge, FALCON-unzip performs a purely physical phasing procedure. Therefore, the extent of LD in a population shouldn't be of any relevance for the extent of phased segments. What determines the sizes of resolved haplotypes blocks (i.e. the haplotig size distribution) is the distribution of distances between heterozygous sites in the diploid genomes (which is determined by Ne, mutation and recombination rate, as well as the level of inbreeding) compared to the distribution of read lengths and sequencing coverage. Given the insufficient description and figure labelling, I might just not correctly understand the reasoning behind the approach taken by the authors here, but I would strongly suggest omitting this part and figure from the paper as it seems conceptually flawed.

Page 40, line 7-9: "We are unsure what the reviewer means, as one of the many things that PLINK can do

is report LD statistics as described."
I was specifically referring to the second part of the sentence: "using the pedigree described above and SNP positions given in [103]." I assume that the SNP positions were not obtained from the paper describing the PLINK software.

Methods

Are the methods appropriate to the aims of the study, are they well described, and are necessary controls included? Choose an item.

Conclusions

Are the conclusions adequately supported by the data shown? Choose an item.

Reporting Standards

Does the manuscript adhere to the journal's guidelines on minimum standards of reporting? Choose an item.

Choose an item.

Statistics

Are you able to assess all statistics in the manuscript, including the appropriateness of statistical tests used? Choose an item.

Quality of Written English

Please indicate the quality of language in the manuscript: Choose an item.

Declaration of Competing Interests

Please complete a declaration of competing interests, considering the following questions:

- Have you in the past five years received reimbursements, fees, funding, or salary from an organisation that may in any way gain or lose financially from the publication of this manuscript, either now or in the future?
- Do you hold any stocks or shares in an organisation that may in any way gain or lose financially from the publication of this manuscript, either now or in the future?
- Do you hold or are you currently applying for any patents relating to the content of the manuscript?
- Have you received reimbursements, fees, funding, or salary from an organization that holds or has applied for patents relating to the content of the manuscript?
- Do you have any other financial competing interests?
- Do you have any non-financial competing interests in relation to this paper?

If you can answer no to all of the above, write 'I declare that I have no competing interests' below. If your reply is yes to any, please give details below.

I declare that I have no competing interests.

I agree to the open peer review policy of the journal. I understand that my name will be included on my report to the authors and, if the manuscript is accepted for publication, my named report including any attachments I upload will be posted on the website along with the authors' responses. I agree for my report to be made available under an Open Access Creative Commons CC-BY license (http://creativecommons.org/licenses/by/4.0/). I understand that any comments which I do not wish to be included in my named report can be included as confidential comments to the editors, which will not be published.

I agree to the open peer review policy of the journal

To further support our reviewers, we have joined with Publons, where you can gain additional credit to further highlight your hard work (see: https://publons.com/journal/530/gigascience). On publication of this paper, your review will be automatically added to Publons, you can then choose whether or not to claim your Publons credit. I understand this statement.

Yes Choose an item.
