# Supplementary material for: Chromosome-scale assemblies reveal the structural evolution of African cichlid genomes
Source: Gigascience. 2019 Apr 3;8(4):giz030. doi: 10.1093/gigascience/giz030 (PMC6447674; doi:10.1093/gigascience/giz030)
Supplement: Reviewer-2-Report-Original-Submission -- Milan Malinsky — 9/10/2018 Reviewed [file giz030_reviewer-2-report-original-submission.pdf]

Reviewer Report

Title: Chromosome-scale assemblies reveal the structural evolution of African cichlid genomes

Version: Original Submission **Date:** 9/10/2018

Reviewer name: Milan Malinsky

Reviewer Comments to Author:

Review for: "Chromosome-scale assemblies reveal the structural evolution of African cichlid genomes"
General comments:
With their high speciation rates and remarkable phenotypic diversity, African cichlid fishes serve as a model for studying a broad range of evolutionary processes. In this manuscript, the authors use two high-quality genome assemblies and five genetic maps to investigate large scale structural variation, changes in broad-scale patterns of recombination, and to compare transposable element contents of the two assemblies. I think that the work is generally well executed and provides valuable new insights. The manuscript is also clearly written and the arguments are easy to follow. My only critical comment related to the current text is its repetitiveness, with the same statements often occurring in several places (see examples below). Especially the discussion is very long and, even though it offers some additional interpretation and explanations, it reads largely as a restatement of the analyses and the results.
Overall, I congratulate the authors on their work. I have some (relatively minor) comments and suggestions that the authors should consider before publication.
Specific comments/suggestions:
Data Description - this could be expanded, especially considering the GigaScience journal's focus. For example, for the new PacBio reads, I would be interested to see the read length distribution which is a crucial factor in genome assembly, especially influencing its contiguity and the types of repetitive elements that can be assembled. Perhaps the two "new genetic maps" should also have a brief description here? I don't know what the GigaScience journal policy is, but this work is not fully reproducible until the "new genetic maps" are published. I highlighted this in the comments to the Editor.
Analyses
Page 10 - "The anchored assembly of LG3 is 54.7% repetitive, compared to repeat rate of 37% genome-wide"
Perhaps a clarification of what is meant "repetitive" would be useful. Do the numbers quoted by the authors refer to the portions of the chromosomes masked by the RepeatMasker software?
Page 11 -"To measure the completeness of the haplotigs, the theoretical sizes of heterozygous regions under null expectations of recombination rates and effective population sizes were compared to the size distribution of the haplotigs. Additional File A shows the…"
I don't understand this analysis and the associated "Additional File A". What exactly are the "haplotigs"? What determines their boundaries? Perhaps the authors should elaborate, and/or at the vary least provide a reference to the theory that predicts how large the haplotigs should be.

Page 24 - "…on the same compute cluster…"
I am not sure how the identity of the compute cluster could affect genome assembly. Read length and quality are two more likely factors that are not mentioned.
Page 24 - "M. zebra genome assembly has a noticeably larger amount of recent TE insertions."
Would be interesting to know where these insertions localised. For example, how many of these localised in gene promoters.
Discussion
Page 27 - "optical mapping, may be needed to resolve the structure of these regions in finer detail."
Again, perhaps longer reads that can span the repeats would help?
Page 29 - "…suggesting the inversion may represent an evolved difference between the Metriaclima and Aulonocara lineages."
I am not sure what exactly is meant by "an evolved difference". Isn't any difference between the lineages evolved? I suggest rephrasing this…
Page 30 - "…suggesting that the rearrangement occurred in the Lake Malawi ancestor and has maintained reduced recombination in this region across all lineages."
The Malawi species used for the maps represent only one genus of mbuna and Aulonocara - a small subset of Lake Malawi lineages. So I suggest rephrasing this, instead of "Lake Malawi ancestor" something like "ancestor of the Lake Malawi species used in our crosses".
Page 30 - "…has an XY sex determination locus on [76]"
I think there is a word missing at the end of the sentence.
Some examples of repetitiveness in the current text:
* on page 10 we learn that LG3 is "the largest and most repetitive chromosome in O. niloticus [15], and is a sex chromosome in the closely related species, O. aureus", then on page 19 we read that "In the related species O. aureus, sex determination is controlled by a locus on LG3 [13,46]. ", then on page 20 we read "LG3, the largest chromosome in O. niloticus", then on page 23 that "LG3 is the largest tilapia chromosome", then on page 29 that LG3 is "where a sex determination locus is located in a sister species, O. aureus.", then on page 31 that this is the most complete assembly of "LG3, the largest chromosome in the O. niloticus karyotype (Figure 1). This chromosome carries a ZW sex locus in several species of Oreochromis [13,46]" on page 35 we read that "On LG3, a WZ sex determination region was previously identified [46] and characterized [13] in the congener species O. aureus" , then "the largest assembled chromosome in O. niloticus is LG3" on the same page. This is all rather tiresome.
* A different example: on page 44 is a description of which genetic map was used for anchoring particular LGs in the M. zebra assembly. But this is also shown in Table 3, and also described in even more detail in pages 14 and 15… and then reiterated again (without details) on page 27.

Methods

Are the methods appropriate to the aims of the study, are they well described, and are necessary controls included? Choose an item.

Conclusions

Are the conclusions adequately supported by the data shown? Choose an item.

Reporting Standards

Does the manuscript adhere to the journal's guidelines on minimum standards of reporting? Choose an item.

Choose an item.

Statistics

Are you able to assess all statistics in the manuscript, including the appropriateness of statistical tests used? Choose an item.

Quality of Written English

Please indicate the quality of language in the manuscript: Choose an item.

Declaration of Competing Interests

Please complete a declaration of competing interests, considering the following questions:

- Have you in the past five years received reimbursements, fees, funding, or salary from an organisation that may in any way gain or lose financially from the publication of this manuscript, either now or in the future?
- Do you hold any stocks or shares in an organisation that may in any way gain or lose financially from the publication of this manuscript, either now or in the future?
- Do you hold or are you currently applying for any patents relating to the content of the manuscript?
- Have you received reimbursements, fees, funding, or salary from an organization that holds or has applied for patents relating to the content of the manuscript?
- Do you have any other financial competing interests?
- Do you have any non-financial competing interests in relation to this paper?

If you can answer no to all of the above, write 'I declare that I have no competing interests' below. If your reply is yes to any, please give details below.

I also work on Lake Malawi genome assembly, but see my work as complementary to the work presented in the current manuscript. Therefore, I declare that I have no competing interests in relation to this paper.

I agree to the open peer review policy of the journal. I understand that my name will be included on my report to the authors and, if the manuscript is accepted for publication, my named report including any attachments I upload will be posted on the website along with the authors' responses. I agree for my report to be made available under an Open Access Creative Commons CC-BY license (http://creativecommons.org/licenses/by/4.0/). I understand that any comments which I do not wish to

be included in my named report can be included as confidential comments to the editors, which will not be published.

I agree to the open peer review policy of the journal

To further support our reviewers, we have joined with Publons, where you can gain additional credit to further highlight your hard work (see: https://publons.com/journal/530/gigascience). On publication of this paper, your review will be automatically added to Publons, you can then choose whether or not to claim your Publons credit. I understand this statement.

Yes Choose an item.
